# 123rd Annual Meeting Medical Library Association, Inc. Detroit, MI May 16-19, 2023

**DOI:** 10.5195/jmla.2024.1872

**Published:** 2024-04-01

**Authors:** Ellen Aaronsen, JJ Pionke

**Affiliations:** 1 aaronson.ellen@mayo.edu, Proceedings Co-editor; Librarian, Mayo Clinic Libraries, 200 First Street SW, Rochester, MN 55905; 2 pionke@illinois.edu, Proceedings Co-editor; Applied Health Sciences Librarian, University Library, University of Illinois at Urbana-Champaign, 1408 W. Gregory, Urbana IL 61801

## INTRODUCTION

The Medical Library Association (MLA) held its 123rd annual meeting May 16-19, 2023, in Detroit, Michigan. This was also a joint meeting with the Special Libraries Association (SLA). The meeting was entitled “MLA | SLA '23: Looking Back, Forging Ahead” and utilized a hybrid model with some events in person, and some virtually. The virtual meeting was again broken into segments, all available using a variety of online platforms. Total attendance for the meeting was 1.321 with 806 attending in-person, and 515 virtually. Additional meeting content—including the meeting program and various electronic presentations from the business meetings, plenary sessions, poster sessions, and program sessions can be accessed by all meeting registrants via the MLA ‘23 website.

## OPENING SESSION (FACE TO FACE AND LIVESTREAMED)

Wednesday, May 17, 2023, 9:00 a.m. - 10:30 a.m., eastern time

- Shannon D. Jones, AHIP, FMLA, 2022/2023 MLA President
- Seema Rampersad, SLA President
- Kate Flewelling, AHIP, MLA ‘23 Cochair
- Ryan Harris, AHIP, MLA ‘23 Cochair
- Ann Cullen, Leiter Lecture Committee Member
- Michele Mason Coles, Leiter Lecture Committee Member
- Craig Robertson, Leiter Lecturer

Shannon D. Jones: Good morning! Thank you all for being with us today! I am Shannon Jones, 2022-23 MLA President. We are thrilled to welcome both our virtual audience and those of you here in Detroit.

We are excited about our conference collaboration with the Special Libraries Association. The collaboration means access to sessions on topics that transcend medical librarianship, like leadership and management, taxonomy, DEI, improving outreach and engagement, recruitment, hiring, job searching, and more. And it means the chance to network with our colleagues who face similar challenges in different settings.

We are also happy to begin our year-long celebration of MLA's 125th anniversary. The MLA 125th Retrospective Task Force has planned many fun activities for us that take place at this meeting and throughout 2023-2024.

As of this morning, we have 1,654 registrants and exhibitor representatives for MLA | SLA '23, and almost 1,200 of you are attending in-person, even if only for one day. I want especially to thank the 38 institutions who are supporting your staff with both in-person and virtual registrations.

For those of you who are unable to attend in-person, the National Program Committee and conference staff have planned yet another new meeting format with a robust and interactive virtual component taking place at the same time as we meet in Detroit. There are more livestreamed sessions, live virtual sessions in the afternoons, and, since none of us can be in two places at once, many recordings and on-demand sessions are available for later viewing.

Both of our associations will continue to experiment, so stay tuned for what may come next! We will keep learning, adjusting, and moving forward, always with our professional needs and interests in mind. And now it's my great pleasure to introduce Seema Rampersad, 2023 President of the Special Libraries Association. Please join me in giving her a warm welcome.

Seema Rampersad: Thank you, Shannon. Good morning! I am equally glad to welcome you all to our first collaborative conference! I certainly look forward to connecting with information professionals from both our organizations and to meeting new colleagues from so many US-based institutions.

Many of you have shared how great it was to connect in-person last year. Your time spent getting together with colleagues, participating in onsite sessions, and taking in the sights, sounds, and smells of New Orleans (for MLA) or Charlotte (for SLA) was a highlight of your professional year. What better place to bring us all together than “Detroit Strong” - a city filled with the glorious and soulful sound of Motown music, art, history, fabulous sightseeing opportunities, and exceptional cuisine. I noted the subtle “Power to the People” from the streets of Detroit on the cover of our Official Program for this meeting as I looked through the schedule for all the extraordinary sessions contributed by both our organizations' powerful and creative members. And I am delighted that so many exhibitors from both our organizations have come together to support us. This will be an amazing conference–and I look forward to sharing it with all of you!

### MLA Statement of Appropriate Conduct

Shannon D. Jones: Thank you, Seema. Now for a bit of housekeeping. This conference is governed by the MLA Statement of Appropriate Conduct, which you'll find on pages 6 and 7 of your Official Program.

It applies to all our activities including conferences, meetings, workshops, online forums, social media, continuing education, and all means of communication. It applies to all of us at this conference. I encourage all of you to read it in the spirit of an open, inclusive, and collaborative environment; for diversity, equity, and inclusion in professional practice; the leadership of information professionals; accessibility for all stakeholder groups; and the ethical standards that call for us to conduct all professional relationships with courtesy and respect.

### Sponsors

Please join me in giving a heartfelt welcome and thank you to our sponsors and exhibitors who have demonstrated their outstanding support for this conference, and for the value they see in information professionals. They contribute to both our organizations in so many ways and help us thrive. Exhibitors and sponsors also engage and collaborate with our members on multiple initiatives and provide education and knowledge sharing sessions that advance our profession. We are grateful for their support and invite you to take time to personally thank them for their support throughout the meeting and afterwards.

Out Bronze-level sponsors are:

- Science AAAS (American Association for the Advancement of Science)
- BMJ
- Clarivate
- Covidence
- LUCIDEA
- McGraw Hill
- OpenAthens
- Rittenhouse
- Siam
- SLACK Journals
- Springer Nature
- WILEY

Our Silver-level sponsors are:

- Couranto
- JAMA Network

Our Gold-level sponsor is:

- Elsevier

Our Platinum-level sponsor is:

- Wolters Kluwer

I'd like to welcome to the stage, representing our gold-level sponsor, Emily Singley, MLIS, Vice President, North American Library Relations at Elsevier, to share a few words with us.

[Emily Singley - 1 minute for remarks].

Shannon D. Jones: Thank you so much, Emily.

We are grateful to our Platinum-level sponsor Wolters Kluwer, who is sponsoring multiple areas of this conference. Please welcome Gareth Williams, VP of Sales for Wolters Kluwer, to the podium to share a few words with us.

[Gareth Williams – 2 minutes for remarks]

Shannon D. Jones: Thank you, Gareth, and thank you to the very supportive Wolters Kluwer team.

We are energized by the enthusiasm of our members, organizers, presenters, MLA staff, and technology partners who have been so committed in preparing this meeting. We salute the hard work and vision of the organizers for their creativity, building on the success of last year's hybrid conference to design yet another “new and improved” experience for all of us to enjoy. The words “incredibly creative” and “awesome” come to mind for both the amount of effort involved and the fabulous content that is coming your way.

### Looking Back, Forging Ahead

Shannon D. Jones: Here to talk to you about the awesome virtual conference you are about to experience, and the extra-awesome in-person conference in Detroit, please welcome your NPC23 co-chairs, Kate Flewelling and Ryan Harris.

Kate Flewelling: Thank you, Shannon. I am Kate Flewelling, your NPC ‘23 Cochair.

Ryan Harris: And I am Ryan Harris, your NPC ‘23 Cochair.

Kate Flewelling: On behalf of our SLA Program Committee Liaisons Nabi Hasan and Andy Shimp, and the entire National Program Committee, welcome to “MLA | SLA '23, Looking Back, Forging Ahead.” In keeping with our conference theme of Looking Back, Forging Ahead, we will be looking at the past and future of the Medical Library Association. But it is also instructive to look at the past and current occupants of this conference location. People have been coming together on the Detroit River for thousands of years and we are on the ancestral and occupied home of the Anishinaabe nations. This land is currently governed by the 1807 Treaty of Detroit between the United States and the Odawa (Ottawa), Ojibwe (Chippewa), Wyandot, and Potawatomi nations. Today, Michigan contains 12 federally recognized tribes represented by The Inter-Tribal Council of Michigan, Inc. Land acknowledgment is a small part of supporting and celebrating Indigenous communities. In addition to this acknowledgment, we hope you will join us in donating to the North American Indian Association of Detroit, the American Indian Library Association, or a local group in your area led by Native people. Beyond this meeting, use resources from the Native Governance Center and others to research on whose land you occupy and identify concrete action items to humbly support Native-lead initiatives.

### Recognition

Ryan Harris: Thank you, Kate. When we invited you all to this conference in August, it was just days after MLA and SLA had signed an agreement to collaborate on this meeting. It was time to pivot, yet again!

Our NPC ‘23 team has been extraordinary and has selected programming to appeal to both MLA and SLA members. Please join Kate and me in recognizing them and the dozens of content reviewers who reviewed the hundreds of submissions for this meeting. You can also find all names on page 10 of your Official Program.

Kate Flewelling: We also want to recognize the two special program committees who assembled impressive Collection Development and Leadership symposia for this conference. We look forward to seeing these sessions being held here in Detroit throughout the week, with several of each being livestreamed to virtual attendees. You can also find their names on page 10 of your Official Program.

Those of us here in Detroit have been amply prepared by our extraordinary Local Assistance Committee (LAC), led by LAC Chair LaVentra E. Danquah, AHIP. From blog posts to dining guide, transportation advice to volunteer wrangling, the LAC has done an awesome job in sharing information about our host city. LaVentra, many thanks to you and your whole team.

Ryan Harris: When you work so closely with the MCI staff conference team to organize this hybrid event, you get a real appreciation for the breadth and complexity of the task. Please join us in showing our appreciation for what they do. Thank you.

### Conference Overview

Kate Flewelling: Many of you have already been exploring the online planner. The planner and the app are the easiest ways to navigate and keep track of the large number of sessions at this conference. Just so you know, the Online Planner home screen will change throughout the conference. We've always been a ribbon crowd, so don't forget to add virtual ribbons to your profile!

For our virtual attendees and virtual sessions: use time zone support! All live sessions are then shown in your time zone so you can plan your time effectively and not miss sessions you want to see. If you are attending in person, revert your preferred time zone to Eastern time while you are onsite and use the app to keep track of your schedule.

Ryan Harris: The app will also let you play MLA | SLA '23 Quest—whether virtual or in-person—and keep track of everything onsite you want to see. You can easily take notes during sessions to download later. The app also hosts the audience response system, so you can ask questions throughout many sessions, whether virtual or in person. Look for icons at the bottom of the App.

There's no doubt: MLA | SLA '23 has a lot of programming, and I can't wait to get started this afternoon.

We really thank our speakers and contributed content presenters for being so flexible and for going the extra mile to make this conference a success for both our virtual and in-person attendees. Your participation is essential to strengthening our community.

Ryan Harris: A final few words: make sure to not overdo, please take breaks, have snacks, or visit the Quiet/Recharge room, and make sure to spend time with your frolleagues!

Kate Flewelling: Ryan, thank you—I second all those suggestions! It is now my great honor and pleasure to transition to the next and highly anticipated segment of today's program: the MLA/NLM Joseph Leiter Lecture. Please join me in welcoming Leiter Lecture Committee Member Ann Cullen to the podium to introduce our speaker.

### Joseph Leiter NLM/MLA Lecture

Ann Cullen: The Joseph Leiter NLM/MLA Lectureship was established in 1983 to stimulate intellectual liaison between MLA and the National Library of Medicine (NLM). Lectures are chosen for their ability to discuss subjects related to biomedical communications. As is our tradition, there will be a question-and-answer session after the lecture. This is being managed through the meeting app, where you may enter your own question, or like and upvote someone else's question. Index cards are available for those not using the conference app.

This year's speaker, Craig Robertson, is an associate professor of communication studies at Northeastern University. He has a PhD from the Institute of Communication Research at the University of Illinois, Urbana-Champaign. An award-winning author, Robertson researches the history of information. He focuses on the relationship between paper and information, specifically how the recording, classification, and storage of information on paper affects not only who gets to handle and access information, but also how information is conceptualized as something people can use. I discovered his work five years ago when I was redesigning and started teaching Simmons University's “Special Libraries” course and found it profoundly transformative in my understanding of the history of our profession. The Atlantic described his most recent book, *The Filing Cabinet: A Vertical History of Information*, as a “captivating history.” In addition to writing two monographs and editing two volumes of essays, Robertson's research has been published and reviewed across a wide range of fields and disciplines including communication studies, design studies, library and information science, history, surveillance studies, immigration studies, and legal history and policy. An internationally recognized scholar, Robertson has been interviewed by multiple media outlets including the New York Times, National Geographic, NPR, BBC, and the Australian Broadcasting Corporation.

Please join me in welcoming my colleague and friend, the amazing Craig Robertson.

[View the Joseph Leiter NLM/MLA Lecture: “The Filing Cabinet: A Vertical History of Information” by Craig Robertson, PhD in the Plenary Sessions section. The session was livestreamed for virtual attendees and is available on demand.]

### Conclusion to Opening Session

Shannon D. Jones: Let's give Dr. Robinson another round of applause for his amazing presentation. I never knew some of the things that he shared. Thank you for an illuminating, engaging, and insightful history of information.

I also want to thank you all for coming to the kickoff this morning. We look forward to spending more time with you here on site and spending time with our attendees who are with us in the virtual landscape. They're going to be a lot of fun events and a lot of engaging events. And I hope you enjoy each and every one of them that you get to attend.

Dr. Robertson is going to be available to sign copies of his book in the back of the room. We hope that you all will go and support him and get to actually meet him in person, but also get to support our local bookstores, source booksellers who is here providing? Well, not providing because that'll make you thank you. Getting a free coffee, selling his books in the back of the room. With that, this session is concluded. Have a good meeting!

## MLA '23 ANNUAL BUSINESS MEETING AND OUTGOING PRESIDENTIAL ADDRESS

Thursday, May 25, 2023, 2:00-4:00 p.m., eastern time

MLA Board Members:

- Past-President Kris Alpi
- Executive Director Kevin Baliozian
- President-Elect Amy Blevins
- Secretary Heather Holmes
- Treasurer Dale Prince
- Directors:
  - Emily Hurst
  - Janna Lawrence
  - Brenda Linares
  - Tony Nguyen
- Community Council Chair Adela Justice
- Chapter Council Chair Keith Pickett

Incoming MLA Board members

- Andy Hickner
- Irene (Rena) Machowa Lubker
- Tamara Nelson
- Dede Rios

Others

- Kate Corcoran
- Erin Fuller
- Maria Lopez

### Agenda

Shannon D. Jones: Good afternoon, everyone. I am Shannon Jones, your 2023 MLA President for about 40 more minutes. It's my pleasure today to welcome you to the 122nd Annual Business Meeting of the Medical Library Association. Last year more than 500 members attended our third virtual business meeting, continuing to surpass our previous attendance records from in-person business meetings. We are thrilled that so many of you attended, and that being virtual, is helping us to be more inclusive, so that many more of our members can attend. It's wonderful to see so many of you all participating with us today.

Here's the agenda for today. We're going to start this meeting today with my Presidential Address so that I can tell you what I've been doing this year. Then we'll move on to the business portion of the meeting which will include the presentation of your current MLA Board of Directors, reports by the MLA Treasurer and Executive Director, election results in the presentation of your new MLA Board, and this year we will address proposed revisions to the MLA Bylaws. New this year, the incoming President, Inaugural Address will be held as a separate virtual meeting for separate virtual events on June 21. More details about that event will be shared later in this session.

### MLA's Statement of Appropriate Conduct

Before we get started today, here are a few guidelines. All MLA gatherings and interactions need to respect MLA's Code of Appropriate Conduct. Please consult it online. Someone may be putting it in the chat box, too. If you need to report a violation, there is a link to do so on the web page. We are using Zoom Webinar today, with which, by now you are all likely familiar with after three years of COVID. We will be using Zoom polls for voting as well as the “raise your hand” feature for official business. We'll walk you through the procedures in a few minutes. We will not be using the Q&A feature today. Parliamentarian Chris Schaffer will share the formal business meeting process for raising an issue soon. During the year we offer topical open forms on many areas of MLA to invite conversations and questions and offer a better experience for dialogue. Feel free to use the chat, but please note that we will not be monitoring the chat for questions. If you have a question that you would like to address after the meeting, please email president@mailmlahq.org. Now we are going to shift to my Presidential Address.

### Outgoing Presidential Address

Shannon D. Jones: I am going to share my journey as the 22-23 MLA President. I'm Shannon Jones. I am also the Director of Libraries at the Medical University of South Carolina, and also the Director of Region 2 of the Network of the National Library of Medicine. My favorite quote, often credited to An African proverb is, “It takes a village to raise a child.” In essence, this quote communicates to support a leader who needs to show up and to stand in their leadership role. Those who have served as leaders in any capacity know that know the commitment and support required for a leader to succeed. Therefore, as I did last May, I'm going to start this address with my village.

My sincere gratitude and respect go out to those who walked with me during my presidential journey. Those who showed me kindness offered words of affirmation and encouragement, stood in my place when I could not, said yes when I asked for assistance, pushed me when imposter syndrome showed its ugly head. Your patience and understanding and grace were appreciated.

The first group that I will thank is my home team, the Medical University of South Carolina Libraries staff. During my presidency, it was the perfect storm of events. We became the newest Regional Medical Library, as part of the NLM. We were displaced due to COVID-19, we were displaced because of our renovation, and I also completed my doctorate. I was away a lot, and we had a lot going on, so I could not do this without their support and their commitment to keep our ship afloat while I took care of MLA business.

I want to thank all the people that you see on the screen. The next group that I want to thank is the mentors who modeled the way for me. A quote from John C. Maxwell says, “A leader knows the way, goes away and shows the way.” A sincere thanks to Tom Basler, Sandra Franklin, Teresa Knott, Sandra Martin Beverly Murphy, Jean Shipman, M.J. Tooey, and John E. Ulmschneider. I would not be where I am today without the leaders who pushed, encouraged, loved, supported, and challenged me to show up as the best version of myself.

The next group is the African American Medical Librarians Alliance Caucus, or, as we affectionately note, call ourselves, AAMLA. I offer them heartfelt thanks for love, encouragement, and support, and most importantly, I thank them for reminding me that this Presidency was not just about me. It was about the collective we. I'm so proud to stand amongst this group, and as our T-shirt says in this picture, “I'm so proud to do it for the culture.”

The next group that I want to thank is the MLA Reads family. Thanks to the planning team for keeping the program rolling so that I could focus on my MLA work. If any of you have ever participated in one of the discussions, you know that it takes a lot of work to execute a book club where you have 150 people participating. I am grateful to Kelsea Bartley, Melissa De Santis, Ryan Harris, Don Jason, Tamara Nelson, Dede Rios, and Jenny Pannebacker for their tireless dedication to executing the MLA Reads program. I could not have, you know, made this happen this year without their commitment, and they're stepping in and stepping up and leading it so that I could step back.

The next group that I want to thank is our pioneering Black library leaders in health sciences. Their advocacy, their resilience, and their power allowed me to find my place in the profession. I stand in reverence to luminaries that you see on the screen that came before me. Josephine G. Morton is not pictured on the screen. I couldn't find a picture of her, but she was the first Black librarian to attend an MLA conference in 1940. Arlene May was the first Black librarian elected to the MLA Board of Directors in 1979. Dr. Gwendolen Cruzat was the first Black librarian to deliver the prestigious Janet Doe lecture in 1979. I was able to meet Dr. Cruzat at the MLA | SLA '23 meeting. Just standing in her presence was the absolute highlight of my conference. You know I've written about her in my dissertation, I've read about her, and to actually meet her and talk to her was wonderful. She is sharp and she had a lot of good things to say (and a lot of funny things to say). Madeline V. Taylor was instrumental in reorganizing and restructuring the MLA to allow us regional chapters to become a part of the association. Finally, we all know Beverly Murphy, who served as MLA's first Black President from 2018 to 2019. I know all too well that representation matters—that you cannot be what you cannot see. Because of them, I could see, believe, and become a leader in health sciences librarianship. So, I thank them, too.

The next group is the MLA headquarters team because none of us leaders can do this without the work of the MLA team. I am grateful for them for everything they do to help MLA thrive and also to help us do the work of the association. I'm very grateful for the team that you see on the screen. You may not know everyone, but, believe me, they are behind the scenes, working very hard for the association, and on our behalf.

The next group is the '22-23 Board of Directors. We had a fantastic year. We have done important work. We've made meaningful decisions, and we have fun doing it. We like to laugh. Everybody on the screen loves to laugh, and we've all been colleagues for a number of years. I'm so very grateful for their ingenuity. I'm grateful for their laughter. I'm grateful for their insight and for their overall commitment to MLA.

Then, last and certainly not least, I am eternally grateful for each one of you who showed up today. I'm grateful for your continued commitment to MLA and just for you, continuing to show up and continuing to do this important work that we must do on behalf of the association.

One of the essential parts of the presidential journey is the opportunity to engage with MLA members and health sciences, colleagues, at the regional, at the national, and in some cases the international level. I've had the opportunity to provide MLA updates at in-person and virtual chapter meetings. I also had the opportunity to travel to Ontario, Canada, to represent MLA at the Canadian Health Library Association Conference last July. And then I traveled one state over to Charlotte, North Carolina, where I attended the SLA Annual Conference. And there's an IKEA in Charlotte. So also got to go to IKEA because it is one of my favorite places. Then I was invited to speak in September, to represent MLA at the Lindberg-King Lecture and Scientific Symposium. The Symposium honors the legacy and contributions of the former NLM Director, Donald A. B. Lindberg MD. And the former NLA Deputy Director for Research and Education, Donald West King, MD. Many of you know that for years Dr. Lindberg supported the mission and goals of the Medical Library Association and was a strong champion and advocate for health sciences librarians throughout the nation and in the world. I'm one of those librarians who benefited from Dr. Lindberg's leadership, service, and advocacy. The day of this session was September 1. It was the 20th anniversary of my arrival in Bethesda, and to NLM. As a member of the 2002 cohort of NLM Associates. And while I was at NLM you know launched my career as a health sciences librarian. I met some really wonderful professionals. I had the opportunity to participate in meaningful projects, and I was exposed to tools and information that made my journey as a health sciences librarian easier. And so just you know, it was a pleasure to represent MLA in that space. You all can watch that whole program. It was a day long program. The recording is on YouTube. And at some point, I will put the link in the chat box. You can go to the next slide Kevin. Then it's our advocacy work. You all know that role for MLA members and leaders for MLA members and our association leaders alike. Whether we do it on the local, or the regional, or the national levels. We know that advocacy works, but it takes time, and it's not an overnight process. In my inaugural address, I asked you to do like Beyonce Knowles, and to get information. And you all did in a variety of ways. While advocacy is happening throughout MLA, my goal today is to amplify a few of the areas to show the dearth of work and areas in which the association is engaged.

You can go to the next slide. The first is the MLA issue Advocating for Authorship Librarians and Information Professionals as authors on Evidence, Synthesis, Publications campaign which encourages guideline associations, journal editors, peer reviewers, and collaborators to ensure that all authors who met authorship criteria established by the International Committee of Medical Journal editors receive authorship credit for their contributions. The people you see on the screen Kris Alpi, Emily Brennan, and Heather Homes, were instrumental in writing this statement. And kudos to Emily Brennan because she was actually able to use this statement to get authorship with a society that had never granted a librarian authorship credit before. She blazed that trail for us. We say kudos to her because I know she put in a lot of work on the publication that she was initially not allowed to be an author on, and that association did change their perspective. So, you all have this statement so that if you're ever in that situation, you can go forth and have a piece of paper to show that there is a reason for you to have it.

We also appointed some task forces and work groups. One of the first actions that I took as President was to write solidarity emails to MLA members who lived in who lived or either worked in Buffalo, New York, or the State of Texas, due to mass shootings on May 14, 2022, in Buffalo, and on May 24, 2022, in Uvalde, Texas. As President, messages of comfort were sent to members just to express our express sorrow and solidarity, which is always the right thing to do. However, as a community we have to do more than write statements. We don't want to be performative in our social justice efforts. We endeavor this year to take action. For us, taking action resulted in the formation of multiple working groups. The first group, oh okay, good, you are there. The MLA role in Societal Issues Task Force was charged with developing and recommending guidelines, objectives, and processes to address social justice and human rights issues. The Board of Directors approved their statement in May 2023. You can see the members of the task force on the screen. So please give them kudos to them for serving on the group and completing their work. And the outcome of their work will be shared with the membership very soon. And I think you all will be impressed and pleased with their work.

The next group was the MLA Social Justice Statement on Work Group, who is reviewing MLA's Core Values Code of Ethics, previously released statements, and existing literature to develop an overarching statement. This statement will integrate guidelines, objectives, and processes created by the MLA Role of Society Issues Task Force. The goal is to create or to craft a collective social justice statement, to provide meaningful recommendations for the Association, to respond to societal situations suggest internal and external resources for action, and to intentionally align with MLA's 501(c)(3) status. A final statement from the group of individuals that you see on the screen should be available this summer. And so, I look forward to the outcomes of their work.

The next group is the Censorship of Library Practices Work Group. This group is working to develop guidance that provides librarians with the tools and resources to keep up with what is happening in their states, and how to be positive. That's reactive, when issues arise. A final toolkit will be ready for the Board's review in June. And once that happens, you will also all be notified, and I imagine through some *MLAConnect* articles. Please give this group a round of applause. Thank each one of these groups for the work that they are doing, because these are areas in which the membership has been pushing MLA. Advocacy works, but it takes time. Keep pushing, and you may be asked to serve on a group when you push, but it will be all for the greater good of MLA. We can go on to the next slide.

The next group, and you know this is my last segment. You know an important part of my presidency was wellness and wellbeing, my own and that of MLA members. And you know, one of the things that COVID-19 has taught us in that it did for many of us was encouraged us to reconsider the relationship that we have with work. Specifically rethinking and reshaping the sacrifices that we are willing to make for our jobs. And so, we came up with the Be Well MLA initiative. And the people that you see on the screen are the individuals that I invited out through the right emails. Or I sent a tweet out and said, hey, who are our experts and wellness and the association, or who is passionate about wellness. And these were the people who raised their hand, or they were volunteered by one of their colleagues that they were someone who was interested. From September '22 to April ‘23, the Be Well team invited MLA members to participate in the Be Well Wednesday's Webinar series. And in these sessions are our panelists shared lessons, learned strategies and tips about various wellness topics and which all this culminated in Detroit. I will say that if you all are into yoga, Kelsea Bartley has a really nice chair yoga recording in the meeting container or Events Scribe. Whatever the thing is called. Kelsey has a really nice recording there. The recordings for all of the Be Well sessions are on MLANET. So, you can go and watch those. Last year nearly 800 attendees participated in the webinar series. We recorded each session except for the book discussions. You can see the topics that we cover. By all means go back, watch those, use those in your wellness process. And you know I'm especially excited because we will continue this work in fall 2023. So, if wellness is important to you, please reach out to us as we are planning the next phase of the project.

It was my pleasure to serve as your President. Last year I shared with you that I would approach my association work through the lens of radical empathy as my new normal. In all my efforts, I've tried to be the light in my interactions while representing the Association to the best of my ability. I look forward to continuing to contribute to MLA initiatives as the Immediate Past-President, while supporting my dear friend and colleague, President-Elect Amy Blevins, as she takes the helm of what I believe is the best association in the world.

### Business Meeting

Shannon D. Jones: With that we will get the annual business meeting started! To get us started, I'd like to recognize Chris Schaffer, MLA's Parliamentarian. Chris will assist us with the business portion of our meeting. Chris, I turn it over to you.

Christopher Shaffer: Hello, fellow MLA members! This is our fourth electronic business meeting. So, we're getting to be pros at this. Before I get started in my official role, let's practice some of the Zoom Webinar features to make sure you're familiar with them before the real deal. First, let's practice hand raising. During the meeting, there will be times when you will have the opportunity to raise your hand to speak to an issue on the floor in the context of the official business of the meeting. Here's the fine print.

A written motion is introduced. The presiding officer restates the motion and asks if there is discussion. Members wishing to address the motion can raise their hand, as you can see on the screen there. Staff will let the presiding officer know you wish to address the motion. And the presiding officer will give you permission to speak for up to two minutes. And you'll be temporarily promoted to be a panelist in the webinar to do that. Please announce yourself before you ask your questions or raise an objection. So please let us know your name and your institution, and this process will be repeated until all those who have raised their hand have had two minutes to speak to the motion on the floor. Then after that anyone wishing to speak a second time may raise their hand and have one additional minute to speak. And then the presiding officer will repeat the motion and call for the vote which will happen after the meeting, electronically. As to the specifics of how to raise hand and speak in a Zoom Webinar, we'll monitor who has raised their hand, share this information with the President, who will then call on you by inviting you to speak by name. You'll see a button appear on your screen prompting you to turn your microphone on, and you may optionally turn on your video as well. Once you do, please announce your name and institution and speak. And once you've spoken, please use the raise hand feature to lower your hand. Now let's go through the specifics of voting. When the presiding officer calls for a vote on a motion which may not happen today, I will note that the vote on the Bylaws motions will happen electronically following this meeting, you will see a poll up here on the screen. You vote YES, NO, or ABSTAIN. And don't forget to press the submit button to make sure your vote will go in. You'll have 2 minutes to submit your vote, and after the vote the result will appear on the screen and will also be announced by the sergeant-at-arms.

Now let's practice. A poll will appear on your screen. Go ahead and vote. We're going to keep this one short—30 seconds.

Shannon D. Jones: I'll just say I oppose chocolate.

Christopher Shaffer: [Laughs] Here's the results of the votes. Well done. We got a good number of people there. Now that you know how to raise your hand and how to vote, let me share with you that we may not actually have to do that today. we are going to be doing some things through unanimous consent which is permitted by Roberts rules of order. which removes the need for discussion and a full vote. Any member present may object to unanimous consent and require the President to open the floor for discussion and put the question to the members for a vote. Today we plan to use unanimous consent, and we'll use the raise your hand feature to allow any member who wishes to register an objection. All new business must be presented by a member in the form of a written motion and submitted to the President as announced on May 11. Members were strongly encouraged to submit motions in advance of the meeting, and the only motions that were submitted were the Bylaws motion submitted by the Board, and now I'll turn it back over to Shannon.

Shannon D. Jones: Thank you, Kris, and MLA headquarters staff. I think that if we have an ice cream social next year. Y'all got a lot of flavors, so make sure you check in the chat box. I am pleased to now introduce Linné Girouard, AHIP, FMLA, Sergeant-at-Arms, who will assist us with counting of the quorum.

Linne Girouard: Thank you, Shannon. I believe there are 233 participants. We have a quorum.

Shannon D. Jones: Thank you. There being more than 200 voting members present, we have a quorum. I now call our meeting to order. I would like to now welcome Heather Homes, MLA Staff, MLA Secretary. Hi, Heather, who's right down the hall.

Heather Holmes: Hi, Shannon. Yeah, I am representing South Carolina here, as well as MLA. Great to be joining you and all the membership today. Just as MLA's secretary I get the great joy (and I don't mean that tongue-in-cheek) of getting to review the Board meeting minutes. It's always nice to get a reminder of all the important work that we've done and that we've worked on. Our next secretary will get to take over this exciting role. I also get to present the agenda for the 2023 business meeting which you can now see on your screen, and it's also available on MLANET. And we've already done the first three items, so we're almost done—just kidding.

Shannon D. Jones: Thank you, Chris, Linne, and Heather. Please stick around as we meet. We may need to call on you. Now I am very pleased to welcome our Executive Director, Kevin Baliozian. Hello Kevin!

### Presentation of the 2023-2024 Board of Directors

Kevin Baliozian: Hi, Shannon! Hello everyone! Great to be here. I have the honor of introducing your 2022-2023 MLA Board of Directors. As you saw earlier, we have an absolutely wonderful group of leaders. This Board of Directors has expertly led us through the third year—I believe it's three years now—of the pandemic, along with lots of issues that have significantly impacted you and MLA. Thank you very much. Shannon, you've been our wonderful leader this last year. We also have Amy Blevins as President-Elect. Kris Alpi, as Immediate Past-President. Dale Prince as our wonderful Treasurer. Thank you to Tara Douglas Williams, to Heather Holmes, our Secretary, to Emily Hurst, to Adela Justice, who is the Council, Community Council Chair, Janna Lawrence, Brenda Linares, Tony Nguyen, Keith Pickett, who joined the Board following the vacancy of P. J. Grier (he's been on the Board for a month or two, so welcome to the Board!), and I'm on the Board as your Executive Director. Here are pictures of everyone and the next one is a picture we just took in Detroit at the MLA | SLA '23 conference. Look at that, everybody's looking wonderful and very happy. That was before the conference, you should have seen how happy they looked after the conference! Back to you.

Shannon D. Jones: Thank you. And I will note that one of the most beautiful things about this picture that you all is that these are our amazing people, and you'll see from the new Board that we are welcoming the most diverse Board we have ever had in MLA history with our new additions to the Board once we get to that phase. But that makes me so excited about where we are. where we're going in our DEI journey, when it comes to representation. I'm excited about that. Thank you so much, Kevin.

### Treasurer's Report

Shannon D. Jones: Now please join me in welcoming my favorite treasurer, J. Dale Prince. Your MLA Treasurer, and my colleague and friend who will spend the next few minutes updating us on our finances. Hi, Dale. I sure hope you got on the bow tie today.

Dale Prince: I do, Shannon, and thank you. Thank you. I want everyone to look at this picture the picture that was just up and note that that's exactly what I looked like before I became Treasurer. Don't believe him. And now look at the color of my hair. See?

As your Treasurer, I share the financial stewardship of our organization with Kevin, MLA's Executive Director and rely on the insights and review of the Finance Committee to ensure the Board of Directors can exercise its duty of care.

The Finance Committee was really busy this past year. We:

- reviewed the budget and financials prepared by the MLA staff
- worked with the MLA independent auditors to ensure compliance and best practice
- set MLA's investment strategy with the MLA's independent financial advisor
- examined key MLA pricing models
- analyzed contract terms with MLA's management company, MCI U.S.A.
- ensured the continued financial sustainability of MLA during COVID-19
- reviewed and approved funding requests from caucuses and domain hubs in collaboration with representatives of the Community Council.

I'm grateful to have been supported in my role as Treasurer by an experienced group of colleagues.

Please take some time to thank the members of the Finance Committee:

- Kristine Alpi, MLA's Immediate Past President
- Teresa Knott, an MLA member at large
- Janna Lawrence, a member of the Board
- Deborah Lauseng, an MLA member at large
- Tony Nguyen, MLA Treasurer-Elect
- Kevin Baliozian, MLA Executive Director
- Kristie Hammill, MLA's Director of Finance

We're going to start by reviewing financial numbers.

- 2019 was the last year prior to COVID-19, so it's useful to include it for comparative purposes along with the 2020 through 2022 COVID years and the 2023 budget.
- 2019 through 2021 numbers have been audited.
- 2022 numbers are pre-audit, so they may be adjusted later by MLA's audit firm.

The top 3 lines are what we refer to as “operating”: that includes all financial activities except investment revenues and disbursements from the MLA endowment for awards and grants.

- Revenues decreased in 2020 and 2021 because of the loss of the in-person conference, partially recovered in 2022, and are budgeted to significantly increase in 2023, higher than in the 2019 base year.
- Expenses remain high, because the cost of virtual delivery is high, and we opted not to cut programs. That in turn, creates a net operating loss for all 5 years, though significantly reduced to near breakeven in the 2023B budget year.
- The non-operating net margin is the difference between the financial revenue of our endowment and reserves and the disbursements from the endowment fund.
- As you can see, MLA has an excellent financial performance from 2019, or pardon me, had excellent financial performance from 2019 to 2021 more than offsetting the operating losses in those years.
- The financial markets were down in 2022, so the non-operating loss exacerbates the operating loss rather than offsets it for that year. When you add operational with non-operational, you get the net change in assets. In the 2019 to 2022 period, the total drop in net assets is $650,000. Though this is a large number, MLA's financial strength is more than able to absorb this extraordinary financial disruption due to COVID-19, and we are set for rebuilding net assets in the coming years.

The prior slide was the accounting review, based on applying general accounting principles.

For a clear understanding of MLA's operating performance, we have adjusted the numbers to represent an adjusted EBIDTA. I regret that I won't be saying those letters anymore. Also known as Earnings Before Interest, Depreciation, Taxes, and Amortization, amortization. The adjustments include the list on the right side of the graph which are important to note.

- We excluded the investment revenue. It's real, but it doesn't reflect operations.
- We excluded the investment in EFTS and the associated depreciation. This is a technical adjustment to reflect EBIDTA. Note that the investment of $157,000 in the technology platform supporting efts was fully funded by participating libraries.
- We also excluded the investment in education. Note that we invested $191,000 in new education programs, as part of the MLA strategic initiative.
- MLA received $233,000 from the Paycheck Protection Program from the Federal CARES Act, which was forgiven. This is actual money that we will not have to pay back, and it was designed to support associations in our situation. We are grateful for it.
- We removed an additional $43,000 in miscellaneous investments.

Take a look at the graph.

- In 2018 we were balanced.
- In 2019 we had a $275,000 deficit, which we had intended to address over the following years. Then COVID-19 hit.
- 2020 was the first year of COVID, with obvious financial damage, mostly linked to can cancellation of the in-person conference in 2021. The COVID negative effect was even higher. Exhibitors did not value the virtual conference, so they pulled back in year 2.
- In 2022, we showed a partial recovery from COVID disruption and in 2023 our budget is positive, with a $136,000 margin, should we miss the mark. This is an extraordinary turnaround.
- As a result of MLA's continuing to invest in its members and its future, we are a stronger association in 2023 than we were in 2019, though with lower net assets.

MLA net assets represent the cumulative net revenues and losses over the years. 3 million dollars is a strong number, split between the endowment in blue and reserves in orange. An increase or decrease in the value of our investments directly affects that number. Our reserves did decrease, and it is essential to rebuild them over the next 5 to 10 years.

Let's understand the foundation of MLA's 2023 turnaround. An overall price increase of MLA membership dues to keep up with the inflation. We realize that these increases are challenging for you, our members, to absorb. They are, however, essential for MLA to maintain the level and quality of services and are contributing an additional $100,000 to 2023. This year MLA membership counts are at 2022 levels, and we hope that we may exceed them. The return to in-person conferences is an essential part of MLA's financial sustainability. We expect to increase the contribution margin of the 2023 conference by $150,000, though we may miss the $300,000 improvement mark set in the budget. The EFTS program is a significant contributor to MLA, margin as well an essential service, as well as an essential service to libraries. MLA's investment in the new technology platform has been successful on all fronts. MLA continuing education continues to grow. 2022 was the first year CE positively contributed to MLA margin, and 2023 builds on that trend. Note that MLA did not increase CE prices in 2023.

Analyze turnaround is a result of multi-year strategy of a multi-year strategy and MLA is working on the following key areas: expansion of MLA's reach in both specific health scientist segments and selectively in segments beyond health sciences, where the value of diversity of practice can benefit our core constituents. We have to continue to rebuild and even grow our in-person conference presence, which is essential to high quality interactions and learning, and to vendor support through exhibits and sponsorships. As mentioned earlier, continuing education is an important pillar of both mission and finances. We need to further position MLA as the learning destination for specific segments of members and non-members. Managing our overall cost is also essential, and those are directly related to the scope of what MLA does. To reduce costs, we have to prioritize programs that contribute to the mission. This means that we should consider eliminating those that are marginal contributors, especially if they are high in cost. Ultimately, MLA must address it's many unfunded initiatives. So recapping, MLA has a long-term strategy to balance revenues and expenses and rebuild net assets.

Regarding MLA investments, and even taking into account the large $508,000 lost in 2022, the average annual gain over the last 5 years is $175,000 per year. Our cash position is strong. We also applied for and received a $500,000 COVID-19 economic injury, disaster, loan or EIDL in 2021 and a 2.75% fixed interest rate. That increased our flexibility. We are paying it down monthly and can reimburse it at any time over the 25 years of its term.

In summary, I'm happy to report that the state of MLA finances remains strong.

Shannon D. Jones: Thank you, Dale. The Board is grateful for your diligent stewardship of the entire financial team. And just, you know, kudos to you all for just making sure that we are sustainable and a viable association, and that there will be an MLA here in 5 to 10 years, and so thank you so much.

### Executive Director's Report

Shannon D. Jones: The next order of business is the Executive Director's report, and so I welcome Kevin back to the virtual stage.

Kevin Baliozian: Thank you, Shannon. And thank you, Dale, for your wonderful report. So here we go, the Executive Director's report.

We'll talk about all of the annual reports which you should absolutely read, and we'll talk about that in a couple of slides. But when it comes to the part that headquarters does, there are all these different sections in the annual report, and there's lots of great information. If you're interested in just the data behind everything, that is a good reference in place to go to, and you can go back multiple years as well.

Quick picture on membership and other important metrics as of May 19, 2023.

- As of a few weeks ago, we had 2,346 members. Obviously, we get more members throughout the year. Our estimate of membership counts for this year, which peaks August 30, is to be at 2,500 members. We also have what we refer to as customers, a non-member, people who interact with MLA in various ways: take a course register to the conference, sign on to EFTS. But they have an account that they log on to MLANET to do something other than being a member. That's 5,000 additional people over the last 3 years. Now those individuals may not be active every year. Those are people who may take an NLM course, and then get a CE credit from us.
- Our community has 7,500 people and that's a very good number to have. It's a very diverse group, as well.
- Eighty-nine percent of members participate in caucuses as of May 19. That's a staggering number. I mean, let's run it up to 90%. You're not just a member, you're a member and you're joining communities. And if you are joining a caucus, you're joining just under 5 caucuses on average, 4.8 caucuses. So, that's quite a quite a bit. That number has steadily increased since we moved from the sections to the caucuses, because we removed the paywall where you have to pay an additional $15-$20 depending on the caucus, depending on section, to join. So that has affected the number of people who joined caucuses, and how many caucuses members join. So that's wonderful.
- The last section of actual stats from the conference last week. We had 803 in-person attendees, which includes exhibitors. We used to have only an in-person conference, and that was about 1,050 attendees. We have 300 and 300 attendees, or 250 fewer than that. But we do have 514 virtual attendees so far. And we know that with the virtual, since a lot of it is on demand, we're going to get more people. Right now, we have 1,317 and we expect that that number should go up to 1,400 in the next few weeks and month. So, all in all, that combination of virtual and in-person was, and has been, very successful.

I want to just go to a survey that most of you or certainly all of you were asked to reply to last September, which was an engagement index survey. That was a survey done by the management company MCI with 50 other associations. And so, it provided us with benchmarking information to see how MLA is doing with respect to other associations, similar associations in the US and globally. The engagement index is a number that is the result of how many questions are answered. You can see that the MLA's Engagement Index is 78. It's in line with similar organizations. It's considered to be in the moderate category. That includes members and non-members. And when you look at professional associations of MLA's size, that is a very respectable number. That of course, we strive to increase but is certainly great. We have an engaged community.

The Net Promoter Score is also a concept that is a standard it takes the difference between the promoters and the attractors and gives that number. But you want to have more people who promote your association that you say do not come to MLA. That is a positive number. That's good. 17. We'd like to see that higher, but we generally have a robust 41% of our participants in the survey who actively suggest that promote MLA as a go to place. We do have 24% and, you know, that's something we need to work on. This is rather small.

I will just highlight a few items about the data around the performance evaluation. These are the different types of activities. And they're very generic across multiple associations. You're not going to see names of MLA programs here. You're going to see general things. And so, what do people care about? The absolute top-ranking thing is subject matter expertise at 87%. And that includes 88% of non-members who clearly look at MLA for knowledge. The second one is reputation at 83%, you know. And you can see our we're blue and in gray as other associations. You can see these things kind of vary accordingly. We're at below, slightly above the norm. But reputation and subject matter expertise is like, why people go to MLA. So that's great. Providing opportunities for online engagement is third at 83%. And providing relevant content is also important at 80%. None of this is surprising, but it's good to actually see the data behind it. and it's good to know.

Now the lower rankings. Value for money at 47%. That is likely a reflection of tight library budgets, and all personal budgets, and the fact that we often are competing against free offering, supported by government funding outside of MLA. And yes, for a lot of the quality content there is a charge, and that is likely a response reaction to that. So at least we don't have a specific reason why, but certainly what we're inferring. Fostering innovation at 54%. With the interesting observations that non-members scored MLA at 61% for innovation higher than members. So non-members think MLA is a lot more innovative than our members. I'm not sure what to do with that number, but this is interesting: support and transition during COVID, you can see in blue. We did well, compared to other associations. We actually outperformed in providing online education. We were ready for it because we had started investing and having all the infrastructure for online education starting in 2019 and even earlier. We were ready for that. So, we did well.

Community needs, I'm going to just read the highlights. This slide displays what members and customers are looking for from MLA. Not surprisingly, MLA ranks high and above the benchmark in a couple of areas. The first one is increasing my knowledge and professional skills at 89%, you know, people want that. MLA is achieving that. That number goes up to 96% for non-members. When non-members come to MLA, 96% of them come to MLA for increasing their knowledge and their professional skills. We have a role also for non-members in terms of being the provider for that knowledge and skill and that's a staggering high number. 96% is phenomenal. Being part of a community of like-minded professionals at 87% is quite typical from associations that have strong online communities and other communities. That number goes to 90% when looking at members only and goes down to 50% for non-members. That makes sense because non-members don't have access to our online communities. But for members, 90% look at MLA communities and being surrounded or connected with like-minded individuals as an important item.

This next slide looks at in-person versus virtual engagement. And you know, not surprisingly, the combination of the fact of being hybrid, or the ability to have both in-person and virtual, which is what MLA has done, ranks the highest. So what MLA is doing and what people are looking for is in line with what people are hoping for or expecting. So, that's great. So that's an interesting report. The Board looked at a lot more detail. We will also find other ways of communicating the deal of that report to everyone. But I wanted to give you some highlights. And last in my presentation—big drum roll—is MLA technology. We've been hinting about it, talking about it for years. This is actually happening this year. It's a very exciting project. Hard to summarize on the slide. But I'll give it a shot. We're changing 100% of our technology. And we're looking for to starting to roll that out in September. There will be some pre-launches and tests with some subgroups of MLA to give feedback and try things out on various things, so that we integrate that when we actually launch. And there will be multiple re-launches as we stage this and integrate a lot of feedback, so the idea is to get it out and then also then improve it in stages over the following year. For the user experience, we want to have best in class and things like membership, e-commerce communities, learning, recognition, events, those kind of key things. Content integrated from everywhere, easy to find, I'm hearing claps from many of you, and the site point content, content from everywhere. We also want MLA to be easy to do business with. That means a whole bunch of different things. But it also means flexibility in membership, in subscription models, bundles, volume discounts, ability to pay by installments. All those different types of things that we have had a hard time doing, because, frankly, of technology. We also want to be engaged through data driven future development decisions. We don't take the data for people like, and don't like into account enough to drive decisions for future content. We should and will. We also want to give you action dashboards, reminders, incentive, suggestions, all those types of things, so that things are easier for you to get to. And there's also this big concept of journeys. You know whether you're a new member or new to the profession, whether you have those preferences, and we want to be able to have a more relevant experience depending on what you're looking for, so that you're not drowned in too much content. Though you would have access to the whole contact. You may have some suggestions, those types of things or some identified areas that are more specific to your interest, that you've decided on that. You're interested in this or that. For the association, it's really important to have a good return on investment. That we're not spending too much. We're managing our cost so that we're much more productive in how the staff is able to work on the back end of these different things. All of that we're working on. You'll see on the column on the right that we are relying on the platform that is based on 80% open-source technology. That is all integrated together through a middleware and a back end. We're doing this in partnership with our management company, MCI. The reason the math works is because MCI is doing this as a multi-tenant platform for dozens, and eventually hundreds of clients. MLA is going to have a sweet deal because we've been here from the grounds up. We're going to have the really the best of the best at a wonderful price, because of our early engagement. So, look forward to that! That does it Shannon for my report.

Shannon D. Jones: Thank you, Kevin. You got a lot of fanfare in the chat box because all of us are excited and looking forward to the new technology. Thank you for your whole report. We are especially excited about the new technology.

### Annual Report

Shannon D. Jones: The next order of business is to present the 2023 Annual Report. The MLA annual reports are valuable for all of us to read. You may discover parts of MLA you did not know about, or incredible things your colleagues have enabled. Take time to read them, even if it's just the executive summaries. Those reports show the immense diversity of our communities and our programs. They're also part of MLA 's archives. So, if your name is in there, you are officially recorded for prosperity. This slide shows all of the MLA components that contribute to this comprehensive document.

Reports are available on MLANET, along with reports from past years. And I see in the chat box you all were getting those links correct. And we're already ahead of us. Thank you all for your engagement. And we also had a request that Kevin read all of the annual reports out loud on this call today, so I don't know how much time you all got, but you know, Kevin has a good reading voice. Kevin, I don't know if you want to honor that request or not. I'll do it after the Chris reads the Bylaws, and that would be just fine. Excellent!

### Recognition of Retiring Directors

Shannon D. Jones: Those who are nominated each year as potential members of the MLA Board of Directors are selected, by virtue of their experience and reputation, to serve the association. But few can imagine beforehand the level of commitment the election to the Board requires. And it seemed like even more so this year. Directors have completed their term on the MLA Board and have served our association with enthusiasm, dedication, grace, good humor, and perseverance. The Association and the Board of Directors express our appreciation and recognize each of you today for the extraordinary work and thought for leadership you've provided during your term of service. Thank you for a job well done to:

- Heather N. Holmes, AHIP
- Adela V. Justice, AHIP
- Brenda M. Linares, AHIP
- J. Dale Prince, AHIP.

Give them a round of applause. Though they are retiring from the Board of Directors, they are not retiring from their service to MLA. You will see them again, and in another capacity. We'll let them take about a week off, and then we'll put them back in the game.

### Recognition of Immediate Past President

Shannon D. Jones: Now it is time to recognize our Immediate Past-President, and so it is my pleasure to and just an honor to express sincere gratitude to Kris Alpi.

To Kris Alpi, AHIP, FMLA. MLA's '21-22 President.

Kris, I am pleased to thank you on behalf of the MLA membership for your leadership during your Presidential year. We were impressed by all of the activities you undertook during your year as President, and this year as Immediate Past-President. To highlight a few, you have been a strong advocate for the voice of the members striving to ensure that everyone's voice is heard. Your diligence and commitment has led to the establishment of the hospital libraries, caucus advocacy, initiative to the to develop resources for hospital and health care administrators highlighting the mission-critical roles that health sciences information professionals provide in health care settings. One key outcome is the statement calling on hospital and health system, executives to fund libraries and library staff. Another initiative seeks to ensure that librarians and information professionals earn authorship on evidence census publications, such as guidelines and systematic reviews for the intellectual contribution to published works. And you all might remember that Kris and that team received the Presidential Award for their efforts with that statement.

Kris, you've also distinguished yourself in the area of research, generously sharing your knowledge and skills, most recently supporting the assessment of MLA's community's governance model in collaboration with Adela Justice, Helen Ann Brown Epstein and Melissa Rethlefsen. Your work with the MLA Insight Initiative helped lay the groundwork for the development of MLA's 2022 and 2023 Collection Development Symposiums.

This past year you worked with members to name our three scholarships in recognition of three distinguished MLA members for their lifetime achievements and for the efforts to further diversify to further diversity, equity, and inclusion. We are excited to name those scholarships after Gwendolyn S. Cruzat, Beverly Murphy MLA Scholarship for Underrepresented Students, and Ana D. Cleveland MLA Scholarship. Thank you for your leadership and accomplishments during your presidency and your year as Past-President, and for all you have done for MLA and the profession, and for all you will continue to do for the greater good of health sciences. And I look forward to seeing you in other capacities with MLA. Kris, I yield the floor to you in case you want to share any words.

Kris Alpi: Just a few. First of all, thank you, Shannon, for your kindness and everything you've had to say today to our members. It's been an honor and a privilege to be entrusted by our MLA members to represent and serve them. And I have to do a special shout out, like you did, to Beverly and Gwen Cruzat at seeing them together at the fellows meeting in Detroit, celebrating the scholarships with just a real highlight of my time in MLA. As well, that everybody working together on that and grants and scholarships, and awards and history and diversity, equity and inclusion made possible. I'm confident that you're going to rock this Past-Presidential year as well. So, I just want to thank everyone for electing me for this tremendous opportunity. And apparently, you're going to actually have a little check in with me and Adela at me at Amy Blevins' inaugural. So, you're not done with this yet. Thanks, Shannon.

Shannon D. Jones: You are absolutely welcome. Yes, Amy's inaugural address is going to be the best show in town in June. So, you know, be there or be square.

### Announcement of Election Results

Shannon D. Jones: Next, we are going to announce the election results. You all probably have seen them, but we're going to announce them to you anyway. The MLA ‘23 election was conducted from March 8th to March 29, 2023. Voting statistics can be seen on the screen. Election results were announced on April 13th, 2023, in *MLAConnect*. The following are the election results. Nine individuals were elected for a one-year term to the Nominating Committee. Their names appear on your screen [visit the *MLAConnect* link to view the names]. Please join us in congratulating them and offering them kudos in the chat box.

Brenda Linares, AHIP was elected to service President-Elect. We welcome Brenda back to the Board. Brenda's served on the Board from 2020 to 2023, and she retired off the Board for about 10 minutes, and now she's coming back. I look forward to getting to work with Brenda next year as President-Elect.

Congratulations are also in order to Andy Hickner, Irene (Rena) Lubker, AHIP, Tamara M. Nelson, AHIP, who were each elected by the membership for a three-year term to the MLA Board of Directors. Keith Pickett was elected by the Chapter Council to serve as the 2023 to 2025 Chair, filling the vacancy that occurred when P. J. Greer decided to retire. We also offer congratulations to Dede Rios who was elected by the Community's Council to serve as its chair for 2023 to 2026. Both Keith and DeDe will serve three-year terms on the MLA Board of Directors in their roles as Community Council Chairs. Welcome Andy, Rena, Tamara, Keith, and Dede. And I got to spend time with them last week in Detroit. But on your screen, you see Dede, Rena, Andy, and Tamara right after our Board meeting in Detroit.

Now it is time for my year as President to come to a close.

### Introduction of Incoming President

Shannon D. Jones: It is my honor and pleasure to introduce the 2023-2024 President, Amy Blevins, Associate Director for Public Services at the Ruth Lilly Medical Library at the Indiana University School of Medicine. She's also the Evidence Based Medicine Thread Leader for IUSM. Where she leads a team of library faculty and staff in support of the education, research, clinical translation and a professional education and population helped initiatives of the of the Indiana University School of Medicine. Amy's career has focused on teaching evidence-based medicine and critical appraisal as well as partnering on systematic reviews and meta-analysis. She is extensively involved in several continuing education initiatives as an instructor facilitator. For the last few years, she has been using has been using her skills and information retrieval and critical appraisal to support the WISE COVID-19 Expert Review of Relevant and Emerging Literature, COVID-19 Expert Responses to Key Questions, and serves as a member of the WISE Indiana Internal Advisory Team. Amy's strong service record includes service on the Board of Directors, MLA Treasurer, Chair of the Professional Recruitment and Retention Committee Mentor and membership on several committees and task forces. She has also been an active leader in the Midwest Chapter, and many of MLA's caucuses. She has received numerous honors and awards, including the Lucretia W. McClure Excellent and Education Award, the Educational Media and Technology Section Annual Meeting, Grant, Section Project of the Year with EMTS, the Medical Informatics Section of MLA Annual Meeting Grant. Oh, I'm sorry, The Medical the Medical Informatics Section of Medical Library Association Annual Meeting Grant. That is a tongue twister and the ANCHASL Scholar of the Mid-Atlantic Chapter. Amy also has authored or co-authored more than 90 publications throughout her career. Please watch for the upcoming issue of *JMLA* to learn more about Amy.

Now I am very thrilled to pass the gavel to my distinguished colleague Amy Blevins. Amy, I am officially putting this gavel down, and yielding the floor to you. With that Dr. Jones is out.

[Shannon puts down her gavel; Amy takes up the gavel]

### Recognition of Outgoing President

Amy Blevins: Thank you, Shannon, for passing the gavel. I hope you know that there's plenty of work still for you to do with all of us, so don't worry about getting bored, as you're finishing your term as President.

On behalf of the membership and headquarters staff, thank you for your strong leadership and countless hours of service this last year. I feel so fortunate to follow in your footsteps after you mentored me at my very first health sciences library conference, back in Atlanta for the Mid-Atlantic Chapter of MLA. You've been a calm and studied presence, as you encouraged our MLA community to strive for work-life balance during the post-COVID pandemic. Your Be Well MLA series has gifted us with refreshment, emotional strength, and resilience. And I know I've personally benefited greatly from participating in these sessions and share my gratitude for you spearheading the program. And don't worry everyone we're going to continue on with Be Well in the next year, so we'll have some programming to talk about later. Long before and throughout your Presidency you've championed MLA's vision to foster excellence and commitment to diversity, equity, and inclusion in professional practice, leadership of health sciences libraries, and information professionals. We can all learn a lesson from your book by reminding ourselves to be more humble, compassionate, empathetic, and to have genuine care and concern for all of humanity. M.J. Tooey has described you as someone who 'takes chances on people valuing those others might not see as valuable.' This has been visibly transparent when you appointed everyone who applied for a 2022-2023 committee. And finally, I don't want to overlook some of the unique challenges that you faced during your Presidential year: dealing with all of our personalities on the Board of Directors, listening to people you know, joking around during our serious conversations. But seriously, I know you completed your doctoral degree. So now you're Dr. Shannon Jones. You've rebuilt your library. You keep serving as the director of your library, and I know you do a lot for your community through your involvement with the Girl Scouts. So, congratulations on a job well done. Don't relax yet. There's more work to do. And thank you so much, Shannon. And do you have your cup? Oh, there we go. I hope you display your cup proudly, because it symbolizes a year when we and MLA broaden our opportunities to build our future under your leadership.

Shannon D. Jones: Thank you, Amy. I do have this silver cup that says Shannon D. Jones, AHIP, FMLA. President Medical Library Association, 2022-2023. Also developed a habit during the year is that as I was traveling around, Starbucks has 'Been There' mug series so I have a whole collection of mugs to represent the places that I have visited. Unfortunately, none of the Starbucks in Detroit had one, so I'm still going to be hunting for that one.

Thank you all. Thank you to the MLA community who made this year possible. And just thank you to the whole array of people who made my Presidential year enjoyable, and who made it possible. So that I could actually do this. I am so eternally grateful to you all, and I look forward to seeing you all more on the virtual streets of MLA, or somewhere in person. If I didn't get to your chapter meeting, I hope you will invite me at some point to come visit you. But thank you so much, Amy, and I am going to fade into the background.

### Presentation of the 2023-2024 Board of Directors

Amy Blevins: Thanks again, Shannon. MLA members I'm very pleased to present your 2023 - 2024 Board of Directors.

Congratulations to all the directors on being elected. If our names are not familiar to you, especially those of you who may have joined MLA after the election, I hope that you will look us up and connect with us via MLANET. Here are our photos to help put faces with our names, as you see us, either at future virtual forums, committee, caucus, chapter, or domain hub meetings, or in-person next year at MLA, or maybe in chapter meetings. And this is a photo of your Board taken last week in Detroit. I look forward to working together and thank you to the Board members for your commitment to MLA and dedication to the profession.

Let's congratulate our new Board Secretary, Tamara Nelson. Hi, Tamara. You're going to be busy on your first assignment with our next order of business. I hope you are ready for that.

### Resolutions

Amy Blevins: I now have the honor to finish the remaining items of business before we adjourn. Next, we have resolutions.

We have no resolutions at this time so we're moving on to new business.

### New Business

Amy Blevins: Our next order of business is to consider amendments to the MLA Bylaws. Please welcome our Bylaws Committee Co-Chairs, Amy Lyons, and Dave Duggar. Amy Lyons will introduce the Bylaws Committee members and give us background information on these proposed changes, and then David will read the motions.

### Summary of MLA Annual Business Meeting Discussion re: Proposed Amendments to the MLA Bylaws

From time to time, the Medical Library Association (MLA) will revise its Bylaws to adjust with the times, align procedures with current or desired new practices and policies of the organization, and more generally to ensure that MLA's foundational governance document is as effective as possible for the fulfillment of the MLA mission.

In 2022, the MLA Board of Directors charged the Bylaws Committee to review and propose updates to the MLA Bylaws. As part of its process, the Bylaws Committee reached out to MLA components, exchanged with the Board of Directors, and organized two Open Forums with members on December 7, 2022, and January 17, 2023. The Bylaws Committee integrated the feedback from its extensive dialog into the proposed modification of the MLA Bylaws it submitted to the Board of Directors.

The proposed modification of the MLA Bylaws was structured into two motions to allow MLA members to approve or reject those motions as separate items.

The Board of Directors approved the two motions for the proposed amendments to the Bylaws on January 25, 2023. As a result of the Board approval, proposed amendments could be shared with the membership for discussion.

On February 2, 2023, the two motions were presented to MLA members in MLA Connect (https://www.mlanet.org/blog/revisions-to-the-mla-bylaws-proposed), along with the process for amending the MLA Bylaws and rationale for the amendments.

On May 25, 2023, MLA members gathered for the MLA Business Meeting which included the agenda item for discussion and vote on the wording and direction of both motions. Members engaged with each other, members of the Board of Directors and members of the Bylaws Committee for 3+ hours in a constructive dialog that is summarized below.

### Motion 1

The proposed Motion 1 groups a number of changes outlined as follows:

- Codification in the MLA Bylaws of diversity, equity, and inclusion (DEI) as MLA core values along with other changes to use modern language that promotes inclusivity;
- More flexibility and inclusiveness for MLA Chapters by only requiring MLA membership for select positions, rather than all officers and committee chairs;
- Allowance for electronic meetings in accordance with Robert's Rules of Order, 12th edition, September 2020;
- More flexibility in individual membership structure, allowing the MLA Board of Directors to modify the option to modify the membership year from the current and rigid “calendar” year schedule (January 1 to December 31) to the more flexible and association best-practice “annual” year schedule (join anytime for a year);
- More flexibility in the frequency of changes to individual dues, allowing the MLA Board of Directors the option to increase annual dues more frequently and by lower amounts instead of the higher dues increase every three years as currently specified; and
- Multiple changes to the MLA Bylaws language to match current practice and remove outdated information, including updates related to MLA Caucuses replacing MLA Sections (in the current Bylaws) and SIGs (not in the current Bylaws) and the change of name of the council from Section Council to Community Council.

*Summary of the MLA Member Discussion of Motion 1*

- Members had no questions nor motions regarding incorporating DEI, allowing the Board of Directors to modify the membership year from “calendar” to “annual,” and the cleaning up and updating of language.
- Members supported language that would allow Chapters to appoint non-MLA members to Chapter committees, ad hoc committees, task forces and panels. Chapter representatives on MLA committees would have to comply with the committee's stated requirements, which may include MLA membership.
- Multiple amendments were proposed and voted down regarding the article on dues changes, Article III, Section 6.: “The Board of Directors shall fix the amount of membership dues for all membership categories each year. Notice of a proposed change in dues shall be sent to each member at least nine weeks before the start of the Associations' fiscal year.”

The members proposing amendments to the MLA Bylaws amendment expressed their concern with the ability of MLA Board of Directors to modify membership dues annually (versus every three years as specified in the current Bylaws).

MLA officials pointed out that the MLA Board of Directors already had the power to modify dues, that the Board has exercised restraint in raising dues even with higher inflation with the average annual dues increase of the last five years at 1.8% per year, that in some membership categories dues were actually reduced, that requirements for lower dues were relaxed, and that the objective is to avoid the sticker shock of larger more infrequent increases.

Some members indicated that the Board of Directors had MLA's best interest in mind, were well aware of the funding challenges of individuals and employers, that constraining the Board of Directors' ability to manage MLA finances could have adverse consequences such as insolvency, and that MLA members elect members of the Board of Directors to make decisions including at times unpopular ones.

Motion 1 was unchanged.

*Post Discussion Point of Information*

On July 25, 2023, the Board of Directors approved a motion that maintains 2024 individual dues at the 2023 level.

### Motion 2

The proposed Motion 2 addressed changes to the Chapter and Community Councils and their representation on the Board of Directors.

*Summary of the MLA Member Discussion of Motion 2*

Members approved withdrawing Motion 2. The main rationale was that the problem Motion 2 was looking to solve (challenges in attracting volunteer leadership and their ability to commit time) was an outlier situation that had been resolved by other means (recruitment) and that modifying the Bylaws was therefore unnecessary.

Members approved withdrawing Motion 2 resulting in no proposed changes to the Bylaws.

### Next Steps

In compliance with the current MLA Bylaws, MLA members are asked to vote on Motion 1 between July 27, 2023, and August 18, 2023, by electronic ballot, dates set by a motion of the Board of Directors approved on July 25, 2023.

The MLA Bylaws make the following provisions for amendment of the Bylaws:

## ARTICLE XV. AMENDMENT OF THE BYLAWS

### Section 1. Notification

- The Bylaws may be amended or rescinded by two-thirds of those voting by ballot on any properly proposed and considered amendment as specified in this Article.
- Notice of proposed amendments recommended by the Board of Directors (or petitioned by a minimum of one hundred fifty (150) voting members at least sixteen weeks before the start of the next Annual Meeting) shall be sent to each Voting Member at least nine weeks before the date of the meeting. The notice shall indicate the time and place of the next Annual Meeting where the proposed amendments will be considered.

### Section 2. Consideration at Annual Meeting

Opportunity shall be given at the Annual Meeting for debating and amending any properly proposed amendments to any part of the Bylaws.

### Section 3. Ballot

A ballot containing all proposed amendments, along with a transcription or summary of the Annual Meeting discussion on the amendments shall be distributed to each Voting Member. The time of the beginning and closing of the ballot and of the reporting of results shall be fixed by the Board of Directors. To amend or rescind any portion of the Bylaws, twenty-five (25) percent of the total ballots distributed must be returned properly filled in and on time, and two-thirds of these ballots must be affirmative.

### Section 4. Effective Date

The Bylaws and any future amendments thereto shall become effective on January 1 of the year following their acceptance by ballot. [January 1, 2024].

### Conclusion

Amy Blevins: This brings the discussion of our Bylaws to a close. The amendments and summary of your discussion on the Bylaws will be made available to all voting members for their consideration and vote this summer. I want to thank everyone who spent many long hours working on these issues. I especially would like to thank the Bylaws Committee, the Board of Directors, members of the Chapter and Section Councils, the Membership Committee, everyone who attended the two open forums, everybody who attended the Business Meeting today, and MLA headquarters staff for all of your hard work on behalf of the association.

### Adjourn

Amy Blevins: This was quite a start to my year as MLA President. Is this the longest business meeting we've had online? I think it is. But you know, somebody else can tell me if that's true. Please join me on Wednesday, June 20. First for my inaugural address. It will be exactly the amount of time that it says on the screen here. I don't expect it to go long. We're doing something new and hopefully exciting this year. And I'm thrilled to say that you'll be hearing from not just me, but from the people who are working on several initiatives in the new year and finishing up on several of the initiatives that we had before.

Christopher Shaffer: Maria, if you could put up a quick poll? If Amy, as Chair of the Board of Directors, our President of the Board of Directors, puts forward a motion to withdraw the motion, because you are the original mover we can all very quickly vote on it, get the same result we just got, and it will be actually formally withdrawn.

Amy Blevins: And otherwise, as Kevin notes, we'd have to send a blank motion to the membership for a vote, which is silly. Oh, no, I can't imagine how confusing that would be.

Christopher Shaffer: Tell me again, what do you want me to do? You just say I move to withdraw the motion, on behalf of the Board. I move to withdraw Motion 2 on behalf of the Board. Okay, any discussion?

Amy Blevins: No, I don't see any.

Christopher Shaffer: Right, and it doesn't need a second, because it's coming from the Board. Maria, if you could very quickly put up a poll. Yes, is to withdraw the motion. No is to retain the motion and abstain is also an option. And that way we will have crossed all the T's and dotted all the I's for the Bylaws. And the reason we had to do it this way is because the person who submitted the original motion to blank it all out, couldn't actually do the withdrawal because they weren't the original mover.

Amy Blevins: I'm just happy that we're moving forward. I'm going to motion to extend my inaugural address to five hours. I think that's the amount of time that I'm going to go. I'm just kidding everyone. That's a joke. Beverly, you'd stay for five hours of me talking, right? I'm also going to create a mandatory Bylaws listserv. I think that went over well last time. So, I think it's time to bring it back. Also kidding, I'm not creating any mandatory listservs for anybody. I'm just filling space while we all fill out that poll. Hopefully, everybody is voting appropriately, according to their heart.

Shannon D. Jones: Amy, while you are filling time, kudos to you and Chris for leading a robust discussion. I am so grateful that you led the discussion. So, kudos!

Amy Blevins: Well, I learned it from watching you, Shannon.

Amy Lyons: As I said, I think that you know the fact that we were able to have this discussion, I think, really speaks well to the organization in the membership. You and Chris did a really nice job of having it be very civil towards each other. And I think that was important because it could have gone haywire.

Shannon D. Jones: Yeah, especially with those of us who are hungry because we could turn into “hangry” very easily.

Amy Blevins: Everybody, the motion to withdraw Motion 2 passed with 96% with 3% abstaining and 1% no. Tamara Nelson, can I welcome you back for our final business?

Tamara Nelson: Yes, you can, Amy. Okay, everyone. You know it was an amazing robust discussion and meeting. And I get to conclude. That's all, y'all. At this time, I move to adjourn the 2023 MLA annual business meeting.

Amy Blevins: Well, and it has been moved to adjourn. I propose to approve the motion by unanimous consent. Any member may object, and we will all know who you are, in which case call for discussion, and then vote. Please read your hand. If you object, you have 20 seconds.

Kris Alpi: Let's just wave goodbye to everyone.

Amy Blevins: The motion is passed, and the meeting is adjourned. Thank you for attending our MLA Business Meeting.

## MLA 2023/2024 INCOMING PRESIDENTIAL ADDRESS (VIRTUAL)

[View the recorded virtual session of Amy Blevin's Inaugural address via MLANET.]

## OTHER PLENARY SESSIONS

[The following plenaries were also livestreamed for virtual attendees.]

### Wednesday, May 17, 2023, Joseph Leiter NLM/MLA Lecture

Keynote Speaker: Craig Robertson, PhD

The Joseph Leiter NLM/MLA Lectureship was established in 1983 to stimulate intellectual liaison between MLA and the National Library of Medicine (NLM). Lectures are chosen for their ability to discuss subjects related to biomedical communications. The lecture is presented every other year at NLM and in alternating years at the association's annual conference.

Craig Robertson spoke on his book, *The Filing Cabinet: A Vertical History of Information*. This session was livestreamed for virtual attendees and is available post conference on demand.

### Thursday, May 18, 2023, John P. McGovern Award Lecture.

Keynote Speaker: Terri Givens

The 2023 McGovern Lecture was a structured conversation with our speaker and the 2023 National Program Committee Cochairs Kate Flewelling and Ryan Harris.

Terri Givens spoke on her book *Radical Empathy: Finding a Path to Bridging Racial Divides*. This session was livestreamed for virtual attendees and is available post conference on demand.

### Thursday, May 18, 2023, Janet Doe Lecture

Keynote Speaker: Michelle Kraft

[For the Janet Doe lecture, please see Michelle Kraft's piece in the January issue of *JMLA*]

### Symposium on Collection Development & Resource Sharing: Shifting Into Second Gear

Symposia sessions were held in person throughout the conference.

*Wednesday, May 17, 2023*

- The Great Debate on Controlled Digital Lending: And the Checkered Flag Comes Down On…
- To Merge Into the Resource Cost Sharing Lane or Not: Evaluating and Developing an Approach to Resource Sharing

*Thursday, May 18, 2023*

- On the Road to Transformative Agreements: National and International Perspectives (Livestreamed)
- Embracing Health Habits to Create Sustainable Library-Vendor Relationships: A Panel Discussion for Librarians, Publishers & Vendors

*Friday, May 19, 2023*

- Cruising in the “Non-Traditional” Collections Lane with Your MAAP: Challenges and Solutions
- From Manual to Automatic: Improving the Accessibility of Library Electronic Resources

### The Symposia On Leadership & Management

Symposia sessions were held in person throughout the conference.

*Wednesday, May 17, 2023*

- Work & Lead More Effectively: Understand and Adapt Your Preferred Style
- Connecting With Stakeholders and Communicating Your Library's Value Proposition (Livestreamed)

*Thursday, May 18, 2023*

- It's Not Them, It's Us: Understanding and Addressing the Factors that Negatively Impact the Recruitment, Hiring, and Retention of BIPOC Librarians
- Re-Thinking the Definition of Community and Connections in a Virtually Connected World (livestreamed)

*Friday, May 19, 2023*

- Improv and Librarianship
- Forging Ahead: Libraries as Engines of Innovation

## PROGRAM SESSIONS

During the face-to-face portion of the annual meeting, there were 14 immersion sessions, 47 SLA education sessions 100 papers, 10 SLA-contributed papers, and 70 lightning talks. The live immersion sessions included interactive breakout sessions, Q&A, and virtual chat with presenters. There were 16 virtual papers, and 3 SLA virtual papers.

Paper abstracts that were scheduled to be presented are available on the MLA ‘23 website. The final version of the abstracts reflecting only those presented at the meeting is included as an online-only supplemental file to the April 2024 issue of the *Journal of the Medical Library Association* (see Appendices).

## POSTER SESSIONS

The Poster Gallery featured 103 posters in an on-demand viewing format. There were 84 posters that were presented live in Detroit, MI over two sessions: Thursday, May 18, 2023, 12:00-1:30 p.m. and Friday, May 19, 2023, 12:00-1:30 p.m.

Poster abstracts that were scheduled to be presented are available on the MLA ‘23 meeting website. The final version of the abstracts reflecting only those posters presented at the meeting is included as an online-only supplemental file to the April 2024 issue of the *Journal of the Medical Library Association* (see Appendices).

### MLA Research Training Institute Poster Ignite Session

The MLA Research Training Institute had a dedicated in person poster session.

Thursday, May 18, 2023

- Trying Libraries on for Size: Mapping the Anti-fatness Literature in Library Science, Emily Gilbert
- Impact of COVID-19 on Solo Health Librarian Mentorships, Bridget Jivanelli
- A Graphic Medicine Reading Experience for Undergraduate Nursing Students, Michelle Ott
- Knowing how to See Behind the Data Points: Inclusive Data Ethics Competencies for Health Sciences Librarians, Nancy Shin, AHIP
- Pivoting When Burnout Burns Out Your Research Project, Ashley Michelle Thomas
- Consensus for Impact Variable Data Point Terms and Definitions: A Delphi Study, Gwen Wilson, AHIP
- Health Literacy Workshops: Librarian Support in Employee Wellness Programs, Colleen Foy
- Exploring Clinician Expectations and Preferences of Library Study Guides, Ann Biszaha
- Student Wellness Initiatives in Jesuit University Libraries, Claire Sharifi
- Working Toward a Lasting Impression: A Survey Protocol to Measure Recall of Undergraduate Nursing Library Instruction, Jason Wardell
- Moving DEI Forward: Does Cultural Competence Has a Place in Teaching and Learning?, Alessia Aznin-Yost

## OTHER MEETINGS AND EVENTS

Due to the Covid-19 pandemic, the following meetings were held virtually prior to, during, and after MLA ‘23: Academic Librarians Caucus Business Meeting, April 21, 2023; African American Medical Library Alliance Caucus Business Meeting, May 10, 2023; Animal and Veterinary Information Specialist Caucus End of Year/Business and Networking Meeting, April 26, 2023; Experience MLA, February 16, 2023; Business Meeting, August 18, 2022; Business Meeting, October 20, 2022; Basic Science Caucus Business Meeting, April 19, 2023; Cancer Librarians Caucus Business Meeting; Clinical Librarians and Evidence-Based Healthcare Caucus Business Meeting; Collection Development Caucus Business Meeting, April 27, 2023; Community Council Business Meeting; Consumer and Patient Health Information Services Caucus Meeting, March 24, 2023; Data Caucus Business Meeting, May 1, 2023; Dental Caucus Spring Meeting, April 27, 2023; Federal Libraries Caucus Business Meeting, March 29, 2023; Health Association and Corporate Librarians Caucus Business Meeting, April 11, 2023; History of the Health Sciences Caucus Business Meeting, May 3, 2023; Hospital Library Caucus Business Meeting, May 30, 2023; International Cooperation Caucus Business Meeting, April 26, 2023; Latinx Caucus Business Meeting, May 5, 2023; Leadership and Management Caucus Business Meeting, May 24, 2023; LGBTQIA+ Health Sciences Librarians Caucus Business Meeting, December 8, 2022; Libraries in Health Sciences Curriculum Caucus Business Meeting, April 27, 2023; Medical Informatics Caucus Business Meeting, May 31, 2023; NNLM Regional Meetings; New Members Caucus Business Meeting, April 26, 2023; Nursing and Allied Health Resources Services Caucus Business Meeting, May 24, 2023; Osteopathic Libraries Caucus Business Meeting, March 15, 2023; Pediatric Librarians Caucus Business Meeting, May 4, 2023; Pharmacy and Drug Information Caucus Business Meeting, June 13, 2023; Public Health/Health Administration Caucus Business Meeting, May 15, 2023; Public Services Caucus Business Meeting, May 9, 2023; Research Caucus Business Meeting, March 27, 2023; Scholarly Communications Caucus Business Meeting, April 28, 2023; Systematic Reviews Caucus Business Meeting, April 19, 2023; Technical Services Caucus Business Meeting, May 8, 2023; Technology in Education Caucus Business Meeting, January 25, 2023; User Experience Caucus Business Meeting, June 1, 2023; Vision Science Caucus Business Meeting, April 4, 2023.

## PUBMED UPDATE

The PubMed Update and Q&A took place on Wednesday, May 17, 2023, from 3:30-4:30 p.m. and was also livestreamed for virtual attendees.

[Summary of text by AI: for complete item, please watch livestream]

Amanda Sawyer: Good afternoon, everyone! I'm Amanda Sawyer from the PubMed team at NCBI. Excited to share the PubMed update at MLA SLA. Today, I'll cover PubMed's growth, new features, and updates. We now have over 35 million citations, with 27 million linked to full text. In the last year, 1.6 million citations were added. We get 3.5 million visitors per weekday from around the world, conducting 5.5 million searches daily.

Now, let's look at recent updates. We introduced the PubMed Utilities API, aligning it with the website for better sync. This benefits users accessing data programmatically and products using PubMed data. We also enhanced the User Guide for large result sets.

Updates to the Journals Translation Table improve journal searching flexibility. Journal names without initial articles are now searchable, enhancing accuracy.

The Favorites button on PubMed's abstract page became the Collections button. It offers the same functionality, providing a unified user experience between PubMed and PubMed Central.

We refined the Additional Filters interface, renaming the Journal category and adding an Exclude Pre-prints filter based on NIH pre-print pilot feedback.

A significant update addresses lengthy author lists in search results. Author lists exceeding 1,200 characters are truncated with an ellipsis, improving readability.

We enhanced the warning for phrase searches not found in PubMed's phrase index, providing clarity, and linking to the User Guide. Users can suggest phrases for inclusion, meeting specific criteria.

A major update is the introduction of Proximity Searching in November 2022. Users can search for terms in any order within a specified distance in the title or title abstract fields. This powerful tool opens new search possibilities.

To stay updated, follow resources like the NLM Technical Bulletin, NCBI Insights Blog, and PubMed New and Noteworthy RSS feed. The PubMed User Guide remains a valuable resource, regularly updated to address user queries. Explore PubMed tutorials and the PubMed Trainers Toolkit for additional support.

For questions, bug reports, or suggestions, reach out to the PubMed Help Desk. Feedback informs PubMed's development, helping us enhance the user experience. Thank you for your attention and for helping us improve PubMed.

## NATIONAL LIBRARY OF MEDICINE UPDATE

The National Library of Medicine (NLM) Update and Q&A took place on Friday, May 19, 2023, from 10:30 a.m.-12:00 p.m. and was also livestreamed for virtual attendees.

## OTHER SPECIAL EVENTS AND RECEPTIONS

Wednesday, May 17, 2023, 7:30-9:00 a.m.

- New Members/First Time Attendee Program & Networking

Wednesday, May 17, 2023, 12:00-1:30 p.m.

- MLA Fellows Luncheon

Wednesday, May 17, 2023, 12:30-1:30 p.m.

- Communities Lunch for MLA & SLA

Wednesday, May 17, 2023, 4:30-5:30 p.m.

- MLA Leaders' Recognition and International Visitor Reception (by invitation)

Wednesday, May 17, 2023, 5:30-7:30 p.m.

- Welcome Reception & Opening of the Hall of Exhibits: Happy Anniversary MLA!

Thursday, May 18, 2023, 6:00-9:00 p.m.

- Networking and Gaming Night

Friday, May 19, 2023, 2:30-3:15 p.m.

- Closing Session and Welcome Our 125th Year!

## EXHIBIT HALL

The Exhibit Hall was home to 90 vendors who presented various demonstrations of their products. The Exhibit Hall began with an opening reception on Wednesday, May 17, 2023, 5:30-7:30 p.m. that was sponsored by Wolters Kluwer. The Exhibit Hall was open on Thursday, May 18, 2023, 9:00 a.m. - 5:00 p.m. and Friday, May 19, 2023, 9:00 a.m. - 2:00 p.m.

Exhibitors held both sunrise seminars, lunch and learns, as well as technology showcases to highlight new products.

### Thursday, May 17, 2022

Sunrise Seminars

- Wiley – The Shape of Wiley's Portfolio and How It Fits With Your Library
- Elsevier – Embase is Learning a New Language: Use the New Translation Tool to Transform Queries; and PICO Search Form for Systematic Reviews
- Rittenhouse – Meeting the Evolving Digital Needs Your STM Patrons

Lunch and Learns

- EBSCO
- Springer Nature – Empowering Knowledge Managers with Technology to Power Next Generation Discovery and Search Tools
- Third Iron – Is the Article Open Access Retracted of From a Problematic Journal? How LibKey Uses Article-Level Intelligence to Delivery the Fastest Most Accurate and Informed Linking to a Full Test
- Wolters Kluwer

Technology Showcase

- Clarivate – Introducing Endnote
- BMJ – Analyze and Evaluate Real Word ??, Clinical Guidelines, and Point of Care Data
- McGraw Hill – McGraw Hill: Preview the Access ™ App
- McGraw Hill – McGraw Hill: A Discussion with Boarders & Beyond Founder Dr. Jason Ryan

### Friday, May 19, 2023

Sunrise Seminars

- Wolters Kluwer – Connecting the Health Sciences Library with Institutional EBP, QI, and Research Efforts
- East View – Emerging Research from China: A Comprehensive Approach
- Covidence – Covidence: Better Systematic Review Management

Lunch and Learns

- OCLC – Leveraging Your Collection and Serving Your Community an OCLC Update
- Open Athens – Access Lab Forum by Open Athens
- Elsevier – Better Together – Bridging Non-Patent Literature (NPL) and Patents by Indexing

Technology Showcase

- Clarivate – Preparing Future Mental Health Practitioners with ProQuest One Psychology
- Clarivate – Bridging the Gap: Connecting Varied Sources via the Web of Science

## CONTINUING EDUCATION COURSES

Tuesday, May 16, 2023

CE100 Effectiveness and Efficiency in Exhaustive Searches

CE200 Developing a Collection for All Your Patrons: A Workshop on Writing a Collection Development Policy

CE400 Conducting Difficult Conversations: Improving Workplace Effectiveness, Relationships, and Satisfaction

CE500 Research for the Non-Researcher

## RESOURCES AND SERVICES

The online itinerary planner sponsored by Wolters Kluwer allowed attendees to peruse programs and events online. Live streaming was available on Twitter using the hashtag #mlanet23. The annual meeting blog posts are available on the MLA website. The MLA Professional Recruitment and Retention Committee (PRRC) is pleased to sponsor the MLA ‘23 Virtual Resume Clinic.

## CLOSING SESSION

The Closing Session was held on Friday, May 18, 2023, from 2:30-3:15 p.m. and was livestreamed for virtual attendees. This session made an important note of the Medical Library Association's 125th Anniversary year.

## SUPPLEMENTAL FILES

**Appendix:** MLA ‘23 Program Session Abstracts

**Appendix:** MLA ‘23 Poster Session Abstracts

