# Supplementary material for: 123rd Annual Meeting Medical Library Association, Inc. Detroit, MI May 16-19, 2023
Source: J Med Libr Assoc. 2024 May 22;112(2):E1–E22. doi: 10.5195/jmla.2024.1872 (PMC11305459; doi:10.5195/jmla.2024.1872)
Supplement: Supplementary file 1 — Appendix: MLA ‘23 Program Session Abstracts [file jmla-112-2-E1-s01.pdf]

Medical Library Association
MLA | SLA '23 Hybrid Conference & Exhibits

Immersion Session, Paper, Lightning Talk, Education Session, & Symposia Abstracts

Looking back, forging ahead

MLA | SLA '23

Detroit | May 16-19 | Hybrid | #MLASLA23

Abstracts for contributed content (immersion, paper, lightning talk) sessions are reviewed by members of the Medical Library Association National Program Committee (NPC) and volunteer peer reviewers. Designated NPC members make the final selection of content to be presented at the annual conference. Education Sessions were approved by the Special Library Association. Symposia were selected by joint MLA/SLA special content committees.

Information is as provided by abstract submitters.

IMMERSION SESSIONS

We have sorted content in this section by title in alphabetical order.

The Critical Role of Librarians in Enterprise Data Management

Track: Information Management

Susannah Barnes - Data Intelligence Program Lead, Alation, Cambridge, Wisconsin

Session Overview: This session will cover the critical need for library and information science skills within enterprise data management programs. We will discuss how the effective development of metadata standards, controlled vocabularies, and information architecture allow companies to derive faster, more accurate, and more ethical value from the vast volumes of data to which they have access. Topics covered will include the role of metadata in data classification and privacy management, the development of data cataloging as a new skill set, and the growth of metadata management roles within organizations. Attendees will understand how library and information science knowledge and skills can translate into business and public sector data management roles.

Instructional Methods: We will have a recorded panel discussion and a presentation followed by an interactive question-and-answer session with the audience.

Participant Engagement: Q&A with the primary presenter.

Sponsor(s): SLA Data Community

Defining Your Library Value: Successes & Failures

Track: Professionalism & Leadership

Daniel Bostrom - Member Engagement Manager, RAILS, Burr Ridge, Illinois

John J. DiGilio - Firmwide Director of Library Services, Sidley Austin LLP, LOS ANGELES, California

Gabriele Hysong - Information Operative/Librarian, Library & Knowledge Services Center/ Rolls-Royce Corp, Indianapolis, Indiana

Session Overview: It’s crunch time: your boss wants you to prepare a report and/or presentation on what value your library/information center brings to the organization. What do you do? Do you prepare a bunch of charts and throw some numbers around? Do you quickly gather some testimonials and hope the qualitative thing works?

Defining your value in the library relies so much on knowing what will resonate with administrators. Creating a tailored appeal can make all the difference.

In this deep dive into defining value in the information field, presenters will talk about their successes and failures in making that case. What has worked? What hasn’t? What will they try the next time they get called into this same meeting?

Attendees will hear real-life examples of defining library value. This session will also help create best practices around tactics like report writing, determining ROI, and how to become a better advocate for your services.

Instructional Methods: This session will include a panel discussion of ROI and defining library value.

Participant Engagement: This session will include a Q&A and offer participants the opportunity to discuss their own successes and failures in utilizing ROI.

Sponsor(s): Leadership & Management Development (SLA)

Forging Ahead: Key Insights on Global Health & Equity from Around the World

Track: Health Equity & Global Health

Irene M. Lubker, AHIP - Medical University of South Carolina, Charleston, South Carolina

Ana Corral, MLIS - Health Sciences Librarian, University of Houston, University Libraries, Houston, Texas

Margaret Henderson, AHIP - Health Sciences Librarian, San Diego State University Library, Ramona, California

Gurpreet K. Rana - Global Health Coordinator, Taubman Health Sciences Library, University of Michigan, Ann Arbor, Michigan

Mercy Wamunyima Monde - Medical Librarian, University of Zambia, Lusaka, Lusaka, Zambia

Marcia Barretto

Biliamin Oladele Popoola, n/a - Systems, Scholarly Communications and EBM Librarian, University of Medical Sciences Library, Ondo City, Ondo, Nigeria

Dr. Lilian Niwagaba

Session Overview: In this immersion session, health information professionals from around the world will share their global health & equity projects and discuss their experiences, challenges, and successes. The session will be formatted in a Living Library format, with presenters grouped by global health & equity topics that participants will be able to “check out.” Participants will have the opportunity to check out individual topics and converse with the living books (presenters) and gain an understanding of the connections between health and equity across the globe. By bringing health information professionals together from around the world in a Living Library format, participants and presenters will have the opportunity to discuss health equity and global health issues and engage in open, honest discussions.

Instructional Methods: Various introductory videos by information professionals from around the world will introduce key issues of global health & equity before opening up presenters (live books) for participant checkout. This immersion session will use a Living Library format for presenters (live books) to share stories and lived experiences and foster an environment where participants and presenters can learn from each other. Participants will have the opportunity to ask in-person questions or submit questions via the in-app Q&A feature and on a social media platform. No readings or questions will be required ahead of this immersion session.

Participant Engagement: During this session, presenters will be grouped topically and participants will be

able to “check them out” to hear about a range of global health and equity issues from around the world. Participants will be able to submit questions, via the in-app Q&A feature, engage with other health information professionals on a social media platform, and ask questions in-person during the session. Global health & equity topics can include: introduction of the Cunningham Fellows; presenting at international health conferences; critical issues in global health; access to clean water and environmental factors that impact health; and maternal health. We will use an instructional tool such as padlet to encourage engagement and real time sharing of resources.

Sponsor(s):
MLA International Cooperation Caucus, MLA Social Justice and Health Disparities Caucus

How to Work Effectively with Journal Editors and Peer Reviewers

Track: Professionalism & Leadership

Merle N. Rosenzweig, MALS - Informationist, Taubman Health Sciences Library, University of Michigan, Ann Arbor, Michigan

Michelle Kraft, AHIP, FMLA - Library Director, Cleveland Clinic, Cleveland, Ohio

Willow Fuchs - Business Reference & Instruction Librarian, Tippie College of Business, Iowa City, Iowa

Mark MacEachern, MLIS - Informationist, University of Michigan, Ann Arbor, Michigan

Jonathan D. Eldredge, AHIP, FMLA, PhD - Professor and Evidence-Based Practice Librarian, University of New Mexico, Albuquerque, New Mexico

Session Overview: There will be a panel made up of two journal editors, one from the *Journal of the Medical Library Association* (*JMLA*) and the other from the journal Education Libraries, and two peer reviewers who will provide an understanding of the publishing process and how that can culminate in the publication of an article. The editors will explain the role of an editor and will discuss that role in relation to their journal and how submitted manuscripts are selected. The two peer-reviewers will provide a brief explanation of peer review and focus on their specific journal.

Instructional Methods: The presentation of the panel members will provide the participants with an understanding of the roles of journal editors and peer reviewers. With a Q&A session following the panel presentation participants will be able to ask specific questions they have as to what was presented by the panel members. Ending with a breakout session allows the participants to ask individual panelists questions and get guidance on how they can prepare a manuscript that will be accepted.

Participant Engagement: Following the panel presentations, there will be a brief Q&A session for attendees to ask the panelists questions. The session will end with breakout groups in which attendees will be able to ask individual panelists questions and get guidance on successfully getting published.

Sponsor(s): MLA Research Caucus, MLA Scholarly Communication Caucus, MLA Medical Library Education Caucus, SLA Academic and Education Group

The Limit Does Not Exist: Leveraging Technology to Engage Learners

Track: Education

Hilary M. Jasmin, MSIS - Assistant Professor, Research and Learning Services Librarian, Health Sciences Library, Memphis, Tennessee

Rachel Whitney, AHIP - Research & Education Informationist, MUSC Libraries, Charleston, South Carolina

Kayce D. Gill - Health Sciences Collections Librarian, Annette and Irwin Eskind Family Biomedical Library and Learning Center, Nashville, Tennessee

Jason B. Reed - Associate Professor and Health Sciences Information Specialist, Purdue Libraries and School of Information Studies, West Lafayette, Indiana

Molly K. Maloney - Senior Assistant Librarian, Pharmacy Liaison, University at Buffalo, Health Sciences at Abbott Library, Buffalo, New York

Sally Smith - Health Sciences Librarian, Clemson University, Clemson, South Carolina

Taylor Johnson - Contractor, University of North Carolina - Chapel Hill, Chapel Hill, North Carolina

Session Overview: This immersion session will provide a foundation for this lesson transformation through active demonstration of the technologies being discussed. A panel will discuss a variety of ways they leverage technology to engage different learners in evidence based practice materials. Attendees are invited to BYOI (bring your own instruction) and come prepared with either an existing lesson plan or a potential idea for one. Attendees will be encouraged to share their successes, failures, and questions as presenters moderate a virtual brainstorming session to iterate lesson plans to better leverage various teaching technologies. Attendees will leave the immersion with a component enhanced through instruction technology, which could take the form of live assessments via polling tools, a gamification idea, a video component, and more!

Methods: Panelists will share mini case studies of how they have leveraged technology to engage learners in health information instruction. Examples will include a variety of instruction types including one-shot instruction sessions, small group presentations, large lecture hall classes, and more! The session will be broken up into three sections to demonstrate how attendees can incorporate technology before, during, and after their instruction session.

Participant Engagement: Attendees will interact throughout the presentation through a live-polling software and will engage with active learning via demonstrations of the technology that is being discussed. Participants will be provided time to develop an action plan to integrate technology into an instruction session at their home institutions.

Sponsor(s): MLA Pharmacy and Drug Information Caucus, SLA Pharmaceutical and Health Technology Community

Planning for the NIH Data Management and Sharing Policy: Education and Outreach

Track: Information Management

Justin de la Cruz - Program Specialist, NYU Health Sciences Library, Asheville, North Carolina

Nicole Contaxis, MLIS, MA - Data Librarian and Lead of Data Discovery, NYU Health Sciences Library, New York, New York

Levi Dolan, MLIS - Data Services Librarian, Ruth Lilly Medical Library, Indianapolis, Indiana

Jess Newman McDonald - Research Data & Scholarly Communications Lead, Health Sciences Library, Memphis, Tennessee

Session Overview: The new NIH Data Management and Sharing Policy has created new challenges and opportunities for librarians at institutions receiving NIH funding. This immersion session will center on the new policy in terms of outreach and education. It will include panelists who can speak to how they have updated their approaches to reaching and teaching relevant stakeholders about the new policy requirements. It will also include a discussion on how the policy has challenged current infrastructure and the need for more institutional support to meet the educational demands of this new mandate. The structure of the immersion session will be a moderated panel with an open forum for Q&A and will serve as a venue to discuss questions and concerns about the policy. Note: This is one of two Immersion Sessions we are submitting on this topic.

Instructional Methods: Panelists will each present case studies explaining the education and outreach that has been or will be created at their institutions for the new policy. These case studies will be a jumping off point for a community discussion of aspects of the case studies that other librarians can draw from as they create new modes of supporting the policy. Further, the participants will work in small breakout groups to fill out a checklist that identifies approaches to conducting outreach and education. There will be time during the session for participants to work through this checklist and share their findings with other attendees. The session will end with a Q&A forum to address any lingering questions. Overall, the panel will address some of the biggest challenges institutions face in this new policy landscape.

Participant Engagement: Attendees will be grouped into categories such as Education, Institutional Outreach, DMPTool/DMP Review, Policy Support (General), Preparedness (or lack thereof) in order to discuss these topics in relation to their institution. As mentioned, they will also be working on a checklist that will help them identify partners at their institution as well as the groundwork needed to support the policy given their current educational and outreach activities. Moderators will also be using the MLA app throughout the immersion session for periodic check-ins with the audience as well as an opportunity to get anonymous feedback about questions related to the policy.

Sponsor(s): MLA Data Caucus

Planning for the NIH Data Management and Sharing Policy: Infrastructure

Track: Information Services

Genevieve Milliken, MA, MSLIS - Data Services Librarian, NYU Health Sciences Library / Data Services, Wallkill, New York

Nina Exner, PhD, MLS - Research Data Librarian, Virginia Commonwealth University Libraries, Richmond, Virginia

Katy Smith, PhD, MLIS - Health Sciences Reference Librarian, Saint Louis University Medical Center Library, St Louis, Missouri

Roland B. Welmaker, Sr. - Library Manager - Technical Services, M. Delmar Edwards, MD Library, Atlanta, Georgia

Sara Samuel - Informationist, Taubman Health Sciences Library, University of Michigan, Ann Arbor, Michigan

Jake R. Carlson - Director of Deep Blue Repository and Research Data Services, University of Michigan Library, Ann Arbor, Michigan

Session Overview: The new NIH Data Management and Sharing Policy has created new challenges and opportunities for librarians at institutions receiving NIH funding. This immersion session will center on how to prepare for the new policy in terms of infrastructure and institutional support. It will include panelists who can speak to how they have expanded their service model, worked with institutional partners, identified repositories, and developed resources to address the new policy requirements. It will also include a discussion on how the policy has challenged current infrastructure and the need for more institutional support to meet the demands of this new mandate. The structure of the immersion session will include a moderated panel, a breakout session, and an open forum for Q&A. In total, this immersion session will serve as a venue to discuss questions and concerns about the policy.

Instructional Methods: Panelists will each present case studies explaining the infrastructure that has been (or will be created) at their institutions for the new policy. These case studies will be a jumping off point for a community discussion of aspects of the case studies that other librarians can draw from as they create new modes of supporting the policy. Further, the participants will work in small breakout groups to fill out a checklist that identifies institutional partners, IT personnel, DMPTool support, and other relevant parties related to building infrastructure. There will be time during the session for participants to work through this checklist and share their findings with other attendees. The session will end with a Q&A forum to address any lingering questions. Overall, the panel will address some of the biggest challenges institutions face in this new policy landscape.

Participant Engagement: Attendees will be grouped into categories such as Education, Institutional Outreach, DMPTool/DMP Review, Policy Support (General), Preparedness (or lack thereof) in order to discuss these topics in relation to their institution. As mentioned, they will also be working on a checklist that will help them identify partners at their institution as well as the groundwork needed to support the policy given their current infrastructure and internal policies. Moderators will also be using the MLA feedback app throughout the immersion session for periodic check-ins with the audience as well as an opportunity to get anonymous feedback about questions related to the policy.

Sponsor(s): NNLM National Center for Data Services (NCDS)

Professional Competencies, Information Literacy, and You: Leveraging Curriculum Mapping to Advocate for Library Instruction

Track: Education

Laura Menard - Access Services Department Head, Northern Kentucky University, N/A

Lorraine Porcello - Evidence-Based Practice & Instruction Librarian, University of Rochester, Miner Libraries, Rochester, New York

Stephanie M. Swanberg, AHIP - User Services Librarian, Moustakas Johnson Library, Michigan School of Psychology, Farmington Hills, Michigan

Laura Zeigen, MA, MLIS, MPH, AHIP - Health Sciences Education and Research Librarian, OHSU Library, Portland, Oregon

Amy Allison, AHIP - Associate Dean for Library Services, Geisinger Commonwealth School of Medicine, Scranton, Pennsylvania

Abe Wheeler

Hilary M. Jasmin, MSIS - Assistant Professor, Research and Learning Services Librarian, Health Sciences Library, Memphis, Tennessee

Heidi Reis - Information and Research Services Librarian, Laupus Health Sciences Library, East Carolina University, Greenville, North Carolina

Session Overview: Are you interested in doing more information literacy instruction, but aren’t sure how to talk about its value in a way that administrators and faculty can understand? Our experts in curriculum development are here to help! With a panel discussion on curriculum mapping and information literacy offerings, plus a hands-on opportunity to map your program’s courses and specialties to professional competencies, this session will give you a blueprint for using competency-based curriculum mapping to expand and advocate for your instructional offerings.

Instructional Methods: This session will consist of two major components: a panel discussion and facilitated small group activities. Expert panelists will discuss their experience using professional competencies to develop and advocate for information literacy curricula at their own institutions. After the panel session, participants will have time to work with others in their disciplines as well as the expert panelists to use provided outlines in order to conceptualize a competency-based information literacy curriculum at their institution. Worksheets with information on relevant professional competencies will be provided to facilitate this process.

Participant Engagement: Participants will have time to create their own basic curriculum maps in small groups based on their liaison areas. These maps will facilitate creation of session and/or course learning outcomes based on program-specific professional competencies.

Sponsor(s): MLA Libraries in Health Sciences Curriculums Caucus, MLA Pharmacy and Drug Information Caucus, MLA Medical Library Education Caucus

Redefining Professionalism: Reflection on What Professionalism Looks Like in 2023

Track: Professionalism & Leadership

Soph Myers-Kelley - Medical Librarian, East Carolina University, Greenville, North Carolina

Chloe Hough, AHIP - Reference Librarian, Tampa Bay Regional Campus Library, Florida

Jamia Williams - Consumer Health Program Specialist, University of Utah/The Network of the National Library of Medicine/Training Office, Rochester, New York

Melissa L. Rethlefsen, MSLS, AHIP - Executive Director & Professor, University of New Mexico, Health Sciences Library and Informatics Center

Maud Mundava - Campus Head/Assistant University Librarian, A.T. Still Memorial Library, Kirksville, Missouri

Session Overview: Professionalism as a term and approach has historically been weaponized in an effort to maintain the status quo throughout the information profession, including within libraries. Join us in this immersion session to unpack what professionalism historically has represented and who benefits from being considered a professional by default. Learn with us about the importance of framing professionalism in a more equitable, inclusive way by individually redefining professionalism as well as strategizing how to support everyone who works in libraries so they feel empowered as professionals.

Instructional Methods: The session will start with a panel of speakers covering the topic of professionalism and why the term is outdated. Speakers will share recommendations for how participants can reframe the term to make the workplace more inclusive. After the panel presentations, participants will play a version of the board game Life so they can put themselves in the shoes of others with differing identities.

Participant Engagement: There will be a Q&A session for the panelists and we plan to use polling software to allow participants to “vote up” questions posed by others. The Life board game will be played at each table and will give participants an opportunity to interact in small groups. After game play, tables will be asked to volunteer insights learned from the game.

Sponsor(s): MLA Professionalism and Leadership Hub, MLA Hospital Libraries Caucus, MLA Pharmacy and Drug Information Caucus, MLA Social Justice & Health Disparities Caucus, MLA Latinx Caucus

Seeing Isn’t Believing: Identifying Visual Health Misinformation and Results from a Pilot Class

Track: Education

Kelsey Cowles, AHIP - Research & Instruction Librarian, University of Pittsburgh Health Sciences Library System, Pittsburgh, Pennsylvania

Rebekah Miller - Research & Instruction Librarian, University of Pittsburgh Health Sciences Library System, Pittsburgh, Pennsylvania

Rachel H. Suppok - Research and Instruction Librarian, Health Sciences Library System, University of Pittsburgh, Pittsburgh

Session Overview: Discussion of health literacy instruction and the identification of health misinformation often centers around fact-checking or debunking written materials. However, identifying misleading visualizations and imagery is a vital skill for navigating the current health information landscape. This session will increase your confidence in analyzing visual information and empower you to pass that knowledge along to your communities. We will share results from the pilot of a new class developed for a general university audience and other practical ideas for library programming.

Instructional Methods: We will utilize extensive case studies to illustrate concepts. Audience members will be engaged with numerous hands-on activities to practice identifying different types of visual health misinformation. We will also allow ample time for Q&A and discussion.

Participant Engagement: Participants will be asked to analyze examples of misleading health visualizations and engage in discussion at several points throughout the session.

Systematic Review Services: What Works Best for You and Your Users?

Track: Information Services

Lisa A. Marks, AHIP - Director, Library Services, Mayo Clinic Libraries, Arizona, Scottsdale, Arizona

Emily Brennan, MLIS - Associate Professor, Medical University of South Carolina/Research and Education Services, Charleston, South Carolina

Lisa Liang Philpotts - Infection Control RN, Massachusetts General Hospital, Massachusetts

Whitney A. Townsend - Informationist, University of Michigan, Ann Arbor, Michigan

Michelle L. Zafron, AHIP - Associate Librarian/Coordinator of Reference Services, University at Buffalo, Health Sciences at Abbott Library, Buffalo, New York

Session Overview: When it comes to systematic review services offered by libraries, one size does not fit all. One must consider institution type, librarian expertise, patron demand, available resources, among other considerations, when deciding how robust of a systematic review service to offer. During the panel discussion, librarians from a variety of settings will describe their systematic review programs to illustrate the range of possibilities. Attendees will then break into round table discussions with specific topics related to a systematic review service (e.g., how to get started; memorandums of understanding; patron education; engaging stakeholders).

Instructional Methods: This session will offer a panel discussion, round table discussions, and a question & answer period. The panel discussion will be informational, presenting a variety of systematic review services. Round table discussions will provide participants with the opportunity to gain more in-depth knowledge about a specific topic. The question-and-answer period will ensure that all lingering answers are addressed. We will also provide supplemental materials with examples of systematic review services, factors to consider when developing a service of your own, and communication templates.

Participant Engagement: Panel discussion followed by round table group discussion with participants followed by question-and-answer session.

Sponsor(s): MLA Systematic Review Caucus

Teaching Critical Appraisal through Small Group Learning: A Systematic Review Class Approach

Track: Education

Juliana Magro - Education and Research Librarian, NYU Health Sciences Library, New York, New York

Joey Nicholson - Chair and Director, NYU Health Sciences Library, NYU Langone Health

Caitlin Plovnick - Lead, Education & Curriculum Integration, NYU Health Sciences Library, New York, New York

Dorice L. Vieira - Clinical Outreach & Graduate Medical Education Librarian, NYU Health Sciences Library, New York, New York

Gregory Laynor - Systematic Review Librarian, NYU Health Sciences Library, New York, New York

Richard McGowan - Head, Education and Clinical Support, NYU Health Sciences Library, New York, New York

Session Overview: In this session, participants will experience an adaptable, interactive approach to teaching critical appraisal of systematic reviews. Participants will be guided through the preparation and materials needed to deliver the class, after which they will experience the active learning elements of the class from the learner's perspective, ending with a reflection on the experience and discussion of the lesson's adaptability to different institutions and programs.

Instructional Methods: Presenters will introduce themselves and briefly outline their role in teaching critical appraisal in general, and in this particular systematic review appraisal class. They will then engage session participants in the critical appraisal activity, presenting a task to be completed in small groups. They will then engage session participants in the critical appraisal activity, presenting a task to be completed in small groups. After completion, small groups will then report back to the large group, voting on their choices for the questions guided by the facilitators. The session will conclude with a reflection on the structure of the approach and a discussion of how participants may implement it at their home institutions.

Participant Engagement: The small group task includes: (1) reading a brief systematic review; (2) completing one section of a modified critical appraisal checklist provided by the facilitators, and, as a group, agreeing on answers to the checklist questions. The small groups will then report back to the large group, voting on their choices for the questions, guided by the panelists. For each question, panelists will also provide a comparison from a different systematic review which addresses the same topic and was published at the same time but offers a divergent conclusion. Finally, each small group will have an opportunity to comment on (a) their thoughts about the structure, and reflections about teaching critical appraisal following this approach, for example, (b) is it engaging?, (c) does it achieve the goal of learning what is necessary for critical appraisal?, (d) do participants have the resources needed to achieve this kind of session at their institution?, and (e) what barriers might exist to teach critical appraisal following this structure.

Sponsor(s): MLA Systematic Reviews Caucus, MLA Libraries in Health Sciences Curriculums Caucus

The Third Dimension: Supporting Curriculum through 3D Printing in Your Library

Track: Education

Debra L. Loguda-Summers - Public Service and 3D Print Service Manager, A.T. Still Memorial Library, Kirksville, Missouri

Hal S. Bright, IV, MLS/AHIP - University Library Director, A. T. Still Memorial Library, Phoenix, Arizona

Jesse Roitenberg

Session Overview: This presentation will discuss the increased use of 3D printing in educational settings. 3D printing facilitates learning, creativity, and problem-solving. In addition, we will cover a brief history of 3D printing and the benefits/potential issues of having a 3D program at our library.

Instructional Methods: A hands-on practicum will begin our session by demonstrating a current instructional method using 3D printing in the classroom implemented by a medical school. A panel session will follow using a multimedia presentation and a three-to-four-person panel discussing the history of 3D printing, the 3D printing service at a small health sciences library (HSL), the benefits and limitations of establishing a 3D printing lab/service, and the current cutting edge applications of 3D printing in the medical and health sciences library fields. We will then have a small group brainstorming session on how 3D printing could be used or is used in our own institutions to generate ideas for the group as a whole.

Participant Engagement: An instructional session of preparing and painting 3D take-home models for learning anatomy will begin our session and will continue during the panel discussion. A small group exercise will conclude our session by generating ideas for use at each participant's home institution.

Sponsor(s): A.T. Still Memorial Library-A.T. Still University and Stratasys

Understanding and Applying the Evidence-Base in Library and Information Science.

Track: Innovation & Research Practice

Heather N. Holmes, AHIP - Associate Director of Libraries, MUSC Libraries, Charleston, South Carolina

Jonathan D. Eldredge, AHIP, FMLA, PhD - Professor and Evidence-Based Practice Librarian, University of New Mexico, Albuquerque, New Mexico

Margaret A. Hoogland, MLS, AHIP - Associate Professor and Clinical Medical Librarian, The University of Toledo, Toledo

Margaret Henderson, AHIP - Health Sciences Librarian, San Diego State University Library, Ramona, California

Marie T. Ascher - Lillian Hetrick Huber Endowed Director, Phillip Capozzi, M.D. Library / New York Medical College, Valhalla, New York

Session Overview: The session will include a brief didactic session, followed by interactive roundtable discussions, and conclude with a think-pair-share activity. Participants will gain a more solid understanding of what Evidence-Based Library and Information Practice is. Through roundtable discussions, participants will work with others who have similar interests to develop ideas for projects to do in their libraries. They will leave the session with more confidence to approach EBLIP projects as well as with having a connection to peers who they may collaborate with on future projects.

Instructional Methods: A notice about the session will be shared in advance via MLA Connect which will invite potential participants to submit questions for projects they would like feedback on or to suggest topics in advance. Additionally, citations for seminal readings on EBLIP will also be made available through the same MLA Connect article for those who would like to do some “pre-work” before attending the session. During the session, a didactic overview of EBLIP and model of practice will be provided from which participants will move into roundtable working groups to further discuss ideas and approaches to answering their questions. Participants will pair up to discuss potential next steps at their institution, share their ideas, and write out one of their goals. Approximately six months after the session, the coordinators will send a follow-up message to all participants to check in on progress of the goal and to address any questions that may have arisen since returning to work.To conclude the session, we will lead a brief discussion on next steps and allow time for wrap-up questions from participants.

Participant Engagement: The majority of the time will be spent with the participants in roundtable discussions discussing topics and ideas to help answer their research questions. Each table will have a facilitator to guide the discussion. A Think-Pair-Share exercise will be used to generate in-depth discussion and share the results with the larger body of participants. After the session, participants will receive a list of all topics discussed as well as contact information for the other participants to foster collaboration and peer support.

Sponsor(s): MLA Clinical Librarians and EBP Caucus, MLA Research Caucus, MLA Medical Library Education Caucus, MLA Public Health/Health Administration Caucus

PAPER PRESENTATIONS: RESEARCH ABSTRACTS

We have sorted paper presentation abstracts in this section by title in alphabetical order and note presentations available to conference attendees on-demand.

Advancing the Role of Libraries in University-Wide Electronic Research Notebook Conversations at a R1 Institution

Track: Information Management | On Demand

Plato L. Smith II - Data Management Librarian, University of Florida, Academic Consulting Research & Services (ARCS), Gainesville, Florida

Objectives: Electronic lab notebooks, commonly referred to as electronic research notebooks (ERNs) to denote scope beyond traditional wet lab, can improve data management and sharing within, across, and outside of research environments. The objective of this proposal is to articulate the development of university-wide ERN efforts at [institution] from 2020 to 2022. 1. How can the libraries start the conversation in exploring electronic research notebooks? 2. What is the role of libraries in institutional-wide electronic research notebook exploration? 3. How can the libraries as stakeholder support the university in the adoption and implementation of an institutional-wide electronic research notebook solution?

Methods: The project used a mixed-method research approach of a quantitative and qualitative Qualtrics survey in 2020 followed by participant action research stakeholder approach in 2022. The participant action research stakeholder approach included an informal ERN task force, a formal University ERN Committee, and informal conversations with external organizations. The University ERN Committee developed 25 questions for three selected ERN vendors. The three ERN vendors were invited to give a production demonstration at [institution]. Recommendations for two ERN solutions were narrowed down to one resulting in submission to the Office of Provost. Informal ERN conversations with two external institutions provided additional ERN experiences, insights, and perspectives which were helpful to the informal ERN task force at [institution].

Results: The results from an IRB-approved 2020 survey provided the foundation on which to develop an ERN presentation and published journal article in 2022 that led to development of an informal ERN task force which influenced development of an official University ERN Committee. A price quote for three ERN vendor solutions was submitted to the Office of Provost for consideration. The official University ERN Committee developed 25 questions for ERN vendors to answer prior to vendor presentations at [institution]. The recommendations resulted in two ERN vendors then one vendor. A final recommendation with price quote was sent to Office of Provost.

Conclusions: Based on conversations with external organizations, the College of Medicine led ERN exploration, adoption, and implementation without the libraries involved in the process except for one institution in which the libraries serves as the administrator for the institutional-wide license for support and training. In order for an institutional-wide ERN to be successful, collaborations across multiple units, particularly the libraries a stakeholder, are key in exploration, evaluation, adoption, and implementation. An official University ELN Working Group comprised of management, PIs, research, academic units, scientists at all levels and the institute's IT department and the Libraries is best qualified for formal assessment and selection of an institution-wide ELN solution. The Libraries as collaborator, partner, and stakeholder in this

project was socio-technically enlightening.

An Analysis of Publications Regarding Scholarly Activity among Osteopathic Education Institutions

Track: Education | On Demand

Hannah Pollard - Scholarly Communications Informationist, Strauss Health Sciences Library, Education & Research

Molly Montgomery - Director of Library Services, ICOM Library, Meridian, Idaho

Megan DeArmond - Systematic Review Services Librarian, Touro University Nevada, Jay Sexter Library, Henderson, Nevada

Katie Hoskins, AHIP - Research and Instruction Librarian, California Health Sciences University, CLOVIS, California

Objectives: The research question for this project focused on understanding the scope of publications and conference presentations related to the topic of scholarly activity at osteopathic institutions. Our objectives were to describe scholarly activity trends in osteopathic medical education from 2010-2020 and to identify opportunities for librarians to support scholarly activities at their institutions.

Methods: We conducted a secondary analysis from a larger scoping review that looked at trends in academic and clinical settings for osteopathic medical education in the United States from 2010 - 2020, excluding any references to COVID-19. The research pulled was related to staff, faculty, or trainees in colleges of osteopathic medicine, residency training, or other settings where osteopathic medical education occurs. The secondary analysis focused on all articles related to scholarly activity and research in osteopathic medical education. We completed textual analysis to identify the primary features of the works, such as identifying the status (student, faculty, etc.) of primary investigators or the article's topic. We also identified larger themes within the sources and opportunities for medical librarians to impact education related to scholarly activity and research.

Results: From the initial 1,200 results included in the larger scoping review, we pulled approximately 100 works whose primary focus was scholarly activity. These works included peer-reviewed articles, opinion pieces, and conference abstracts. Some of our topics include but are not limited to, rates of osteopathic authorship in specialty journals, increasing research and scholarly activity at the student level, and limitations of and challenges inherent in doing research in an osteopathic setting.

Conclusions: This project highlights opportunities for librarians who support osteopathic students, faculty, and physicians to get involved in research as more osteopathic medical schools and residencies increase their research and scholarly activity focus. Librarians are uniquely suited to be involved in education, collaboration, and support in the research process and may be underutilized in osteopathic educational settings.

Analyzing Changes in Work-from-Home Policies for Academic Medical and Health Science Librarians

Track: Professionalism & Leadership

David Petersen, AHIP - Assistant Professor, Research and Learning Services Librarian, Preston Medical Library, University of Tennessee Graduate School of Medicine, Knoxville, Tennessee

Matthew Covey - University Librarian, The Rita & Frits Markus Library, The Rockefeller University, New York, New York

Janet Crum, AHIP - Director, Health Sciences Library, University of Arizona, Tucson, Arizona

Objectives: In early 2022, researchers surveyed senior administrators in Association of Academic Health Science Libraries (AAHSL) member libraries to examine work-from-home employment policies. In this follow-up project, researchers survey faculty and staff at AAHSL libraries probing whether those libraries still allow remote or hybrid work and assessing whether faculty/staff perceptions of work-from-home policies are similar to or different from the previously surveyed administrators. Researchers wish to discover if/how work from home (WFH) policies have evolved over the last year and whether those policies appear to influence morale, recruitment, and retention of library faculty and staff.

Methods: Researchers constructed a 25 question survey in Qualtrics and sent it electronically to several email lists and online communities frequented by academic health sciences library employees (e.g., AAHSL-all, medlib-l, various MLA caucus and chapter lists). Questions focused on current WFH policies, staff satisfaction with the policies, and the effect of these policies on morale, recruitment, and retention. The survey also included some questions about respondent demographics, geographic region, and type of institution. An open-ended question was included for library staff to make additional comments.

Results: Results were tabulated in Qualtrics and analyzed. There were 410 results, of which 383 met inclusion criteria. Respondents represented public and private institutions, various position lines (e.g. administration, librarian, library staff), and all regions of the United States. Results showed that remote/hybrid work options continue to be available in many academic health science libraries, and that this feature is important to employees. Additional findings indicated the difficult task of implementing a successful remote/hybrid work policy for all library employees. In the future, researchers will compare the survey's results with the AAHSL Library Director’s survey from early 2022.

Conclusions: Researchers believe the data will be useful to leaders in academic health sciences libraries who are developing or reviewing WFH policies in their libraries.

Attitudes on Data Reuse Among Residents in an Urban Internal Medicine Program

Track: Education

Fred LaPolla - Research and Data Librarian, Lead Data Education, NYU Health Sciences Library/Data Services, New York, New York

Genevieve Milliken, MA, MSLIS - Data Services Librarian, NYU Health Sciences Library/Data Services, Wallkill, New York

Colleen Gillespie, PhD - Associate Professor, Institute for Innovations in Medical Education, New York, New York

Objectives: Secondary data analysis, or data reuse, is a topic of growing interest with professional ramifications for librarians working in data curation, management and sharing with growing relevance to clinical work due to the availability of electronic medical record data and large population level health surveillance data sets. Medical residents may provide useful insight on data reuse as they represent future

clinicians, still exploring research avenues, and their views may highlight forward looking trends. In this study, we sought to understand what internal medicine residents’ attitudes were toward secondary data analysis.

Methods: We surveyed internal medicine residents in three distinct tracks (Primary Care (PC), Categorical, and Clinician-Investigator (CI)) using questions based on a 2011 NSF-supported DataOne survey [1]. While all tracks focus on clinical care, PC has a greater emphasis on ambulatory care, Categorical has a greater focus on inpatient care, and CI has a greater research focus. In discussions with residency directors and the chief resident, the term “secondary data analysis” was chosen over “data reuse” due to this being more familiar to clinicians, but examples were given to define the concept. Core domains assessed in the survey included: X, Y and Z. Survey data collected was analyzed using R version 4.0.3 and RStudio Version 1.3.1093.

Results: We surveyed a population of 162 residents, and 67 residents (41%) responded. A strong majority of residents, 55 (88.7%) out of 62 who answered the data reuse questions, felt secondary data analysis is worthwhile. 49 (79.0%) out of 62 stated that residents should be trained in secondary data analysis. The PC residents, who have curricular exposure to data reuse, more strongly endorsed positive aspects of secondary data analysis, for example 61% stating disagreement with the idea that secondary data analysis is more difficult than other types of analysis, as compared with 41% for Categorical and 42% for CI.

Conclusions: The survey reflects that residents believe secondary data analysis is worthwhile and this highlights opportunities for data librarians. In particular those who have teaching roles in their residency programs may be able to leverage data curation and analysis expertise to help increase data reuse education in graduate medical education. As today’s residents become tomorrow’s clinicians, educators and researchers, libraries have an opportunity to bolster support for data curation, sharing and education, including research data management, data analysis, and data visualization. 1. Tenopir C, Allard S, Douglass K, Aydinoglu AU, Wu L, Read E, Manoff M, Frame M. Data Sharing by Scientists: Practices and Perceptions. PLOS ONE. 2011 Jun 29;6(6):e21101.

Consolidating and Delivering Comprehensive Library Services Following a Health Care System Merger or Acquisition: The e-Delphi Research Experience

Track: Innovation & Research Practice | On Demand

Stacy F. Posillico - Senior Librarian, Northwell Health Eastern Region Hospitals, Hempstead, New York

Jaclyn Morales, AHIP - Senior Librarian, North Shore University Hospital at Northwell Health, Manhasset, New York

Saori Wendy Herman, AHIP - Director of Health Sciences Library, Assistant Professor of Science Education, Donald and Barbara Zucker School of Medicine at Hofstra/Northwell, Hempstead, New York

Objectives: While there are well-researched standards for healthcare executives to follow when healthcare system mergers occur, little empirical research is available for librarians to use in these situations. As a result, librarians are often left to develop their own methods of providing access and services in the newly merged system. In the Fall of 2020, the research team began to conceptualize an e-Delphi study intended to achieve expert consensus from library professionals in the development of research-based recommendations for delivering library services through one comprehensive, geographically distributed system following a merger or acquisition.

Methods: In 2021, the research team developed an e-Delphi research study protocol after exploring the

literature and consulting experts on the e-Delphi research method and hospital librarianship. The protocol was presented at professional conferences later in 2021, and Institutional Review Board (IRB) approval with “Exempt” status was granted in January 2022. Shortly thereafter, expert panelists for the study were recruited through email campaigns to library professionals from each of the seven NNLM regions. In Spring 2022, the research team reviewed the qualifications of the applicants and invited those that qualified to be a part of the expert panel. The team also created a validated qualitative questionnaire to begin the e-Delphi process. In April 2022, the Round 1 questionnaire was sent to expert panelists who were asked to identify key tasks to be addressed during a healthcare merger or acquisition. Thematic analysis was performed over Summer 2022, and the process served as the foundation for the quantitative Rounds 2, 3, and 4 questionnaires, which were completed by the panelists in Fall and Winter 2022. Data extraction and analysis was completed over the Winter of 2022 through 2023.

Results: The research team expects to publish the results in 2023. This paper presentation will provide a deep dive into the researchers’ experience with conducting an e-Delphi study involving an expert panel of librarians from around the country.

Conclusions: This conference paper presentation will be a transparent and in-depth exploration into the research study’s methodology. It will also serve as an opportunity for medical and special librarians to gain a greater understanding about what it takes to successfully carry out an e-Delphi research study.

Cross-Discipline Collaboration: Medical and Law Librarians Identifying and Correcting Uses of Law Databases in the Medical Literature

Track: Innovation & Research Practice

Cecelia J. Vetter - Liaison Librarian, Ruth Lilly Medical Library, Indianapolis, Indiana

Benjamin J. Keele - Associate Director & Lecturer in Law, Ruth Lilly Law Library, Indiana University Robert H. McKinney School of Law, Indianapolis, Indiana

Lee Little - Research and Instruction Librarian & Adjunct Lecturer in Law, Ruth Lilly Law Library, Indianapolis, Indiana

Objectives: The research team of one medical librarian and two law librarians explored the question “Are legal databases correctly used by medical researchers?” The narrow coverage of literature within legal databases differs significantly from the wide scope of literature found in medical databases; therefore, medical researchers may not understand the limitations of legal databases. Both law librarians on the research team assisted medical faculty with medical-litigation papers with significant research limitations because legal databases lack a comprehensive list of case outcomes. This team hopes to provide guidance for medical and law librarians supporting medical-litigation research.

Methods: The research team focused on the use of LexisNexis and Westlaw, the two most common legal databases utilized in medical literature. The medical librarian conducted a search in PubMed for direct references to LexisNexis and Westlaw, retrieving over 600 articles. The research team screened articles to remove irrelevant references and then reviewed full-text articles to identify common uses of legal databases in medical literature. While examining the literature, both types of special librarians had to educate the other on research practices within their respective field. The medical librarian taught the law librarians about systematic review methodology and levels of evidence within medical literature. The law librarians explained how law databases are limited to literature such as appellate cases and state and federal statutes, while excluding litigation outcomes when cases are not appealed or settled out of court.

Results: The research team found that certain types of articles in PubMed, for example surveys of statutes, appropriately searched LexisNexis and Westlaw , while articles that focused on the legal outcome of medical malpractice suites inappropriately used law databases to make claims about case outcomes. For example, using systematic review methodology in legal databases can lead researchers to make incorrect assumptions about the legal landscape. Given the narrow coverage of literature in legal databases, if medical researchers approach those databases as having a similar scope to medical databases, they risk making inaccurate claims about the status of medical malpractice litigation outcomes.

Conclusions: Examining the use of legal databases in medical research required expertise from both medical and law librarians. Our role as special librarians siloes us to different parts of campus, but we learned there is a need to collaborate when supporting faculty conducting cross-discipline research, such as medical-litigation research. The team’s next steps include alerting medical librarians to the limitations of legal databases, so they can deter researchers from incorrectly making conclusions about the legal landscape of medical malpractice.

Developing and Validating the Nursing Student Misinformation Attitudes, Confidence, and Knowledge Assessment Tool

Track: Innovation & Research Practice

Rebekah Miller - Research & Instruction Librarian, University of Pittsburgh Health Sciences Library System, Pittsburgh, Pennsylvania

Theresa Koleck, PhD, RN - Assistant Professor, University of Pittsburgh School of Nursing

Urszula Lechtenberg - Learning Design Coordinator, University of Pittsburgh University Library System

Objectives: Misinformation has become an increasing concern in healthcare. But how much do students know about health misinformation, and how confident are they when trying to mitigate it? To find out, we are developing a survey instrument to understand nursing students’ attitude, confidence, and knowledge about misinformation in the patient care setting. This survey will inform the development of open educational modules that will teach students about health misinformation as well as two strategies for mitigation: pre-bunking and motivational interviewing.

Methods: Our interdisciplinary team designed the 7-item Nursing Student Misinformation Attitudes, Confidence, and Knowledge Assessment Tool. To evaluate content validity, we assembled a panel of expert judges (n=10) in nursing, misinformation, and instrument development. Experts scored each item on the tool in terms of necessity, relevancy, clarity, and importance. We calculated content validity ratio (CVR), content validity index (CVI), multi-rater kappa, and impact score. Judges also provided qualitative feedback on item wording. We revised the tool based on expert recommendation. Then, to obtain baseline information and evaluate reliability, specifically internal consistency using Cronbach’s alpha, we invited n=211 baccalaureate sophomore-level nursing students to complete the tool electronically using Qualtrics. The final tool features five quantitative items that use a 5-point Likert scale from 1 - “not at all” to 5 - “extremely” and two free response items. The tool will be used to evaluate the impact of our open educational modules on nursing student attitude, confidence, and knowledge about misinformation at an R1 research university as part of a required course on nursing research and evidence-based practice. The tool will be made available to the research community at large.

Results: Experts determined that all items were relevant (item CVI=0.9-1; kappa=0.9-1) and important (impact score=3.52-4.9). Further, experts deemed all items necessary (CVR=0.8-1), with the exception of an item asking students to provide a short definition of misinformation (CVR=0.2). Five out of the seven items required revision to improve clarity (item CVI=0.7). Thus, the tool was revised based on expert qualitative

feedback. N=115 (54.5%) nursing students completed the revised tool. Students endorsed the importance of addressing health misinformation with patients and their families or caregivers (mean=4.66, SD=0.54) and other health care providers (mean=4.6, SD=0.65) but reported much lower confidence in their ability to address health misinformation (mean=2.90, SD=0.83; mean=2.57, SD=0.93, respectively). Likewise, students report that their nursing education has moderately prepared them to address health misinformation (mean=3.2, SD=0.99). The tool had adequate internal consistency (Cronbach’s alpha=0.73).

Conclusions: As health misinformation continues to proliferate, educating future health care professionals is of utmost importance. Having a tool to help understand students’ baseline comfort with (and understanding of) dispelling misinformation will be essential when building educational tools.

Development and Validation of a Database Filter for Study Size

Track: Information Services

Wichor M. Bramer - Information specialist, Erasmus MC - Medical Library, Gouda, Zuid-Holland, Netherlands

Sabrina Gunput

Elise Krabbendam

Maarten Engel - Biomedical Information Specialist, Erasmus MC, Netherlands

Objectives: Researchers performing systematic reviews often limit the search results to a certain study size: "we include only studies of more than 100 patients." While we of course can discuss about the validity of such a request, limiting the search results to match the inclusion criteria can reduce the burden of screening for reviewers. The aim of our study was to develop a filter in embase.com and Medline Ovid to retrieve references above a certain threshhold of sample size. We compared the effectivenss of our filter in development using existing systematic reviews that report using sample size as an inclusion criteria.

Methods: Together with researchers who expressed the desire to limit search results to a certain number of patients we constructed preliminary filters which were tested on the spot by evaluating the patient numbers of relevant references that had not been retrieved. If the patient numbers matched the inclusion criteria, the filter was adapted to retrieve the missed articles. After several rounds of improvement of the filter the filter was then tested against existing systematic reviews that used sample size as inclusion criteria but did not limit their search to a sample size.

Results: The filter that was developed consists mainly of truncated numbers in proximity with words such as patients, cases, adults, females etc and phrase like "n=". The filter can and should be adapted to the research topic by combining these truncated numbers with specific terms for diseases, interventions, or body parts of interest such as melanomas, surgeries, eyes or knees. The sensitivity of the filter as evaluated on existing systematic reviews was at least 94%. The references that were not retrieved were older articles that did not include the study size in their abstract.

Conclusions: The study size filter is a good way to limit search results to a certain number of patients. It is not 100% sensitive, but few filters are. Current guidelines for abstract formats advice authors to include in their abstract the number of patients in their research. We therefore expect the sensitivity of the filters only to improve for newer studies. A limitation is that the filters are only available in the interfaces of embase.com and Ovid and cannot be translated into PubMed, as the filter uses proximity operators which are not available in PubMed.

Escaping the Mundane: Using a Virtual Escape Room to Assess Epidemiological and Biostatistical Concepts before STEP 1

Track: Education

Julia M. Esparza, AHIP, FMLA - Associate Director/Professor, LSUHS Library, Shreveport, Louisiana

Montie' T. Dobbins, AHIP - Assistant Director for Technical Services, LSUHS Library, Shreveport, Louisiana

David C. Duggar, AHIP - Head, Liaison Section Program, LSUHS Library, Shreveport, Louisiana

Sarah P. Jackson - Education Librarian, LSUHS Library, Shreveport, Louisiana

Erin Ware, MLIS - Reference Librarian, LSUHS Library, Shreveport, Louisiana

Christopher Schmoutz, Ph.D. - Assistant Professor, Department of Pharmacology, Toxicology and Neuroscience, Shreveport, Louisiana

Rachel G. Barlow - Medical Student Researcher, School of Medicine, Shreveport, Louisiana

Ashley A. Barras - Medical Student Researcher, School of Medicine, Shreveport, Louisiana

Carol N. Crochet - Medical Student Researcher, School of Medicine, Shreveport, Louisiana

Kyah Dowell - Medical Student Researcher, School of Medicine, Shreveport, Louisiana

Robert J. Klazynski - Medical Student Researcher, School of Medicine, Louisiana

Rachel K. Parks - Medical Student Researcher, School of Medicine, Shreveport, Louisiana

Wybra J. Price, III - Medical Student Researcher, School of Medicine, Shreveport, Louisiana

Colton R. Toups - Medical Student Researcher, School of Medicine, Shreveport, Louisiana

Objectives: In the last few years escape rooms have become a new modality for medical education. However, only one article in PubMed utilizes an escape room modality to review biostatistical and epidemiological concepts https://doi.org/10.1128/jmbe.00129-21. The primary outcome of this study is to determine if students find reviewing biostatistics and epidemiological concepts for STEP 1 using a virtual escape room based on third-year medical student experiences a good method of review. The two secondary outcomes are to determine if their confidence improves in their knowledge of biostatistical and epidemiological concepts and if they plan to study more.

Methods: This is a minimal risk, anonymous, convenience sample, education research study using qualitative descriptive survey methods. With an estimated population of 152 in the class, the sample size needed to meet a confidence interval of 8 at 95% would be 75. After obtaining IRB exemption, third-year medical students were contacted to help develop a virtual escape room. Using their experiences on their clerkship rotations, students wrote clinical scenarios that involved biostatistical or epidemiological concepts for our fictional patient Mary from her birth to late life. The concepts highlighted by the students were considered as high-yield biostatistical and epidemiological concepts that may be on the STEP 1. After Library faculty reviewed many different ways to develop an online escape room, the Library faculty created a virtual escape room using deck.toys software. The escape room included activities such as jigsaw puzzles, matching, multiple choice questions, and locks. In the pretest and posttest, students were asked about their confidence level in biostatistical and epidemiological concepts. At the end, they were asked if they planned to study the concepts more before taking STEP 1 and if the virtual escape was a good way to review concepts.

Results: Prior to beginning the virtual escape room, students rated their confidence level in biostatistical and epidemiological concepts. Rating results showed that students were less confident prior to going through the

escape room. After the review, using a paired Wilcoxon signed rank test, student confidence ratings in biostatistical and epidemiological knowledge went up from a median of 2 to 3 ($z = -6.327$, $p < 0.001$). Of the students, 64% planned to study epidemiological and biostatistical concepts more after the review. Finally, 77% found the virtual escape room was a good way to assess their knowledge.

Conclusions: A virtual escape room is a good method for students to review epidemiological and biostatistical concepts. Students’ confidence in their knowledge increased and students planned to study these concepts more for STEP 1. We plan to repeat the process again this upcoming year with new students writing the scenarios to build a new virtual escape room.

Evidence-Based Design of Vaccine Hesitancy Information Resources for Health Care Providers

Track: Clinical Support

Margaret Ansell, MLIS, AHIP - Nursing & Consumer Health Liaison Librarian, University of Florida, Health Science Center Libraries, Gainesville, Florida

Lauren E. Adkins, MLIS/AHIP - College of Pharmacy Liaison Librarian, Health Science Center Libraries/University of Florida, Gainesville, Florida

Mary E. Edwards, MLIS, EdD - Associate University Librarian, University of Florida, Health Science Center Libraries, Gainesville, Florida

Jane Morgan-Daniel, MLIS, MA, AHIP - Community Engagement and Health Literacy Librarian, University of Florida Health Science Center Libraries, Gainesville, Florida

Hannah F. Norton, MS, AHIP - Chair, Health Science Center Library - Gainesville, University of Florida, Health Science Center Libraries, Gainesville, Florida

Nina Stoyan-Rosenzweig - Archivist and Historian, Health Science Center Libraries/University of Florida, Gainesville, Florida

Objectives: After conducting interviews to assess the vaccine-related information needs of local health care providers, librarians at [institution] analyzed the resulting data, first thematically by transcript, then focused on the responses to the interview question about information resources. The team will use the results of these analyses to develop vaccine information resources that providers can refer to during conversations with patients and community members about vaccines. The resources will be presented to providers at relevant departmental and community-based meetings.

Methods: Team members analyzed the transcript data from 18 interviews thematically using an inductive coding process that included 1) group pilot coding and code generation, 2) a first round of independent coding by pairs, 3) code reconciliation again in pairs, 4) further development and definition of codes, and 5) a second round of individual coding to incorporate the newly developed codes. Frequency of code use was also recorded and analyzed. A secondary analysis of the interview question “what resources or tools would make these [vaccine hesitancy-related] conversations easier for you?” was conducted, alongside an examination of any parts of the transcripts tagged with the “information gaps” and “patient information needs” codes, to generate more targeted guidance for developing information resources for clinical use.

Results: The initial thematic analysis of the transcripts resulted in the development of 86 unique codes, with the three most frequently-used codes being “providers reducing barriers [to vaccination via] reframing the conversation/education”, “individualized conversations”, and “trust.” Other notable themes included “provider role”, “mis/disinformation”, and “patient/provider emotions.” The targeted secondary analysis revealed that providers' had opinions about resource design (handouts were a popular request), as well as content

(personal stories, resources tailored to the specific reason for hesitancy). In response, the project team has drafted a guide to COVID-19 vaccine hesitancy resources for local providers, which includes resources with printable patient handouts, background information for providers, and a section organized by reason for hesitancy. Resources required thorough evaluation to avoid redundancy, ensure relevance and convenience for the provider, and guarantee updated and timely information.

Conclusions: Conversations about vaccination are nuanced and often are influenced by personal, relational, logistical, and societal factors. While holistic analyses of interview data reveal rich information about the context and experiences of health care providers related to conversations about vaccines, they are limited in the specific guidance provided for the creation of information resources. Similarly, while there are many COVID-19 vaccine information resources available online, finding resources that meet the specific needs of providers can be challenging.

Hardback Life: The Impact of a Hospital Book Club on Staff Empathy and Comradery

Track: Professionalism & Leadership

Lynn Kysh, MLIS, MPP - Clinical & Research Librarian, Children's Hospital Los Angeles, Los Angeles, California

Kyle Horne - Program Manager, Literally Healing / Children's Hospital Los Angeles, Los Angeles, California

Olivia Bates - Volunteer, Literally Healing/Children's Hospital Los Angeles

Kelleen Lopez - Clinical Research Manager, Children's Hospital Los Angeles, Los Angeles , California

Julie Werner - Researcher, Children's Hospital Los Angeles

Objectives: Incorporating the literary arts through book clubs in the health sciences has demonstrated changes in cultural perspective, communication, and daily practice. After determining the feasibility of a recurring book club for a pediatric hospital staff focused on literature for young people, we now seek to examine the potential impact on participants’ reading behavior, comradery, and empathy

Methods: We designed a prospective observational study with mixed methods analysis of three groups: non-participants (n=18), readers (n=31), and reader-attendees (n=62). Non-participants did not read the books nor participate in meetings. Readers read the books but did not participate in meetings. Reader-attendees read the books and participated in meetings. We offered multiple meetings for each book either in-person or online using WebEx (Cisco). We ran three iterations of the book club where readers and reader-attendees received a free copy of the pre-selected book for young people. Reader-attendees also signed up to attend an hour-long book club facilitated by the co-primary investigators where they participated in activities designed to promote reflection and discussion with colleagues. We used the Assessment Scale of the Work Environment-10 and Interpersonal Reactivity Scale as pre- and post-tests among all groups following each iteration of the book club. Descriptive statistics will be used to summarize participant demographics and study measures. Repeated measures ANOVA will be performed to study the effect of group (non-participant, reader, reader-attendee) and time (pre/post-book club) on scores. We recruited 15 participants from different groups for semi-structured interviews at the study conclusion. With permission, we audio recorded the interview which will be transcribed and coded using grounded theory.

Results: At baseline we found no significant differences in Interpersonal Reactivity Index (IRI) score nor Assessment Scale of the Work Environment Survey (AWE) score. We used two-way mixed design ANOVAs to examine change over time in IRI and AWE, respectively, by group. There was a significant main effect of

group on IRI score (F[2,452] = 10.617, $p < .001$), but not of time, nor a significant interaction effect. Post-hoc analysis revealed that the reader-attendees scored significantly higher on IRI than readers ($p = .045$) and non-participants ($p < .001$). Likewise, there was a significant main effect of group on AWE score (F[2,452] = 17.745, $p < .001$), but not of time, nor an interaction. Both reader-attendees ($p < .001$) and non-participants ($p < .001$) scored significantly higher on AWE than readers.

Conclusions: We look forward to completing the qualitative results so that they can be incorporated into the data, allowing us to analyze the full picture. In considering our quantitative results, while the outcomes didn’t support our hypothesis, it has been incredibly helpful in considering which questions we should be looking to answer as this study has ventured into unchartered territory of research on book clubs in hospital settings. Our quantitative analysis is likely reflective of the limited nature of a short intervention with time in between them. In considering that participants self-selected, we find these outcomes interesting in that the measurements reflect the characteristics of the groups. We intend to continue to examine the impact of facilitated book clubs focused on different and potentially more relevant outcomes.

Health Science Libraries Policy Support Preparedness for the Implementation of the 2023 NIH Data Management and Sharing (DMS) Policy

Track: Information Management

Lisa Federer - Acting Director, Office of Strategic Initiatives, National Library of Medicine, North Bethesda, Maryland

Kelly West - Associate Fellow, National Library of Medicine

Justin de la Cruz - Program Specialist, NYU Health Sciences Library, Asheville, North Carolina

Genevieve Milliken, MA, MSLIS - Data Services Librarian, NYU Health Sciences Library / Data Services, Wallkill, New York

Objectives: In January 2023, the NIH will implement the Data Management and Sharing Policy to promote the sharing of scientific data to accelerate biomedical research. The policy requires submission of a data management and sharing plan, which is a new requirement for many researchers, and health science libraries (HSLs) and librarians can play a crucial role in assisting researchers with policy requirements. This study considers libraries’ and librarians’ preparedness for supporting the policy. These results will help elucidate gaps in existing expertise to inform the development of training and resources, as well as provide insight into existing services and expertise.

Methods: This study used surveys to elicit responses from HSLs and librarians about their preparedness for supporting the Data Management and Sharing (DMS) policy. HSL directors were contacted via the Association of Academic Health Sciences Libraries (AAHSL) listserv and responded to one survey on behalf of their institutions. Questions addressed the services HSLs currently offer or plan to offer to assist researchers in complying with the DMS policy, challenges to offering services, and institutional characteristics. A second survey was announced through various listservs and social media hashtags for biomedical and data librarians. This survey geared towards individual librarians asked questions about how prepared respondents felt to engage with researchers on the DMS policy and what additional training or support they would need to to feel confident in assisting researchers with DMS policy questions. Data analysis was conducted using a combination of R and Qualtrics’ analysis features.

Results: Individual librarians reported a high level of preparedness in many of the skills needed to support the DMS policy, although there was a lower level of preparedness for skills related to informed consent and

information privacy. Libraries are also offering a range of services in support of the policy. For services they are not currently offering, the primary reasons for not doing so were lack of staff expertise and bandwidth.

Conclusions: Health sciences libraries and librarians have a range of skills and are providing services in supporting the DMS policy and can serve as an important resource at their institutions. However, some gaps in knowledge do exist and could be addressed by new training opportunities. Librarians also have opportunities to partner with other campus organizations to address researchers' questions related to the policy.

How Do Medical Students Approach Critical Appraisal? Results from a Mixed-Methods Study

Track: Education | On Demand

Laura Menard - Access Services Department Head, Northern Kentucky University, N/A

Amy Blevins - Associate Director for Public Services, Ruth Lilly Medical Library

Cecelia J. Vetter - Liaison Librarian, Ruth Lilly Medical Library, Indianapolis, Indiana

Daniel J. Trujillo, PhD - Director of Assessment, Harrison School of Pharmacy, Auburn, Alabama

Objectives: Our research team wanted to find out what principles and best practices medical students use when prompted by a clerkship assignment to complete a critical appraisal of an article of their choosing. Our hypothesis was that, outside of a structured classroom environment, many students would default to more basic literature evaluation strategies or even apply proxies for methodological rigor such as journal reputation or peer review status of a study.

Methods: All first-year clerkship students at the School of Medicine are required to complete a patient-focused evidence-based medicine (EBM) assignment during their Internal Medicine clerkship. A team of three librarians and one statistician undertook a mixed-methods approach to identify and quantify themes that emerged in the text of one year's worth of these assignments (n=343). A mixed method research approach was implemented to gain a greater understanding of the EBM principles and best practices that students reference in their assignments. Within this approach a qualitative content analysis was conducted, followed by a quantitative analysis of patterns within the sample. The research team used first- and second cycle coding and a collaboratively developed code list of nine major codes to ensure accuracy and standardization. Additionally, the research team's statistician implemented an inter-rater reliability plan and examined inter-class correlations to ensure grading consistency across team members and student assignments. Once all assignments had been coded, the team used statistical analysis to find correlations between codes as well as frequency of code application within the sample in order to identify five major critical appraisal themes which emerged in the students' assignments.

Results: After a rigorous coding process, several codes and related themes emerged. The research team identified nine main codes and five major themes. These themes are as follows: Theme 1: Comparing the study population to the patient being treated and recommending a course of action Theme 2: Identifying study type and position in hierarchy of evidence Theme 3: Identifying proxies for study quality, including provenance and timeliness of chosen study Theme 4: Summarizing study methodology and results Theme 5: Attempting a critical appraisal of chosen study Additionally, we identified correlations between themes as well as frequency of application in the sample.

Conclusions: A few notable results from our analysis of this sample are the frequency with which students were able to summarize the results of their chosen study and apply what they had learned to patient care (54.5% and 46.9% of all assignments, respectively). However, a notable number of students (35.2%)

incorrectly used journal reputation, peer review status, h-index, impact factor, or similar metric, as a proxy for critical appraisal without engaging with the study methodology. This indicates that there is a need for further education and engagement with clerkship directors regarding the utility and application of EBM skills in the clinical curriculum.

"I Wish There Was a One-Stop Shop for a Lot of This": Participant Design of an Infertility Consumer Health Resource

Track: Information Services@@@In Person@@@On Demand

Emily Vardell, AHIP - Assistant Professor, Emporia State University School of Library and Information Management, Olathe, Kansas

Ting Wang, PhD - Lecturer, Emporia State University School of Library and Information Management

Brenda Linares, MLIS, MBA, AHIP - Associate Dean of Library Services, Miller Nichols Library at the University of Missouri - Kansas City

Objectives: By exploring the barriers individuals with infertility face when accessing health information, this study identifies preferred information channels to connect this population with authoritative and relevant medical and mental health information to design an infertility consumer health resource. Infertility impacts approximately one in every eight people; however, there is a lack of information available on the topic and a social stigma that often keeps individuals from seeking or sharing information. Online communities often support individuals and facilitate access to information related to building a family.

Methods: The researchers conducted interviews with members of an online infertility support group. The project used purposive sampling methods to recruit participants through both direct contact (identified from participant observation within the online support group) and through recruitment messages posted in the Facebook group. Virtual, semi-structured interviews consisted of questions about preferred information sources, barriers to accessing needed information, and stigmas they faced as people going through infertility. Each research participant also shared what they would include if they were to design an information resource for a person going through infertility. The findings were analyzed with an open coding scheme, looking for emerging patterns from the transcripts.

Results: Participants described the desire for a “one-stop shop” online information resource, which would include information about medications, treatment timelines, health insurance coverage, online/in-person support groups, mental health, and communicating about this sensitive topic with partners and loved ones. The online information platform could provide varying levels of depth and content regarding infertility depending on where the individual was in their infertility journey. For example, the resource might initially outline the multiple types of infertility and their frequency of occurrence in plain language, then describe the effects of and distinctions between different tests and medications based on the diverse types of diagnoses. As an individual progresses through their infertility journey, their knowledge about infertility increases accordingly, so information with complicated medical terms as well as relevant academic research papers on specific symptoms and treatments is well suited for individuals at a later stage. Providing information based on the timeline helps individuals at various stages find the information they need more quickly. The information can be presented in various forms, such as medical websites, videos, books, forums, and podcasts. Also, participants expressed their desire for information about the success rates of different clinics as well as information on the topic of male infertility.

Conclusions: This study demonstrates the need for an online platform that provides individuals with infertility with comprehensive and accessible consumer health information. Participants highlighted the need for a one-stop shop of information regarding infertility with varying levels of depth and content based on the

individual's timeline and infertility journey. The study provides valuable insights into the information needs of individuals with infertility and potential ways to meet those needs.

Improving LibGuides at a Health Sciences Library Using Feedback from Focus Groups and User Experience Testing

Track: Information Services

Lydia A. Howes, AHIP - Assistant Librarian, Education and Research, Eccles Health Sciences Library | University of Utah, Salt Lake City, Utah

Elizabeth Frakes, MSIS, AHIP - Assistant Librarian for Clinical Services, Spencer S. Eccles Health Sciences Library, Salt Lake City, Utah

Donna Baluchi - Equity, Diversity, & Inclusion Librarian, Eccles Health Sciences Library, Salt Lake City, Utah

Carmin Smoot - Digital Publishing Coordinator, Spencer S. Eccles Health Sciences Library, University of Utah

Nena Schvaneveldt, MLIS - Associate Librarian, Eccles Health Sciences Library, Salt Lake City, Utah

Yingying Zhang

Objectives: While LibGuides are widely used in libraries to curate resources for users, it’s challenging to ensure they are effective. Common problems include guide maintenance, unintuitive design and layout, and irrelevant or overwhelming content. Existing literature concerning effective LibGuide design largely focuses on the general undergraduate population, and findings may not apply to a more specialized, health sciences population. This paper describes the University of Utah Spencer S. Eccles Health Sciences Library’s efforts to include actionable feedback from focus groups and individual user experience (UX) testing, alongside recommendations from the literature, in their efforts toward LibGuide redesign and updated maintenance, content, and design guidelines.

Methods: To gain a better understanding of what users expect from and would like to see in LibGuides, researchers conducted focus groups and UX testing on potential guide designs. Investigators advertised the need for UX testing through the Library website, flyers posted in locations that students frequent and distributed at tabling events, and leveraging existing relationships with professors and students to elicit more participation. Researchers conducted both online and virtual focus groups and individual feedback sessions, lasting 15-30 minutes. Each session was facilitated by one or two team members. The initial goal was to recruit 2-7 students of differing academic levels from a particular program or field of study (e.g. medical students, undergraduate and graduate nursing students) for feedback on useful resources to highlight on subject guides. Following a pilot focus group, investigators began drafting a guide to test via UX tests, informed by themes identified in focus groups and current guide statistics. When possible, UX testing focused on the participants' actions as taken on the screen, but also took into account their narrated thoughts and reactions. A second version of the test guide was drafted midway through the UX testing phase and included in remaining tests. Using the recorded sessions and transcripts, results were coded and analyzed for themes.

Results: In total, the team conducted 6 focus groups and 19 UX tests, speaking with 27 students, 6 staff, and 1 faculty from a variety of health-sciences programs and levels of study. Notable themes related to guide design include: the lack of clear preference between side and top navigation layouts; a disinclination to scroll down long pages or click into multiple tabs without prompting; an appreciation of chunking resources into smaller groups; the preference for visual elements, such as database logos, to aid in locating desired resources; and desire for a consistent layout across university and departmental websites. Themes

related to guide content include: confirmation that most go-to and otherwise expected resources were included in subject-specific guides; the need to rename or rephrase some headings and tabs to more clearly convey to users what to expect; and general confusion over icons intended to represent resource availability and access.

Conclusions: Consulting with the intended audience was instrumental in an on-going LibGuides revamp. While feedback concerning LibGuides was generally positive, focus groups and UX testing identified both areas for improvement and existing features that worked well. These themes will inform updated guidelines and templates, which are currently nearing completion.

Integrative Health-Related Information Needs and Interests of MLA Members

Track: Information Services | On Demand

Terry K. Selfe, AHIP - Translational Research and Impact Librarian, University of Florida, Academic Research Consulting and Services (ARCS), Gainesville, Florida

Yumin Jiang - Head, Collection Management, Strauss Health Sciences Library, University of Colorado Anschutz Medical Campus, Aurora, Colorado

Objectives: As integrative healthcare education and research becomes more widespread across health sciences centers, including traditional medical schools, the Complementary and Integrative Health (CIH) caucus leaders were interested in learning more about the current CIH-related information needs of MLA members and identifying how to best support those needs. To that end, we conducted a survey of MLA members, including those not belonging to the CIH caucus. The purpose of this study was to better understand the integrative health-related information needs and interests of MLA members to inform future CIH caucus activities.

Methods: All MLA members were eligible to participate in this IRB-exempt study. Invitations to participate in the online, anonymous survey were posted to each MLA caucus forum on February 9, 2022, with a reminder invitation posted on February 25, 2022. The invitations contained informed consent language and the link to the survey, which was created using Qualtrics. Survey topics included: the types of CIH-related questions respondents receive (e.g., questions related to instruction, research, clinical practice, patient education), the frequency of these requests, whether their library resources were sufficient to answer questions received, their confidence in their ability to respond to these inquiries given the necessary resources, their level of satisfaction with access to training to further develop knowledge of these resources, content respondents would find helpful from the CIH caucus, and how they would like for this content to be delivered. In addition, we asked about the type and size of their institution, and whether it offers any educational programming or conducts research on integrative medicine or complementary/alternative therapies. Descriptive analysis of the resulting quantitative data was conducted and will be presented.

Results: Of ___ members belonging to at least one caucus, 218 responded to the survey (__% response rate). Only 18 respondents reported being CIH caucus members and less than 3% (n=5) worked at educational institutions based primarily on a complementary/alternative medical model. CIH content respondents would find helpful was as follows: CIH resource recommendations (n=157), CIH database instruction (n=135), instruction on CIH practices for self-care (n=79), and other (n=13). The top three delivery methods preferred by respondents were: via a free workshop/webinar (no MLA CEs) (n=145), in a LibGuide (n=120), and MLA CE workshop/webinar (n=94). [will be updated when info received]

Conclusions: This study suggests there is a broad interest in CIH-related information resources among

MLA members, including those not belonging to the CIH caucus. Given that the vast majority of survey respondents were not CIH caucus members, any future programming by the CIH caucus would ideally be disseminated in a way accessible to non-caucus members. While the subject matter topics we covered in our survey were specific to CIH, the preferred methods of content delivery may be generalizable to other caucuses. Based on our findings, the CIH caucus will explore developing some free workshops on CIH content areas of interest to be delivered via Zoom and open to all MLA members.

Library Faculty and Staff Education Program Survey on Artificial Intelligence

Track: Innovation & Research Practice

Sarah Meyer - Assistant University Librarian, University of Florida, Health Science Center Libraries, Gainesville, Florida

Borui Zhang - AI/Natural Language Processing Specialist, University of Florida, Academic Consulting Research & Services

Aida Miro-Herrans, PhD - Bioinformatics Librarian, University of Florida, Academic Research Consulting & Services, Gainesville, Florida

Hao Ye, PhD - Reproducibility Librarian, University of Florida, Academic Research Consulting & Services (ARCS)

Plato L. Smith II - Data Management Librarian, University of Florida, Academic Consulting Research & Services (ARCS), Gainesville, Florida

Natya Hans - Informatics Librarian, University of Florida, Academic Research Consulting and Services (ARCS), Florida

Michele R. Tennant, AHIP, FMLA - Interim Senior Director, University of Florida, Academic Research Consulting & Services, Gainesville, Florida

Xiaoli Ma, MSI, MA - Metadata Librarian, University of Florida, Resource Description

Tiffany C. Esteban, MSI - Digital Humanities Associate, University of Florida, Humanities and Social Sciences Library West

Objectives: To prepare for the increasing need of academic support in the artificial intelligence (AI) era. A research team at [institution name] libraries is developing an artificial intelligence (AI) educational training series for library faculty and staff. To maximize effectiveness and customize training materials, the team required first-hand information from potential participants.

Methods: The research team collected qualitative data from potential trainees that agreed to participate in a seven-question online survey, to gain insight into the AI information and training needs of library faculty and staff. Questions were aligned to assess AI comfort level; explaining AI concepts to others and identifying technologies and types of resources necessary to support AI education and research. Survey data was analyzed and used to tailor design of a training series. Then, to assess effectiveness of trainings a one-question post-training survey was conducted.

Results: Sixty-seven library employees participated in the survey. Most respondents (25.1%, 26 of 67) were extremely uncomfortable to neutral about explaining and identifying AI. Resource requests spanned topics such as: foundational training, basic terminology, and practical skills in building applications for library services. Survey responses guided creation of a three-part training series and research guide tailored to academic libraries. The inaugural sessions began Fall 2022 and ninety-seven attended. Post-course survey

responses were positive, and learners expressed a desire for more advanced topics such as machine learning, natural language processing, and data visualization.

Conclusions: Assessing the needs of library faculty and staff identified a need for foundational AI training and resource materials. Based on learners’ feedback three new workshops will be added to the series in Spring 2023. Tailoring AI training to specific audience needs may improve effectiveness and willingness to engage in more advanced training. A tailored AI training approach may also be applicable to broader populations such as other types of libraries and non-STEM educators.

Looking Back to Look Forward: 50 Years of Educating Health Sciences Librarians

Track: Professionalism & Leadership | On Demand

Ana D. Cleveland, AHIP, FMLA - Regents Professor, Sarah Law Kennerly Endowed Prof., Director. Health Informatic Program, University of North Texas, Denton, Texas

Jodi L. Philbrick, AHIP - Principal Lecturer, University of North Texas, Denton, Texas

Objectives: The research questions for this study include: 1. How has the education of health sciences librarians evolved over the last 50 years? 2. What has been the nature of the programs and courses for the education of health sciences librarians in the last 50 years? 3. What is the current state of educating health sciences librarians?

Methods: For the historical perspective on the topic, the authors conducted a literature review focused on the topic of the education of health sciences librarians from 1973 to present. Based on a content analysis of the articles, patterns and trends from the literature were examined to create a picture of the education of health sciences librarians for the past 50 years. The number of programs and nature of courses reported in the literature were captured. For the current state of educating health sciences librarians, the authors used the Association for Library and Information Sciences Education (ALISE) 2022 Statistical Report to determine the master's programs in library and information sciences with courses in health sciences librarianship and reviewed and analyzed the websites of the master's programs for more detailed information.

Results: The number of master's programs in library and information sciences (LIS) with courses in health sciences librarianship was stable from the 1970s to the 2000s with an increase in the number occurring in the 2010s. However, in the last decade, the number has decreased. A review of the curriculum section of the Association of Library and Information Sciences Education (ALISE) 2022 Statistical Reports, which has data for 58 LIS master’s programs in the United States and Canada, revealed that less than half (21) of the LIS master’s programs offer courses in health sciences librarianship, with most (10) programs only offering one course. Upon analysis of the master’s programs’ websites, eight (8) had official concentrations in health sciences librarianship. Most of the courses offered focused on an overview of health sciences librarianship and information resources in the field.

Conclusion: From the review of the last 50 years of health sciences librarianship education, there has been a decline in the number of courses and concentrations in this area in LIS master's programs in the most recent decade. Future research should focus on the reasons for this decline.

Mapping New Landscapes: Comparing Web of Science, Scopus, and Dimensions for Reporting Research Impact of New Global Diabetes Research Center

Track: Innovation & Research Practice

Kimberly R. Powell - Research Impact Informationist, Emory University Woodruff Health Sciences Center Library, Atlanta, Georgia

Maithree S. Venkatesan - Graduate Assistant, Emory University, Atlanta, Georgia

Objectives: A Global Diabetes Research Center (GDRC) was recently established at our institution. This project aims to assess and compare the article coverage, impact metrics, and associated funding acknowledgments to the center’s foundational grant (NIH P30) from member publications as provided by Web of Science, Scopus, and Dimensions databases. These databases represent three possible solutions for measuring and reporting journal article outputs and impact of GDRC supported researchers. The ultimate project goal is to recommend a single reporting database, or provide empirical justification for the use of multiple databases, for tracking center outputs, impact, and funding acknowledgements going forward.

Methods: Full bibliographic profiles were created in each of the three databases for GDRC supported investigators. Member CVs were provided by the center director and consulted to assist with name disambiguation and affiliation details- of the approximately 150 member researchers, 30% are not otherwise affiliated with our institution. Results were stored in an EndNote library and analyzed using Excel. Comparisons were made for the overall coverage of journal articles available in each database for the full career bibliography and over the last 5-years (2018-2022), author impact as modeled by the h-index, and the number of reported acknowledgments to the GDRC project grant. Initial assessments were made on the full bibliographic scope of GDRC associated researcher publications. Further assessments will be made on the subset of publications directly related to “diabetes” as determined by a term search within each database.

Results: Data analysis is on going but preliminary results show Scopus provides 13% and 27% greater journal article coverage over Web of Science and Dimensions for full career bibliographies, respectively. Scopus also reports a 20% increase in median h-index over Web of Science, and 40% increase in median h-index over Dimensions for full career bibliographies. Meanwhile, Dimensions identified 61% more publications with GDRC grant acknowledgements than Scopus and 45% more acknowledging publications than identified by Web of Science.

Conclusions: [not entered]

Mobile Technologies for Learning among Medical Graduates

Track: Education

Nosheen F. Warraich, n/a - Director, Information Management, Lahore, Punjab, Pakistan

Faiza Bashir - Assistant Professor, Higher Education Department, Government of the Punjab, Lahore, Pakistan, Lahore, Punjab, Pakistan

Amara Malik - Assistant professor, University of the Punjab, Lahore , Pakistan

Objectives: The use of mobile technologies in the medical field enables mobility and mobile learning. These technologies are not only transforming the healthcare environment but also influencing the landscape of medical education. This ongoing study aims to explore the behavioral intention of medical students while using mobile technologies for learning purposes. To map out the core benefits associated with these mobile technologies through the eyes of medical students in Pakistan is another objective of the study. This research study intends to highlight the limitations of mobile technologies as a learning tool in medical education as well.

Methods: To get the set objectives, the Theory of Planned Behaviour (TPB) will be adopted. MBBS students from public and private sector medical colleges recognized by the Pakistan Medical and Dental Council (PMDC) in Lahore will be the population. A self-structured questionnaire will be used to collect data from the proposed population with the convenience sampling method. The data will be analyzed through SPSS-22 &AMOS.

Results: This study is the offspring of an ongoing Ph.D. project and it is in the data collection stage. Its results will provide valuable insights regarding the application of mobile technologies in medical education in Pakistan. Being the first study of its nature, it is anticipated that the findings of the study will be a valuable contribution to filling the gap in existing knowledge and literature and will offer new insights from a developing country’s perspective. The author believes that the data collection and analysis will be completed in a couple of months and ready to present at the SLA conference.

Conclusions: Mobile technologies allow learning to extend to spaces beyond the traditional classroom. On the other hand, within the classroom, mobile learning increased flexibility and opens new opportunities for interaction. Numerous types of research showed that mobile technologies had a visible impact on the learning environment and academic interactions. There are multiple benefits associated with the use of these technologies in the healthcare education sector as well. Concerned stakeholders and policymakers will also benefit from the results of this study in developing develop a policy that will direct to harnessing the use of mobile technologies as an additional means of learning in medical curriculum and training.

“Next Gen”: Leadership in the 21st Century!

Track: Professionalism & Leadership | On Demand

Catherine B. Soehner, AHIP - Associate Dean and Director, Univ of Utah / Eccles Health Sciences Library, Salt Lake City, Utah

Maud Mundava - Campus Head/Assistant University Librarian, A.T. Still Memorial Library, Kirksville, Missouri

Objectives: A recent article by Crystal Goldman (2020, Journal of Library Administration, 60:1) describes a crisis in librarianship caused by a “high number of upper and middle management positions opening in the past five years and an underqualified applicant pool available to fill those positions.” Health sciences libraries need new and diverse candidates to fill leadership roles. This study aims to answer two questions: (1) What would encourage someone to consider leadership as a path for their career? and (2) What skills, training, experience, and leadership styles are required to lead libraries of the 21st century in the most turbulent times?

Methods: In January 2023, academic health sciences librarians in the United States were invited to participate in an anonymous online survey about potential barriers to considering leadership as a viable career path. The survey was created by the authors using Qualtrics, pre-tested with colleagues at the authors’ institutions, and delivered using a link embedded in an email using email listerservs through the Medical Library Association (MEDLIB-L), the Association of Academic Health Sciences Libraries (AAHSL-

ALL), and the Association of College and Research Libraries Health Sciences Interest Group (ACRL-HSIG). The survey was based on research conducted in 2013 and presented at the American Library Association national conference that year. Survey responses were analyzed using Survey Monkey and Excel. Answers to open ended questions were categorized into broad topics for analysis. This study reflects an update to the previous study to take into account current conditions.

Results: Based on the earlier research conducted in 2013, we expect that survey respondents will identify barriers related to lack of skills, the need for mentorship and succession planning initiatives, adequate financial support, effective training programs, and a desire to gain experience needed for leadership. What is less known is the perception of the kinds of skills needed for leadership and how institutions, professional organizations, and library school curricula are identifying and preparing librarians, particularly librarians from diverse backgrounds, for a career that includes a formal leadership position.

Conclusions: The aims of this study are to establish new mechanisms to attract librarians to leadership in health sciences libraries while increasing the diversity of potential leaders and to influence skills taught in leadership programs. It will establish ways to heighten the desire among librarians to gain experience and skills to be ready for leadership roles. It will examine what is already in place as lessons to enable the next generation leadership to smoothly transition into these roles and eagerly take health sciences libraries into the future. This information is important because the profession needs to proactively put in place strategies that attract a diverse pool of talent which is ready and prepared for future leadership in the 21st century.

Opening Soon: Potential Impacts of the OSTP Mandate on the Biomedical Literature

Track: Information Management | Virtual | On Demand

Edwin V. Sperr, Jr., AHIP - Clinical Information Librarian, AU/UGA Medical Partnership / GME, Athens, Georgia

Objectives: The new mandate from the White House Office of Science Technology and Policy promises big changes in scholarly communication. Within the next four years, peer-reviewed papers from all federally-funded projects will need to be made publicly accessible in a designated repository at the time of their publication. As speculation builds about what this will mean for authors and publishers, the question remains of exactly how much of the biomedical literature will be subject to this mandate. This study attempts to address this question.

Methods: This study centers on searches of the PubMed database. PubMed has broad coverage of the biomedical literature with a particular emphasis on items pertaining to clinical medicine. As PubMed is compiled and maintained by the National Library of Medicine, a U.S. Government agency, records there often contain relevant information about government funding. Using the “Funding Support (Grant) Information in MEDLINE/PubMed” search strategies (https://www.nlm.nih.gov/bsd/funding_support.html), this study seeks to identify the proportion of government-supported items in PubMed for a number of different topics. Searches are repeated using publication date limits of ten years andi five years to ascertain if these results follow a chronological trend.

Results: Initial results confirm that a significant portion of PubMed items would have been subject to the OSTP mandate, as approximately 11% of all PubMed records appear to be funded with federal monies. The proportion of government-funded items returned by individual PubMed searches varies dramatically, from only around 1% of items for the MeSH headings “Venereology”, “Anesthesiology“ and “Osteopathic Medicine'” to around 27% of of items with the publication type of “Clinical Trial, Phase I.” Most notably, a search using one hedge designed to find items published about or by authors based in the United States

would seem to indicate that a full 40% of such items ultimately derive from federally funded projects. Changes over time in the percentage of government funded items varied between searches, in some cases decreasing, while increasing in others. For example, the percentage of federally funded items retrieved using the very broad MeSH heading “Diseases” doubled from 5% in all years to nearly 11% for the last ten.

Conclusions: These findings make clear that making federally-funded articles publicly available at the time of publication will have a significant impact on access to the biomedical literature by the general public. The large proportion of items pertaining to such broad categories as “clinical trials” or “behavioral medicine” that will soon be covered by the OSTP mandate means that non open access publishers of such items may well have to examine their operations going forward.

Osteopathic Medical Student Attitudes towards Research: Consumers or Creators?

Track: Education

Molly Montgomery - Director of Library Services, ICOM Library, Meridian, Idaho

Austin Cuttone, n/a - Medical Student, ICOM

Marisa Helm, n/a - Medical Student, ICOM

Mindy Hoang - Medical Student, ICOM, Great Falls, Montana

Nahleh Koochak, n/a - Medical Student, ICOM

Morton Joshua, n/a - Medical Student, ICOM

Nicholas Rincon, n/a - Medical Student, ICOM

Objectives: The overall research question for this project was to investigate why few osteopathic students pursue research projects and why many hold negative attitudes towards doing research and to understand what research information they want to learn about. The specific objectives include: To describe the attitudes and opinions of osteopathic students regarding research and identify the research-related skills osteopathic students feel they need before graduating from medical school.

Methods: This mixed methods research study included a survey and individual interviews conducted at one osteopathic medical school. The survey was sent to all four classes (both didactic and clinical) with 106 respondents. Survey data was analyzed using descriptive statistics to identify what research skills students value and why they would or would not participate in research as medical students. Fourteen students participated in interviews conducted via Zoom with representation from students from all four years. Transcripts of the interviews were uploaded into a qualitative data analysis software (Dedoose), and the data were analyzed using inductive thematic analysis.

Results: Themes identified from the interviews include the idea that research only means bench/basic science research, students feel pressure to produce research to be competitive without valuing research, they don’t have time to do research, but they want to be able to read and interpret research. The survey data indicates that students understand the importance of research but mostly only do it to be competitive for residency and are more interested in clinical vs. bench research. And as with the interviews, the survey respondents placed more emphasis on skills related to understanding and communicating research than producing research.

Conclusions: Some findings from this study were not surprising, such as the limitations medical students face in terms of lack of time, training, and resources to start a research project and that research is seen mostly as a way to be more competitive for residency. Other findings were more surprising, such as the

strong recommendation for more information on how to read and interpret research and the recurrent theme that research mainly means pipettes and drudge work in a lab. The findings influenced our school’s decision to transition a mandatory, 4-week research rotation in the 3rd year from about creating research projects to more about how to consume and communicate research findings.

Patron and Librarian Perspectives of a Long-Standing Health Science Liaison Librarian Program

Track: Information Services | On Demand

Michele R. Tennant, AHIP, FMLA - Interim Senior Director, University of Florida, Academic Research Consulting & Services, Gainesville, Florida

Carrie D. Adams, MA, LIS; MA, IDT - Program Director, University of Florida Health Science Center Libraries, Borland Library, Jacksonville

Margaret Ansell, MLIS, AHIP - Nursing & Consumer Health Liaison Librarian, University of Florida, Health Science Center Libraries, Gainesville, Florida

Mary E. Edwards, MLIS, EdD - Associate University Librarian, University of Florida, Health Science Center Libraries, Gainesville, Florida

Jane Morgan-Daniel, MLIS, MA, AHIP - Community Engagement and Health Literacy Librarian, University of Florida Health Science Center Libraries, Gainesville, Florida

Hannah F. Norton, MS, AHIP - Chair, Health Science Center Library - Gainesville, University of Florida, Health Science Center Libraries, Gainesville, Florida

Ariel F. Pomputius, AHIP - Health Sciences Liaison Librarian, University of Florida, Health Science Center Libraries, Gainesville, Florida

Objectives: The Health Science Center Library (HSCL) at the University of Florida has operated a liaison program since 1998, with each health-related college or department assigned a liaison librarian as their primary point of contact for library and information services. An extensive assessment of this service model took place in 2003; the current study provides an update to this previous work, using similar data collection tools. This is part of ongoing efforts to periodically request input from faculty, students, and staff about their information needs and whether library services are meeting them. Such input is used to improve library services and the user experience.

Methods: A team of librarians updated the two surveys from the 2003 assessment: one for the faculty, students, and staff the HSCL serves (patron survey) and one for HSCL liaison librarians (librarian survey). The patron survey consisted of 13 questions about participants’ experiences and preferences related to liaison services and 6 demographic questions. The librarian survey consisted of 20 questions about experiences offering liaison services and needed supports from library administration. A number of questions appeared on both surveys, allowing comparison of patron and librarian perceptions of the same issue. Both surveys were approved as exempt by the IRB02 (Behavioral/Non-medical Institutional Review Board) and distributed via email by librarians posting to departmental and college email lists, links on the library website and Facebook page, and email distribution by trusted administrators (e.g., Associate Deans for Education). At MLA’s annual conference in 2022, we highlighted results from the survey of liaison librarians. This presentation will concentrate on results from the patron survey, as well as comparisons of patron and librarian perceptions.

Results: Data from the patron survey indicates that while the majority of patrons are aware of the liaison librarian program, there is a significant number who are not aware of the service, or do not understand how

liaison librarians can support their information needs. Patrons find a number of liaison services very useful, especially assistance with database searches, help using electronic resources, and support for systematic reviews.

Conclusions: The team expects that survey responses will yield valuable insights into how effectively our liaison librarian services meet the needs of our patrons, from the perspectives of those patrons and the liaison librarians themselves. Results will guide any future changes to liaison librarian services at our institution.

Perceptions and Behaviours of First Year Health Sciences Undergraduate Students Conducting Online Research

Track: Education | On Demand

Susanna Galbraith - Virtual Services Librarian, Health Sciences Library / McMaster University, Hamilton, Ontario, Canada

Objectives: To explore the perceptions and behaviours of first year health sciences undergraduate students when conducting online research during their transition from secondary school into university. The study aims to answer the following question: How is the user experience of online library research impacted through increased usage of the resources over time?

Methods: An ethics-board approved qualitative ethnographic study is underway using semi-structured interviews. Students were recruited from an introductory first year health sciences course. They were screened to ensure they are first year students and have no prior higher education experience. 13 students were successfully recruited. These students provided a 1-hour interview using Zoom during the first 3 weeks of their undergraduate degree in the fall of 2022. Transcriptions have been verified by the principal investigator. The data is currently being analyzed using inductive coding methods, and will undergo multiple rounds of coding. Following this a second round of interviews as conducted in February to provide further insights into how their experience has changed over time.

Results: Initial results are very preliminary at the time of this submission. The students interviewed described in detail all aspects of their online research process including: how they made decisions when searching Google; whether they tended to go to peers, instructors or online communities for support; when and why they would turn to YouTube; determining credibility of resources identified through Google; information literacy instruction they received during their secondary school years.

Conclusions: The data is currently being analyzed, however much can be learned from this study. The conclusions will help to inform information literacy instruction for health sciences undergraduates, collection development and user experience design of library websites.

Planning for Data Services: Researcher Needs & Perspectives on Data Management and Sharing

Track: Information Management

Jess Newman McDonald - Research Data & Scholarly Communications Lead, Health Sciences Library, Memphis, Tennessee

Sarah Newell - Assessment and Data Services Librarian, Health Sciences Library, Memphis, Tennessee

Objectives: The National Institute of Health’s (NIH) Data Management & Sharing (DMS) Policy requires researchers to prospectively plan for how scientific data will be preserved and shared. [Institution] Library faculty began to field questions about the upcoming policy in the spring of 2022 with respect to the Library’s and campus’s plans for assisting researchers in compliance. These conversations greatly energized efforts to assess and define the Library’s role in providing research data services. Prior to developing new services, the authors set out to better understand researcher needs and perspectives in order to identify service gaps, collaboration opportunities, and existing expertise.

Methods: [Library] serves an academic health science center with five colleges: Medicine, Nursing, Dentistry, Graduate Health Sciences, Pharmacy, and Health Profession. 60-minute semi-structured data interviews were conducted via Zoom with 10 [Institution] faculty researchers. Participants were selected from a variety of health science disciplines and faculty statuses in order to engage with multiple perspectives. Interview questions discussed whether researchers currently share research data, which campus data services or support units they utilize, approaches and barriers to data management, and their thoughts on the new NIH Data Management and Sharing Policy. Anonymized transcripts were created with Word, cleaned for intelligent transcription, and inductively coded in Nvivo by two independent coders. Selected themes were pertinent to designing new research data services from the Library.

Results: Analysis of the interviews identified several opportunities for the Library to take ownership of research data services, including assisting with Data Management Plans, instruction on data cleaning, and education about sources for data re-use and sharing. Additionally, interviews highlighted confusion about existing campus services and a desire for centralized guidance and support. This project also facilitated collaboration with other campus units desiring to better understand researcher needs and enhance their own support, including Research Information Technology and local Clinical and Translational Science Institute (CTSI), among others. The data interviews and related conversations have spurred data services conversations across campus.

Conclusions: Conversations continue with [Institution] stakeholders (including the Library) about the best way to meet the needs of researchers on campus as well as centralize and coordinate existing endeavors. Partners in these efforts include the Office of Research, Information Technology Services, [Statistical Support Unit], and various College and Department level units. Select interview findings are also set to be included in the research support section of a campus-wide strategic plan.

Progressing from Diversity, Equity, and Inclusion (DEI) Dialogs to Inclusive Actions: Preliminary Assessment of the Medical Library Association (MLA) Reads Book Club

Track: Professionalism & Leadership

Don P. Jason, III - Health Informationist / Associate Senior Librarian, University of Cincinnati Libraries / Donald C. Harrison Health Sciences Library, Cincinnati, Ohio

Kelsa Bartley, AHIP - Education & Outreach Librarian / Learning, Research, and Clinical Information Services, Louis Calder Memorial Library / University of Miami Miller School of Medicine, Miami, Florida

Melissa Desantis, AHIP - Director, Strauss Health Sciences Library / Univ of Colorado Anschutz Medical Campus, Aurora, Colorado

Ryan Harris, AHIP - Associate Dean for Public Services, UNC Charlotte / J. Murrey Atkins Library, Charlotte, North Carolina

Shannon D. Jones, AHIP, FMLA - Director of Libraries & Professor, Medical University of South Carolina, Charleston, South Carolina

Tamara M. Nelson, AHIP - User Services Coordinator / Senior Research & Learning Services Librarian, The University of Tennessee Health Science Center Health Sciences Library, Memphis, Tennessee

Virginia (Ginny) Pannabecker, AHIP - Assistant Dean and Director, Research Collaboration and Engagement, Virginia Tech, Blacksburg, Virginia

Dede Rios, AHIP - Director of Optometric & Clinical Library Services, UIW Libraries / Rosenberg School of Optometry, San Antonio, Texas

Objectives: Librarians have been interested in ways to increase actions that would make librarians and librarianship more inclusive and diverse. The Medical Library Association’s (MLA) Reads Virtual Book Club is a book discussion group that explores topics pertaining to Diversity, Equity, and Inclusion (DEI). The 2022 club was framed around Isabel Wilkerson's Caste: The Origins of our Discontents. Participants completed an optional survey at the conclusion of the program. The survey answered the research question “Will participating in a DEI book discussion group for six months translate into more willingness to adopt inclusive behaviors in the workplace within the next year?”

Methods: The electronic survey used Likert Scales to measure the impact of (and satisfaction with) the Medical Library Association (MLA) Reads Book Club. The electronic survey was created in Microsoft Forms. The survey received a “Not Human Subjects” determination from an Institutional Review Board (IRB). The survey was deployed to 200 book club participants consisting of librarians, medical professionals, and diversity enthusiasts. These participants are based around the United States. Participants’ names and other identifiers were not collected. The survey was voluntary. Participation in the survey had no effect on participants’ ability to earn Continuing Education (CE) Credit or on their career trajectories. The survey started with a set of behavioral questions that asked participants to anticipate how the book club might affect their actions. The second section focused on attitudes toward Diversity, Equity, and Inclusion (DEI). Respondents were presented with several DEI prompts/scenarios, and they rated their level of comfort with the scenario. Next, there were several free response reflection questions. These questions allowed participants to share lessons learned, comments, and suggestions. The survey concluded with a set of demographic questions. Survey responses were collected during a three-week window at the conclusion of the current cycle of MLA Reads.

Results: In total, 70 participants completed the mixed methods survey. Respondents were given 10 behavior prompts and 10 attitude prompts measured with quantitative Likert scales. The survey also asked qualitative open response questions. Responses to questions were overwhelmingly positive, with respondents eager to engage in 7 of the 10 inclusive behaviors. However, 3 out of the 10 behaviors had more neutral responses. This trend continued with 10 attitude questions, which measured comfort level in various DEI scenarios. Respondents were comfortable in 7 of the 10 scenarios, and neutral or uncomfortable in 3 of the scenarios. Open response questions provided candid testimonials.

Conclusions: Respondents' eagerness to engage in 70% of the inclusive behaviors and their comfort level in 70% of the DEI scenarios, is a noticeable trend. We conclude that participation in a DEI book discussion group had a positive impact on MLA Read participants. The 3 behaviors that respondents gave the most neutral ratings include joining a committee that advocates for DEI causes, seeking volunteer opportunities on campus to further DEI causes, and starting a DEI book discussion group at their workplace. Coincidentally, these are also the 3 most time-consuming behaviors. Further study is needed to identify the reason behind the responses. In the future, a pretest and posttest model should be used to track behavior and attitude change during MLA Reads.

The Role of Health Sciences Libraries in Supporting Consent Standards for Medical Image Publication

Track: Information Management | On Demand

Sarah McClung - Head of Collection Development, University of California, San Francisco Library, San Francisco, California

Rachel Keiko Stark, AHIP - Health Sciences Librarian, California State University, Sacramento Library, Research and Instruction, Sacramento, California

Megan DeArmond - Systematic Review Services Librarian, Touro University Nevada, Jay Sexter Library, Henderson, Nevada

Objectives: The objectives of this research are to: 1.) assess health sciences library workers' current knowledge of best practices and recommendations for informed consent for the publication of medical images and 2.) determine if knowledge of these standards informs the professional practice of health sciences library workers.

Methods: The authors developed a survey to measure the familiarity of health sciences library workers with established informed consent standards for the publication of medical images. The survey will be distributed in December 2022, primarily through listservs, and current health sciences library workers in the United States will be invited to complete the survey. For those survey respondents already aware of these best practices and recommendations, the authors will ask how their professional practice is informed by this knowledge, if at all. For those respondents not aware of these best practices and recommendations, the authors will ask how they anticipate their professional practice being changed, if at all, now that they are aware. Demographic questions will also be asked. Primarily quantitative analysis will be utilized to evaluate the response data. Open-ended survey responses will require additional qualitative analysis.

Results: Results will be provided during the presentation.

Conclusions: This paper will establish a baseline from which further discussion and research can emerge regarding health sciences library workers’ roles, as resource purchasers and educators, in supporting best practices and recommendations for informed consent for the publication of medical images. With the strengthening of open science initiatives and advancement of computational analysis of large datasets, there is an increased need for resource creator accountability. History features many examples of unethical medical acts that disproportionately affected and continue to affect marginalized individuals. Awareness of how technology is radically changing the meaning of informed consent in the twenty-first century is an imperative first step towards improving professional practice and decreasing the likelihood that librarians are contributing to unethical behavior.

The Role of Libraries and Librarians at the Intersection of Public Health and the Arts

Track: Information Services | On Demand

Karin J. Saric - Medical Librarian, Norris Medical Library, Los Angeles, California

Objectives: This paper explores the role of libraries and librarians at the intersection of public health and the arts, within the framework of Creative Placemaking (CP). CP is a community development design structure

that makes intentional use of local places, and can leverage the power of the arts to connect people, expose root issues, center underrepresented voices and concerns, and shift sociocultural norms and collective behaviors. Over the last few decades CP has been widely integrated throughout the healthcare environment, including creation of evidence-based findings that support the benefits of incorporating such a framework into public health work.

Methods: Using historiography as a research method, the author formulated a series of questions that focused on gaining understanding of the underlying theories, frameworks, and evolution of programming within the sectors of Creative Placemaking, public health, knowledge translation, the arts in healthcare, and libraries as place over the last few decades. Literature, reports, online documentation, and websites were reviewed and analyzed to identify themes across the various focus areas, implementation of successful programming, and to identify areas where libraries and librarians can make future contributions. A resource list was created that includes links to key groups, toolkits, frameworks, and relevant literature.

Results: Themes that emerged include focusing on community level behavioral change, and formalizing work at institutional levels in order to scale to broader environments. Research evidence supports the ability of arts-based programming to engage populations by transcending barriers such as language, traditions, and belief systems. While performing & visual arts, and interactive activities were found to impact behavior, the most relevant factor that induced change was active involvement in the creation of art. As libraries are trusted, networked, centrally located public spaces, they are natural partners in the effort to provide access to arts-based programming that focuses on health behavior change.

Conclusions: Public health has moved into a fifth wave that focuses on creating a community level "culture of health." Using the CP framework, libraries can impact community level health behavior in two ways. Locally, they can increase dissemination of reliable health information, that supports behavior change, by formally incorporating evidence-based arts programming within collaborative learning environments. At the institutional level, collaborative, capacity building efforts can leverage nationwide networks to incorporate consistent, robust, evidence-based, arts programming within community-based health models, such as that of social prescribing. The main challenge within libraries is the lack of staff to coordinate and run such robust programming. Libraries should hire community engagement librarians to address the scope of the work required to succeed in such endeavors.

A Scoping Review on Diversity, Equity, and Inclusion Initiatives for Health and Medical Library Workers

Track: Professionalism & Leadership

Jane Morgan-Daniel, MLIS, MA, AHIP - Community Engagement and Health Literacy Librarian, University of Florida Health Science Center Libraries, Gainesville, Florida

Amy Taylor, AHIP - Medical Librarian, Houston Methodist Education Institute, Houston Methodist Hospital, Houston, Texas

Xan Goodman, AHIP - Health Sciences Librarian, UNLV University Libraries, Henderson, Nevada

Chloe Hough, AHIP - Reference Librarian, Tampa Bay Regional Campus Library, Florida

Objectives: Four librarians conducted a scoping review on diversity, equity, and inclusion (DEI) initiatives for workers in health and medical libraries. The review's objective was to map out how these DEI initiatives vary in terms of 1) how DEI is defined; 2) their focus, such as policies, programming, recruitment/retention, services, or space; 3) their stated impact; 4) how libraries advocate for involvement in DEI and the associated challenges they face; and 5) recommendations for practice or research gaps. The review encompasses the years 2014 onwards due to the globally emerging Black Lives Matter movement, which

catalyzed awareness around DEI.

Methods: The project was guided by the Joanna Briggs Institute's Framework for Scoping Reviews, as well as PRISMA Sc-R. A protocol was registered in the Open Science Framework in September 2021. The following databases were searched for a total of 7,360 results: ERIC, Library Literature & Information Science Full Text; LISTA, Library and Information Science Source, Library Science Database, PubMed, Scopus, and Web of Science. De-duplication and screening took place in Covidence. After pilot screening, four reviewers independently carried out title and abstract screening of the 5622 results, with two reviewers per article. Full-text screening used the same process and screening disagreements were resolved through discussion. Twenty-eight articles progressed to the data extraction stage, where the pre-populated data extraction forms were filled out by two team members independently. Data extraction is still in process; once complete, the results will be analyzed thematically and summarized narratively.

Results: The review is still in progress, but it is anticipated that the results will encompass a map of the types of DEI initiatives conducted for workers in health science libraries over the past seven years in different countries, as well as levels of impact, the challenges and successes involved, and strategies for justifying and advocating for library involvement in DEI.

Conclusions: Once the review is complete, the co-authors will be able to provide evidence-based recommendations for other libraries who would like to create DEI professional development opportunities for their employees.

Scoping the Systematic Review Landscape: Service Level Fluctuations at Health Sciences Libraries

Track: Information Services

Annie M. Thompson - Head, USC Norris Medical Library, Los Angeles, California

Karin J. Saric - Medical Librarian, Norris Medical Library, Los Angeles, California

Hannah Schilperoort, MLIS, MA - Head, USC Wilson Dental Library, Los Angeles, California

Objectives: In 2020, our library paused our still-in-demand, full level systematic review service due to limited bandwidth. There was a perception that providing 1-hour consultations meant we were falling behind our peer institutions. Upon reflection, we did not have an accurate idea of what other institutions offered. This paper will summarize survey data from AAHSL Full Member libraries in the United States to analyze fluctuations in service level due to resource availability. Libraries can use this information to gauge where they stand in comparison to the overall service landscape.

Methods: The authors will distribute a survey to AAHSL Full Member library leaders in the United States to gain insight about systematic review service levels and fluctuations in service provision from 2020. The survey will include questions that gauge current level of service provision ranging from; basic resource support (libguide, software), education, consultations, to full participation, including authorship and fees. Fluctuation in service questions will consider factors such as; departmental and institutional reorganizations, librarian attrition, lack of knowledge, and budget cuts. The survey will be reviewed by the Institutional Review Board.

Results: Results will be included later.

Conclusions: Conclusions will be included later.

Search Filters Designed to Identify Black Persons Living in the United States

Track: Innovation & Research Practice

Robin A. Paynter - Research Librarian, Scientific Resource Center, Effective Health Care Program, Agency for Healthcare Research and Quality, Portland, Oregon

Michele A. Datko - Senior Medical Research Librarian, ECRI Information Center, Evidence-based Practice Center, Plymouth Meeting, Pennsylvania

Melinda C. Davies - Associate Research Informationist, Kaiser Permanente Center for Health Research Evidence-based Practice Center, Portland, Oregon

Shannon S. Glover, AHIP - Health Sciences Library Coordinator, Northeast Georgia Health System, Gainesville, Georgia

Irene M. Lubker, AHIP - Medical University of South Carolina, Charleston, South Carolina

Dorice L. Vieira - Clinical Outreach & Graduate Medical Education Librarian, NYU Health Sciences Library, New York, New York

Christiane Voisin - Research Specialist, Department of Internal Medicine, Ohio State University College of Medicine / Research Triangle Institute-University of North Carolina Evidence-based Practice Center

Objectives: Increasingly searchers need to limit their searches to specific populations. This has traditionally been difficult due to the existence of a wide array of synonyms and changes in terms over time. This is especially evident in the terms associated with Black Persons Living in the United States (BPLiUS), i.e., US-born Black Americans and US immigrants of African or Caribbean ancestry. To increase the efficiency, reliability, and inclusivity of population searches we will create and validate search filters for biomedical and health sciences-related information on BPLiUS populations in Ovid MEDLINE and PubMed.

Methods: We will use a handsearch method to create our gold standard citation set. To gather the most representative citation sample we will draw from Black-focused journals along with journals in the following categories: race/ethnicity-focused, high impact/large audience general medical journals, clinical/health systems, anthropological/cultural/psychosocial/genetics, and lastly regional/state medical society journals published in areas with large Black populations. To capture the breadth and depth of explicit and implicit Black ethnonym terms over time we will sample citations in the above categories in twenty year increments, i.e., 1955 (Jim Crow era); 1975 (Civil Rights era); 1995 (African-American era); 2015 (People of Color era); and 2021 (BIPOC era) to capture more recent language. To identify subject terms, keyword terms and phrases, we will use the following methods: text-mine Black-focused journal citations using the AntConc software; consult the National Library of Medicine MeSH database; identify extant example search filters; review published systematic review search strategies, etc. The gold standard set will be divided into test and validation sets. The filters will be internally and externally validated. We will predetermine target sensitivity/precision.

Results: We will report on our novel search filter methodology and progress to date on testing our filters.

Staying Current In-Patient Care: How Physicians in Medically Underserved Areas Gain Knowledge for Health Care Decision-Making

Track: Clinical Support

Irene M. Lubker, AHIP - Medical University of South Carolina, Charleston, South Carolina

Objectives: The purpose of this study was to explore the ways primary care physicians who practice medicine in medically underserved areas (MUA/Ps) maintain knowledge for patient care and decision making.

Methods: A qualitative research study utilizing semi-structured interviews and physician observations of ten diverse primary care physicians who work in medically underserved areas in one southern state was analyzed to describe their experiences in obtaining information for patient care and decision making. Participation was limited to primary care physicians who have worked in health centers designated as MUA/Ps for at least five years. The physicians who participated in this research were selected via convenience sampling. An inductive process in ATLAS.ti Web was used to determine common themes.

Results: The findings of the study revealed that despite some barriers to information seeking such as time constraints, access issues, database complications, and inadequate information seeking skills, physicians in MUA/Ps seek information to aid in practicing evidence-based patient care when possible. The study also found that these physicians are mostly self-directed in their learning to maintain knowledge for patient care, and are mostly motivated by patient safety, proliferation of biomedical literature, need to be lifelong learners and their status as patient educators.

Conclusions: Overall, the knowledge gained from this study can be used to increase support by health sciences librarians and public health networks for physicians who practice in MUA/Ps. Some of the barriers can be alleviated through partnerships and collaborations with health sciences librarians, finding opportunities for instruction including making physicians aware of open access biomedical literature. Limitations: The findings are limited by the small sample size of convenience, the study design which relied heavily on self-reports and perceptions, and the setting. Implications and recommendations for practice, research, medical education, and health sciences librarianship will be discussed.

Subjected: Investigating the Impact of MeSH Terms on Underrepresented Groups

Track: Information Management

Lorin K. Jackson - Executive Director, Region 2 Regional Medical Library, Medical University of South Carolina, Network of the National Library of Medicine, Charleston, South Carolina

Jamia Williams - Consumer Health Program Specialist, University of Utah/The Network of the National Library of Medicine/Training Office, Rochester, New York

Kelleen Maluski, MLS - Student Success and Engagement Librarian, Health Sciences Library & Informatics Center, University of New Mexico

Alexis Ellsworth-Kopkowski

In this research abstract, our team outlines the project's background, provides the methodology, and encourages participation. The project aims to investigate the impact of referencing Medical Subject Headings (MeSH) for library workers who utilize MeSH terms in their instruction. Historically, many MeSH terms have not been inclusive or trauma-informed. Continued unfettered use of these terms can (re)traumatize those who must reference them in their work as library personnel. Previous research about MeSH terms is limited. In our collaborative research, we seek to center the stories of librarians and users gained through conducting focus groups to draw preliminary conclusions. We hope our findings also clarify future research recommendations. Overall, the primary goal of this research study is to amplify the

narratives of many Indigenous, Hispanic, Latino/a, Latinx, Black, People of Color, people with disabilities, and LGBTQIA+ library workers in an intentional, analytical way. In addition, this research supports an effort to continually motivate our field to improve the categorization and definition of MeSH terms.

Systematic Searching for the Social Determinants of Health

Track: Information Services

Rosie Hanneke, MLS, AHIP - Associate Professor & Head, Information Services and Research, Library of the Health Sciences, University of Illinois Chicago, Chicago, Illinois

Amelia Brunskill, MSIS - Assistant Professor & Information Services and Liaison Librarian, University of Illinois Chicago, Chicago, Illinois

Objectives: The social determinants of health (SDOH) are the focus of an exponentially increasing number of publications across health sciences disciplines, including evidence syntheses. However, there does not yet exist a validated search hedge for the concept of SDOH. Consequently, expert searchers must start from scratch in building their own searches for this sprawling concept. This study seeks to examine published SDOH searches to address the following: What terms do authors use to search for SDOH literature and how do they justify their approaches? What can we learn from the papers cited in these reviews and their indexing? How might this knowledge inform best practices for systematic searching of the SDOH literature?

Methods: A search was conducted in PubMed, Embase, and Scopus for systematic reviews, scoping reviews, and other evidence syntheses which 1) explicitly mentioned the SDOH in their central research questions and 2) included a full SDOH search strategy for at least one database. The search strategies of these reviews were extracted and analyzed using Microsoft Excel and Access. Relevant data extracted from each review included databases searched; search terms used for the SDOH, including keywords and controlled vocabulary; and conceptual frameworks cited by review authors as a source of search terms. Authors' self-described methods, including individual keyword selection as well as their overall approaches to designing search strategies, were analyzed. Primary studies cited in the published reviews were collected in a set to investigate how these papers were indexed and compare them to the relative recall of the SDOH search strategies and relevant MeSH terms.

Results: 64 published search strategies were identified and included in the analysis. The set of cited papers included 2518 PMIDs. There were few commonalities across search methods. Authors used a total of 204 unique MeSH terms and 1,384 keywords or phrases to search for the SDOH concept. The frameworks used by the most reviews to define the SDOH concept were those from the World Health Organization and HealthyPeople 2020/2030. Among the 2518 cited papers, less than 3% were indexed with the "Social Determinants of Health" MeSH term. The most frequently appearing MeSH, "Risk Factors," was not used in any of the search strategies we analyzed.

Conclusions: There is no single agreed-upon definition for the SDOH in these reviews; it is a sprawling concept that can be defined in hundreds of ways. We cannot rely on MeSH to retrieve the SDOH literature. Our findings point to the usefulness of using one or more conceptual frameworks to guide term selection, as well as using both specific and non-specific search terms.

Technical Services Librarians in the Medical Library Association

Track: Information Management

Toni Hoberecht, AHIP - Technical Services Librarian, Schusterman Library at OU-Tulsa, Tulsa, Oklahoma

Emrys Moreau - Reference and Instruction Librarian, Schusterman Library at OU-Tulsa, Tulsa, Oklahoma

Objectives: Some technical services librarians do not participate in MLA because of a perceived lack of support from the organization. This study’s chief objectives are to investigate technical services librarians' MLA experience and discover what could be done to improve that experience. Using the Perceived Organizational Support framework, this project will address the following research questions:
What perceptions do technical services librarians in health sciences libraries have regarding MLA support?
What barriers do technical services librarians face in their MLA participation?
What do technical services librarians feel either they or MLA could do to reduce those barriers?

Methods: In August 2022, a survey was sent to listservs likely to be read by technical services librarians who work or have worked in health sciences: MLA’s Technical Services and Collection Development Caucus listservs, ACRL Health Sciences Group and Technical Services Interest Group listservs, and the AAHSL Directors listserv. The survey recorded attitudes that technical service librarians have about their participation in MLA using validated questions from the Perceived Organizational Support framework. The survey collected information on possible barriers to participation and solicited information on how perceptions might be improved. Correlations will be measured between Perceived Organizational Support variables and reported level of MLA participation as well as years in librarianship and perceived levels of support. Correlations will also be measured between Perceived Organizational Support variables and barriers encountered. Interviewees were recruited through the survey to discuss personal experiences regarding the study’s research questions. Nine interviews were conducted. Interview transcripts will be coded to develop themes relating to the research topics, with the intent to illuminate specific perceptions and barriers experienced by technical services librarians.

Results: There were 74 responses to the survey. Four respondents did not answer the inclusion criteria questions and were excluded. Of the 70 valid responses, 72.5% (n=50) of respondents are or had been members of MLA. Among individuals who had been or are currently MLA members, there was a moderate positive correlation between the question of whether MLA values their contribution and self-perceived perception of overall participation in MLA, $r(27) = .43$, $p = .021$. There was a strong negative correlation between the created scale variable for positive opinions about volunteering for the MLA and the number of years worked as a technical services librarian, $r(9) = -.82$, $p = .002$. There was a moderate negative correlation between the question of how well respondents felt MLA supports technical services librarians and their number of years worked as a technical services librarian, $r(42) = -.46$, $p = .002$. The most frequent barrier identified for lack of participation or not joining MLA was feeling a lack of content relating to their work concerns and interests (38.6%, n=27).

Conclusions: The qualitative analysis of textual responses to open-ended survey questions is ongoing. Common themes have included the perception that MLA does not offer enough applicable programming, a desire for an increase in relevant resources, and a need for support for technical services staff.

The coding of interview transcripts and qualitative analysis is in progress. Complete results will be presented at the meeting.

"You Need to See It": Attitudes and Preferences of Health Sciences Librarians Regarding Assessment of Evidence-Based Medicine via an Objective Structured Clinical Examination

Track: Education

Caitlin Plovnick - Lead, Education & Curriculum Integration, NYU Health Sciences Library

Joey Nicholson - Chair and Director, NYU Health Sciences Library, NYU Langone Health

Objectives: While the ability of trainees to demonstrate competence in Evidence Based Medicine (EBM) is an expectation, concerns have surfaced regarding medical schools’ ability to provide comprehensive assessment. Previous efforts to improve this led to the development of a tool and process to assess EBM behaviors via observation in an Objective Structured Clinical Examination (OSCE). The next step in this process is evaluating its feasibility for wider adoption across institutions. The purpose of this study is to understand and document the experiences and preferences of health sciences librarians regarding use of OSCEs in assessing medical students’ EBM behaviors.

Methods: We used a grounded theory approach to explore librarians’ perceptions of assessing competence of medical students in practicing evidence-based medicine using an OSCE. Seventeen librarians who had participated in such assessment were invited to participate in focus groups about their experiences. All participants had received training on the background and use of the OSCE assessment form and process, were provided samples to assess in order to calibrate, and then each participated in assessing medical students as a primary or secondary assessor. During the focus groups, the interviewer asked open-ended questions, inviting participants to describe their roles in teaching and assessing EBM at their home institutions, gaps and opportunities they see in EBM assessment, and thoughts on the potential for expanding the use of the OSCE. All interviews were audio recorded and transcribed verbatim for analysis. The authors conducted a round of initial coding to surface main themes, which were used to develop a formal coding structure. A second round of focused coding using this structure was completed, using the Dedoose Desktop App to store and code the transcripts.

Results: Analysis revealed high variability in existing EBM assessment activities and the role of the library across institutions, with many common challenges. Time constraints, limited staffing, and the need for administrative support and faculty partnerships were cited as barriers to successful, scalable EBM instruction and assessment. Benefits of the OSCE in addressing these barriers were explored, including the impact of results on student attitudes and faculty awareness regarding EBM skills. Benefits and challenges of implementation were also addressed, and specific feedback was collected on how to improve the experience and implementation of the OSCE.

Conclusions: Wider adoption of the EBM OSCE across institutions is feasible and would be welcomed by health sciences librarians, but buy-in and support from administration is needed to make this happen. Evidence of student level of ability from existing implementations of the OSCE may be helpful in attaining buy-in and securing more time for libraries within curricula. Cross-institution collaborations to support a standard for assessing EBM behaviors will also improve the reliability of using this structured rubric.

Whole-Task Assignment for Pre-Clinical Students: Assessing EBM Skills before Clerkships

Track: Education | On Demand

Juliana Magro - Education and Research Librarian, NYU Health Sciences Library, New York, New York

Joey Nicholson - Chair and Director, NYU Health Sciences Library, NYU Langone Health
Caitlin Plovnick - Lead, Education & Curriculum Integration, NYU Health Sciences Library

Objectives: As medical students develop clinical expertise and begin to provide direct patient care, it is important that they are able to apply Evidence Based Medicine (EBM) principles to practice. Whole-task EBM assignments have been associated with improved learning and application of EBM skills, but tend to be applied in the clerkships and beyond. The aim of this study is to determine effectiveness of course-integrated EBM instruction as evidenced by student performance on a capstone whole-task assignment at the end of a six-part EBM course series embedded in the first-year, pre-clinical curriculum.

Methods: At the end of a series of seven EBM-focused sessions embedded in a first-year Practice of Medicine course, students were asked to complete a whole-task assignment. This assignment requires demonstration of EBM skills that had been covered in the course, including: formulating a question based on a patient scenario; documenting their database searches when attempting to acquire evidence; selecting an article to answer their question; appraising the article; and discussing how they would (or would not) apply that evidence to the patient in the scenario. They were also asked to reflect on their process and assess their own skills. Students submitted their completed assignments via the School’s learning management system, where two librarians reviewed them and provided structured individual feedback using a rubric adapted from the Fresno test. The librarians analyzed the complete set of assignment results to identify patterns and provide a holistic snapshot of student performance.

Results: 95 first-year students completed the assignment, out of 111. A preliminary assessment of their overall performance showed 15% of students scoring below expectations, 56% meeting expectations, and 29% exceeding expectations. Mechanical errors in basic question formulation and syntax for database searching were revealed as common challenges, while selection and appraisal of evidence provided insight into students’ strengths and gaps in understanding. 88% of students chose a recent systematic review; 70% selected what the librarians determined as best evidence available on the topic. Analysis is ongoing. Further details regarding performance on distinct EBM steps will be included in the final presentation.

Conclusions: Results from the whole task assignment provided a useful demonstration of students’ abilities at the end of the first-year EBM course. This provided librarians with insights into areas of improvement for future iterations of the course and enabled us to provide constructive feedback to students to guide their continued growth. This information can also be used to enhance communication with faculty, increasing awareness of students’ level of mastery and informing the continuity of EBM content into Clerkships and beyond. Future studies will connect this whole-task assessment framework to other EBM assessments to track student performance and provide feedback on EBM skills throughout their education.

PAPER PRESENTATIONS: PROGRAM DESCRIPTION ABSTRACTS

We have sorted paper presentation abstracts in this section by title in alphabetical order and note presentations available to conference attendees on-demand.

Activating Public Health Library in the Midst of a Pandemic: Experience from Nigeria Centre for Disease Control

Track: Health Equity & Global Health | On Demand

Chinenye Benjamin - Senior Knowledge Management Officer, Nigeria Centre for Disease Control, Abuja, Federal Capital Territory, Nigeria

Biliamin Oladele Popoola, n/a - Systems, Scholarly Communications and EBM Librarian, University of Medical Sciences Library, Ondo City, Ondo, Nigeria

Chinwe Ochu, n/a - Director, Prevention Programmes and Knowledge Management, NCDC, Abuja, Federal Capital Territory, Nigeria

David Olatunji, n/a - Library and Knowledge Management Officer, Nigeria Centre for Disease Control, Federal Capital Territory, Nigeria

Background: At the wake of the COVID-19 pandemic in Nigeria, the Library and Knowledge Management (LKM) unit of the Nigeria Centre for Disease Control (NCDC) was activated to support the pandemic response and other activities of the agency. NCDC is Nigeria’s public health institute saddled with the mandate of protecting the health of Nigerians. This paper highlights the steps taken to establish the public health library, presents an account of the experiences involved in implementing the various activities in the LKM unit, and the challenges in setting up a library amidst a global pandemic.

Description: Establishing the NCDC Library and Knowledge Management (KM) services encompasses the following:

Mentorship for NCDC LKM Focal Persons: This was pedalled on orientation, question and answer sessions, expository discussion, role-playing, job shadowing and coaching through commissioned consultancy with an astute health sciences librarian.

Knowledge Audit: This stage explored the KM awareness of the NCDC staff, existing knowledge-sharing practices and knowledge assets, and the knowledge needs of NCDC staff for public health functions.

Creation of Library and Knowledge Management Strategy and Roadmap: Based on the knowledge audit, the library and KM strategy and roadmap were created to guide LKM services and activities within the agency.

Intra-Action Review (IAR): The LKM unit implemented an IAR within the U.S. Centre for Disease Control and Prevention (CDC)-supported Advancing Capacity for Epidemic Preparedness and Response in Nigeria (ACEPRIN) project. The IAR identified the best practices and lessons learned from NCDC’s COVID-19 response across Nigeria, and serve as an impetus for enhancing the evidence-based public health functions of the NCDC.

Other initiatives: launching a bi-weekly Evidence Brief; library automation; establishing a digital archive for public health response resources; installation of digital information display; and information literacy training for the public health team of NCDC.

Conclusion: Activating the Library and Knowledge Management (LKM) unit of the NCDC is crucial to the pandemic response activities of the NCDC. Libraries can play significant roles in enabling access to evidence and up-to-date knowledge to support and enhance public health functions. Establishing the LKM unit at NCDC highlights the important roles libraries could play in global health. The paper shows that existing knowledge needs among public health workers can be significantly addressed through library and knowledge-related services, thereby highlighting the important functions of library and knowledge management in public health. Recommendations are made on sustaining the effectiveness and impact of library and knowledge management functions in public health environments.

Advocacy Resources for Hospital Librarians -- Hospital Library Caucus LibGuide

Track: Professionalism & Leadership | On Demand

Basia Delawska-Elliott, MLS, AHIP - Medical Librarian, Providence System Library Services, Portland, Oregon

Nancy A. Clark - Director, VHA Library Network Office, Washington, District of Columbia

Sondhaya (Sunny) McGowan, AHIP - Manager, Medical Library, Scripps Mercy Hospital, San Diego, California

Vida M. Vaughn - Director, Kornhauser Health Sciences Library, Louisville, Kentucky

Brian L. Baker - Library Services Program Manager & Literacy Program Coordinator, Medical Library, Valley Children's Healthcare, Madera, California

Kathy Zeblisky, AHIP - Medical Library Manager, Phoenix Children's Hospital, Phoenix, Arizona

David King

Angela Spencer, AHIP - Health Sciences Librarian, Saint Louis University, St. Louis, Missouri

HelenAnn B. Epstein, AHIP, FMLA - Informationist, Virtua Health Sciences Library, Monroe, New Jersey

Michelle Kraft, AHIP, FMLA - Library Director, Cleveland Clinic, Cleveland, Ohio

Jill (Tarabula) Daby, AHIP - Regional Medical Librarian, Northern New York Regional Medical Library, Sebastian, Florida

Priscilla L. Stephenson, AHIP, FMLA - Retired, James A. Haley Veterans Hospital, Tampa, FL, Tampa, Florida

Donna S. Gibson - Director, Library Services, Memorial Sloan Kettering Library, New York, New York

Elizabeth Ten Have

Halyna Liszczynskyj

Caroline Marshall, AHIP - Senior Librarian for Public Services, Cedars-Sinai, Los Angeles, California

Mary Gaudette

Elizabeth Laera, AHIP - Medical Librarian, McMahon-Sibley Medical Library / Brookwood Baptist Health, Birmingham, Alabama

Jess L. Callaway, AHIP - Clinical Research Librarian, Noble Library & Research Center, Atlanta, Georgia

Heidi Sue Adams - Lead Medical Librarian, Logan Health, Kalispell, Montana

Lindsey Gillespie, AHIP - Medical Librarian, Dignity Health, Northern California Division

Stacy F. Posillico - Senior Librarian, Northwell Health Eastern Region Hospitals, Hempstead, New York

Ellen Aaronson, MLS, AHIP - Librarian, Mayo Clinic Libraries, Rochester, Minnesota

Background: Hospital librarians frequently have to justify their role to health system administration. Additionally, often new librarians receive little or no training or onboarding explaining the unique nature of hospital librarianship. Some, working as solo librarians, may not know where to turn for the resources needed to learn the necessary skills. In the fall of 2019, the Professionalism & Leadership Hub reached out to the Hospital Library Caucus (HLC) asking if Caucus leadership was interested in updating the existing Values 2 Toolkit created as a MLA Rising Star project in 2014-2015 by Roy Brown and Helen-Ann Brown Epstein. HLC leadership and members responded enthusiastically and plans for developing a new web-based, scalable, and updatable tool were put into motion.

Description: A call to HLC membership was made, asking Caucus member about the shape and content of the new toolkit and volunteers for the project. Many caucus members shared ideas for content and expressed interest in helping. Ideas about areas of concentration were collected to serve as the basis for the project.. After initial organizing meeting in 2020, volunteers were divided into task forces in charge of main specific areas – Standards of Practice, New Hospital Librarians Resources, Advocacy Toolkit, and Guidance for Healthcare Administrators. Task forces met to discuss member roles and split up responsibilities. Each main area of concentration was divided into smaller topics. MLA was approached for platform suggestions. LibGuides by SpringShare were selected as the platform. After the launch of the project, task forces met on a regular basis to discuss content inclusion, search strategies, and progress. The work to select articles, books, websites, blogs, and other media to be included in the guide in each category was under way. At the same time, a separate effort to work with MLA to develop a Hospital Library Advocacy Statement was launched. The final statement was included as a section of the LibGuide.

Conclusion: The project culminated in launching the Hospital Library Caucus: HLC Advocacy Home LibGuide in August 2022. The new resource included the Hospital Libraries Advocacy Statement released in February 2022, the draft version of the Standards of Practice for Hospital Libraries and Librarians, information for new hospital librarians, and sections on Library as Hub, Dazzling Stakeholders, and Excellence in Librarianship. Since its release, the Hospital Library Advocacy Statement has been signed by 11 organizations; outreach is ongoing. The Hospital Library Advocacy LibGuide has been accessed more than 2,200 times within the first two months of launch. Next steps include putting into place structures that will ensure that the Advocacy Toolkit will continue to be updated with relevant information.

Advocating for the Value of Health Sciences Librarians

Track: Innovation & Research Practice | On Demand

Mark MacEachern, MLIS - Informationist, University of Michigan

Susan Lessick, AHIP, FMLA - Distinguished Librarian Emerita, University of California, Irvine, Anaheim, California

Janene Batten - Senior Research & Education Librarian, Harvey Cushing/John Hay Whitney Medical Library - Yale University, New Haven, Connecticut

Brian A. Conn - Technical Information Specialist, Minneapolis VA Healthcare System, Minneapolis, Minnesota

Jennifer Deberg, OT, MLS - User Services Librarian, University of Iowa, Iowa CIty, Iowa

HelenAnn B. Epstein, AHIP, FMLA - Informationist, Virtua Health Sciences Library, Monroe, New Jersey

Mary Pat P. Harnegie, AHIP - Medical Librarian, Cleveland Clinic, Olmsted Falls, Ohio

Ellen M. Justice - Clinical & Research Librarian, MAHEC, Asheville, North Carolina

Liz Kellermeyer, AHIP - Director, National Jewish Health, Denver, Colorado

Valerie Lookingbill - Sciences Librarian, University of South Carolina, Columbia, South Carolina

Laura Menard - Access Services Department Head, Northern Kentucky University, N/A

Tanisha N. Mills, MLIS, AHIP - Medical Librarian, Northeast Georgia Health System-Gainesville, St. Thomas

Maggie Shawcross, MLIS, MPH, CHES - Teaching and Outreach Librarian, University of Northern Colorado, Greeley, Colorado

Julia C. Stumpff - Instructional Design Librarian, Ruth Lilly Medical Library, Indianapolis, Indiana

Jennifer C. Westrick, AHIP - Library Research Information Specialist, Rush University, Westchester, Illinois

Douglas L. Varner, AHIP - Assistant Dean for Information Management, Georgetown University, Washington, District of Columbia

Andrea Lynch - Scholarly Communication Librarian, Lee Graff Medical & Scientific Library, City of Hope, Duarte, California

Terry Ann Jankowski, AHIP, FMLA - Librarian Emerita, University of Washington, Lake Forest Park, Washington

Background: Since 2016, the Evidence You Can Use To Communicate Library Value page on the Medical Library Association (MLA) website has highlighted studies that demonstrate the tremendous value health sciences librarians bring to their institutions and communities. It serves the profession as a freely accessible resource for library advocacy. This year the MLA/RTI (Research Training Institute) Value Studies Working Group was tasked with the first significant update to the page since it was originally published in 2016.

Description: The goal of the project is to communicate the profession’s value through the selective use of the strongest evidence. The Working Group used several methods to identify the new value studies. They conducted database searches and hand searches of select journals, talked to domain experts, and checked references of key studies. Priority was given to studies published after the original work was done in 2016. After reviewing hundreds (maybe thousands) of studies, the Working Group found important new research that showcases librarians' impact on clinical decisions, patient care, health profession education, evidence syntheses, scholarly research, and institutional bottom lines. The group also sought value studies in domains that were not represented on the original website, such as data management, patient safety, and hospital librarianship. As a collection, these studies show the impact of librarians to be significant and sweeping. This presentation will cover the project's goals, search methods, and challenges, while also summarizing the studies that are highlighted on the site.

Conclusion: The MLA/RTI Value Studies Working Group successfully identified many new studies demonstrating librarians' value to institutions and communities. Since 2016, the number of value studies in the profession has grown significantly, and a selective sample of these studies will be reflected on the updated website.

Building across Silos: Integrating Specialized Data Tools with a Library Data Catalog

Track: Information Management

Nicole Contaxis, MLIS, MA - Data Librarian and Lead of Data Discovery, NYU Health Sciences Library, New York, New York

Background: Researchers require specific tools, software, and infrastructure to meet the technical, legal, and ethical requirements for the analysis, storage, and sharing of their data, particularly data from human subjects research. While using tools designed to meet specific needs allows for specialized support, it also increases the possibility of information silos; researchers are unlikely to know about data stored across the institution that employs solutions for those particular use cases. To address this issue, the library at an academic medical center employs a data catalog to render visible data across the various repositories, services, and platforms available at the institution. This supports an information environment with resources that can be geared towards specific use cases, while preventing information silos.

Description: To leverage the library's data catalog to address data discoverability and access issues at the institution, the library (1) established collaborations with academic and operational departments across the institution, (2) developed in-depth knowledge of tools used at those departments, and (3) developed workflows to connect data analysis and storage tools with the data catalog. Due to the specialized nature of many of these data analysis and storage tools, bespoke workflows are developed to integrate each of these tools with the library’s data catalog. Collaborations with Information Technology (IT), the Shared Scientific Cores, the Office of Science and Research, and a clinical data management support service were established through outreach by data librarians and library leadership. For each collaboration, librarians attended relevant meetings to determine which data analysis and storage tools were suitable candidates for integration with the library’s data catalog. Once a tool was determined to be a suitable tool for integration, librarians create workflows that supported the tools technical specifications and interfaced with the pre-existing workflows for describing data in the library’s data catalog. Each workflow focused on easing researcher tasks like sharing and finding data while bringing together disparate pieces of infrastructure, technology, expertise, and staff.

Conclusion: To determine the success of each integration, data on the number of datasets described, the number of datasets shared, and the number of successful research outputs related to a shared dataset will be tracked. Additionally, the library will compare the rate of growth of the data catalog when this systems-level approach is employed to the rate of growth when datasets were added in a piecemeal fashion. The library will also track secondary benefits to the approach, including the creation of established collaborations across the institution.

“Can You Turn Down the A/C?” Using Free Response Assessments to Gather Medical Student Feedback

Track: Innovation & Research Practice | On Demand

Shalu Gillum, AHIP - Head of Public Services, Harriet F. Ginsburg Health Sciences Library University of Central Florida College of Medicine, Orlando, Florida

Natasha Williams, MLIS, AHIP - User Services Librarian, Harriet F. Ginsburg Health Sciences Library University of Central Florida College of Medicine, Orlando, Florida

Background: Since 2019 the library’s public services team has been utilizing a “free response” interactive assessment featuring large bulletin boards and sticky notes to get candid feedback from medical students. Students post comments in response to several questions. Comments are used to better understand and respond to students’ library needs. After a one-week assessment, the public services team responds to student comments by posting replies on the same boards.

Description: Three bulletin boards are placed in front of the library displaying three open-ended questions: (1) What do you love about the library? (2) What can we do to improve your library experience? (3) What questions do you have for the library? Responses to the questions have helped inform several actions. For

example, multiple comments about the cold temperature led to the library team requesting that building operations increase the temperature in the library space. Comments about noise led to a reiteration of library policies for the commons area of the library space. Questions about specific resources led to better communication to students about those resources. Student comments have also highlighted positive aspects about the library and library team. This assessment has been repeated annually about a month after the start of the new academic year.

Conclusion: The free response boards are a low- to no-cost, fun and interactive assessment that create opportunities for student engagement. The visibility of the questions, comments, and responses from the library let students know that the library team is open to their feedback and responsive to their needs.

The Changes that Lie Ahead: Teaching the History of Dentistry with Rare Materials

Track: Education | On Demand

Nicole Theis-Mahon, AHIP - Liaison Librarian & Collections Coordinator, University of Minnesota, Minneapolis, Minnesota

Lois Hendrickson, MLS - Curator, Wangensteen Historical Library

Ai Miller - Reference Specialist, Health Sciences Library, University of Minnesota, Minnesota

Anna Opryszko - Public Services Supervisor, Wangensteen Historical Library, University of Minnesota

Emily Beck - Assistant Curator, Wangensteen Historical Library of Biology & Medicine

Background: All year one dental students (105) have a two hour history of dentistry class as part of their professionalism course. In 2022 the course director invited the dental librarian and curators of the health sciences rare book collection to develop a new direction for this class. The librarian and curators wanted the class to be an engaging, relevant, active learning session for the students. They created a novel approach where groups of students engaged with historical materials. Class objectives were to understand dentistry’s past, present, and future; juxtapose history and progress; and reflect on the past and one’s place in dentistry.

Description: The dental librarian collaborated with the course director to identify current themes in the dental literature to explore in class, including oral/systemic health; disparities; diversity, equity and inclusion; technology; consumerism; and oral healthcare settings. Curators from the rare books library identified over 100 print materials and artifacts from the late 19th and early 20th century related to the themes. Students were divided into groups of 8-10 and rotated through six themed tables using a World Cafe methodology. Each table had rare materials relevant to that theme and questions for the students to discuss. Questions were provided on large post-it notes and a Google form to provide multiple options for responding. Groups were asked to think about and discuss the questions within the context of the materials and the contemporary theme they were paired with. Thoughts were recorded on the post-it notes or entered into the Google form. An optional Qualtrics survey was distributed at the end of class to gauge their interest in the themes discussed, reflections of working with rare materials, if the class enhanced their understanding of the history of dentistry, and how the themes applied to their education and future as a dental practitioner.

Conclusion: 80% (84/105) students responded to an optional Qualtrics survey. Results showed that the class stimulated students' interest in the history of dentistry, that they learned something new, they were going to share what they learned in the class, and that engaging with historical materials provided a different way of learning history. An open-ended question prompted students to reflect on the session and how it would apply to their education and future as a dental practitioner. Students made connections between the history of dentistry and evidence-based practice. This class presents a model that librarians can use to

actively engage students and learners with historical themes in the health sciences, encouraging students to consider ways of improving future dental practice.

Clinical Support in the Hospital Library: Automating Reference and Patron Management with Customer Service Software Tools

Track: Clinical Support | On Demand

Virginia Trow - Program Manager, Hunter-Rice Health Science Library, Ithaca, New York

Background: A regional hospital library with shrinking resources needed to convert to a digital platform and determine how to maintain and grow remotely its strategically valuable clinical support service.

Description: The library created a new service concept leveraging customer relationship management (CRM) functionality combined with a helpdesk ticketing system model. Using a cost-effective commercial customer service product encompassing contact database, online ticketing system, and multichannel communication features, the library was able to automate staff workflow tasks, develop a deep understanding of the patron base, track patron information needs and behaviors, and leverage multiple communication channels, within a single online interface.

Conclusion: The new clinical support helpdesk provides access to detailed patron and service data and offers very intelligent reporting for healthcare departments and hospital management. The success of this service project should encourage library managers to think about integrating standard business software products into their libraries in order to offer higher-quality features at lower cost compared to purpose-built "library-only" tools.

Customizing Faculty Success to Showcase Librarian Work in an Academic Health Sciences Library

Track: Professionalism & Leadership | On Demand

Lisa M. Acuff, AHIP - Education and Research Librarian, University of New Mexico, Health Sciences Library and Informatics Center

David Lucero - Unit Administrator 3, University of New Mexico, Health Sciences Library and Informatics Center, Albuquerque, New Mexico

Jonathan Pringle - Scholarly Communications and Digital Librarian, University of New Mexico, Health Sciences Library and Informatics Center, Albuquerque, New Mexico

Melissa L. Rethlefsen, MSLS, AHIP - Executive Director & Professor, University of New Mexico, Health Sciences Library and Informatics Center

Background: In October 2019, the University of New Mexico’s School of Medicine (SOM) launched Digital Measures, now known as Faculty Success. The platform’s primary focus was to collect, store, and report faculty activity data in order to streamline promotion and tenure processes. The program was then expanded to other health sciences colleges to create a new campus online directory. The original program was built for the SOM, and faculty at the Health Sciences Library and Informatics Center (HSLIC) experienced challenges with many data fields and reports. The existing options did not adequately represent library professionals and their work.

Description: Faculty Success did not accommodate many activities critical to the diverse types of

librarianship. Therefore, HSLIC created an ad-hoc committee comprised of three library faculty and one staff member to address these concerns. Following an environmental scan of library professional CVs and Academy of Health Information Professional (AHIP) categories, the group removed irrelevant and redundant fields, defined current fields as they relate to library activities, created new fields for library activities not yet represented, and developed a custom CV template tailored to highlight what mattered most to the library. Throughout the summer of 2022, the committee shared their work with other library faculty, incorporating feedback until all activities were represented, and faculty consensus was reached.

Conclusion: Representing library instruction was a key, problematic area. Available fields did not convey the types of instruction developed and shared by library faculty. Another challenge was demonstrating archival work, collection development, and large-scale projects because existing categories did not showcase faculty’s activities in these areas. The new system will be vital to the library’s promotion and tenure process, in the creation of annual reports, and for annual faculty evaluations. Next steps include testing, creating user instructions, and self-entering CV data, a large undertaking, by June 2023. The library hopes the changes and templates will benefit other health sciences librarians using Faculty Success.

Combining Conceptual and Practical Intensive Learning - An Innovative Approach to Professional Development with the Data Services Continuing Professional Education (DSCPE)

Track: Education | On Demand

Ashley Thomas - Digital Initiatives and Accessibility Librarian, Countway Library of Medicine, Harvard Medical School, Boston, Massachusetts

Zhan Hu - Ph.D. Candidate, School of Library and Information Science, Simmons University, Boston, Massachusetts

Rong Tang, Ph.D. - Professor, School of Library and Information Science, Simmons University, Boston, Massachusetts

Elaine Martin - Director & Chief Administrative Officer, Countway Library of Medicine, Harvard Medical School, Boston, Massachusetts

Background: In response to the forthcoming 2023 NIH Data Management and Sharing Policy, the Data Services Continuing Professional Education (DSCPE) program is an eight-week online intensive learning experience geared toward preparing and retooling early to mid-career librarians to provide data services and build a community of practice for research data management. Following an orientation and live session on leadership and collaboration, the DSCPE enhances the learning units of the Research Data Management Librarian Academy (RDMLA) with a 70-hour capstone field experience. The RDMLA is a free online professional development program for librarians and information professionals with a curriculum focused on the essential knowledge and skills needed to collaborate effectively with researchers on data management.

Description: The DSCPE includes an orientation, collaboration and leadership seminar, self-paced RDMLA units, and capstone field project with institutions that have established data service programs. The virtual Collaboration and Leadership session introduces critical concepts and models to help students build foundational knowledge. The class features panels and guest lectures focusing on leading and building partnerships to provide successful data services. The learning component has students work through eight RDMLA units, introducing them to essential RDM concepts and tools. Each unit contains lectures, knowledge checks, and other interactive activities. To fully engage in peer-learning, we encourage learners to post on discussion boards and leave comments. Instructors from the units hold short presentations expanding on important topics, discussing updated content, and answering student questions. The capstone is the culminating practical experience for DSCPE students. During their capstone, students work with a

data services librarian or team at a partner institution on a data project. Examples of pilot cohort projects include: preparing educational and outreach material related to the new NIH DMSP, and creating communication strategies for institutions’ DMS policies. The student keeps a journal, documenting their tasks and experiences. At the end of the capstone, students summarize and present their reflections and takeaways.

Conclusion: The DSCPE fills a gap in research data management and data services professional development. The existing workforce needs to be retooled with hands-on experience in order to support research communities. The DSCPE is an innovative initiative in data training – combining conceptual and experience-based learning, while being cognizant of librarians’ needs and working schedule. The promotion of the capstone, in pairing early-career librarians with established data teams, fosters an immediate community of practice between student, mentor, and cohort. Through a gift from Elsevier to cover student tuition, access to this training is not barred by the burden of cost. The delivery of DSCPE enables equitable learning and empowers individual students to take command of their own education and professional growth.

Completing a Major Library Renovation in a Global Pandemic

Track: Professionalism & Leadership | On Demand

Katie A. Prentice, AHIP - Executive Director, TMC Library, Houston, Texas

Background: Learn the timeline, activities, and results of the complete renovation of the Texas Medical Center Library (TMC Library) in Houston, Texas. The TMC Library is located in an older, but not historic, building from the 1950s (expanded in the 1970s) that has not previously undergone major renovation. This paper will also share the adventures, successes, and challenges of a major library renovation during the Covid-19 pandemic, including the additional leadership and management issues due to renovating during a pandemic.

Description: The complete renovation of the library from studs to furniture was completed between 2019-2021. The new library was launched in 2021 to very low use due to few on-campus classes and a locally high Covid-19 case count. The growth in use from 2021-2022 will be shared, including marketing and outreach efforts about library spaces. Post-renovation updates and continuous improvement plans will also be described. As the library restored standard services in 2021 and students returned to regular use, markerboard question prompts and QR code surveys were utilized to obtain direct user feedback.

Conclusion: Completing a major renovation is expensive and challenging. The added challenge of launching the new space was compounded by pandemic related online courses and very few students or faculty being physically around. The combination of construction, pandemic, and student satisfaction was difficult at times, but the complete results set the library on a positive path for the future. Data from the first year of use show steadily increasing use of TMC Library spaces.

Coral Calcium, Coconut Oil and Corgis: Leveraging the Hospital Library to Combat Health Misinformation in the Clinical Setting

Track: Clinical Support | On Demand

Melissa Spangenberg - Lead Librarian, Kaiser Permanente, West Sacramento, California

Sian E. Aynsley - Lead Librarian, Kaiser Permanente, San Jose, California

Background: In an era of information overload, which the World Health Organization has termed an ‘infodemic’, many people are being exposed to health misinformation: medical information that is false, inaccurate, or misleading. This is causing people to make decisions that can have dangerous consequences for their health The hospital library's experience and scope of practice offered an opportunity to train clinicians on this important and timely topic.

Description: In early 2022, the Health Sciences Library approached the Continuing Medical Education (CME) department to present a virtual Grand Round on the topic of health misinformation to an audience consisting of hospital and family practice clinicians. With the Surgeon General’s “Community Toolkit for Addressing Health Misinformation” as a foundation, the library created an interactive online presentation introducing tools and resources available for clinicians to communicate effectively with patients about health misinformation. The library worked in partnership with the CME and physician stakeholders through the process of Accreditation Council for Continuing Medical Education (ACCME) accreditation to make CME available to participants. Desired knowledge, competence, and performance were measured through interactive activities and surveys posted immediately after the activity and three months later.

Conclusion: Goals of the CME activity included increased knowledge about health misinformation and how to confront it. Outcomes included awareness of tools available; improved skills in differentiating health misinformation from evidence-based information; and better recognition of the value of evidence-based information and how it improves patient care outcomes and increases trust in the medical profession. Preliminary results show that clinicians were able to define health misinformation and identified actions to undertake in confronting it in the clinical setting. More than half planned to make changes in practice.

Creating Discipline-Specific Systematic Review Guidance for Food Science

Track: Innovation & Research Practice

Carol Hollier - Senior Information Literacy and Outreach Manager, IFIS Publishing, Wokingham, United Kingdom

Background: The number of systematic reviews published in the area of food science has, as in other fields, skyrocketed in recent years. But those reviews sometimes betray confusion over the principles and processes which underpin a systematic review’s integrity. Concerns about the reliability of reviews published in the field were raised by a board of faculty advisors to an international organization devoted to educational and information support for food scientists. Part of the problem might have stemmed from the fact that no discipline-specific systematic review guidance existed. The organization took on the challenge of producing this guidance.

Description: Developing the guidance, which resulted in an online handbook, was a collaborative endeavor. After extensive research of literature and guidance in neighboring disciplines, a guide for food science was drafted by two main authors, a research and regulatory consultant food scientist and the organization’s librarian, both of whom had experience in the methodology. The first draft of the guide was distributed to the organization’s faculty advisory board and their newly formed librarian advisory board for review. Feedback led to extensive revisions. Ninety pages of material were condensed to thirty, with some of the earlier material retained as supplementary material to the core guide. More extensive templates were developed to make the guide more hands on, and a skew towards nutrition which had crept in was corrected. The handbook points users to other sources of guidance when they are applicable, as food science is an extremely interdisciplinary field, but it also aims to stand as solid guidance for food scientists in the instances when other guidance is not appropriate.

Conclusion: In its first month of publication, online statistics show the handbook has been viewed more

than 1000 times. We plan to solicit feedback from researchers and librarians who use the guide over the coming year. We will then revise its content based on that feedback and a new round of advisory board advice. The hope is to raise the quality of systematic reviews submitted for publication in the field. We may be able to measure success in part by anecdotal evidence shared by board members who are journal editors, who were the first to raise their concerns and suggest that this project should be pursued.

Creating Recommended Practices for Retractions: An Update from the NISO CREC Working Group

Track: Information Management | On Demand

Caitlin Bakker, AHIP - Discovery Technologies Librarian, University of Regina, Regina, Saskatchewan, Canada

Background: The inconsistent representation of retraction publications poses challenges for librarians and library users, and is indicative of the broad range of workflows and the lack of international standards in this area. To address these issues, NISO formed the Communication of Retractions, Removals, and Expressions of Concern (CREC) Working Group which consists of 27 members representing publishers, aggregators, libraries, and other stakeholders.

Description: After establishing the scope of work, the CREC Working Group undertook an intensive information gathering process to understand the current landscape, including the broad range of challenges faced by both producers and users of content. Workflows associated with producing, transmitting, receiving, and displaying metadata related to retractions were documented, and best practices in these areas are being developed to create greater consistency and improve the experience of information consumers.

Conclusion: This presentation will describe the process of developing a NISO recommended practice to support consistent, timely transmission of retraction information.

Deepening Research Capacity of Health Sciences Librarians: Effectiveness of the MLA Research Training Institute (RTI) Online Program

Track: Innovation & Research Practice

Susan Lessick, AHIP, FMLA - Distinguished Librarian Emerita, University of California, Irvine, Anaheim, California

Jodi L. Philbrick, AHIP - Principal Lecturer, University of North Texas, Denton, Texas

Emily Vardell, AHIP - Assistant Professor, Emporia State University School of Library and Information Management, Olathe, Kansas

Ana D. Cleveland, AHIP, FMLA - Regents Professor, Sarah Law Kennerly Endowed Prof., Director. Health Informatic Program, University of North Texas, Denton, Texas

Background: Launched in 2018, the MLA Research Training Institute (RTI) aims to address the gap in research education for health sciences librarians by providing advanced research training and support to increase their research competencies, confidence, and productivity. In 2020 RTI’s in-person curriculum was transitioned to a fully online program consisting of 16 online modules, mentoring support, a one-year hands-on research project experience, and a capstone research presentation at the MLA annual meeting. The number of participants was also expanded, including a select number of LIS graduate students who

participate in the institute alongside practicing librarians. The RTI online program (tested and refined in RTI ’21 and RTI ’22) is funded in part by the Institute of Museum and Library Services (IMLS).

Description: This paper will focus primarily on assessment methods and outcomes for RTI ‘21 and RTI ’22. Multiple assessments of RTI’s effectiveness are ongoing and include prior research engagement of participants, research confidence of participants across three time-points (pre-, post-, and one-year after the core modules), perceptions of participants concerning program performance and learning outcomes, and program impacts on participants’ research attitudes and behaviors in their institutions. The instrument used for assessing research confidence of participants is based on methods of Brancolini and Kennedy (2017) and adapted for the RTI training model. The Wilcoxon Signed Ranks Test was performed to determine if there were statistically significant differences in the self-reported research confidence of the participants before and after the delivery of core modules each year. The non-parametric Friedman’s test of differences was used to evaluate the differences in median research confidence levels of RTI Fellows across three different time points. Content analysis was used for theming open-ended responses in participants’ reports concerning RTI program impacts. Descriptive statistics were used to analyze data related to participants’ perceptions of program performance and learning outcomes.

Conclusion: RTI assessment findings for three RTI institutes (2018-2020) demonstrated that the RTI training model is sound and effective, and increases the research competencies, confidence, and productivity of participants, as well as having other positive impacts. This paper will describe assessment findings related to the two revamped RTI online institutes held May 2021-June 2022 (RTI ’21) and May 2022 – June 2023 (RTI ’22). These findings will show shifts of research confidence levels due to RTI online program participation and be compared with outcomes of the prior RTI in-person programs. Comparisons will also be made between the outcomes of librarian and LIS graduate student participants. Authors will add these results and offer conclusions in February 2023.

Developing a Culture of Research in a Health Sciences Academic Library

Track: Professionalism & Leadership | On Demand

Michele R. Tennant, AHIP, FMLA - Interim Senior Director, University of Florida, Academic Research Consulting & Services, Gainesville, Florida

Hannah F. Norton, MS, AHIP - Chair, Health Science Center Library - Gainesville, University of Florida, Health Science Center Libraries, Gainesville, Florida

Background: Librarians who perform research bring many skills, experiences, and insights to the table. The results of their research can inform practice at their libraries and provide models for others. Participating in and leading research projects provides opportunities to collaborate with other librarians, as well as with library users. Engaging in research can offer librarians a more direct understanding of the challenges that library users experience when performing research. And for those at an institution where research has an impact on librarians’ tenure and/or promotion, it is essential. Since 2009, the Health Science Center Libraries (HSCL) at the University of Florida has embarked on creating a culture of research within the library.

Description: In 2009, a change in leadership at the HSCL brought about a change in attitude regarding research performed by the library’s faculty and staff. This began an intentional shift toward supporting such research endeavors, required for tenure and promotion within the libraries. Supportive measures were developed at all levels - leadership, departmental, and individual – to engender buy-in for library-based research; support research activities through training, release time, and funding; and promote an overall culture of research. These strategies resulted in an increase in the number of presentations, publications and grants/contracts coming out of the HSCL, and anticipated changes to the tenure and promotion process

that brought the libraries across campus more in line with the requirements of the rest of the University of Florida research community. This presentation will focus on the intentional strategies HSCL leadership employed for new hires and existing librarians, how to get started in research, and lessons that HSCL administration and faculty/staff have learned throughout this journey.

Conclusion: The advantages of library-based research are apparent, but a culture of research can be difficult to implement without a deliberate strategy, especially when research is a new emphasis. To develop such a culture, library leadership, as well the librarians on staff, must have a sufficient level of buy in, and such buy in is facilitated through strong institutional support, collaboration, and individual accountability.

Developing and Assessing Data Workshops Supporting Local and Global Research and Service Projects

Track: Information Management | On Demand

Margaret Henderson, AHIP - Health Sciences Librarian, San Diego State University Library, Ramona, California

Background: Growing Research Data Services (RDS) is a [university name] Library goal, and data management training for students was requested by faculty and the graduate school dean, so the health sciences librarian secured a NNLM grant to develop a series of workshops. Funder emphasis on data management and sharing has increased the need for libraries to help train student researchers in these skills. This new series of data workshops is designed to introduce health sciences students to open data, and help them learn how to manage, analyze, and visualize data. The goal is to create a series of workshops that can be presented to students and faculty to support their research projects, using modules that can be reused in other contexts.

Description: The “Finding, Managing, and Analyzing Data for Your Health Sciences Research” workshop series was developed to help students find open data on health, demographics, the environment (eg. climate change), and geographical information that can be used to understand and support the communities they work with. Health sciences students are encouraged to conduct research or service projects in the local area, or any of the international sites where health sciences faculty conduct research. For example, the School of Public Health has study abroad, international courses, and research activities in Germany, Chile, Mexico, Peru, Uganda, Nigeria, Brazil, Costa Rica, Columbia, Vietnam, and India. Finding government and other open source data will be demonstrated, and methods to combine data from different sources will be reviewed. Workshops will also cover ethics, social justice, health disparities, and the value of open science.Some students also have the option of a capstone research project, and secondary analysis will also be presented as an option for that type of project.

Conclusion: Formative assessment will be administered at the end of each workshop, and a month-out survey will check if participants were able to find the data they needed, as well as the status of their projects. Follow up surveys will be sent to ascertain the long-term impact of the training. All surveys will help to determine if there is a need to offer refresher workshops, or create a community of practice group, and will also be used to plan or adapt future iterations of the workshop series and any openly available teaching materials.

Developing Educational Materials on the Medicalization of Race

Track: Education | On Demand

Nina Stoyan-Rosenzweig - Archivist and Historian, Health Science Center Libraries/University of Florida, Gainesville, Florida

Background: This paper describes a project—funded by an internal fellowship—to develop educational materials focused on describing and discussing the historical development of modern ideas about and, particularly, ideas about biological differences between races and how those differences are believed to influence health and wellbeing. This funding allowed for research in four archives whose collections include a focus on eugenics and 19th century theories on race and racial origins. Information on other topics was accessed through digitized archival collections & other online sources, and published materials filled in gaps. This portion of the project also focused more on ideas about race, ethnicity, health, and illness in America and explored ideas developed through immigration, African slavery, imperialism and other areas.

Description: This project developed a comprehensive list of topics that could be covered in 500 words, and that could be covered chronologically, or topically, thus providing individuals choices as to how they can delve into the material. Upon completion, the material will be available in an online database with an organizational tree further enabling users to envision how they can select particular topics as well as understand how ideas developed because of specific interactions with disease, needs to justify continuing enslavement, spread of European settlement, to control or limit immigration, access to political power, fear of change, and other factors that can drive how individuals and their societies react to how their world develops. The impetus for creating this project was the lack of educational materials that were comprehensive but focused so their overall relationship and structure was understood and students could see how they were all related to one another. This project also works with students and individuals who want to research and write on topics and contribute to the project, and develops to provide material for doctors, medical students, other healthcare providers and students, and undergraduates interested in the topic.

Conclusion: This project outcomes will include the web based program that facilitates use by healthcare providers, students, and instructors, as well as outreach to courses, and development of a libguide to provide information on digitized archival collections, image collections, and databases, as well as physical archive collections available for further research on the topic. Outcomes will include measure of usage of the materials and the libguide and engagement with student researchers and writers.

Development of an Introductory, Online, and Open Research Curriculum

Track: Innovation & Research Practice | On Demand

Emily S. Mazure, AHIP - Clinical & Research Librarian, MAHEC, North Carolina

Chelsea Atkins - Research Associate, Research at MAHEC

Charles Micha Belden - Research Scientist, Mixed Methods, Research at MAHEC, Asheville, North Carolina

Wendy Nuzzo, MS - Research Data Analyst, Research at MAHEC

Edward C. Jauch, MD, MS FAHA - Chair, Department of Research at MAHEC

Jacqueline Halladay, MD, MPH - Professor, Department of Family Medicine, UNC School of Medicine

Background: Across many organizations of higher learning, educators have created courses to enhance fundamental research knowledge and skills. However such courses are often restricted and difficult to access. Learners seek opportunities to engage in scholarly activities that can be included on resumes/curricula vitae. We set out to understand which key research topics were deemed important, with the aim of creating a brief research curriculum for those without opportunities to attend longer courses and/or who may benefit from "just in time" exposure to key research concepts. We created a novel research

curriculum to provide foundational knowledge for diverse learners to become familiar with common research terms and processes to better prepare them to understand and perform research.

Description: An interdisciplinary team of researchers, clinical educators, and librarians formed to develop a self-paced, online, open curriculum on basic research concepts. Initial discussions focused on selecting key topics to include in the module-based curriculum. The final modules covered 8 topics: 1) Formulating a research question, 2) Literature searching, 3) Critical appraisal, 4) Qualitative research, 5) Quantitative research, 6) Data management, 7) Protection of human subjects, and 8) Manuscript preparation. For each module, several learning objectives were developed. To conserve our time and effort, we directed learners to freely available videos, articles, and other resources when possible. When unable to find freely available content that covered the objectives for a module, we developed content ourselves. For each module, we developed simple assessment questions for students to test their knowledge. For the course as a whole we developed a simple form learners can complete to self-attest and automatically receive a certificate of completion. Learners are asked to provide feedback for each module and the collective course. The course content is hosted in Sprinshare’s LibGuides, with self-assessment quizzes in LibWizard. Voluntary feedback for each module and the overall course is collected via LibWizard.

Conclusion: After the course goes live, we plan to collect basic usage statistics of the course content including number of page visits, link clicks, file downloads, etc. We will collect and explore basic demographics of learners who attest to completing the course. We will gather and report on feedback from learners related to their experience of and knowledge gained from each module, and the course as a whole. Finally, we will compile feedback from key stakeholders, including graduate medical education program directors. We expect to have approximately 5 months of data to report.

Eliminating Barriers to Publishing Case Reports in Open-Access Journals for Medical Trainees

Track: Information Services | On Demand

Elizabeth R. Lorbeer, AHIP - Chair, Medical Library, Professor & Library Director, Western Michigan University Homer Stryker M.D. School of Medicine, Kalamazoo, Michigan

Ann Harris, AHIP - Senior Informationist & Medical Librarian Team Leader, Assistant Professor, Western Michigan University Homer Stryker M.D. School of Medicine, Kalamazoo, Michigan

Juli McCarroll, AHIP - Informationist/Medical Librarian & Assistant Professor, Western Michigan University Homer Stryker MD School of Medicine Medical Library, Kalamazoo, Michigan

Background: A learner’s first peer-reviewed clinical sciences paper is often a case report. This publication type is attractive to early career researchers as it focuses on a single clinical presentation of a patient exhibiting unusual symptoms, followed by a diagnostic assessment, a therapeutic intervention, management, and outcome. Clinically meaningful and novel cases are used as teaching tools in the educational setting to practice problem-solving skills, such as systematically approaching a differential diagnosis, integrating evidence-based medicine by practicing literature searching in PubMed, and studying disease states. Trainees, as they rotate through the medical specialties in the 3rd and 4th years of medical school, present clinical cases of patients they have followed in the in-patient or ambulatory setting at teaching rounds.

Description: Trainees are encouraged to co-author their case with the attending physician when they have identified an unusual presentation that warrants reporting in the medical literature. However, the fees associated with publishing are a barrier to submitting a manuscript. Learners reported they could not afford the fellowship fee for BMJ Case Reports and the optional Open Access (OA) fee to make the paper available upon publication. It led to authors withdrawing their accepted manuscripts and letting them go

unpublished or seeking out other journals without an article processing fee. Then, that led to the learner alternatively finding a journal title not indexed by PubMed and publishing the work with a potential predatory open-access publisher. In June 2018, the library piloted a 19-month institutional subscription to BMJ Case Reports, which paid the fellowship fee for all affiliated institutional authors. Before the pilot, 19 case reports were published from January 2012 to May 2018, compared to 12 case reports published during the pilot period, from June 2018 to December 2019. The pilot was considered a success. Post-pilot, the library maintained a subscription to BMJ Case Reports. For 2020 and 2021, 16 of the 20 case reports had a trainee as the first author.

Conclusion: The library absorbing fees associated with publishing through transformative agreements allows ECRs equity and inclusion into the academic medicine community as they establish their career. Additionally, as medical schools respond to social justice and health inequity issues within the community they serve, the library is well positioned to respond by supporting scholarly productivity attainable for all authors at any stage of their career.

Escape the Lecture: Using a Virtual Escape Room Activity During Library Instruction for Medical, Nurse Anesthesia, and Physician Assistant Students

Track: Education | On Demand

Rozalynd P. McConnaughy, AHIP - Assistant Director for Education & Outreach, School of Medicine Library, School of Medicine Columbia, University of South Carolina, Columbia, South Carolina

Steven P. Wilson, MLIS, MA, AHIP - Web Architect & Outreach Librarian, School of Medicine Library, School of Medicine Columbia, University of South Carolina, Columbia, South Carolina

Ruth A. Riley, MS, AHIP - Assistant Dean for Executive Affairs & Director of Library Services, School of Medicine Library, School of Medicine Columbia, University of South Carolina, Columbia, South Carolina

Background: The objective of this project was to design a virtual escape room as an engaging activity for students to get hands-on practice searching databases during library instructional sessions. The Library strives to include hands-on activities in all of its curriculum-integrated instruction. Having seen examples of virtual escape rooms used in undergraduate library sessions, the lecturing librarian offered to design a virtual escape room activity for the physician assistant course, for the nurse anesthesia course, and for incoming medical students during orientation, where students would work in-person to solve puzzles and find answers to questions using specific library resources. All three course directors were excited about the activity.

Description: LibWizard from Springshare was the platform used to create the virtual escape room, Defeat the Disinformationists! The same scenario was used for all three escape room sessions, but the puzzles and searching questions were adjusted for the different student groups. The medical students had forty minutes to complete six puzzles and correctly answer eight questions that required students to search six different resources. Compared to the other escape room sessions, this was the only student group that did not receive in-person instruction. The medical students were assigned an orientation tutorial to go through prior to the session for an overview of the information resources that they would use in the escape room. After introducing the scenario and rules for the escape room, three librarians served as “gamemasters” to answer questions and provide hints as the students worked through the activity. The physician assistant students had sixty minutes to solve eight puzzles and fourteen searching questions. The nurse anesthesia students had forty-five minutes to resolve five puzzles and ten searching questions. The nurse anesthesia course was unique in that some of the students were participating virtually from a satellite campus, so they had the option of calling or texting with questions.

Conclusion: Although each of the virtual escape rooms were different, the same optional, five question survey about the experience was used for each session to collect feedback. Overall, the students reported their skill level as more advanced after completing the escape room. Sixty-nine percent of the survey respondents rated their skill level in using library resources as “novice” or “advanced beginner” prior to the session. After the session, ninety-six percent rated their skill level as “competent”, “proficient”, or “expert.” In the open-ended questions, students commented that they enjoyed the interactivity and found the session engaging and fun.

Expanding Library-Based Research Support through Innovative Interdisciplinary Services: Academic Research Consulting and Services (ARCS)

Track: Innovation & Research Practice | On Demand

Michele R. Tennant, AHIP, FMLA - Interim Senior Director, University of Florida, Academic Research Consulting & Services, Gainesville, Florida

Aida T. Miro-Herrans, PhD - Bioinformatics Librarian, University of Florida, Academic Consulting Research & Services (ARCS)

Borui Zhang - AI/Natural Language Processing Specialist, University of Florida, Academic Consulting Research & Services

Chelsea Johnston - Scholarly Publishing and Repository Librarian, University of Florida, Digital Partnerships and Strategies

Hao Ye, PhD - Reproducibility Librarian, University of Florida, Academic Research Consulting & Services (ARCS)

Joe Aufmuth, MS - Spatial Data Consultant, University of Florida, Academic Consulting Research & Services

Natya Hans - Informatics Librarian, University of Florida, Academic Research Consulting and Services (ARCS), Florida

Patti McCall-Junkin, MA, MLS - Systematic Review Librarian, University of Florida, Academic Research Consulting & Services (ARCS), Gainesville, Florida

Plato L. Smith II - Data Management Librarian, University of Florida, Academic Consulting Research & Services (ARCS), Gainesville, Florida

Sarah Meyer - Assistant University Librarian, University of Florida, Health Science Center Libraries, Gainesville, Florida

Suzanne Cady C. Stapleton, MS - Agricultural Sciences & Digital Scholarship Librarian, University of Florida, Marston Science Library, Gainesville, Florida

Tara Tobin Cataldo, MLS - Biological Sciences Librarian, University of Florida, Marston Science Library

Terry K. Selfe, AHIP - Translational Research and Impact Librarian, University of Florida, Academic Research Consulting and Services (ARCS), Gainesville, Florida

Tiffany C. Esteban, MSI - Digital Humanities Associate, University of Florida, Humanities and Social Sciences Library West

Xiaoli Ma, MSI, MA - Metadata Librarian, University of Florida, Resource Description

Background: The George A. Smathers Libraries have a long history of identifying information service gaps and filling them through innovative hires (e.g., bioinformatics, geospatial analysis.) The addition of data

management and informatics positions, as well as a growing technical skillset among existing librarians, provided the opportunity for synergy and collaboration in the library’s research support services. In 2018 the Academic Research Consulting and Services (ARCS) team – meant to provide such research support services – was introduced to the University of Florida campus. ARCS now includes 15 experts, and continues to support research and research-related instructional efforts across campus. This presentation addresses the need for such services; describes the services provided, subteams created, and collaborations developed throughout campus; and discusses ARCS’ plans for the future.

Description: ARCS provides expert support services throughout the research process – from research design, through data collection, analysis and management to publication and beyond. Team members have expertise in artificial intelligence (AI) and natural language processing, bioinformatics and genomics, data management and archiving, data science, data visualization, digital humanities, geospatial analysis, informatics, open science, public access compliance, publishing and copyright, research metrics and impact, research reproducibility, responsible conduct of research, statistical analysis, and systematic reviews. ARCS subteams have organically grown based on university needs and initiatives, including those related to AI, informatics training, and research metrics and impact. A partnership has been forged with Research Computing allowing unfunded students access to the university’s high-performance computing cluster. ARCS has developed close working relationships with the Office of Research, Research Computing, and the Center for Undergraduate Research, among other campus entities. ARCS members consult with faculty, students, and staff from throughout the university on projects for all disciplines and of all sizes, whether funded or not, and provide expertise to grant submissions at the pre-proposal as well as post-award stages. ARCS members provide research-related instruction in a variety of formats (course-integrated, library-created credit-bearing courses, workshops) and venues (laboratories, unit meetings, journal club).

Conclusion: Effective library-based research support can take many forms, and the inclusion of technological, methodological, and interdisciplinary expertise can successfully meet the needs of patrons and address campus research support gaps. Increasingly complex scholarship provides the rationale for these services being provided by the library since researchers are familiar with library support of all disciplines and researcher needs in information management, data, and publications are longtime library domains. A large team with diverse skillsets can create synergies in library-based research support, and facilitate partnerships between the libraries, other research-support entities on campus, and individual researchers. ARCS at the University of Florida can serve as a model for other institutions looking for innovative approaches to library-based research support.

An Exploration of Workflows for Monitoring Open Access Publishing at the University of Michigan

Track: Information Management | On Demand

Tyler Nix, MSLS - Assistant Director (Interim), Research & Informatics, Taubman Health Sciences Library, University of Michigan, Ann Arbor, Michigan

Leena Lalwani - Associate Director HS-STEM/Engineering Librarian, University of Michigan, Ann Arbor, Michigan

Rebecca Welzenbach, MSI - Research Impact and Information Science Librarian, University of Michigan Libraries, YPSILANTI, Michigan

Background: Dr. Alondra Nelson's August 2022 Office of Science and Technology Policy (OSTP) memo, "Ensuring Free, Immediate, and Equitable Access to Federally Funded Research," calls for federal agencies to develop or update plans to ensure immediate, public access to federally-funded research publications and data. The memo is expected to advance change across the Open Access (OA) publishing landscape, placing increased significance on the authors' ability to track and report on [Institution's] OA publishing

characteristics. Where ad hoc database searches for OA publication trends met our institutional needs in the past, the authors have begun intentionally testing and documenting select workflows to better understand pros and cons of various OA tracking methods.

Description: The authors will describe the administrative context for OA reporting efforts at [Institution], and will report on three OA tracking methods, their pros and cons. The three OA tracking workflows will include: 1. Periodic search and collection of OA publication rates in Scopus, Web of Science, and Dimensions. Each database is capable of institutional filtering, and filtering to Green, Gold, and Bronze OA status. 2. Using OpenRefine and the Unpaywall API to query OA publications by institutional affiliation [1]. 3. Coordinating with [Institution's] faculty expertise platform (Symplectic Elements) and administrative team to test the feasibility of OA tracking in an administrative module, as well as the feasibility of tracking OA publications at the departmental level. We will evaluate OA tracking workflows based on administrative reporting as our primary end use. Rather than attempting a comprehensive bibliographic analysis of [Institution's] OA publishing trends, our goal is to test workflows based on efficiency and their ability to track with requisite granularity (e.g., Green, Gold, and Bronze OA) to inform administrative awareness and OSTP policy compliance.

Conclusion: If selected to present, we will share an overview of these three workflows and various strengths and limitations associated with each. We will also provide context on how the workflows have or have not met our institutional reporting needs, and any future plans for OA tracking and administrative reporting at [Institution]. References 1. Langham-Putrow A, Enriquez A. Analyzing institutional publishing output: a short course. [Internet]. Penn State Universities ScholarSphere. [version 3, March 21, 2022; cited October 31, 2022]. .

Forging Ahead After the Shake-ups

Track: Professionalism & Leadership | On Demand

Sheila W. Green, AHIP - Associate Librarian, Texas A&M University Medical Sciences Library, Bryan, Texas

Paria Aria - Coordinator of Client Services, Texas A&M University Medical Sciences Library

Robin Sewell - System & Data Analytics Librarian, Texas A&M University Medical Sciences Library, College Station, Texas

Ana Ugaz, MLS - Librarian Director, Texas A&M University Medical Sciences Library, College Station, Texas

Stephanie Fulton, AHIP, FMLA - Associate University Librarian, Texas A&M University Medical Sciences Library, COLLEGE STATION, Texas

Background: Texas A&M University embarked on a reorganization of academic and service groups called “Path Forward” in 2022. University wide business services, HR, marketing, facilities, and IT functions were consolidated, and the University Libraries were shifted from an academic to a service unit. Faculty librarians could stay with the library as staff or find a faculty role with an academic department. Between previously unfilled positions, COVID, and the Path Forward, the Medical Sciences Library (MSL) contracted from a team of eighteen librarians to six. The loss of expertise and experience limited the ability of remaining librarians to provide the same level of public services. This paper addresses MSL’s approach to forging ahead with services while simultaneously rebuilding teams and raising morale.

Description: The Path Forward for MSL was threefold - provide services for the looming academic year with limited staff, identify future goals, and rebuild a team to maximize opportunities. The Libraries have a clear mandate from University leadership to examine operations and look deeper than we might otherwise for innovation. We reviewed analytics, discovered gaps, and identified services to stop now, pause or start.

Instructional and research requests were distributed amongst the librarians to manage the workload. We communicated changes to school deans and academic leaders in the health sciences and veterinary medicine and discovered their Path Forward goals, agreed upon priority hires, and discussed collaborations for mutual success. These processes informed decisions about the structure and skills needed for our new team. By reviewing the organizational chart, the Libraries’ leadership was able to create a new reporting structure that better reflected the services and created more collaboration among different units. The transition from faculty to staff allowed the libraries to expedite its hiring process and now we are in the process of hiring and rebuilding our education and research team.

Conclusion: The Path Forward, while disruptive to the previous status of operations, provided opportunities for the MSL and the Libraries to consider innovative solutions. The new career ladder created for staff librarians gives advancement opportunities to library staff who have earned an MLS. The librarian model is enhanced to incorporate functions, such as education or research focus, and campus locations as well. MSL’s collection team remained intact and is taking the lead on reviewing the print collection in anticipation of space changes. We are also enhancing collaboration across MSL and the Libraries and removing silos which previously existed. Although our team was significantly reduced, the opportunities presented are giving us hope to rebuild a 21st century library.

Forging Ahead with the NAHRS Nursing Essential Resources List

Track: Information Services | On Demand

Rebecca Raszewski, AHIP - Associate Professor, University of Illinois at Chicago, Chicago, Illinois

Alissa Fial - Head, Research & Instructional Services, Associate Librarian, Raynor Memorial Libraries Marquette University, Milwaukee, Wisconsin

Rachel Keiko Stark, AHIP - Health Sciences Librarian, California State University, Sacramento Library, Research and Instruction, Sacramento, California

Karen S. Alcorn - Reference and Instruction Librarian, Blais Family Library, Worcester, Massachusetts

Carolyn Ching Dennison, AHIP - Librarian, University of Hawaii at Manoa Library, Hawaii

Lorraine Porcello - Evidence-Based Practice & Instruction Librarian, University of Rochester, Miner Libraries, Rochester, New York

Basia Delawska-Elliott, MLS, AHIP - Medical Librarian, Providence System Library Services, Portland, Oregon

Background: The NAHRS Nursing Essential Resources List (NNERL) Working Group took on the task of updating the (26th) edition Essential Nursing Resources formerly created by the Interagency Council on Information Resources in Nursing, last published in 2012. Volunteers recruited from the NAHRS Caucus have worked on the NNERL project since October 2020. The goal of the NNERL Working Group is to create a nursing resource list that is available to a broad audience, represents current materials and formats, and utilizes a selection process that is transparent and reproducible.

Description: The NNERL Working Group has three areas of concentration: evaluation of current and prospective list items; an item-level metadata creation process to ensure that resources are easily findable; and format and accessibility of the NNERL. During the Summer of 2022, the NNERL Working Group finished updating and scoring resources from the 26th edition. The total number of remaining resources is 210 as of May 2023. One hundred eighty-three resources were eliminated from the original list due to being discontinued, outdated, or irrelevant to nursing. In tandem with the screening of resources for the NNERL, two subgroups formed within the Working Group. The Tagging Subgroup developed metadata generation guidelines and metadata tags to describe the content of each resource and assist in findability. The NNERL Working Group recruited 20 MLA and/or NAHRS members to tag all 210 resources. Two information professionals independently evaluated each list item and assigned tags identifying the item’s format, cost,

and topics. Similarly, two information professionals independently reviewed each list item using a tool developed by the Evaluation Rubric Subgroup. The rubric scores use a numeric system to indicate existence of information about authorship, currency of publication, intended audience, and potential areas of bias or conflict of interest. At this time, the rubric score is not intended to connote validity or reliability. Rather, the score is metadata that is useful for data visualization and discovery.

Conclusion: The future draft of the NNERL will include complete APA Style citations, content metadata tags, and a rubric score for every item on the list. The NNERL will be copyrighted to the NAHRS NNERL Working Group and will be made available to all MLA members under an Attribution-NonCommercial-NoDerivatives Creative Commons License. The Working Group aspires to make the NNERL available via an open internet source and therefore be available to the general public under the Creative Commons License.

Forging Collaboration: Centering the Library in Coordinating Education on the NIH Data Management and Sharing Policy at an Academic Medical Center

Track: Information Services | On Demand

Michelle Yee - Senior Project Coordinator, NYU Health Sciences Library, New York, New York

Nicole Contaxis, MLIS, MA - Data Librarian and Lead of Data Discovery, NYU Health Sciences Library, New York, New York

Background: Policy changes from the National Institutes of Health (NIH) present a novel opportunity for libraries to center themselves in coordinating data management policy changes within their institutions. The new Data Management and Sharing (DMS) policy requires researchers to submit DMS plans with their applications for funding. Per the policy, an investigator’s non-compliance with approved plans may reflect on future funding for an institution as a whole, necessitating widespread awareness. At an academic medical center, librarians led a coordinated cross-departmental effort to disseminate information and skills about the forthcoming DMS policy.

Description: When the NIH DMS policy was finalized, the library approached institutional research leadership with concerns about institutional readiness. To prepare, leadership and the library created a cross-departmental working group to determine educational and outreach priorities for the policy. Working group membership included representation from academic departments, shared scientific cores, the institutional review board (IRB), information technology, and research administration offices. With this working group, the library developed a multi-pronged approach to educate researchers and staff impacted by the NIH DMS policy through synchronous classes and asynchronous materials distributed through official channels. Training courses were tailored to meet various needs of research and grant staff: voluntary synchronous classes were approximately an hour long; and a 15-minute asynchronous online course was developed to cover DMS policy fundamentals, including elements of DMS plans. The cross-departmental working group partnership will extend beyond the rollout of training through participation in a shared inbox for DMS queries that is managed by representatives from the offices participating in the working group.

Conclusion: Spearheading the education effort, the library will be able to access metrics on attendance to trainings on the NIH DMS. To date, the synchronous workshops have been attended by over 200 research staff and a newly launched institutional homepage on the DMS policy has received nearly 500 views. The asynchronous course is accessible to all research staff at the institution through a distributed learning platform. The volume of queries received through the shared inbox and DMPTool feedback request function will be assessed at a future date. Direct feedback from researchers will be an invaluable metric for the effectiveness of available resources and for continued development of materials to support policy compliance.

Forging Connections through Collection: Reinvigorating Community Engagement with a Graphic Medicine Collection

Track: Information Services | On Demand

Renee A. Rau - Information Services Librarian, Norris Medical Library, Los Angeles, California

Background: This paper examines the creation of a Graphic Medicine collection: how it was funded, developed, promoted, and takeaways. The library lacked a defined Graphic Medicine collection and sought a collection as a way to provide both leisure reading and informal learning opportunities. The genre of Graphic Medicine is a visual medium featuring topics related to health, wellness, and healthcare systems. It provides readers with ways of engaging with diverse perspectives on health. This collection complements an existing leisure reading collection, better supports cultural competency courses, and the narrative medicine program. As an urban health sciences campus, topics like LGBTQ+, maternal, sexual health, and trauma are commonly encountered and are discussed in curriculum, and are the focus of this collection.

Description: During the academic year 2021-2022, the library applied to the Network of the National Library of Medicine Region 5 Collection Equity Award with the goal of building a Graphic Medicine collection and promoting its use within the medical community and the broader University community. The Collection Equity Award sought to “help build collections that are reflective of voices of the communities served which may be revealed through different genres.” The awardee formed a cross-departmental team including cataloging, grants, communications, and programming, and created a collection in four months. The collaboration in purchasing and promotion also resulted in the creation of an exhibit, and once launched, led to further opportunities to present in the curriculum and co-curricular activities of the School of Medicine, and proposals for campus-wide programming on graphic medicine with the School of Medicine. There have been opportunities to present in Narrative Medicine courses, guest lecture in a Lunch and Learn series hosted by a humanities and medicine program, and invitations to provide elective courses for students transitioning to residency. The campus-wide programming opportunity seeks to collaborate with School of Medicine faculty and bring influential illustrators and creators in Graphic Medicine, web comics, and medical illustration.

Conclusion: This grant and collection have had a consequential impact on our community. The exhibit includes topic-targeted panels, allowing patrons to explore each of themes in this collection and view exemplars. The graphic medicine collection circulates at a higher rate than the existing leisure reading collection, and surprisingly, has had a number of ILL requests, allowing the library to strengthen our ties within our local lending communities. There have been requests for instruction, and the library has been invited to collaborate on future graphic medicine related events across the university increasing the library’s partnerships and presence.

Forging Data Skills: A collaboration to offer The Carpentries to multiple institutions

Track: Education | On Demand

Christine Nieman - Data Management Librarian, University of Maryland, Baltimore, Network of the National Library of Medicine Region 1, Baltimore, Maryland

Peace Ossom-Williamson - Associate Director, NNLM National Center for Data Services, Fort Worth, Texas

Katie Pierce-Farrier - Data Science Strategist, University of North Texas Health Science Center, NNLM Region 3, Fort WOrth, Texas

John C. Bramble - Executive Director, University of Utah Eccles Health Sciences Library, Salt Lake City, Utah

Sean Corning, MLIS - Education and Outreach Coordinator, Lamar Soutter Library

Elizabeth Roth - Research and Data Science Strategist, NNLM Region 2, Charleston, South Carolina

Background: The Carpentries is a community-led organization teaching foundational coding and data science skills. It includes lessons for Data Carpentry for learning data literacy, Software Carpentry for learning programming languages, and Library Carpentry, which is focused on teaching information professionals these skills while empowering them to advocate for reproducible data practices. Libraries are at the forefront of offering Carpentries workshops. Typically, organizations purchase a membership or number of workshops organized through The Carpentries. Hosting workshops is a major undertaking requiring institutional staff time and resources. As a collaborative network of multiple health sciences organizations, we coordinated efforts to offer frequently occurring workshops across a large user base and geographical area, while tailoring offerings to specific audiences.

Description: A collaborative, cross-institutional taskforce created a plan to facilitate and fund a series of Carpentries-hosted workshops. The taskforce assessed and discussed staff capacity and anticipated interest based on experience from hosting Carpentries workshops in the past to determine how many workshops to host and how to distribute workshops. One central office was designated to handle the coordination and communication with The Carpentries, including negotiating a discount for purchasing multiple workshops and liaising between Carpentries staff and local contacts at each partner organization to work out scheduling of workshops. We will discuss how we collaborated to develop this coordination plan, tips for successful implementation of workshops, and how we plan to evaluate this initiative in terms of redistributing staff effort and time and meeting the demand for Carpentries activities across a diverse and complex network of learners.

Conclusion: The team will measure the success of implementation techniques, including the frequency of offering, variation in time zones, similarities in format and differences in lessons across offerings. Success will be measured as engagement through registration and completion of workshops, post-workshop satisfaction survey data, and long-term follow-up evaluation of data skills attainment and use.

Forging the Future: Building a System to Cultivate a Cohesive and Collaborative Library Team

Track: Professionalism & Leadership | On Demand

Lin Wu, AHIP - Assistant Director for Research & Learning Service, University of Tennessee Health Science Center Health Sciences Library, Memphis, Tennessee

Rick Fought, EdD, MLIS, AHIP - Assistant Vice Chancellor, University of Tennessee Health Science Center Health Sciences Library, Memphis, Tennessee

Randall Watts, AHIP - Associate Director, University of Tennessee Health Science Center Health Sciences Library, Memphis, Tennessee

Jenessa M. McElfresh, AHIP - Systematic Review Service Coordinator / Senior Research & Learning Services Librarian, University of Tennessee Health Science Center Health Sciences Library, Memphis, Tennessee

Paul C. Gahn - Assistant Director for Electronic & Collection Services, University of Tennessee Health Science Center Health Sciences Library, Memphis, Tennessee

Kaitlyn Ritacca - Senior Reference Services Specialist, University of Tennessee Health Science Center Health Sciences Library

Background: Organizational change can make or break a library team. One academic health science center library has intentionally utilized a period of change to strengthen its workforce and services. Numerous upheavals including new leadership, space renovations, workforce reductions, and organization restructuring centered the need to cultivate a cohesive and collaborative team to ensure high quality, seamless library services. Successes, challenges, and tactics to improve productivity and avoid burnout supplement this paper’s findings. The paper demonstrates a holistic approach to allow team members to develop a sense of belonging, find value in their efforts, and become stakeholders in the library’s transformation.

Description: The library has onboarded 9 librarians since 2017. The team is currently composed of 10 professional librarians and 9 paraprofessionals. New hires came on board with diverse experiences, skills, knowledge, and backgrounds. These team building strategies employed an integrated approach including taking self-assessments using MLA Competencies for Lifelong Learning and Professional Success, collaborating on on-boarding materials, reviewing service practice guidelines, participating in professional development activities, shadowing library staff, attending department in-services, and engaging in strategic community of practices. The process of cultivating a cohesive and collaborative team also involves building soft skills such as open communication, providing and receiving constructive feedback, time management, budget management, and stress management. All library staff contribute to the process throughout large-scale library projects, which made it a collective effort not only supporting the new hires but enhancing the team cohesion and collaboration.

Conclusion: Data driven outcomes to be measured include such indicators as library budget increases, faculty salary comparisons with peer institutions, librarians’ achievements, impact on service provisions, and librarian-driven service development and expansion of collaborative professional development opportunities.

Free Money: Reevaluating, Reassessing, and Removing Barriers to Chapter Scholarships

Track: Professionalism & Leadership | On Demand

Electra Enslow, AHIP - Director, Clinical Research and Data Services Librarian, University of Washington

Basia Delawska-Elliott, MLS, AHIP - Medical Librarian, Providence System Library Services, Portland, Oregon

Mary Beth McAteer, AHIP - Medical Librarian, Virginia Mason Franciscan Health

Background: PNC/MLA Scholarship Committee has examined, expanded and updated our student scholarship application and distribution process. Our committee goals were to remove barriers to receiving the scholarship and that the money given was unrestricted. To achieve this goal we reviewed and simplified the application process and increased the number of scholarships and the amount of funds disbursed.

Description: The PNC/MLA Scholarship Committee started the review of our student scholarship application process in 2021. Our priorities were to simplify the process and make it more equitable. To achieve that goal, we increased the number of scholarships granted, added a category for underrepresented groups, and increased the scholarship minimum. We developed a rubric that would make the process more consistent and transparent. We eliminated routine requirements that we identified as discriminatory such as requiring chapter membership and letters of reference. We expanded this opportunity to include newly enrolled students by accepting a wider array of documentation such as acceptance letters instead of just transcripts. We extended our deadline to be more in line with the school year. The new rubric facilitated a quick turnaround time for application review and notification to recipients. We gave recipients a choice between being mailed a paper check or fund transfer depending on their needs. Expanded choices allowed

for quicker funds disbursement. In view of the process improvement success, in the fall of 2021 the PNC/MLA Scholarship Committee recommended removing the letters of reference requirement from the MLA scholarship process to the MLA Grants & Scholarship Committee.

Conclusion: After putting our scholarship application through a thoughtful and rigorous examination we were able to create an application process that is simplified, succinct, and equitable. All of these changes culminated in awarding funds to excellent candidates who expressed how much they helped ease their student experience. In August 2022 we were informed by the MLA Grants & Scholarship Committee that our recommendations have been accepted and implemented for the 2022-2023 scholarship year.

Healthcare & the Humanities: A Program for Healthcare Associates Hosted by the Nemours Medical Library

Track: Professionalism & Leadership | On Demand

Olivia E. DiLeonardo - Medical Librarian, Nemours Children's Hospital Florida- Medical Library, Orlando, Florida

Ann Ferrari, M.L.S., University of Pittsburgh, M.A.. Education, American University - Medical Librarian, Medical Library, Wilmington, Delaware

Susan Harnett, AHIP - Medical Librarian, Medical Library, Jacksonville, Florida

Background: Librarians at a multi-state pediatric health system have noticed increased stress, compassion fatigue, and a lack of fulfillment among healthcare associates. We sought to develop a program that would help mitigate these stressors and reduce alienation caused by COVID-19. Our program is grounded in principles of Narrative Medicine, and we chose Microsoft Teams as our meeting platform to make our program available to associates across three states. Participants who attend this session will be able to: • Use close reading and writing to process difficult events and mitigate compassion fatigue. • Discuss works of art, prose, poems, and graphic novels and reflect on their assumptions and biases. • Develop relationships with teammates based on shared discussions and participation in activities.

Description: We met with stakeholders, including our Chief Wellness Officer; the Office of Health Equity and Inclusion; and Marketing and Communications to discuss our ‘Healthcare & the Humanities’ (H&H) program. We also met with Continuing Medical Education to obtain approval to issue CME credit to participants. We decided to hold the program monthly and limit participation to a maximum of 15 attendees per session. Collectively, we chose the focus pieces and flow for the first three sessions- these included excerpts from graphic novels and works of poetry, followed by discussion. Pieces would be encountered as a group during the sessions; no advance reading required. We chose pieces coinciding with heritage months and identity recognitions (e.g. Black History Month and Latinx Heritage Month). We developed announcements to market H&H via organizational newsletters; we also added a dedicated program page to our library portal. Announcements included a link to register for the program via SignUpGenius. A week prior to the program, we sent a Teams meeting invitation to all registrants. We held our first program on January 19, 2022, for 10 attendees. 5-15 attendees have consistently attended subsequent sessions, with attendance trending higher as more associates become aware of the series. We are evaluating our program by conducting surveys and assessing informal feedback.

Conclusion: We will utilize pre- and post- intervention surveys to assess this program’s success at reducing levels of stress and compassion fatigue; increasing connectivity between team members across states and associates working remotely; and engendering reflection on participant biases and assumptions based on individual experiences. To date, we have held 11 H&H sessions. Informal feedback has been positive: participants have stated that they enjoy the sessions and appreciate the opportunity for meaningful discussion and reflection. We are partnering with Associate Resource Groups (groups bringing together

associates with similar demographics and interests, such as the African Heritage Group, Pride Group, and Caregiver Group) for future sessions to enrich discussions, obtain suggestions for additional focus pieces, and assess informal feedback.

Help Your Library Patrons Understand Clinical Trials: An Overview of a Clinical Trials Program for Rural Public Libraries

Track: Education | On Demand

Dana L. Ladd, AHIP - Health and Wellness Librarian, VCU Libraries, Lanexa, Virginia

Jackson Wright, - Health and Wellness Library Assistant, VCU Libraries, Richmond, Virginia

Background: Research suggests that rural and minority communities participate in research at lower numbers. Diverse representation in clinical trials is essential to ensure representation among populations who may use the medical resources. The [Redacted] to develop a workshop to promote clinical trials in rural areas of [Redacted] aimed at public libraries. We aimed to provide rural public librarians with the skills to increase the health literacy of community members by bringing awareness of clinical trials, how to participate in clinical trials, and how to find an appropriate clinical trial.

Description: We applied for and were awarded a Network of the National Library of Medicine (NNLM) Community Outreach grant. We developed and conducted Help Your Library Patrons Understand Clinical Trials Workshops. We planned and facilitated a speaker event: Promoting Patient Engagement through Clinical Trials featuring e-Patient Dave and we conducted two book discussion sessions of the book A Series of Catastrophes and Miracles. The workshop was designed to give library employees the confidence to search for health information for library patrons and included information on the basics of clinical trials, suggested methods for finding reliable consumer health information, and provided interactive demonstrations of MedlinePlus and ClinicalTrials.gov. To assess the program we collected the following data: number of registrations; number of attendees; formal evaluation question feedback, and informal feedback in the chat sessions of the programs.

Conclusion: We conducted five workshop sessions with a total of twelve public librarians attending. We used NNLM's required evaluation questions to assess the programs. Formal evaluation (n=2) and Zoom chat feedback for all clinical trials workshops and programs were positive. The library staff who participated expressed gratitude and information learned and that they would use the information to pass along to staff and to patrons. Sixty people attended the e-Patient Dave speaker Zoom speaker event. We provided two book discussion sessions using the book, A Series of Catastrophes and Miracles: A True Story of Love, Science, and Cancer. Seven people registered for session one and of those seven four people attended. Two people signed up for session two and both attended.

How Academic and Special Libraries Can Support Community Health Literacy Education Through Technology

Track: Health Equity & Global Health | On Demand

Lesley Farmer - Professor of Library Media, California State University Long Beach

For technology to play out its role effectively in community-based health literacy education (CHLE), community educators need to design instruction, curriculum, and programs that offer customizable learning and socially rich learning environments. Academic and special libraries can partner with community entities to provide the resources (including technology) and their ADA-aligned access, instructional design,

knowledge management, opportunities for community building and socializing, and venues for individuals to gain and practice health literacy. This session is based on a literature review of effective technology-enhanced community health literacy education (CHLE) efforts that involved academic and special libraries. Starting with UNESCO's interest in technology-enhanced community health literacy, this session identifies components for effective CHLE - and barriers that impede health literacy. Several aspects of CHLE are extrapolated from researched projects involving academic and special libraries: partnerships, education or community health workers, resources including technology, instructional strategies, policies, and assessments. Exemplar technology-enhanced CHLE with academic and special library engagement, are detailed.

In the Library with a Paintbrush: Artwork as a Pathway to Community Engagement and Wellness

Track: Professionalism & Leadership | On Demand

Gail Kouame - Director, Charles M. Baugh Biomedical Library, Mobile, Alabama

Lisa A. Ennis - Director of Library & Learning Resources, Alabama College of Osteopathic Medicine / Library & Learning Resources, Dothan, Alabama

Paula Webb - Outreach & Communications Librarian, Marx Library, Mobile, Alabama

Justin Robertson, MLIS - Head of Reference/Interlibrary Loan Coordinator/ Website Manager, Charles M. Baugh Biomedical Library

Background: The benefits of art-making, creativity, and interaction with the creative arts are well documented. Art is an avenue for self-expression and a way to facilitate dialog and engagement between the artist and the observer. It can also be used therapeutically in health care. Directors of two health sciences libraries have employed innovative approaches to integrate restorative practices utilizing art into their library spaces, and their lives outside the office as well.

Description: One academic health sciences library developed a new art gallery space hosting local artists on a rotating basis, employing practices based on well-established gallery spaces in another library on campus. The Outreach & Communications Librarian who operates these gallery spaces approached the new Director of the Biomedical Library and suggested installing an exhibition system at their location to enhance the physical space and increase engagement with library users and other members of the campus and surrounding communities. The Director enthusiastically agreed and initiated the development of the gallery space and planning group, building on the success of the library galleries program. The Director of a second health sciences library is an artist by avocation. She teaches art classes at a local museum. She also hosts sketching and painting events for faculty, staff, and students as part of her university’s overall wellness initiative. Additionally, she teaches a lecture about art therapy and how using an art therapist in clinical practice benefits patients. The class session includes an assignment where students engage in the creative process while monitoring their stress levels before and after creating something.

Conclusion: The new gallery space has been wildly successful. The space is booked through October of 2023 with a variety of exhibits including images from science, photography, painting, and textile arts. The physical space has been transformed from drab to fab. Receptions are held in conjunction with the exhibits, drawing in people from the campus and community at large. There are benefits of expressing oneself through the creative arts. Mindful sketching is a way to relax, slow down, work out problems and have fun. Students who attend guest lectures that include engaging with the creative process are initially skeptical, but most report that an assignment involving producing artwork does lower their stress level and is fun.

Is It Possible to Determine Citation Meaning with Machine Learning?

Track: Information Management | On Demand

Nicole Theis-Mahon, AHIP - Liaison Librarian & Collections Coordinator, University of Minnesota, Minneapolis, Minnesota

Caitlin Bakker, AHIP - Discovery Technologies Librarian, University of Regina, Regina, Saskatchewan, Canada

Sarah Jane Brown - College of Pharmacy and Medical School, Duluth Campus Librarian, Health Sciences Library, Minneapolis, Minnesota

Background: Citations do not always equal endorsement and it is important to understand their context. Researchers may rely heavily on a paper they cite, may refute it, or they may only mention it. Understanding why a paper is cited is more complex than determining the number of times it has been cited, and often requires readers to find and assess the citation within the full text. This classification could be valuable for researchers and users, but many faculty and students cannot devote the time to reading and classifying every article in order to have a sense of the landscape of the literature. The objective was to assess the accuracy of machine learning to classify a sample of citations.

Description: Scite is a “smart citation index” that utilizes machine learning to classify a citation’s meaning by rhetorical function (supporting, mentioning, contrasting, or unclassified). Our sample included 324 references in 286 publications. We conducted known item searching in scite and collected data into a Qualtrics form. We noted whether the item was included in scite, the citing publication’s meaning (supporting, mentioning, contrasting, or unclassified), the sentence(s) that included the citation, and a question asking if we agreed with scite’s assessment of the citation. Where we did not agree with scite’s assessment, we entered our own assessment of the citation’s meaning into the form. From these data, we calculated the precision, recall and accuracy to assess scite’s accuracy of classifying citation in our sample.

Conclusion: We found that scite’s overall accuracy was low, tending to underrepresent supporting and contrasting references. Despite this, scite shows promise in increasing the transparency of citations and has the potential to aid researchers and students by immediately displaying the broad landscape of the literature. It can be a valuable information literacy tool, highlighting for library patrons that a high number of citations is not a proxy for high quality research and instead encouraging more critical engagement with the literature.

It’s Complicated: Collaborating for National Institutes of Health Data Policy Changes Across an R1 Institution

Track: Information Management | On Demand

Kelsey Badger - Assistant Professor/Research Data Librarian, The Ohio State University, University Libraries, Columbus

Anna Biszaha - Assistant Professor/Research & Education Librarian, The Ohio State University Health Sciences Library, Columbus, Ohio

Stephanie J. Schulte - Professor/Assistant Director, Research & Education, The Ohio State University Health Sciences Library, Columbus, Ohio

Background: Though the 2023 NIH Data Management and Sharing Policy was announced two years ago, full scale preparations did not begin at an R1 Midwest university until the second half of 2022. The Office of Research Compliance approached the Libraries after identifying data management resources on a library

website. That serendipitous discovery by an office with substantial influence within the university soon blossomed into a working group with representation from nearly a dozen offices across the research support enterprise. This presentation will discuss the development of this partnership, highlight educational resources produced by the group, and share lessons learned about defining roles and responsibilities in a cross-functional collaboration.

Description: While few on the group had worked together regularly, the combination of librarians’ relationship-building expertise and the credibility afforded by the Office of Research Compliance grew this small partnership into a larger working group. Though the Office of Research Compliance convened the group, there was no designated leader. The group created agendas based on concerns brought by individual units and focused largely around event-planning. Additional members were added based on researcher questions, including intellectual property considerations and ethical sharing of human participant data. The range of campus units in the group has allowed us to pool our expertise and reach a wider audience with a unified message about the policy. The group first compiled information about existing materials and events to help our members stay up-to-date and minimize unnecessary duplication. Resource-sharing, internal referrals, and peer-to-peer education have continued to be important outcomes of the group. The Libraries took the lead in producing asynchronous educational resources and a highly successful informational webinar with over 200 participants. The group is currently planning a Q&A follow-up event featuring a panel of experts from multiple campus offices.

Conclusion: Early signs of success are evident in an increasing number of requests from departments that have not previously engaged with Library services. The service orientation of librarians can sometimes create tension due to variations in grant support across units, and working group representatives can differ in perception about the kind of assistance researchers would need and which campus units will provide that support. Additionally, the evolving group membership paired with a leadership void exacerbates the operational differences across units. Moving forward, capacity and scalability are ongoing barriers to the group’s shared goal of increasing researcher success. Despite challenges, the NIH policy opened dialogue and strengthened the reputation of librarians with research support units on campus and researchers.

Learning from Each Other: Results from the First Year of a Peer-Reviewed Literature Searching Project

Track: Information Services | On Demand

Heather J. Martin, MISt, AHIP - Director, System Library Services, Portland, Oregon

Carrie Grinstead, AHIP - Regional Medical Librarian, Shared Services, Burbank, California

Danielle N. Linden, AHIP - Program Manager, Providence St. Joseph Hospital, Orange, California

Background: Amidst shrinking staff and increased demand for services, a peer-learning program was developed to build literature search capacity and self-efficacy amongst staff at a large health system. A secondary objective of this program was to provide team-building opportunities for colleagues who may not work together directly. Participants included ten professional librarians and two library specialists working as part of a centralized department spread across seven western states with most staff working remotely or alone on site with few opportunities to meet in person. This project was first presented as a lightning talk at MLA ’22 and due to positive feedback from the audience is being presented here in greater depth with end of first year results.

Description: When a literature search showed no adequate search-self efficacy instruments, we designed a brief survey in REDCap to assess staff comfort with searching and with asking for colleagues’ help. This survey was administered before the project began and will be repeated when the project concludes in December 2022. Library staff are partnered and rotate monthly. Partners may be library specialists paired

with librarians, or librarians paired with each other. At the beginning of this project, experience levels ranged from one to twenty-seven years, and the rotating pairs allowed early-career staff to learn from senior librarians. Each librarian chooses a recent clinical question or other search request received from a patron. Partners independently perform each other’s searches, then meet virtually to compare results and strategies. The debriefing sessions are unstructured, and the unique features and challenges of each search drive discussion. Partners may share thoughts on database functionality, best practices in search strategy, or approaches to the reference interview and other communication with the patron.

Conclusion: Our pre- and post-survey asks staff to rate their confidence in searching on a scale of 1-5. In the pre-survey, librarians (9) reported that they were very confident (2), confident (5), or somewhat confident (2). Library specialists (2) were somewhat confident (1) and a little confident (2). The survey also includes two open-ended questions about facilitators and barriers to requesting help. The most common barrier reported was concern about imposing on colleagues’ time or adding to their workload; as our staffing has further decreased over the course of this project, we expect this concern to appear again in the post-survey. However, we also hope that the opportunity to talk with colleagues regularly will have increased familiarity and eased anxiety.

Learning from Medication Errors: Leveraging a Journal Scan Assignment to Teach Medication Safety

Track: Education | On Demand

Rachel Whitney, AHIP - Research & Education Informationist, MUSC Libraries, Charleston, South Carolina

Marc Lapointe, PharmD, BCPS - Medical University of South Carolina

Background: Medication Safety is an integral part of health sciences education that is not always provided enough dedicated time during the standard didactic curriculum. At our institution, medication safety is an elective course offered to second-year College of Pharmacy students (P2s). This elective is meant to supplement the ‘Just Culture’ concept in healthcare that is embraced by our university medical center. This elective is taught through a combination of hands-on assignments and guest lecturers sharing their real-world experiences along with the direct impacts of medication errors. This session will discuss how a journal scan assignment was structured to help reinforce the importance of medication safety.

Description: This medication safety elective presents a novel pedagogic approach for P2 students to become more aware of the consequences of medication errors prior to their objective structured clinical examinations (OSCE) as a P3 and Advanced Pharmacy Practice Experiences (APPE) as a P4 before graduation. The course coordinator invited the pharmacy liaison librarian to become integrated throughout the course and act as co-coordinator and to directly engage the students through the course’s journal scan assignment. The liaison librarian performs a search of the literature in PubMed to identify recent articles discussing medication errors. Students are then partnered in groups of two to review an article and create a 10-minute journal scan presentation reviewing their assigned article. In addition to this journal scan assignment, students complete an interview assignment with an experienced pharmacist or pharmacy technician. Feedback from the course is collected via a standard course evaluation which is provided at the end of the semester. In addition to the course evaluation, current and previous students are surveyed to evaluate their opinions on medication safety and the impact of the assignment during their OSCE, APPE, internship, and in the workplace.

Conclusion: This elective course presents a unique opportunity for the liaison librarian to become more involved in the pharmacy curriculum and directly engage with students. It also exposes students to a unique learning experience that allows them to use their literature evaluation skills acquired in the curriculum while delivering a presentation meant to engage the audience. We are expecting to: 1) measure whether the

journal scan and literature evaluation presentation are viewed as an effective way to learn about medication safety, and 2) determine whether this elective course impacts student opinions and practice on medication errors. Future steps include the potential to create a similar but interprofessional course with nursing, physician assistant, and medical students at our institution to strengthen collaboration between health sciences students.

Let’s Make a Deal: Gamification of Literature Searching

Track: Education | On Demand

Rachel Whitney, AHIP - Research & Education Informationist, MUSC Libraries, Charleston, South Carolina

Background: In the Doctor of Pharmacy program at our institution, first-year pharmacy students (P1s) are introduced to literature searching during the Introduction to Drug Information course. The liaison librarian embedded into this course found that students were struggling with feeling confident in their literature searching skills despite participating in active-learning activities. An area of opportunity was identified through the course evaluation feedback that students wanted more fun and engaging ways to practice literature searching. This paper will discuss one librarian’s experience with gamification, and provide two examples of gamified literature searching instruction sessions.

Description: Introduction to Drug Information is set up in a completely flipped classroom style, where students watch pre-class videos, and complete pre-class assignments before coming to an in-person 50-minute lecture. During these in-person lectures, active learning techniques are used to reinforce the skills taught in the pre-class videos. Literature searching is taught over a two-week period and is broken up into basic searching techniques and advanced searching techniques. Previously, the active learning strategy utilized was a handout that students needed to complete before being reviewed as a group. After reviewing the literature and conference abstracts for currently used techniques, two more interactive strategies were selected. A crossword puzzle was created for the basic literature searching instruction, and a virtual escape room was created for the advanced literature searching instruction. Students were given the option to work individually or in small groups. Initial feedback from the students has been positive, and formalized feedback will be collected at the conclusion of the course.

Conclusion: Overall the gamification of literature searching in this course was a success in getting students more engaged with the content. Student feedback after each session was positive, and a comparison of end-of-year evaluations showed that students enjoyed gamification, even going as far as to ask for more games and more group work. Students scored higher on their literature searching assignment after implementing these activities and the librarian received fewer emails asking for clarification or requesting to meet. Future steps include a research project to evaluate ways to incentivize student engagement with flipped content throughout the course.

Libraries and Data Ethics: Teaching a Data Ethics Course to a National Audience

Track: Professionalism & Leadership

Nicole Contaxis, MLIS, MA - Data Librarian and Lead of Data Discovery, NYU Health Sciences Library, New York, New York

Justin de la Cruz - Program Specialist, NYU Health Sciences Library, Asheville, North Carolina

Genevieve Milliken, MA, MSLIS - Data Services Librarian, NYU Health Sciences Library / Data Services, Wallkill, New York

Fred LaPolla - Research and Data Librarian, Lead Data Education, NYU Health Sciences Library/ Data Services, New York, New York

Background: As more libraries provide data services and analyze their own data on library collections and services, it is important for librarians to be informed on the possible ethical implications of how data is collected, stored, and disseminated. Using student feedback on a pilot course on data ethics for librarians, the authors expanded the pilot to include an additional class session and to accommodate a larger class size. Working with a national library organization, the authors ran the expanded class for a national audience.

Description: Before expanding the class, librarians reviewed student comments from the pilot course. Students requested more time for discussion as well as more information on implications in libraries specifically. The expanded class on data ethics for librarians included four classes: (1) An overview of data ethics and ethical decision-making; (2) Data collection; (3) Data communication, including data visualization; and (4) Data ethics and technology in libraries specifically. The fourth class was added to address student feedback. Each class included a guest speaker with research experience and expertise in a specific ethical area. Additionally, each class was designed to allow for significant student discussion on the lecture topic and on the guest speaker’s topic. The expanded class has been taught once, through a national library organization. The national library organization helped perform outreach to expand the course’s reach, with students from across the country from a wide variety of organizations.

Conclusion: Students were surveyed about the class after the expanded class was taught. Over 95% of respondents noted that the class was engaging, informative, and led by a knowledgeable instructor. Students did note that the classes could have used longer breaks and a slower pace to help students internalize the new information. The students appreciated the guest speakers, with many survey respondents writing in about specific speakers and their perspectives. Future iterations of the class will respond to these comments and integrate longer times for breaks and further student discussion.

A Library Sponsorship Program to Support Independent Student Research on High Performance Computers

Track: Innovation & Research Practice

Aida T. Miro-Herrans, PhD - Bioinformatics Librarian, University of Florida, Academic Consulting Research & Services (ARCS)
Hao Ye, PhD - Reproducibility Librarian, University of Florida, Academic Research Consulting & Services (ARCS)
Borui Zhang - AI/Natural Language Processing Specialist, University of Florida, Academic Consulting Research & Services
Joe Aufmuth, MS - Spatial Data Consultant, University of Florida, Academic Consulting Research & Services
Plato L. Smith II - Data Management Librarian, University of Florida, Academic Consulting Research & Services (ARCS), Gainesville, Florida
Natya Hans - Informatics Librarian, University of Florida, Academic Research Consulting and Services (ARCS), Florida
Daniel Maxwell, PhD, MLIS, MA - A.I. Trainer and Consultant, University of Florida, Research Computing
Michele R. Tennant, AHIP, FMLA - Interim Senior Director, University of Florida, Academic Research Consulting & Services, Gainesville, Florida

Background: The [Institution name] Libraries' research support services include consultations and research-related instruction in data analysis and data management for a variety of data types (e.g., biomedical, molecular,spatial, and text data). These data support librarians have technical expertise that allows them to instruct on methods and tools requiring the use of the university’s high performance computer (i.e., supercomputer). In 2019, in response to an increase in student requests for access to higher capacity computing resources, the Libraries partnered with campus Research Computing to provide high performance computer (HPC) access to students. Through this library-based program, students can perform independent research using the HPC resources and receive individualized technical and research support from the data librarians. This presentation describes the services provided.

Description: The [Institution name] houses the 3rd most powerful supercomputer among public universities in the U.S. University researchers can access these large scale computing activities through a fee-based service. This leaves students (undergraduate and graduate) who want to perform data analyses or have research experiences limited to working with researchers who have funding to pay for access to the HPC. The Library HPC Use Sponsorship Program, a collaboration between the library and campus Research Computing, was created so students could access HPC resources, regardless of their funding status (or that of their research advisers). Research Computing granted access to 200 computer cores for the Library to manage for student use. The HPC Use Sponsorship Program team is composed of librarians with expertise in bioinformatics, data management, geographic information systems, informatics, natural language processing, and reproducibility. Team members assess applications and determine the domain expertise required for the project. Students that are accepted into the program are paired with the librarian that can best support the project’s research needs and they work together through the life of the project. The librarians provide the students with both technical support to work on HPC and domain specific support.

Conclusion: Participation in the HPC Use Sponsorship Program has steadily increased over the last two years. However, the impact of the program is most noticeable in the projects and products that students have achieved, including capstone and thesis projects. This Program demonstrates how libraries can broaden access to research and can serve as a model for other institutions, particularly those looking to support students in data intensive research.

Looking Back and Forging Ahead with a New Systematic Review Service

Track: Innovation & Research Practice | On Demand

Sunny Chung - Health Sciences Librarian, Health Sciences Library, Stony Brook, New York

Laurel P. Scheinfeld, AHIP - Health Sciences Librarian, Health Sciences Library, Stony Brook, New York

Jessica A. Koos, AHIP - Interim Head of the Health Sciences Library, Stony Brook University, Stony Brook, New York

Jamie Saragossi - Interim Associate Dean of Collection Strategy and Management, Health Sciences Library, Stony Brook, New York

Background: The purpose of our systematic review task force is to create formal documents and policies, and streamline workflows so that any librarian can step in and continue the review process. Previously, there were no formal systems or policies in place. There has also been a high number of uncompleted reviews, and no triaging system. This systematic review service is provided by the Health Sciences Library to support an academic medical center.

Description: The documents developed include systematic review intake forms, a memorandum of understanding, protocol, and LibGuide that detail the estimated timeline for the systematic review to define

the roles and expectations for the entire team. Our team researched different types of free and paid models for project management software. Prior to the first meeting, research teams are encouraged to complete the intake form so that the librarians can determine who will take on the systematic review. After the initial meeting, a memorandum of understanding will be signed by the team leader and a librarian so that both parties understand their responsibilities before the search strategy is created. Next, the PI will need to complete the protocol template.

Evaluating new service, program, and initiative: Data regarding the progress of reviews with a health sciences librarian as a co-author will be examined in the future in order to gauge efficacy.

Conclusion: The updated formal systematic review service creates a clear understanding between the librarian and the research team. The internal workflow has been standardized as a result of this new triaging system and there is a better awareness of active systematic review projects amongst the librarian team. The implementation of a project management tool has allowed for better time management and allocation of resources to support the health sciences library. The systematic review task force will continue to use the new documents and policies that have been embedded into our workflow.

Making Inroads: Outreach to Pharmacy Residents

Track: Clinical Support | On Demand

Rachel Whitney, AHIP - Research & Education Informationist, MUSC Libraries, Charleston, South Carolina

Hilary M. Jasmin, MSIS - Assistant Professor, Research and Learning Services Librarian, Health Sciences Library, Memphis, Tennessee

Jason B. Reed - Associate Professor and Health Sciences Information Specialist, Purdue Libraries and School of Information Studies, West Lafayette, Indiana

Emily F. Gorman - Research and Education Librarian, Health Sciences and Human Services Library, University of Maryland, Baltimore, Baltimore, Maryland

Background: While many librarians find success embedding into the didactic curriculum, residencies can provide new challenges and opportunities, such as connecting with external preceptors and site coordinators; serving a unique body of researchers; and providing resources to users who are less traditional in the student-user sense. These factors can be compounded for pharmacy librarians, as pharmacy residencies are less common than medical ones – with around 67% of Doctor of Pharmacy (PharmD) students matching into residency nationally according to the American Society of Health-System Pharmacists (ASHP) in 2021. In this session, you will hear from four pharmacy librarians on their experiences with pharmacy resident programs, including initiatives for outreach, educational strategies employed, and collaborations with preceptors.

Description: While pharmacy residents are a unique subset of users, librarians can leverage the skills and connections they already have to build the foundation for a successful partnership with a residency program. Four pharmacy librarians will share what lessons they have learned, ideas for how to get involved with pharmacy residency programs, how to outreach to fourth year students (P4s) on rotations, and proposals for expanding involvement and assessing what these users need. Examples of these experiences include delivering an orientation to new residents that is specifically tailored to their resource needs, teaching topic development for research projects, and meeting with residents one on one to assist with their research. These efforts have been evaluated in various ways, including feedback surveys.

Conclusion: All four presenters have experienced some success getting involved with their residency programs, but there is still much that can be done. The experiences shared in this session aim to assist

others who are looking to get involved with their own residency programs. Future goals of these pharmacy librarians involve a multi-site needs assessment of residency programs, research into formalized learning objects that cater to residents, and potentially pharmacy resident focus groups.

Making the BRAIN Initiative FAIRer

Track: Information Management | On Demand

Alisa Surkis - Deputy Director/Vice Chair for Research, NYU Health Sciences Library, New York, New York

Peter Petersen, PhD - Assistant Professor, University of Copenhagen

Rodrigo Amaducci, PhD - Postdoctoral Researcher, University of Copenhagen

Background: With the advent of the new NIH Data Management and Sharing requirements, institutions are turning to libraries to provide increased data sharing support for their research community. Within neuroscience research, the landscape around data sharing has been developing through the support of the NIH BRAIN Initiative, which in 2017 began funding the development of data archives and standards emerging from the community. A key mechanism for dissemination of these resources were large collaborative grants supporting the study of brain circuits underlying behavior for which a data science core was a required component. However, the bottom up approach to the development of archives and standards left gaps in coverage that could make adherence to the top down mandated FAIR principles difficult.

Description: Insufficient or uninterpretable metadata results in data that cannot be reused. This issue was a primary concern for both the data science core director of one of the collaborative circuit function grants, a librarian, and a postdoctoral researcher in one of the collaborating labs, an experimental neuroscientist who collected behavioral and electrophysiological data. The postdoc had piloted a tool to facilitate the collection of structured, standardized metadata in his lab, and, as part of the work of the data science core, the postdoc and the librarian collaborated on the further development and dissemination of the tool. One of the biggest challenges in capturing experimental metadata is that the collection of complete and understandable metadata during an experiment is very often an unwelcome burden, therefore central to the development of the metadata collection tool was a recognition that if it required a steep learning curve and/or significant disruption of experimental workflows, widespread adoption would be very difficult to achieve. Additional support for tool development and dissemination from a grant supplement awarded by NIH allowed for retaining an outside development firm with expertise in both front and backend development and user experience to maximize the functionality and usability of this tool.

Conclusion: While the ultimate measure of success of the metadata collection tool will be the number of labs in which it is adopted, there will be a number of points of evaluation over the course of tool development. The first milestone will be, through a process of usability testing and continuous quality improvement, achievement of satisfaction of users with the tool within the context of usability testing. During early dissemination efforts, visits to the website will indicate that a broader community is becoming aware of the tool. The number of registered users will be a further indication of interest. Finally, a reflection of success in these early stages will be the number of labs/researchers willing to serve as beta testers.

Measuring the Impact of a Research Impact Challenge

Track: Innovation & Research Practice | On Demand

Samantha Wilairat - Research Communications Librarian, Lane Medical Library, San Jose

Amanda Woodward, MLIS - Web Services Librarian, Lane Medical Library, Stanford, California

Sonam Soni, PharmD, MLIS - Resource Management Librarian, Lane Medical Library, Stanford, California

Kaitlynn Ryan Stinson - Access Services Specialist, Lane Medical Library, Stanford, California

Background: In September 2022, the Lane Medical Library hosted its 4th annual Research Impact Challenge (RIC). The 2022 RIC was designed to accomplish the following goals: 1) Align with the institution’s Open Access Policy, 2) Increase the adoption and integration of ORCID identifiers on the medical campus, 3) Connect patrons with bibliometric-related tools that the library subscribes to in order to help patrons gain a better understanding of their personal research metrics, 4) Establish the medical library as the primary point of contact for research impact-related questions on campus, and lastly, 5) Increase the visibility of the medical library as a resource for medical campus community members.

Description: As research impact and bibliometric services gain popularity within medical libraries, RIC events are becoming more common. There is limited information published about the impact that these events have on medical library patrons. For this reason, the Marketing and Communications Team (MCT) and the Research Communications Librarian, developed several methods to better assess the participation and engagement of RIC registrants, and the impact the RIC has on the Stanford medical campus with the hope of sharing their findings with the medical librarian community. The MCT is composed of three librarians across three different departments. MCT promoted the challenge via targeted emails to past challenge participants, newsletter subscribers and liaison contacts. We shared the challenge announcement on our library blog and social media. In 2022, the RIC consisted of five daily emails with suggested challenges scaffolded at three different experience levels for participants to complete independently. A raffle prize element was added for the first time to the RIC, which was used as a marketing strategy to advertise the event. Data sources covered in this presentation include: Mailchimp activity, prize raffle survey data, institutional ORCID integration statistics, Libguide statistics, qualitative feedback from RIC raffle winners, and post event evaluation survey responses.

Conclusion: We expect to report on the impact the RIC had on the Stanford Medicine community based on the data sources mentioned above. Historical data available from previous iterations of the event will be used as a benchmark to monitor progress. Interest and engagement with the event disaggregated by departmental groups and patron type will be shared. The five RIC goals used to help focus the event will be reflected upon. The presenters will share whether the added element of a raffle prize was a successful incentive to the Stanford Medicine community and will share marketing and event evaluation tips with attendees who wish to plan a similar event.

Mediating Copyright Permissions for Integrating Questionnaires and Scales into the Electronic Medical Record in an Academic Health Sciences Center

Track: Information Services | On Demand

Katherine Hoffman - Public Services Specialist, The Ohio State University Health Sciences Library, Columbus, Ohio

Stephanie J. Schulte - Professor/Assistant Director, Research & Education, The Ohio State University Health Sciences Library, Columbus, Ohio

Background: An academic health sciences library (AHSL) serving a large academic medical center launched a service focused on providing information about copyright and obtaining copyright permissions. Requestors were expected to make decisions about fair use and their ability to comply with copyright license terms and handle all correspondence when requesting permissions from the copyright holder. Over time, the AHSL saw an increase in requests for assistance obtaining permissions to integrate published

questionnaires and scales within the electronic medical record system (EMR). The complexity of these requests and the potential extent of use pushed the boundaries of fair use and, possibly, state and university policies. The AHSL staff felt compelled to investigate a formal contractual oversight process for these requests.

Description: AHSL staff took their concerns to the medical center’s legal affairs and also worked with the university’s intellectual property (IP) legal specialist, resulting in approved permissions templates and contract review processes that will be detailed in this presentation. The ultimate goal was to protect the university, which was counterintuitive to staff more accustomed to discussing exceptions to copyright in academic situations. It was also a cultural shift for medical faculty who have the authority to make determinations of fair use for their own teaching and scholarly work, even with limited knowledge and personal legal counsel. The first several months of this program have highlighted issues that other libraries can learn from. One of these biggest lessons learned is that restricting access in ways a typical copyright permissions agreement might aim to do is much more complex at the scale of an EMR. Other issues include clinicians’ attitudes and expectations about the speed of working with institutional IP officers when copyright is perhaps lower priority than other IP endeavors. Practical issues for library staff include the kind of expertise that must be developed, fair compensation for staff working in this realm, and decisions about funding permissions now and in the future.

Conclusion: The use of questionnaires and scales in the EMR for clinical care is increasingly widespread; given the potential scope of this use, it is likely that neither the requesting clinical users, nor AHSLs – even those with a copyright librarian with a JD – have the authority to independently negotiate and sign copyright license agreements. Formal processes are sometimes needed even when institutions want to make the most of copyright law. However, while clinical and health systems informatics faculty and staff may lack knowledge of copyright law and policies, institutional intellectual property officers may lack familiarity with the scope and needs of clinical users. The AHSL can serve as a bridge between these groups to facilitate the copyright permissions process.

A New Normal: Pivoting Strategies for Technology Engagement in Libraries

Track: Education | On Demand

Brandon Patterson - Technology Engagement Librarian, Eccles Health Sciences Library, Salt Lake City, Utah
Bryan E. Hull - Head, Digital Publishing, Eccles Health Sciences Library, University of Utah, Salt Lake City, Utah
Mark Howell - XR Design Engineer, Eccles Health Sciences Library, Utah

Background: Educational technologies that were once synonymous with hands-on learning and maker communities are finding new uses in a post-COVID setting. Before the pandemic, a 3D printing service required thumb drives, filament replacement and in-person assistance for successful prints. A multimedia, audio and video creation space focused on easy “one button” solutions to video creation. A VR symposium hosted in-person demonstrations and speakers.

Description: Based on user feedback and usage data, a team of library staff and faculty reworked technology “hubs” to meet the adjusted demands of patrons after returning to campus. A 3D printing service that was once hands-on has been fully automated through an online submission platform. A multimedia, audio and video creation space has been replaced with audio equipment for checkout as a result of heightened user demand for podcasting. Simulations in VR have pivoted to largely monitor-based or headsets available via a checkout service. Monitors for hybrid meetings and study spaces are used in abundance. Guidance to rework similar spaces in other libraries will be given, as well as do’s and don’ts for

ways to think of technology in a new learning environment.

Conclusion: The 3D printing service is seeing greater use and partnerships with campus simulation centers and research labs has greatly benefitted from an automated service. While the multimedia studio has seen an overall decrease in the number of sessions, the number of sessions for podcasting became the vast majority of the uses.There has also been heightened collaboration with campus units using VR technologies with direct involvement in courses for surgery, dentistry, nursing and biology.

Pathways of Engagement for an R2 Campus with the All of Us Researcher Workbench

Track: Health Equity & Global Health

Lisa A. Connor - Assistant Professor, Research & Instruction Health Sciences, Sam Houston State University, Huntsville, Texas

Background: A novel way to engage faculty and students and promote the use of large data sets supporting innovation and research practice, and health equity, will be described. The All of Us Research program is an ambitious and historic endeavor that will vastly change healthcare in many ways for both patients and clinicians. The program aims to gather unique data providing opportunities for research at the intersection of biology, environment, and lifestyle from one million or more people in the United States. More than 80 percent of current participants are historically underrepresented in biomedical research. The data provided from program participants provides billions of data points to conduct interdisciplinary research by accessing the All of Us Researcher Workbench.

Description: Becoming a Carnegie Classified R2 – Doctoral Research University with High Research Activity and recently earning the federal designation of Hispanic Serving Institution (HSI), a health science librarian was inspired to initiate an institutional agreement and develop a program providing training and support of institutional engagement for the All of Us Researcher Workbench. The available data is diverse and longitudinal and offers, for example, physical measurements, electronic health records data, Fitbit records, survey data, and whole genome sequences. The abundant data represents an opportunity to engage in biomedical research and new ways for librarians to support research and scholarship. The presentation will discuss working with the Office of Research and Sponsored Projects to create a pathway for any individual affiliated with the institution to pursue research topics of interest. Efforts to raise awareness across campus that the researcher workbench is accessible will be described along with directing individuals to follow protocols to become a registered user and agree to a data user code of conduct. Ways to collaborate across disciplines supporting interprofessional education and research initiatives will be reviewed. Library guides developed and educational programs created will be demonstrated. Assessment of the program efforts will be provided.

Conclusion: Preliminary informal conversations with faculty about the researcher workbench have demonstrated the need and desire to gain access to this data in support of undergraduate, graduate, and faculty research projects. At the present time the program has not concluded, but quantitative data on the program progress can be readily measured and presented by the number of researchers at this institution choosing to become a registered researcher of the workbench by the time of MLA 2023. The presenter intends to make this program a formal research project and hopes to provide more detail on development of a research protocol if selected and approved for this presentation.

Putting the Fun in Fundamentals: Developing a New Course for Health Sciences Research Data Management

Track: Education | On Demand

Justin de la Cruz - Program Specialist, NYU Health Sciences Library, Asheville, North Carolina

Peace Ossom-Williamson - Associate Director, NNLM National Center for Data Services, Fort Worth, Texas

Alisa Surkis - Deputy Director/Vice Chair for Research, NYU Health Sciences Library, New York, New York

Genevieve Milliken, MA, MSLIS - Data Services Librarian, NYU Health Sciences Library / Data Services, Wallkill, New York

Nicole Contaxis, MLIS, MA - Data Librarian and Lead of Data Discovery, NYU Health Sciences Library, New York, New York

Fred LaPolla - Research and Data Librarian, Lead Data Education, NYU Health Sciences Library/ Data Services, New York, New York

Background: Research data management (RDM) is an essential component of data services in health sciences libraries, yet there are scant opportunities to engage with learning and practicing fundamental RDM skills. With more demand for data curation, management, documentation, sharing, and publisher and funder requirements, such as the National Institutes of Health’s Data Management and Sharing Policy, it is clear that education in these areas is essential. Accordingly, there is a growing need for medical librarians to learn the skills needed for a changing data ecosystem centered on data support, policy compliance, sharing, and transparency. This presentation focuses on the development of a new eight-week class for practicing medical librarians interested in learning more about research data management.

Description: A team of data services librarians developed a new eight-week course to teach fundamental concepts in health sciences research data management. The team determined which topics to cover based on their experiences working in research data management and reviewed existing materials to select readings, tutorials, videos, and other resources for the class. They also created discussion prompts and innovative assignments that provided learners with the opportunity to work on common tasks in this area of work, including reviewing an existing data management plan and critiquing a clinical survey instrument’s approach to data collection. One major focus was encouraging learners to connect with their local communities, with one assignment asking them to interview a researcher about their data needs. The class covered a wide range of topics, from the research data lifecycle through reproducibility, open science, data documentation, sharing, and more. Throughout, learners had assistance from course facilitators as well as “community experts,” who are established data services librarians who advised learners on conducting a data interview and producing a final presentation about a research data management topic of their choice. Evaluation included surveys with written feedback from learners, facilitators, and community experts.

Conclusion: More than 100 people applied, 60 were selected, and 44 completed the eight-week course. This shows a strong demand for learning in this area. Learners interacted a great deal during the course, showing a high level of engagement on the course’s discussion boards. Learner feedback was exceptionally positive — some learners are already using materials from the course in their work. The majority “strongly agreed” that the course was “engaging” and “informative.” One response said, “I am getting ready to be the data management lead for my library and the readings, the very relevant homework assignments, the discussions, and prepping for the final project presentation all were very beneficial to me.” Feedback will be considered in future classes.

The Redesigned Core Clinical Journals Filter: What It Is, How to Use It, and Why to Use It

Track: Clinical Support | On Demand

Michele Klein-Fedyshin, AHIP, FMLA - Research and Clinical Instruction Librarian, University of Pittsburgh/Health Sciences Library System, Pittsburgh, Pennsylvania

Background: The Medical Library Association convened a committee at the National Library of Medicine’s request to review PubMed’s Core Clinical Journal (CCJ) Filter. The committee reviewed both the subject coverage of the list for the first time and the journals included on it using real world data from actual clinical journal usage (not subscriptions), clinical diagnoses and frequent medical topics. The resulting list covers more health professions, contemporary clinical concerns, and new clinical disciplines. The methodology and resulting list in article form were submitted to the Journal of the Medical Library Association (JMLA). This paper describes how to implement the new data-driven filter in health sciences libraries and the enhancements to service it offers, delving beyond the scope of the article.

Description: The new CCJ filter, tentatively renamed Clinically Useful Journals, includes 241 titles representing 80 subjects. Thirty-three of the subjects are new. The objectives are to maximize the subject coverage as it relates to an individual institution and to describe creation of a search string for the alphabetical list of titles. A complete subject index of the covered journals is an appendix of the JMLA article. Its formation, benefits and potential usage will be discussed. In addition, a PubMed strategy to create a hedge using field codes for the alphabetical list will be described. Customization for both the subject and alphabetical strings are possible. The subject index facilitates use of the list for collection choices. How the subjects were selected is explained to learn where to find sub-concepts within the broad MeSH subject terms. Also, where to find the actual rank of a particular journal within a particular subject will be presented. A clinical filter is missing from PubMed presently, and this redesigned filter can be deployed individually using the techniques covered here.

Conclusion: The new CCJ/Clinically Useful Journals filter is more reflective of real-world clinical concerns incorporating journals more representative of actual clinical usage. Professions beyond medicine are covered, such as nursing, psychology and addiction. Anticipated outcomes from using the innovative filter are faster identification of clinically relevant articles by librarians and clinicians. Search efficiency and shorter turnaround to finding the journal article you need can be based on actual clinical usage counts. A filter of those journals used most clinically will benefit patient care and education. It is like adding a clinical impact factor to your searches and collections. The innovative, data-driven methods redefined the process and product, focusing on clinical impact. Here’s how to make it work for your institution.

Reflecting on Working-From-Home while Forging Ahead to the Hybrid Library Workplace of the Future

Track: Professionalism & Leadership | On Demand

Kathy Downing - Digital Services Librarian, NNLM Public Health Digital Library, Worcester, Massachusetts

Becky Baltich Nelson - Education & Clinical Services Librarian, Lamar Soutter Library, UMass Chan Medical School

Catherine Carr, MLIS - Education & Clinical Services Librarian, Lamar Soutter Library, UMass Chan Medical School

Morgan Kolinski - Project Manager, Lamar Soutter Library, UMass Chan Medical School, Worcester, Massachusetts

Robert Vander Hart - Electronic Resources Librarian, Lamar Soutter Library, UMass Chan Medical School, Massachusetts

Tess Grynoch - Research Data & Scholarly Communications Librarian, Lamar Soutter Library, UMass Chan Medical School

Background: In March 2020, the Library closed due to the COVID-19 pandemic, and all Library employees began working remotely. The Library reopened with restrictions and partial staffing in August 2020. To smooth the transition from a completely remote work environment to a long-term hybrid arrangement, a Hybrid Working Environment Team, composed of non-managerial staff members, was formed. The Team was charged with identifying the various work models being utilized in libraries and beyond, the pros and cons of each model, and making recommendations along with considerations for implementation. Additionally, the Team investigated the physical space/office requirements for each model. The Team also investigated issues of visibility, team cohesiveness, Library culture, productivity, home office requirements, and Library in-person staffing levels.

Description: After completing a literature review, staff survey, and review of available Library statistics and functions pre- and post- March 2020, the Team proposed a Service-First Hybrid Model. The Service-First Hybrid Model prioritizes providing services at the point of need for the Library’s various communities while balancing the benefits of onsite and offsite work for each individual staff member. Rather than a blanket requirement for all Library staff, the Team recommended that the number of days onsite be determined based on the role and duties of each staff member regardless of department. The findings from the survey and literature review also stressed the importance of deliberate strategies to build and maintain team cohesiveness through setting time for work-related discussions and non-work social engagement. The Team also put forward recommendations on technology required for hybrid work, developing hybrid work guidelines, onboarding, managing a hybrid team, communication, and increasing library visibility to the Library’s various communities. The Team also recommended a plan for assessment of the hybrid work model on a semi-annual basis.

Conclusion: After receiving the Hybrid Working Environment Team’s final report, the Library’s management team was faced with the difficult task of deciding how to implement the recommendations while finding an equitable solution for all employees and responding to University administration. This report has informed our plans for the renovation of the Library as well as other initiatives, including the new hybrid staffing model that was implemented in November 2022, the creation of a new onboarding document, regular group lunches with the Library director, and the distribution of the Library’s marketing plan.

A Sensory Room Pop-Up: Supporting Student Wellness in the Library

Track: Professionalism & Leadership | On Demand

Brian Maass - Digital Technologies Librarian, University of Nebraska Medical Center, McGoogan Health Sciences Library, Omaha, Nebraska

Hannah Nelson - Student Senator, UNMC College of Medicine, Omaha, Nebraska

Emily J. Glenn, AHIP - Interim Dean, McGoogan Health Sciences Library, Omaha, Nebraska

Background: In 2022, McGoogan Health Sciences Library at the University of Nebraska Medical Center launched a sensory room pop-up experience. A student senate representative proposed that the library create a sensory environment for students to explore and integrate the use of their senses to advance their education and increase well-being. The objective of the room was to provide space to explore all five senses

as an integral part of students’ educational journey. In creating a customizable room, students could choose to reduce stress, improve focus, or take time for a “brain break.” While sensory rooms may be present in K-12 schools, clinical settings, and public libraries, there was little evidence of sensory rooms in academic medical center libraries or other higher education student-focused spaces.

Description: The student’s proposal outlined goals for a sensory room pop-up that included background research and a detailed mood board that provided examples of items, activities, and lights to convey a “feeling” for the space. A team of library staff considered the proposal alongside library space constraints, budget availability, and facility rules. Once the project plan was approved, the team worked with the student to launch a summer pilot and two fall pop-up sessions, each lasting five days. In preparation, a scale model was created to visualize how to best use the chosen classroom. Due to the large room size, the space was divided into smaller sections to offer multiple experiences. Areas included a semi-enclosed private tent with bean-bag pillows, small projection lights and battery-operated candles (sight), a kinetic sand play space as well as craft tables and fidget toys (touch), a video and music relaxation nook (sound), a snack bar (taste), and takeaway scented items (smell). Overhead lighting was also adjustable to allow users to further customize their experience at any chosen station. Completed artwork was displayed in the entrance and exit walkway. Anonymous assessments were passively gathered via a paper form.

Conclusion: The summer pilot permitted the team to assess the set-up with a smaller invited group of testers and students. The planning team reviewed feedback from the pilot to prepare for the fall pop-ups. The first fall pop-up created significant buzz across campus, including requests for a permanent sensory room in the library. For the second fall pop-up, we partnered with occupational therapy students to create “did you know” messages to promote engagement with learning about sensory experiences. We plan to consider all feedback and talk with campus partners to determine the best way to include sensory experiences within the library.

StudyWell at HSL: Forging Campus Partnerships to Foster Student Wellness

Track: Information Services

Emily J. Hurst - Deputy Director, Head of Research and Education, VCU Libraries, Health Sciences Library, Richmond, Virginia

Background: When a large, urban, academic research library and the related health sciences library reopened in 2021 from pandemic closures some students reported feelings of fatigue and inability to reconnect effectively due to their recent online-only learning environment. Library events staff collaborated with service and program leaders at both libraries to brainstorm appropriate measures the libraries could take to address student health and wellness needs. By forging partnerships with Student Affairs leaders across the university unique programming opportunities were developed that would allow students to use library spaces in new ways.

Description: With the library as lead, the StudyWell program was created and programs were designed to meet the wellness needs of the unique student bodies at both campus libraries. Looking closely into library resources programs were created to both provide wellness breaks as well as introduce students to services offered at the library or on campus. This presentation will focus on StudyWell at HSL and the lessons learned when developing campus partnerships and providing services for students on a health sciences campus as compared to those on a more traditional arts, humanities, and science campus. Emphasis will be given to the development of relationships with Student Service leaders to sustain programming throughout the year as well as the evolution of StudyWell from the conceptualized semester-long offering to nearly two years of successful engagement.

Conclusion: Review of data from the first one and half years of StudyWell shows a continued increase in programming and events at both libraries. Increase in attendance at events as well as support from library leadership has provided additional support for the continuation of the program. This presentation will include analysis of response to StudyWell at HSL events. Qualitative feedback from students as well as Student Affairs leaders will be shared.

Taking Action to Reduce Health Misinformation: Training Medical Learners How to Disseminate Information to the Consumer

Track: Health Equity & Global Health | On Demand

Ann Harris, AHIP - Senior Informationist & Medical Librarian Team Leader, Assistant Professor, Western Michigan University Homer Stryker M.D. School of Medicine, Kalamazoo, Michigan

Juli McCarroll, AHIP - Informationist/Medical Librarian & Assistant Professor, Western Michigan University Homer Stryker MD School of Medicine Medical Library, Kalamazoo, Michigan

Elizabeth R. Lorbeer, AHIP - Chair, Medical Library, Professor & Library Director, Western Michigan University Homer Stryker M.D. School of Medicine, Kalamazoo, Michigan

Background: Distrust of the U.S. healthcare system by the general population in the United States remains high, leading to poor health outcomes and misinformation. In a 2006 study published in the Journal of General Internal Medicine, the amount of distrust is higher for those who lack health insurance coverage between the ages of 31-60 and reported poorer health. Since the early 1970s, health misinformation has increasingly been reported, reaching its first peak in 1995 and again in 2020, and it continues to grow. To combat misinformation, the faculty librarians created health literacy courses to teach trainees how to find high-quality evidence in English and Spanish, rewrite scientific articles into plain-language summaries for public websites, and practice teach-back.

Description: The faculty librarians teach electives in the foundations of personal and organizational health literacy to train medical learners to search the evidence-based literature and author online plain-language summaries. It allows medical learners to read studies primarily funded by U.S. taxpayers, synthesize scientific discoveries, and write lay summaries for health consumers to read online. By incorporating elements of evidence-based medicine, these electives also reinforce the students' ability to appraise and summarize scientific studies critically and rapidly disseminate knowledge to provide synthesized information from peer-reviewed and authoritative sources. Most learners need help writing plain-language summaries. The electives bring to light how to communicate as clearly and simply as possible to improve the general population's understanding of science, improve outcomes for eliminating health disparities, participate in a clinical trial, and justify spending on research. Furthermore, teaching students to get in the habit of writing plain-language summaries of their research helps reduce misinformation for papers published in Open Access journals. Finally, providing opportunities to practice health literacy supports the longitudinal learning of the trainee actively involved in the community healthcare setting as part of their clinical training.

Conclusion: Health misinformation harms the welfare of our citizens, as some already find recognized authorities untrustworthy and choose not to accept scientific discovery and sound medical knowledge from these sources. In addition, it may only sometimes be possible to change a patient's opinion during face-to-face care. Yet, another approach is authoring online sources of information that consumers use to look up medical conditions. Training future doctors on how to write plain-language summaries of published studies and their research, plus where to deposit content on public sites that consumers use, will assist in curbing health misinformation.

Telehealth in Libraries: An Opportunity for Community Service

Track: Health Equity & Global Health | On Demand

George G. Strawley - Library Engagement Specialist, Network of the National Library of Medicine, Salt Lake City, Utah

Darlene Kaskie - Community Engagement Coordinator, Network of the National Library of Medicine, Iowa City, Iowa

Faith Steele, AHIP - Outreach and Education Librarian, Network of the National Library of Medicine

Abby Dowd - Instructional Designer, Eccles Health Sciences Library, University of Utah / NNLM NTO

Katie Pierce-Farrier - Data Science Strategist, University of North Texas Health Science Center, NNLM Region 3, Fort WOrth, Texas

Background: As the COVID era demonstrated, telehealth holds promise for increasing access to and reducing costs of healthcare. However, many people lack broadband access, private space or digital literacy skills necessary to benefit from telehealth. Libraries are well positioned to step into the gap and provide telehealth access for communities encountering such challenges. Health sciences libraries in particular can support their public library and community-organization partners in connecting to and promoting telehealth services. This session will present models for library access to telehealth services used around the country and offer resources, training and networking opportunities available from state, regional and national organizations to prepare staff for supporting telehealth, including courses that can help with making decisions and serving as digital navigators.

Description: In Spring 2023, staff members from the Network of the National Library of Medicine expect to launch a free continuing education course geared toward library administrators and staff that offers an overview of important aspects related to the provision of telehealth services in libraries. The asynchronous course will teach learners to recognize the different approaches and models used in providing telehealth access within libraries, describe resources available to libraries for telehealth technology, discuss the role of the library in support of digital skills training, and address potential ethical and legal concerns that may be on the minds of libraries interested in telehealth.

Conclusion: Full evaluation of the course is not expected to be complete by the time this session is presented. However, the authors will be able to describe resources and opportunities for audience members and share comments from test users of the course. Outcomes of the course over time are expected to include increased understanding of telehealth as it applies to libraries, development of new skills related to assessing opportunities for service in this area, and clearer discernment of whether and how telehealth access is suitable to the participant’s own library.

Translation of Systematic Review LibGuide Content Using Plain Language and Scientific Writing Best Practices

Track: Information Services | On Demand

Elizabeth Moreton - Clinical Nursing Librarian, UNC Health Sciences Library, Chapel Hill, North Carolina

Terri Ottosen, AHIP - Community Engagement and Health Literacy Librarian, Health Sciences Library, University of North Carolina, Chapel Hill, Chapel Hill, North Carolina

Hannah Burrows - Community Outreach and Global Health Librarian, UNC Health Sciences Library, Chapel Hill, North Carolina

Sophie Nachman - Graduate Assistant, UNC Health Sciences Library, Chapel Hill, North Carolina

Luke Barron, BBA - Graduate Assistant, UNC Health Sciences Library, Chapel Hill, North Carolina

Emily Jones, AHIP - Health Sciences Librarian, UNC Health Sciences Library, Chapel Hill, North Carolina

Background: Plain language summaries (PLS) and scientific communication best practices focus on style and delivery of content, as they translate difficult and complicated topics into engaging and easy to understand material, often intended for members of the public or non-specialists. Systematic reviews are increasingly undertaken by novice reviewers as “easy publications” or even as class assignments. However, the process of conducting a systematic review remains complex and sensitive to the authors’ interpretation, knowledge, and skillset. Many libraries serve as the institutional point-of-contact for systematic review instruction and training. This paper describes the incorporation of plain language and scientific communication best practices to supplement and simplify complex language and processes described on our highly utilized Systematic Review LibGuide.

Description: Librarians with experience conducting and teaching systematic reviews revised our Systematic Review LibGuide in several iterations between 2014-2022, yet a recent review of guide content revealed a large amount of technical information and long lists of resources despite the guide’s intended audience of students or novice researchers. Mid-2022, two health sciences librarians streamlined the guide and arranged the content in a stepwise manner to better aid beginner researchers. Then, they partnered with two other librarians, one with a background in patient education and plain language summaries and another with a background in scientific and technical writing, who rewrote text from the most used pages of the systematic review guide to apply best practices in plain language and scientific and technical writing respectively. As a group, the librarians then created a plain language version of the existing guide pages by consolidating and combining their revisions together. **Conclusion**: Guide content will be evaluated pre- and post- edit using well-known metrics, such as readability (Flesch-Kincaid reading grade level), understandability, and actionability (Patient Education Materials Assessment Tool). Although many changes to the guide consisted of small adjustments that did not allow for user-related metrics to be gathered, the improvement process is relatively straightforward and is expected to provide additional clarity for diverse users. Our team plans to continue updating the language and organization of the Systematic Review guide and to apply the same critical eye to our other instructional guides.

Using Monthly Book Lists to Showcase Library Resources with an Emphasis on Work-Life Balance, Marginalized Communities and Patient Care

Track: Information Services | On Demand

Chloe Hough, AHIP - Reference Librarian, Tampa Bay Regional Campus Library, Florida

T. Brandon Hall - Circulation Supervisor, Tampa Bay Regional Campus Library, Clearwater, Florida

Background: When [institution] opened in 2019, the small library staff sought ways to build an inclusive and engaging library culture. The Circulation Supervisor developed the practice of creating monthly reading lists to showcase the wider library system’s holdings, draw attention to important topics, and encourage student interaction with the library. Over the past few years, this passive programming project has developed into a larger collaboration between the Circulation and Reference departments, as well as created a robust workflow among the various full-time, part-time, and temp employees. Using a three-prong marketing approach (a LibGuide, library social media, and physical displays), the booklists promote work-life balance to create well-rounded healthcare students and provide diverse perspectives on patient care.

Description: Each month, two themed reading lists are created: Take A Second, which promotes work-life balance for busy students, and Topics in Healthcare, which focuses on an educational health-related topic. The reading lists are presented both virtually through a LibGuide, and physically via displays at the library desk. The LibGuide features full lists of all the selected materials in multiple formats with summaries and links to check out the items. The physical displays feature paper crafted imagery and printed book covers with QR codes to the books’ catalog entries. Each month, the Circulation and Reference staff collaborate to develop topics for both lists; these topics are often based on monthly commemorations, such as Black History Month and Traumatic Brain Injury Awareness Month. The topics chosen aim to bring awareness to important issues and uplift marginalized voices. A list of 5-10 books for each topic is then selected- Take a Second by a full-time circulation staff member, and Topics in Health Care by one of the two Reference Librarians. After lists are curated, additional tasks are split up amongst the rest of the circulation staff, including pulling quotes, developing descriptions, and updating the LibGuide and physical displays.

Conclusion: This monthly project has not only facilitated engagement with the library’s student patrons but has also created an ongoing workflow within the library’s Circulation department and allowed for collaboration between the Circulation and Reference teams. Since instituting the monthly booklists, many students have checked out items from the library catalog, increasing both usage of library materials and discussion around highlighted topics. The library’s book display on Hispanic Heritage Month was recently used at a campus-wide event, opening the door to future partnerships within the [university] community.

Using ThingLink as an Educational Roadmap for Evidence Syntheses Skill Building

Track: Education | On Demand

Caitlin McClurg - Associate Librarian, Libraries & Cultural Resources, University of Calgary, Calgary
Bronte Chiang, BA, MLIS - Assistant Librarian, Libraries & Cultural Resources, University of Calgary, Calgary, Alberta, Canada

Background: The Covid-19 pandemic continues to be a disruptive event for the Canadian healthcare sector, and impacts can be felt throughout post-secondary health programs and by extension, the libraries that collaborate with them. With staff and learner absences and jobsite access restrictions, the authors saw a notable uptick in requests for fulsome systematic and scoping review assistance as clinical psychology graduate students, undergraduate honors students and medical learners did more work outside of the clinic for their research output over the past three years.

Description: The authors will describe how the swiftly changing library staff complement, shifting library priorities and increased intake volume prompted a new way of providing support for post-secondary learners undertaking knowledge syntheses. Notably, authors moved away from traditionally long-form library-oriented instruction, including hosting instructional workshops and creating libguides to the provision of self-paced modules and interactive learning objects that encourages learner independence while balancing the realities of the limited resources of time, staffing, and threshold concepts for library and information science skills. ThingLink was primarily used as a software program for virtual tours at the authors’ institution, and a reimagining of the tool from a tour-based application to one of interactive learning is a novel and first-use example of this product at the organization. The ultimate goal of the ThingLink learning objects was to provide a sustainable offering to meet patron requests for support engaging with knowledge synthesis processes and skills development. The session will reference adult learning theories such as the opportunities and drawbacks of workplace learning knowledge translation, application and retention.

Conclusion: The program will describe the development of learning objectives, discuss the growing pains of the philosophy of service and role of librarians in knowledge syntheses, and identify the areas of opportunities for continued presence in knowledge synthesis output at the institution. Comparative metrics

will be shared with attendees including some descriptive statistics. Learner feedback will be shared, as well as lessons learned and further directions and scope of this type of provision of instruction and service within the arena of systematic and scoping reviews.

What Is My Affiliation? Fostering Faculty Engagement and Institutional Affiliation in Publication Authorship

Track: In Person | On Demand

Mirian Ramirez - Research Metrics Librarian, Ruth Lilly Medical Library, Indianapolis, Indiana

Elizabeth C. Whipple, MLS, AHIP - Assistant Director of Research and Translational Sciences, Ruth Lilly Medical Library, Indianapolis, Indiana

Background: When individual researchers enter their affiliations for publication authorship, there can be a plethora of variations on how their institutional name is represented, including: using acronyms, abbreviations, including the unit’s name with or without a comma, and also, accidental misspellings can occur. This often causes issues when librarians have to search and collect bibliographic information and prepare custom reports based on the school’s or department’s publishing and impact. For instance, librarians struggle to develop specific search strategies to find all faculty bibliographies in databases. As a way to promote the use of uniform affiliation name, the librarians from a midwest medical school’s library created an affiliation lookup tool, using available resources and free online tools, with no coding/programming skills needed.

Description: The affiliation lookup tool was designed to search for uses (correct and incorrect) of affiliation names of the school of medicine units, and assign the “correct” affiliation. We started by creating an Excel spreadsheet using the slicer tool to dynamically display and filter which affiliation names were written in the correct or incorrect form. Using the affiliation slicer as a starting point, the librarians used it to search and quickly check and filter affiliation names to use normalized names while inputting data into the internal journal management and publishing system (OJS systems for the Proceedings of IMPRS). To extend the use of the tool to faculty, students, and staff, we created an online searchable tool using Datawrapper, an online free tool to create responsive, customizable, and live-updating tables. The affiliation lookup search allows researchers to discover, search, sort, and explore affiliation names used in the school of medicine and other clinical partners. In this presentation, we will share the steps we used to create the affiliation slicer and how to transform it into an online affiliation search tool using Datawrapper, a free tool to create visualizations, maps, charts, and tables. In this presentation share the steps for developing the tool.

Conclusion: It’s beneficial for institutions to be able to assert control over how the institutional name should be displayed in authors’ works, not only to track productivity across departments but also to improve the national and international reputation of their research. Librarians play an important role in promoting the uniform use of institutional affiliation in publication authorship. In addition to guiding researchers as part of our outreach activities, this tool is an important component for using the correct and standardized use of affiliation names to reduce future affiliation indexing errors in databases. The final product is shared in the library’s libguide and handouts, and it has led to faculty engagement to promote the correct use of their departments' affiliation names.

PAPER PRESENTATIONS: SPECIAL LIBRARY ABSTRACTS

We have sorted paper presentation abstracts in this section by title in alphabetical order and note presentations available to conference attendees on-demand.

“Chaos is a friend of mine”: Creating and planning services for a new medical library space

Track: Special Libraries | On Demand

Ana Corral, MLIS - Health Sciences Librarian, University of Houston, University Libraries, Houston, Texas

Rachel Helbing, AHIP - Head of Health Sciences Libraries, University of Houston, University Libraries, Houston, Texas

Objectives: This paper will discuss the implementation of a new medical library location in a newly-constructed building at a Carnegie-designated R1 university in a major metropolitan area. The medical library staff report up through the university libraries; however, the building is managed by the medical school. Establishing this new space requires many considerations including effective methods of co-administration, usage, and the provision of shared services. This paper will include strategies, lessons learned, and foster dialog on the creation of library services at a new location.

Methods: Establishing a medical school focused on community health and primary care took many years of planning. In conversations with medical school leadership and the university libraries, planning for a new medical library space began in 2018 with completion of the physical space occurring in September of 2022. Multiple meetings focusing on budgets, services, and planning took place and informed the collaborative approach taken in setting up the medical library. The authors co-wrote a planning proposal that went through several iterations and incorporated feedback from both university libraries and medical school administration. The authors will discuss how flexibility has been an essential component of their process, both for the planning proposal and throughout the implementation of the new medical library space. Factoring in personnel, budgetary, and construction delays has been necessary to certify that the establishment of the space and new services align with the needs of the medical school and the capacity of the new medical library team. The creation of a staggered timeline for the medical library space has been crucial and the authors will share lessons learned along the way, along with strategies for assessment of both the services and the space.

Results: The medical library space is new, with services being approached in a collaborative manner by the health sciences libraries team, consisting of both librarians and circulation staff. Mirroring collections and services offered at the larger health sciences library while identifying and addressing the unique needs of the medical school, and taking a staggered approach to purchasing, personnel, and deployment of services and programming, has been key to ensuring a successful opening without overstretching the capacity of those involved.

Conclusions: Medical library spaces are common additions to new buildings and are necessary for medical education success. The manner in which these spaces are used has evolved over the years and will continue to change with advances in curriculum, technology, and remote learning. With this constant change in mind, planning for and establishing a new medical library requires hard work, clear communication, and a phased planning approach. Being prepared for multiple contingencies is helpful; however, it will be

necessary to embrace the chaos as plans inevitably fall through and new strategies must be created on the fly. Flexibility and patience are required, and the evolving needs of students, faculty, and staff should be at the forefront of planning and execution.

Identifying Knowledge Practices in an Infodemic Era: Rediscovering the Professional Identities of LIS Professionals in an Infodiverse Environment

Track: Special Libraries | On Demand

Joseph M. Yap - PhD Student, Institute of Library and Information Science, Eotvos Lorand University, Budapest, Hungary

Agnes Hajdu Barat - Professor, Institute of Library and Information Science, Eotvos Lorand University, Hungary

Peter Kiszl - Director, Institute of Library and Information Science, Eotvos Lorand University, Budapest, Budapest, Hungary

The professional identity of librarians is contextually established as to the current understanding of their knowledge practices. The framing of librarians with regard to their status has already been explored. With the global phenomenon of false and trustworthy information circulating on social media platforms paints a new issue that librarians must conquer. Messages, content, news, and information on the web make it challenging for librarians to educate users as the sources where they come from need to be evaluated for credibility and trustworthiness.

During the pandemic, a surge of information disorders keeps floating in social media. The health of the people is at stake as infodiverse society listens to many viewpoints some of them harmful to humans. The World Health Organization recommends good health practices through various activities by listening to community concerns and questions, promoting understanding of risk and health expert advice, building resilience to misinformation, and engaging and empowering communities to take positive action. At the height of social media, misinformation is being formed. At some point, it can be a product of an infodemic, false or misleading health information during a disease outbreak.

This LIS research aims to understand how librarians define themselves in recognizing their professional identity in times of infodemic. Their established personal aspects must be reflective of their professional identities. This research seeks to explore the relationship between professional identities and the participation of librarians in an infodiverse environment.

Specific questions include: 1. What aspects or components of professional identity are present in Filipino librarians? 2. Do these aspects or components reflect their knowledge practices when applied to the evaluation of social media health information during the pandemic? 3. How do they respond to an infodiverse environment during a pandemic?

Impact of the Reference Manager Software Zotero on Library Users: A Study

Track: Special Libraries | On Demand

Praveen K. Jain - Librarian, Library, Delhi, Delhi, India

Objectives: Zotero workshops at the College of Veterinary Science, Proddatur are conducted for M.V. Sc, Ph.D. scholars, and veterinary faculties. Librarian collaborates to solve student and staff query about Zotero use. This research initially arose from questions about if and how Zotero was used by library users who had attended the Library’s Zotero sessions. It subsequently developed into a broader investigation of the reference management practices of research scholars and veterinary faculties in the College of Veterinary Science, Proddatur. This article details the findings of the study which asked the following research questions: For what purposes is Zotero being used?

Methods: Users of the library were asked to fill out 100 questionnaires. A total of 64 valid responses from 40 students and 24 faculty members were received and included in the analysis, for a 64% response rate. The SPSS statistical package was used for the analysis (Version 26). Library patrons' opinions on Zotero's usefulness, relevance, and limitations for academic work were compiled using a simple percentage, and binary logistic regression was used to identify statistically significant differences in Zotero use between students and faculty.

Results: Analysis of user data reveals that nearly two-thirds of students and one-third of faculty members use Zotero for article writing, and nearly as many again use it for thesis or dissertation research. More than a third of libraries and more than half of students agree that Zotero is crucial when compiling citations. While writing, 49% of students and 30% of teachers had trouble with properly citing sources. It was found that 44% of students and 30% of teachers felt they had inadequate training on the citation management tool Zotero.

Conclusions: To determine whether there is a discernible distinction between the effects that Library users (veterinary students and faculties) have on Zotero for their professional work, a binary logistic regression was conducted. Two-tailed test: 2(4) = 32.054, p .0005; significance level: high. The model correctly classified 95.3% of cases, and it was able to explain 74.4% (Nagelkerke R2) of the effect of Zotero. While writing, professors are 1.48 times more likely to run into Zotero citation limitations than master's and doctoral students. However, no statistically significant differences were found between faculty and students on any of the other survey variables

In This Together: Challenges and Opportunities for Collaborative Health Sciences Collection Development

Track: Special Libraries | On Demand

Ana Corral, MLIS - Health Sciences Librarian, University of Houston, University Libraries, Houston, Texas

Stefanie Lapka, AHIP - Health Sciences Librarian, University of Houston, University Libraries

Objectives: In this paper, two health sciences librarians at a Carnegie-designated, R1 university located in a major metropolitan area will discuss their experiences navigating collection development in the first two years of their collaboration. The authors will present an account of collection development during the initial stage of the establishment of a new Medical School Library location and activities performed to diversify and enhance existing health sciences collections, including the creation of a health humanities collection, at the Health Sciences Library university branch location.

Methods: All libraries aim to provide reliable, relevant, high-quality, useful, and easily accessible information and services. An ideal objective is the development of a central and reliable library collection that meets all the needs of a library’s users; in the case of university libraries these are the academic and research needs of a campus community. But in today’s technology-driven, budget- conscious environment, collection development at academic health sciences libraries presents issues that can be challenging for the librarians

responsible, especially for librarians new to collection development or whose primary responsibilities are not in collection development. Issues can include: gaining familiarity with institutional collection development and purchasing processes, collaborating with other selectors, managing multiple library locations, acquiring non-traditional material, and developing more diverse and inclusive collections. The authors will describe their approach to collaborative collection development and their efforts to implement this coordinated approach in practice across two health sciences library locations. Particular emphasis will be placed on local and system-wide budgetary, time, and collection development constraints; demand of their respective liaison areas; incorporation of diverse, equitable, and inclusive collection practices; and inclusion of user feedback.

Results: The authors discuss their experiences and efforts implementing a coordinated approach to collection development in the health sciences at an academic research institution. How the collection in a new medical school library location was planned and established is outlined, including a targeted, customized humanities collection that connects to the broader humanities collection. An account of collection development activities to enhance the collection at the Health Sciences Library university branch location, including a health humanities collection, is also given. Lessons learned and strategies for leveraging challenges into opportunities are presented.

Conclusions: Collection development in the health sciences is dynamic and complex. Academic health science libraries must support the ever-increasing needs of their institutions while balancing a finite budget and responding to fluid collection development practices. Despite these ongoing challenges, collaborative approaches to collection development in the health sciences allow for building inclusive, intersectional collections that support discovery and health in the twenty-first century, and reflect voices of the communities served. The authors recognize the need to document their activities over the course of the next several years to assess the effectiveness of their collaboration. Analyzing both quantitative and qualitative data will help identify potential areas of improvement and to elaborate more fully on areas that have proven to be effective.

Piloting a Buddy Program for New Hires

Track: Special Libraries | On Demand

Dana Abbey, AHIP - Engagement Specialist/Associate Professor, Strauss Health Sciences Library, Aurora, Colorado

Objectives: In 2021, we piloted a Buddy Program for new hires as a part of the onboarding process. The buddy served as an additional resource to the new employee’s supervisor for onboarding. The intent of the buddy relationship was to ease the transition for the new hire and provide a peer connection to assist with questions in the first months of hiring and promote a friendly working relationship across departments. The buddy program was not intended to limit contact or replace informal communication with other colleagues. All library staff were encouraged to interact with the new hire either formally or informally.

Methods: The pilot was launched during the COVID-19 pandemic, with the intent that it would fill the gap of serendipitous interactions that would have occurred if the new staff member was on site. All library staff were invited to be considered as buddies, with the final selection made by the new hire’s supervisor. Matches were made based on common interests, work experience, or the potential for collaboration between the individuals, as well as peer-to-peer level. During the pilot, three new hires were assigned a buddy. Each pairing committed to meeting regularly during the first two months, and then once a month for the remaining two months. The pair decided if they wanted to continue meeting after the initial four months.

Results: Overall, participants thought the pilot was a valuable experience. Feedback from the Buddies and the new hires indicated positive interactions and the program helped new hires build community in a virtual setting.

Conclusions: The pilot was successful in that it provided new hires with a support system outside of their supervisor interactions, and provided an opportunity to interact with other staff outside of their department.

Mentoring Health Science Library Students: Practices and Perceptions from a Regional Program

Track: Special Libraries | On Demand

Dana Abbey, AHIP - Engagement Specialist/Associate Professor, Strauss Health Sciences Library, Aurora, Colorado

David M. Brown - Engagement Specialist, Coe Library, Laramie, Wyoming

Objectives: Developing the skills, knowledge, and abilities to be successful in health sciences librarianship is challenging. Only 40% of health sciences librarians in the U.S. and Canada responding to a survey were interested in health sciences librarianship when starting their graduate programs. Only 40% of the 64 institutions offering ALA-accredited MLIS programs offer health sciences librarianship or health informatics in the curriculum. Many of these programs have very few courses addressing the breadth of skills needed in the subspecialty. Mentorship programs focused on health sciences librarianship can bridge the gap for students wanting to explore and gain experience in this specialization.

Methods: We utilize a number of theories and models to engage students and ensure both mentors and protégées receive a quality experience when working on a project. The basis of all of our mentorship comes from the Theory of Cognitive Development and Social Learning Theory. These theories allowed participants to learn from one another and make the program rewarding for both. Within these theoretical foundations, some specific models and theories are highlighted in our program: Vygotsky’s Zone of Proximal Development emphasizes the relationship between mentor and protégée. Both the mentor and protégée work on a feedback loop to enhance their skills both as mentors and protégées. Within the Social Learning Theory, we mainly focus on modeling/observational learning. We have our protégées observe and then model/practice quality health librarianship. Students applied for consideration as a protégée in the regional program to work on a specific project with the program specialists and program administrators who all are MLIS/MLS-trained librarians. During the first weeks of their internship, they were acclimated to the region and our program. They then began working on a special project in the area of health science librarianship. Recent regional projects focused on diversity, underrepresented communities, and working with diverse populations.

Results: Students met regularly with the program specialists and also received guidance from their academic institution's internship advisors to ensure they were making progress on their projects as well as achieving the goals of their internship program. Students attended staff meetings and shared questions and or findings discovered while working on projects. A unique expectation of the program is that we request/require students to take their projects to a professional library conference or publish a peer-reviewed journal article on their experiences. This helps them learn the art of scholarly tone and writing and provides an additional artifact for their professional dossier.

Conclusions: Our regional program has a history of successful mentoring opportunities for graduate library and information studies students. Through the use of established principles and procedures, students and mentors have reported positive experiences and have been able to use the projects to advance their skills and professional development in the field of health science librarianship. While no program is perfect, our mentorship program relies heavily on established theoretical foundations and established practices to ensure the best possible outcomes for both the students and the mentors. We hope to continue outreach

and engagement with student mentees to assist in filling the need for highly trained health science information professionals.

Teambuilding and Leadership Development with Action Learning

Track: Special Libraries | On Demand

Susan H. Schneider, M.S. Library/Info Sci; EdM - Director, Anhinga Coaching LLC, Annapolis, Maryland

Objectives: The teambuilding and leadership development potential of Action Learning are well-documented by Coaches accredited by the World Institute for Action Learning (WIAL). The approach supports teambuilding by giving each member ideal conditions for participation in the problem identified by the Stakeholder(s). At the beginning of the Action Learning "set" each participant identifies the leadership skill they want to develop (for example, Active Listening/Collaboration/Influencing).

Methods: When an urgent and important problem is posed by the Stakeholder, a diverse team of six to eight individuals comes together virtually, in-person, or in a hybrid setting. After introductions, each team member states the leadership competency they want to develop. The Action Learning Coach presents the two (only two) rules for the session, called a "set." They are: 1: a statement can only be made in response to a question, and 2: the Coach may intervene when the Coach sees a learning opportunity. Then, the Stakeholder describes the urgent and important problem. As the set progresses, the problem is further defined. Each team member asks questions to clarify the problem. At the right time, the Coach asks if all team members understand the problem. If yes, then one of the team writes the problem statement. When all team members agree that the problem is well-stated, the next phase begins. The team discusses solutions. When the coach observes potential solutions, the Coach checks if the Stakeholder agrees. If not, solutions are explored further. When the Stakeholder is satisfied, the team members reflect on the Action Learning approach, and comment on their own and their teammates' demonstration of their leadership competency.

Results: The achievements of an Action Learning set occur on several levels. First, the problem is explored and agreed-upon. The team does not waste time working on the wrong problem. Second, each team member has the opportunity to ask (and answer) questions without interruption. None of the team members can make statements unless they are asked a question; no one dominates the Action Learning session. Third, the power of questions is evident and developed with practice. Fourth, each team member practices their chosen leadership competency, reflects on their competency demonstration, and receives feedback from the team members and the Coach.

Conclusions: The teambuilding and leadership competencies development supported by the Action Learning model succeed on several levels. Members practice their competency of interest, reflect on their practice, and receive feedback from team members and the Coach. The technique for gaining consensus on the problem saves time and creates space for solutions from people who think differently. All team members contribute. The extraordinary power of asking questions, (instead of giving advice, or prematurely solving the wrong problem) result in remarkable achievements when Action Learning is facilitated by an experienced, credentialed Coach.

Waste Not, Want Not: Exposing the Secret Fragments of History in a Medical Rare Book Collection

Track: Special Libraries | On Demand

Damien Ihrig - Curator, John Martin Rare Book Room, University of Iowa Libraries Hardin Library for the Health Sciences, Iowa City, Iowa

Elizabeth Stone - Collections Conservator, University of Iowa Libraries

Eric Ensley - Curator of Rare Books & Maps, University of Iowa, Iowa City, Iowa

Objectives: This paper details a project within a health sciences rare book collection embedded in an academic health sciences library to locate, describe, identify, digitize, and catalog manuscript/printer's waste - manuscript or printed material recycled to make new books. This new information broadens the connections to non-medical/non-scientific areas of western history and extends the collection's usefulness to disciplines beyond those typically accessing history of medicine collections.

Methods: Using material from existing books to make new books is a well-established practice. These bits of books, referred to as waste, are found throughout rare book collections. Book makers were not picky about the waste they used. They used leaves from old manuscripts, test prints, usable pages from damaged books, and printed ephemera, among others, to support spines, make repairs, and cover books. And occasionally, the only existing versions of particular works are found as fragments in other books. Librarians at a health sciences rare book collection undertook an effort to locate, describe, identify, digitize, and catalog manuscript/printer's waste in the collection. Phase 1 of the project identified books most likely to contain waste. The books were inspected for evidence of waste in Phase 2. Other curators/conservation staff will try to identify the hands and the type used and, if possible, the original work in Phase 3. The catalog will be updated in Phase 4. Digitization of the waste will take place during Phase 5. Future phases may include inspecting more books and utilizing imaging technologies.

Results: Based on a cutoff date of 1700 and binding condition (e.g., we excluded modern bindings), 1,311 books were surveyed for waste. This maximized the likelihood of finding waste while providing a reasonable number of books to survey. 63 books contained evidence of waste, with 152 individual instances identified for verification and description. Preliminary images and metadata were recorded. Phase 3 is ongoing as the team works to verify the waste,and identify the hand/type and the original works. The status of Phases 3 and 4 will be updated at the time of the presentation. Potential future phases will also be discussed.

Conclusions:

LIGHTNING TALK PRESENTATIONS: RESEARCH ABSTRACTS

We have sorted lightning talk presentation abstracts in this section by title in alphabetical order and note presentations available to conference attendees on-demand.

Asking Nurses How They Really Evaluate Information on the Job

Track: Innovation & Research Practice | On Demand

Brandon Patterson - Technology Engagement Librarian, Eccles Health Sciences Library, Salt Lake City, Utah

Betsy S. Hopkins, MLS - Nursing Librarian, Harold B. Lee Library, Provo, Utah

Nena Schvaneveldt, MLIS - Associate Librarian, Eccles Health Sciences Library, Salt Lake City, Utah

Anne R. Diekema, MLS - Instruction Librarian, Gerald R. Sherratt Library, Cedar City, Utah

Objectives: This talk includes preliminary results from a study of evaluation processes and criteria that nurses use on the job, completed by a multi-institutional research group studying nurses’ information behavior. Previous studies from this group resulted in more specialized approaches to teaching evaluation skills and reformed information literacy instruction in baccalaureate nursing programs.

Methods: To capture how professionals apply information literacy skills at their place of work without running into HIPAA constraints, we conducted an anonymized survey and scenario-based interviews with a variety of nurses connected to the Mountain West. We surveyed close to 500 nurses and conducted eighteen interviews during the summer of 2022.

Results: Preliminary findings indicate that nurses have confidence in their existing knowledge, implicitly trust certain sources, and tend to utilize basic evaluating criteria similar to the CRAAP test (currency, relevance, authority, accuracy, and purpose).

Conclusions: For this talk, we will discuss scenario-based interviews as a data collection method and highlight compelling findings from the study including evaluation remorse, container focus, and librarian connections.

Assessment of Library Services in Pediatric Hospitals in the United States and Canada

Track: Professionalism & Leadership | On Demand

Brian L. Baker - Library Services Program Manager & Literacy Program Coordinator, Medical Library, Valley Children's Healthcare, Madera, California

Kate Daniels, n/a - Clinical Librarian, Children's Healthcare of Atlanta, Atlanta, Georgia

Christine Willis, AHIP - Clinical Librarian, Inman Medical Library / Children's Healthcare of Atlanta, Atlanta, Georgia

Amy Six-Means, MLIS - Medical Librarian, Holman Family Resource Library

Claudia Schuchardt-Peet, MS, MLIS - Medical Librarian, Education Resource Center, Chambersburg Hospital, zoom, Pennsylvania

Priscilla L. Stephenson, AHIP, FMLA - Retired, James A. Haley Veterans Hospital, Tampa, FL, Tampa, Florida

Objectives: The aim of this project was to examine the scope of library services in pediatric hospitals in the United States and Canada. The study sought to determine how library services at these hospitals compared on 20 specific metrics and to see if having a U.S. News and World Report designation or Magnet Accreditation influenced support or services. This expands the research of a 2021 survey by authors involved with this study which focused on pediatric hospitals in the southeast United States.

Methods: In October 2022, the authors compiled a list of pediatric hospital librarians using directories of the Children's Hospital Association, the MLA Pediatric Librarians Caucus, the MLA Hospital Library Caucus, and DOCLINE libraries. These 149 librarians received email invitations to participate in the research project and a link to a REDCap survey. In November 2022, survey reminder messages were sent to the electronic mail lists of both the Hospital Library Caucus and the Pediatric Librarians Caucus. The team reviewed the combined list of responses to limit responses to single entries from individual facilities. The survey asked respondents to identify their hospital's size and organizational structure, the library's predominant user groups, scope of library services, databases provided, staffing, fiscal operations, and library space allocations.

Results: After eliminating duplicate responses, 54 remained (36% response rate). Most (85%, n=46) facilities were teaching organizations, 96% (n=52) had residency or fellowship programs, 61% (n=33) U.S. News designation, and 65% (n=35) held Magnet Accreditation. Staff size varied: 35% (n=19) were solo librarians, another 22% (n=12) reported 2 FTE, and 30% (n=16) had 3-5 FTE staff. Although 83% (n=45) of study participants were members of a national professional association, 57% (n=31) lacked AHIP certification. While clinical staff made up the primary patron group, 54% of users (n=29) were faculty. Patients and families were library users for 35% (n=19) of the respondents.

Conclusions: Pediatric hospital libraries provide a full range of medical library services with small staff sizes and modest budgets. The number of mediated, topic-focused searches does not correlate to the number of FTEs or number of ILLs exchanged. Responses to the four open-ended questions reveal that librarians are working at their fullest capacity, generally feel supported, and would like to expand services and resources. The authors need to review the data, but at this time it can be said that almost all hospitals have some institutional motivation to provide library staff. Additionally, the responding hospitals’ data needs to be compared to the MLA Hospital Library Standards and Canadian Health Libraries Association Standards to see what insights that can provide..

The Best Rx is Better UX: Redesigning, Migrating, and Usability Testing a Hospital Library Website

Track: Education | On Demand

Brandy Klug - Clinical Engagement Librarian, Gibson D. Lewis Library, UNT Health Science Center, FORT WORTH, Texas

Objectives: The John S. Marietta Library team provides onsite services as part of a collaborative agreement between the UNT Health Science Center and JPS Health Network. The team was tasked with migrating the hospital library website to a new content management system (CMS) in Summer 2021. The library has an existing Springshare subscription which includes access to a CMS platform. Options were limited due to budget constraints, so it was decided to move forward with migrating the website to LibGuides CMS. The project also provided an opportunity to completely reimagine the website and conduct usability testing to improve user experience (UX).

Methods: A literature review identified several examples of library website usability testing which integrated

a series of tasks and the System Usability Scale (SUS). Very little literature in hospital library settings was identified. Work began on a LibGuides CMS development website in Fall 2021. The team analyzed frequently asked questions and “pain points” users often reported when using the website. This information helped inform early design choices and guided the creation of a set of usability testing tasks. A group of eight JPS Health Network clinicians and staff were recruited for testing. Participants were observed during the process and qualitative data such as comments and questions were noted. Quantitative measures such as time spent on each task and rate of task completion were also recorded. Participants completed the SUS to rate their experience after completing the tasks on each website. Data and feedback were analyzed and incorporated into the new website design once testing was completed.

Results: Usability testing data indicated marked improvements in user experience and other measures with the new website design. The former website received an average SUS score of 69.7 which is equal to a letter grade of “C” according to the curved grading scale interpretation of SUS scores. The new website received an average SUS score of 81 which is interpreted as a letter grade of “A.” Participant comments were much more favorable for the new website. Data also indicated a reduction in the amount of time required to complete tasks on the new website.

Conclusions: Conducting thorough usability testing with a diverse group of clinicians and staff provided valuable insights regarding the search behavior and needs of this unique patron population. The process also identified additional areas for improvement moving forward. The new website launched June 7, 2022. Staff plan to conduct additional usability testing in 2023 to ensure the improvements made continue to benefit website users.

A Bibliometric Analysis of Graduate Medical Education (GME) Trainees' Publications in a Large Medical School

Track: Information Services | On Demand

Julia C. Stumpff - Instructional Design Librarian, Ruth Lilly Medical Library, Indianapolis, Indiana

Mirian Ramirez - Research Metrics Librarian, Ruth Lilly Medical Library, Indianapolis, Indiana

Rick Ralston, MSLIS - Associate Director for Content Management and Assessment, Ruth Lilly Medical Library, Indianapolis, Indiana

Objective: Graduate Medical Education (GME) residents are required to meet ACGME-mandated scholarship requirements. (1) Often those residents are encouraged to publish the resulting scholarship, and librarians are asked for assistance during that process. We will investigate two things: the publication patterns of residents and the journals they cite in their publications. By examining publication patterns, we will learn which types of articles residents typically author, in which departments trainees publish, etc. This knowledge will help librarians better target guidance provided to residents. Likewise, the analysis of the cited journals will inform collection development efforts that support resident publishing.

Methods: This is a descriptive ‘baseline’ exploratory study. 266 residents who completed their training between July 1, 2018, and June 30, 2019, were searched in Scopus. Included in this analysis are case study/case series, original research, or review articles authored by residents and with our institution listed by any author. Articles were limited to publication dates that were one year after the trainee start date and 18 months after the trainee termination date. Excluded were published curriculum, editorials, conference papers, and conference posters. Articles were coded separately by two different authors (case study/case series, original research, or review articles). When there were disagreements, a third author made the final decision. The analysis included journals in which GME residents published, number of publications, number of residents publishing, number of publications cited, distribution of publishing among programs, and journals cited within publications.

Results: Out of the residents matriculating 2018-19, 34% published at least one article, and they published in 188 different journals. 291 total articles were published by residents, and of those articles, the majority, 60%, were original research. 41% of first authors were GME residents. Of resident program areas, Surgery had the highest number of publications and citations. When analyzing publications per resident, Urology and Otolaryngology tied for first with 8.33. Otolaryngology had the most citations per resident with 110.7. There were 8073 cited references in 2288 journals. The top 20 journals were cited more than 50 times. CONCLUSIONS: GME residents publish in a wide variety of journals. Libraries might provide guidance about publishing original research to support their scholarly publishing. Further, libraries can evaluate if their collections meet their residents’ research needs by examining journals cited in residents’ publications.

The limitations of this study are that the analysis included one institution and one year of matriculating residents. Also, inter-program comparisons did not account for differing time-in-residency. Next steps are to analyze the inter-program data by time-in-residency, use the Chi-Squared test to look for relationships, and analyze journal data by program area. Eventually, other GME trainees’ publications will be analyzed. The analysis will then expand to trainees from multiple years for a more complete picture of GME trainee publishing. 1. Accreditation Council on Graduate Medical Education. ACGME Common Program Requirements (Residency). July 1, 2022. https://www.acgme.org/globalassets/pfassets/programrequirements/cprresidency_2022v3.pdf

Changing Internal Budget Models: An Opportunity to Reconnect with Library Users

Track: Information Services | On Demand

Clare F. Keating - Electronic Resources Librarian, Assistant Professor, University Libraries, The University of Toledo, Toledo, Ohio

Derek Wilmott - Acquisitions and Collection Management Librarian, Assistant Professor, University of Toledo, Toledo, Ohio

Gerald R. Natal, BFA, MLIS, AHIP - Health and Human Services Librarian, Associate Professor, University Libraries

Daisy Caruso, BA - Library Media Technology Assistant, University Libraries

Margaret A. Hoogland, MLS, AHIP - Associate Professor and Clinical Medical Librarian, The University of Toledo, Toledo

Objectives: Switching internal budget models from a centrally-determined budget to one that includes contributions from colleges provides University Libraries with an opportunity to revisit current services and support provided to community members.

Methods: A team consisting of an Acquisitions and Collection Management Librarian, the Electronic Resources Librarian, two Health Science Liaisons, and a Staff Member considered distributing well-known and recognized surveys (LibQual+, MISO) to Health Science faculty, staff, and administrators. After considering the needs of the community, however, the team decided to start by creating a local survey. After receiving IRB approval, the team distributed the survey in Mid-November to Mid-December of 2022.

Results: The team received 112 responses from Health Science administrators, faculty, and staff. Many participants have used library services for 16+ years, but most Administrators started using the library in the past six years. Many participants expressed curiosity about cost-share agreements for maintaining or subscribing to databases and e-journals. The majority of participants supported improving immediate access to full-text articles instead of relying on interlibrary loan. Many participants had some experience with Open Access publishing. When determining the publishing model for future submissions, however, participants had mixed feelings.

Conclusions: The team will present their findings to University Library administration in early Spring 2023. The team will further investigate issues such as interest and support for Open Educational Resources, educating the community about publishing formats, exploring the feasibility of cost-share agreements, and improving communication between the University Libraries, Health Science administrators, faculty, and staff in the community.

Data Service Needs of Graduate Students and Postdocs During Research Disruptions

Track: Information Management | On Demand

Debra A. Werner - Director of Library Research in Medical Education, John Crerar Library, University of Chicago, Chicago, Illinois

Jenny Hart, MLIS - Mathematics, Statistics, Computer Science and Physics Librarian, John Crerar Library, University of Chicago

Objectives: The COVID-19 pandemic and subsequent shutdown of physical work spaces on university campuses had a significant effect on the work processes of graduate students and postdoctoral researchers in the sciences. Many were unable to physically access their laboratories and other facilities. Consequently, research workflows were disrupted, including data collection, analysis, and management. Our aim was to determine the research data service needs of graduate students and postdoctoral researchers, under normal circumstances and during the disruption created by the COVID-19 pandemic, in order to determine where library services could be implemented to fill service gaps.

Methods: We conducted focus groups of graduate students and postdoctoral researchers in science disciplines at the University of Chicago in the winter and spring of 2021. Four focus groups were held via Zoom with a total of 11 participants; participants were grouped by similar research activities to the extent possible. The focus groups took the form of semi-structured interviews with a script of prepared questions related to the kind of data they collected and how they collected it, who in their lab was responsible for managing the data, how their data was managed and stored, and what kind of data services they used or would like to be able to use. Interviews were recorded and transcribed. Two librarians and a data management fellow coded transcripts in Dedoose and performed a thematic analysis to inform the conclusion. The questions for the semi-structured interviews were written before the Covid-19 shutdown, therefore specific questions about the effects of the shutdown were not included. However, because the focus groups were conducted a year into the pandemic, many of their responses reflected the conditions the participants worked under during the shutdown and problems they encountered over this time.

Results: Results indicated a wide variety of data management practices across labs, even within the same subject domain. Reported issues included: difficulty organizing and naming files; trouble processing, sharing, and storing large data files; and issues accessing previous graduate students’ data. Due to limitations in licensing and funding, often attributed to inequities, participants lacked necessary software to process and analyze their data. Participants also reported problems specific to the shutdown: difficulty accessing data remotely from laboratory instruments and campus core facilities due to policies and security protocols and lack of remote access to needed software and hardware.

Conclusions: Graduate students and postdoctoral researchers face challenges collecting, organizing, and managing their data, challenges that are amplified in an increasingly remote environment. Laboratory practices and policies can inadvertently create barriers, causing students and postdocs to alter their research practices, which, at times, adversely affects their work. While some issues necessarily need to be addressed by individual laboratories, graduate programs, and core facilities, health sciences librarians and science librarians can provide beneficial services in data management principles and best practices, in both in-person and remote environments.

Experiences of Librarians Working with Physician Associate (Assistant) Programs: A Mixed Methods Study

Track: Education | On Demand

Megan Jaskowiak - Health and Social Sciences Librarian, Miami University Libraries, Oxford, Ohio

Michelle Nielsen Ott - Sciences/Health Sciences Librarian, Bradley University, Cullom-Davis Library

Karina Kletscher, AHIP - Reference & Instruction Librarian, Creighton University Libraries, Phoenix, Arizona

Objectives: Physician Associates (PA) graduation rates from the 217 accredited Physician Associate (Studies) programs are increasing while 20 new programs are in development (https://www.ajmc.com/view/physician-assistants-associates-at-6-decades). The research reporting on how academic health sciences libraries serve these programs is minimal. The purpose of this mixed methods exploratory study is to examine the practices and perceptions of librarians/information specialists who are or will actively support PA programs.

Methods: This study is being conducted in two phases. First, librarians or information specialists who are or will actively support PA programs will be surveyed. Descriptive statistics will be used to evaluate the survey results. The survey includes an invitation to participate in semi-structured interviews. Second, the persons who respond ‘yes’ to the semi-structured interview request will be contacted and will be virtually interviewed until saturation is reached.

Results: Upon acceptance of this abstract for a lightning talk, this section will be updated with our preliminary survey results.

Conclusions: This study’s results will expand the knowledge of what resources and services PA programs utilize and how libraries can best collaborate with them. These findings will assist both librarians/information specialists already working with PA programs and those at institutions developing new PA programs.

Identifying Predatory Journal Publications: An Institutional Review

Track: Innovation & Research Practice | On Demand

Nicole T. Alexander - MLIS Graduate Student, University of South Carolina/Medical University of South Carolina, Decatur, Georgia

Emily Brennan, MLIS - Associate Professor, Medical University of South Carolina/Research and Education Services, Charleston, South Carolina

Objective: Predatory publishers attempt to lure authors into publishing their research in questionable, non-peer-reviewed journals that can affect one’s professional reputation, research reproducibility, and the integrity of scientific literature. To better inform and target educational efforts for the faculty and staff we support, we sought to identify university-affiliated articles that have been published in potentially predatory journals within the past three years.

Methodology: A health sciences librarian compiles publications authored by students, staff, and faculty at our academic health sciences system. We examined those published during a three-year period (January 1, 2020, to December 31, 2022) to determine how many were published in potentially predatory journals. This subset of publications was compiled in EndNote and then exported to Excel for analysis. The evolving

nature of predatory journals makes identification challenging. Therefore, multiple sources were consulted to identify potentially predatory journals, such as the NLM Catalog, Directory of Open Access Journals (DOAJ), Open Access Journals List of Predatory Journals, Beall’s List, JANE (Journal/Author Name Estimator), and Scopus. If needed, individual publishers' websites were evaluated using the “Think. Check. Submit.” checklist criteria. From the resulting set of potentially predatory publications, we analyzed data elements such as journal titles, authorship details, publication types, and trends over time.

Results: 7459 MUSC-authored publications were identified in the three-year period. Data analysis is in progress but analysis of half of the publications (n=3730) yielded preliminary results. 86 duplicates were removed, resulting in 3644 unique publications: 1144 publications in 2020, 1154 in 2021, and 1346 in 2022. The NLM Catalog was searched, and 602 publications (16.5%) were published in journals not indexed in MEDLINE. 92 publications (from 39 journals) required further evaluation; 2 were book sections.

Conclusions: This research study is still in process, with final results and conclusions forthcoming. Librarians will use the institutional data to target outreach and educational efforts related to predatory publishing, such as potential department-level workshops and resource development.

Is Your Brain Hungry? A Mixed-Methods Study of the Whole Academic Student

Track: Innovation & Research Practice | On Demand

Jasmine Shumaker - Reference & Instruction Librarian, University of Maryland, Baltimore County, Baltimore, Maryland

Semhar Yohannes - Science Reference & Instruction Librarian, University of Maryland, Baltimore County, Baltimore, Maryland

Objectives: An ongoing, mixed-methods multi-part study using statistical software between a public institution Library and the on-campus food bank. This research explores the possible link between student usage of the Library pantry and a positive impact on their lives in the following areas: academic performance, mental health, social health, and physical health.

Methods: Academic librarians, specifically those in the health sciences field, are experts at navigating information and resources regarding food insecurity. They distribute materials and find unique ways to accomplish their health literacy goals. This includes “providing instruction on locating current and reliable information on nutrition and other components of a healthy lifestyle and dietary needs [to] enable them to make healthier lifestyle changes” (Lemley & Fenske, 632-633). This study examines an ongoing mixed-methods, multi-part study between a public institution’s Library and the on-campus food bank. The research aims to explore the possible relationship between student usage of the Library pantry and a positive impact on their lives in the following areas: academic performance, mental health, social health, and physical health. We hypothesize a positive association between the two variables. Part one of the initiative includes a discussion of campus demographics, specifically campus food insecurity statistics, and how that contributes to the overall need for the Library mini pantry space. Part two examines the in-progress identification of the main campus pantry users and the mini pantry users. In part three, a report will be generated using campus data software assessing the positive association between student usage of the main pantry and a positive impact on their lives. Part four will be a duplication of part three but focusing on the mini pantry users. Possible workflows and obstacles will also be discussed.

Librarians' Perspectives on Restricting Literature Searches to the "5-year rule" and Other Strict Search Criteria

Track: Information Services | On Demand

A'Llyn Ettien, AHIP - Head of Resource Sharing and Discovery, Boston University Alumni Medical Library, Boston, Massachusetts

Eleanor Truex - Medical Librarian, Chicago Metro Region for Ascension Illinois, Ascension Saint Joseph Hospital, Chicago, Illinois

Emily N. Spinner - Program Information Specialist, Ellis Medical Library, Schenectady, New York

Jean Hillyer, AHIP - Assistant Director, Learning Resource Center retired, Baylor University, School of Nursing, Double Oak, Texas

Sarah Wade, AHIP - Medical Librarian, Campbell University School of Osteopathic Medicine, Lillington, North Carolina

Grace Wolf, MLS - Instruction and Outreach Librarian, Central Carolina Community College, North Carolina

Courtney Calhoun, DNP, RN-BC, CNE, CHSE - Nursing Instructor, Nursing, Bailey, North Carolina

Rebecca Hedreen, MLIS, MAEd, MSBio - Librarian, Research & Instruction, New Haven, Connecticut

Layla Heimlich, AHIP - Medical Librarian, William B. Glew, MD, Health Sciences Library, MedStar Washington Hospital Center, Washington, District of Columbia

Annie Nickum, AHIP - Assistant Professor & Information Services Librarian, Library of the Health Sciences, University of Illinois Chicago, Chicago, Illinois

Susan C. Vonderheid - Clinical Assistant Professor, UIC; Director Nursing Research, UIH, College of Nursing, University of Illinois Chicago (UIC); University of Illinois Hospital (UIH), Illinois

Objectives: The purpose of the larger study was to explore reasons for common literature search criteria (i.e. the 5-year rule, nurse as author, and primary study) used by nurses, and attitudes toward searching and subsequent participation in evidence-based practice. This analysis describes medical librarians’ perspectives on the criteria and the implications for practice when working with direct care nurses, nursing faculty, and student nurses.

Methods: Design: This was a qualitative descriptive study using online focus group interviews of four target populations: medical librarians, direct care nurses, nursing faculty, and student nurses. We conducted 3+ focus groups per target population, allowing us to obtain rich, detailed information to achieve our study purpose. Transcripts were examined using thematic analysis. Sample: Convenience and purposive sampling was used to create a study sample of medical librarians, direct care nurses, nursing faculty, and student nurses that represents maximum variation in geographical regions across the USA. Inclusion criteria are faculty or registered nurses who are direct employees of an organization; faculty who teach in associate degree or baccalaureate degree programs; nurses with a terminal degree of ADN or BSN; librarians who work with any of the above. Analysis: Content analysis was conducted to identify key words and phrases representing themes from the four study populations. Participant socio-demographics and other characteristics were analyzed using descriptive statistics.

Results: Analysis revealed several common themes across populations, such as prioritizing a date range when searching the nursing literature. The limits of “primary study” and “nurse as author” were not as common. Some evidence from focus groups suggests that “nurse as author” is a waning trend, and that “primary study” is a vague term not readily understood by the librarians, faculty, nursing students or practicing nurses involved in our study.

Conclusions: Our initial premise, that the stringent “5 year rule” criterion for searching the nursing literature is ubiquitous, appears to be borne out by discussions with all groups. This presentation will report on the impact of that criterion as observed by medical librarians, with implications for work with practicing nurses, nursing students and faculty.

Library Snackables: A Study of One-Minute Library Videos

Track: Education | On Demand

Robert Tomaszewski - STEM Librarian, California State University, Fullerton, Fullerton, California

Objectives: Library snackables can be defined as pedagogical approaches that communicate useful and understandable information content in short bites. The objective of the study is to connect and communicate with Generation Z students on library services and resources through the use of one-minute videos. The effectiveness of one-minute library videos were examined from the following research questions: RQ1 - What are the perceptions of one-minute library videos? RQ2 - Do one-minute library videos provide audience engagement? RQ3 - Do one-minute library videos provide knowledge retention? and RQ4 - How can one-minute library videos be improved?

Methods: Six one-minute videos were created using the Camtasia software (i.e., Chat Online, Interlibrary Loan, Online Guides, Subject Librarians, Free Software, and Accessing E-Books). Student learning outcomes and one broad learning outcome question were developed for each video. The videos were aligned to the ACRL Framework for Information Literacy for Higher Education. The KISS principle (“Keep It Short and Simple) was applied when designing these short videos to avoid superfluous redundancy and complexity. Appropriate branding and closed captions were added to each video in Camtasia to meet ADA web accessibility standards. A mixed-methods survey instrument using Qualtrics and YouTube viewing metrics was used to gauge student opinion, analyze audience engagement, and evaluate knowledge retention. The library videos were used between June 2021-2022 during instruction and made available on LibGuides.

Results: An analysis of 206 survey respondents determined quantitatively and qualitatively that library users have positive perceptions of one-minute videos (RQ1 and RQ4). The high scores from the survey questions testing students’ understanding and recall of information indicate a high level of knowledge retention (RQ3). A total of 802 YouTube views were used to compare watch times, viewing duration, and audience retention graphs from the one-minute videos (RQ2 and RQ4). YouTube metrics uncovered an average percentage viewed coverage of 76% on average per video, indicating that one-minute videos hold the watchers’ attention (RQ2).

Conclusions: Short videos serve as practical tools for communicating library information. As a result of the tech-savvy nature of the next generation of students (Gen Z and beyond), it is anticipated that the demand for short videos and other “Library Snackables” will only continue to increase. The onus will be on librarians to move forward with using innovative communication tools and pedagogy approaches to accommodate the changing needs of library users.

Open Access and Open Data Sharing Among Health Sciences Researchers in Nigeria

Track: Education | On Demand

Biliamin Oladele Popoola, n/a - Systems, Scholarly Communications and EBM Librarian, University of Medical Sciences Library, Ondo City, Ondo, Nigeria

Violet Ikolo, n/a - Senior Librarian, Medical Library, Abraka, Delta, Nigeria

Objectives: The aim of this study is to access the extent to which open-access publishing and open data sharing are being adopted by health sciences researchers in Nigeria. Specifically, the study focuses on determining: the rate of publication in open-access journals among health sciences researchers; the availability of openly-accessible research data from the health sciences researchers; the proportion of the journals with data availability policy, and the extent of compliance by the researchers; the open data-sharing platforms commonly used by the researchers; and the library services that would be suitable to support the researchers towards adopting Open Science.

Methods: We conducted a literature search for publications in the last five years (2018 - 2023) from Nigerian authors on PubMed. Thereafter, we reviewed the latest 500 publications from Nigerian health sciences researchers as of the date of conducting the literature search. Article types labeled as "reviews" and "systematic reviews" were excluded from the search.

Results:

Conclusions: Data collected from this study are presently being analyzed to draw inferences and conclusions before the MLA annual meeting. Findings from the study are expected to provide a hint about the understanding of African researchers on research data management and open science. This is expected to aid targeted support services from libraries in order to promote research data management and open science in Africa.

Partnering for Research in Healthcare and Computable Biomedical Knowledge

Track: Education | On Demand

Deborah E. Swain - Professor, NC Central University, School of Library and Information Sciences, Raleigh, North Carolina

Objectives: Review results of surveys and interviews of 1) librarians and knowledge managers; and 2) medical and health researchers/developers. The primary research question was: What hinders library and research partnerships? Technology? Trust? Semantics? Training and expertise? Metadata? The objective of this knowledge-based research and presentation is to identify aids and barriers to sharing data and programs on healthcare in open publications, accessible repositories, and collections of knowledge objects, artifacts, and resources in learning health systems.

Methods: Sharing knowledge and working in a “commons” environment can promote growth in healthcare and enhance experiences for professional librarians and biomedical researchers. The Mobilizing Computable Biomedical Knowledge (MCBK) movement (learning model in Figure 1) advocates in the US and globally and is supported by the Learning Health Sciences Department at Michigan’s Medical School. This presentation explores ways to build partnerships among librarians and researchers to support open access in healthcare and to promote effective learning. Note: The continuous cycle of learning in MCBK is based on David Kolb’s experiential learning cycle (Kolb, 1984). Building on MLA 2020 presentation (Conte, et al., 2020), and multiple collaborations by members of MCBK workgroups and pilot training on MCBK funded by the Institute of Museum and Library Services (IMLS), about 10 librarians and 10 researchers/developers were surveyed and interviewed (semi-structured) in a preliminary research study. Social research methods (Wildemuth, 2017) were applied in gathering input from the volunteer subjects. Using the SECI model (Figure 2) for knowledge management (KM) conversion of and creation of knowledge

(Nonaka and Takeuchi, 1995), the collected data was analyzed to define both explicit and tacit ideas from the subjects that hinder or support stronger partnering.

Results: Results are a process for sharing knowledge as an example of how looking back at SECI and MCBK models can help professionals in libraries and clinics to forge ahead as partners (theme of the conference). The process from this preliminary research study provides specific actions to apply in current jobs. Learning health systems can reduce the time from lab or data research to implementation from years to months –if data, programs, algorithms, and knowledge resources are shared openly and validated by data librarians. However, success will require partnerships with library, repository, and knowledge experts by medical and health clinical researcher.

Conclusions: It is hoped that MLA and SLA conference attendees can apply the experience-based ideas collected and analyzed to learn how to partner for stronger health systems and to expand experiential learnings about computable biomedical and health data. Continued data collection and collaborative efforts are proposed for future research. Learning modules on aspects of MCBK and metadata models have been developed as Open Education Resources (OER) at NC Central University (www.nccu.edu) to sustain research and learning by library and clinical professionals to create digital libraries that store collections of computable knowledge objects (code) in addition to human-readable knowledge. Researchers and biomedical developers may forge a future with reduced time-to-implementation knowledge through partnering with library and information professionals.

Understanding Medical Librarians' Role in LGBTQ+ Competency in Medical Education and Training

Track: Education

Jordan Correia, AHIP - PhD Student, Rutgers School of Communication and Information

Objectives: Several medical and healthcare institutions have called for LGBTQ+ and transgender competency within medical curricula because of the health disparities they face and have taken steps to achieve this goal, including hosting workshops and panelists focused on LGBTQ+ competency in patient care. Several different groups aid in LGBTQ+ competency interventions for healthcare professionals, staff, and students, including medical librarians. This is an exploratory study aimed to understand the scope of medical librarians' participation and assistance with LGBTQ+ workshops within their institutions.

Methods: A Qualtrics survey was designed to focus on the work medical librarians perform or don't perform for LGBTQ+ competency workshops within their institutions for their healthcare professionals, staff, and students and the institutional support for these workshops, those who conduct them, and LGBTQ+ competency. This survey was targeted towards medical librarians who were based in the USA and had worked within their current role for at least one calendar year. The term 'medical librarian' in this study is used to describe a librarian or library worker that works within a medical, clinical, healthcare, academic (with a focus on medical or health sciences subject area), or health sciences institution. Eleven respondents were selected for 60-90 minute semi-structured interviews in order to better understand their role in LGBTQ+ competency in medical education, institutional support they receive, and any barriers they face in conducting or supporting these workshops.

Results: From the 60 survey responses, 45 were analyzed. The majority of respondents stated they primarily served medical students, faculty, and staff. Most respondents also indicated that their institution and/or department had a DEI policy. However, only four respondents indicated that their libraries held LGBTQ+ competency workshops. Interviews revealed that other individuals or groups held these competency workshops, but the majority of them stated that they would like to play a larger role in providing competency. They stated that barriers to helping conduct workshops included time constraints, workload,

and lack of staffing. Participants overall felt supported by their institution. When asked how their institution could better support them, they stated better funding and acknowledgement of their work would be ideal. From questions about LGBTQ+ competency within their medical institution, it was shown that there was a distinct difference between cis LGB and T health needs, such as gender clinics.

Conclusions: Overall, medical librarians do not play a large role in LGBTQ+ competency workshops, but many would like to have a role in helping develop or conduct them. Significant barriers are involved in allowing them to contribute, including under staffing, workload, and time constraints. It's particularly important for medical librarians to assist with LGBTQ+ competency as they are information professionals, often support instruction and curricula, and assist with research. Institutions can better support their medical librarians by providing more funding, including more staff support, and highlighting their work more. Finally, while medical institutions often distinguish between the health needs of the different LGBTQ+ community members through services offered, competency needs to highlight to medical students, faculty, and staff those differences and the importance of them, both the patient-provider relationship needs and medical needs of each member group.

Updating and Re-Validating LGBTQIA+ Search Filters

Track: Information Services | On Demand

Andy Hickner - Education and Outreach Librarian, Samuel J. Wood Library, Chicago, Illinois

Hannah Schilperoort, MLIS, MA - Head, USC Wilson Dental Library, Los Angeles, California

Chelsea Misquith - Librarian, currently unaffiliated, Toronto, Ontario, Canada

Margaret J. Foster, AHIP - Director, Center for Systematic Reviews and Research Syntheses, TAMU, Bryan, Texas

Jane Morgan-Daniel, MLIS, MA, AHIP - Community Engagement and Health Literacy Librarian, University of Florida Health Science Center Libraries, Gainesville, Florida

Kate M. Saylor - Informationist, Taubman Health Sciences Library, Ann Arbor, Michigan

Tracy C. Shields, AHIP - Biomedical Librarian, National Institutes of Health Library

Francis Toole, N/A - Student, School of Information Management

Robin M. N. Parker, MLIS - Evidence Synthesis Librarian, W. K. Kellogg Health Sciences Library, Halifax, Nova Scotia

Objectives: To update and re-validate a previously validated PubMed filter for LGBTQIA+ populations to incorporate new MeSH and keywords, and re-validate filters for some subpopulations (e.g., Women who have Sex with Women, bisexual people) using larger gold standard sets, with input from community members and stakeholders. A secondary objective of this project is to develop a comprehensive list of relevant terms to describe each subgroup (and relevant research related to the population in question), recognizing that not all terms may be currently used in health-related literature and also acknowledging that harmful language may have been used historically.

Methods: Each subpopulation filter is being updated in collaboration with one or more members of that subpopulation, adapting a collaborative model developed for similar projects by the MLA Latinx Caucus. Using the relative recall method, we are creating gold standard sets for each subpopulation filter from relevant reviews and will use them to evaluate the performance of each filter. In addition to summarizing our research methods, we will discuss theoretical frameworks that informed both our definitions and the inclusion criteria for selection of the gold standard sets for each subpopulation. We will also describe our approach to project management, team roles, and division of labor.

Results: We will summarize lessons learned during the first year of this ongoing project related to barriers and facilitators; definition of subpopulations; protocol and action plan development; project management; cross-institutional collaboration; and cultural competence. We will offer key takeaways for other groups seeking to undertake similar projects.

Conclusions: N/A

LIGHTNING TALK PRESENTATIONS: PROGRAM DESCRIPTION ABSTRACTS

We have sorted lightning talk presentation abstracts in this section by title in alphabetical order and note presentations available to conference attendees on-demand.

Academic Publishing is Not Neutral: Explorations of Bias and Constructed Authority in Health and Science Knowledge Production in a Term-Long Course for Students

Track: Education | On Demand

Tova Johnson - Critical Pedagogy & Research Librarian, OHSU Library, Portland, Oregon

Background: Two librarians at Oregon Health & Science University designed a 1-credit, term-long elective for graduate students in the biomedical sciences that provided students with functional knowledge of information searching and information management through a critical framework. This inclusion of critical information literacy aligned with a shift in 2016 in how the Association of College and Research Libraries (ACRL) framed information literacy. This talk will focus on how the instructors designed the course to facilitate students' recognition that how the authority we grant to information and information sources is “constructed and contextual,” one of the principles in the new ACRL Framework. Audience members for this talk will gain ideas for their own critical information literacy instruction.

Description: Instructors provided weekly content to read or view that guided students to reflect on the non-neutrality of information, including seemingly “objective” scientific information. Some aspects of information creation and appraisal that students were invited to critically reflect on included the problematic nature of how information is categorized in knowledge organization systems, the effects of algorithmic bias on search results in information retrieval systems, how bias shows up in research studies, and biases and exclusions in scientific publishing. Concerning the latter, and the focus of this talk, students recognized that research studies with positive results are much more likely to be published, much of what is published is from researchers or perspectives in the “global north,” alternative paradigms of knowledge production are often ignored or not recognized, and women or people of color are less likely to have their scientific research published. Students developed strategies for discovering and accessing literature representing a broader diversity of scholars/scholarship and for mapping the gaps and biases in the literature related to their research topics. Students also reflected on the politics of citation and how they could improve their own citational practices to better recognize the contributions of marginalized scholars.

Conclusion: An end of term survey and student reflections on their growth over the course of the term in their information practices revealed that students were more aware of and reflective about bias in academic publishing, how to develop more comprehensive search strategies to overcome gaps caused by issues of bias in the research literature, and how to improve their own citational practices in the furtherance of social justice.

Adding Value to Project ECHO’s Tele-Mentoring Model: The ECHO Librarian Role

Track: Innovation & Research Practice | On Demand

Bonnie Leigh Reifsteck, MA - Digital Librarian, University of New Mexico - Project ECHO, Albuquerque, New Mexico

Kent Norsworthy - Library Services Manager, University of New Mexico - Project ECHO, Albuquerque, New Mexico

Background: Project ECHO was launched by Dr. Sanjeev Arora in 2003 with the goal of delivering specialized medical knowledge to rural health care providers through no-cost virtual mentoring and case-based learning. ECHO has proven effective across disciplines and geographies as a way to reduce disparities, strengthen health systems, and drive collaborative solutions. There are now over 900 ECHO hubs running more than 5,500 ECHO programs in 62 countries, reaching participants in 193 countries. We introduced the ECHO Librarian role to our showcase ECHO programs in 2020 to enhance the value of each session for ECHO participants, medical directors, and subject-matter experts on the hub team, and to reinforce ECHO’s brand as a trusted source of evidence-based information.

Description: The ECHO Librarian role (aka, “embedded librarian” or “digital librarian”) was first developed by our partners at ECHO Ontario in 2015. We adapted and piloted the role in New Mexico as part of our COVID-response ECHO programs beginning in 2020. The Librarian serves as a member of the interprofessional hub team, providing links to references and resources in the Zoom chat during the session. Most ECHO programs run weekly for one hour, and include a didactic presentation and an anonymized clinical case presentation. The Librarian reviews presentation slides prior to the session, reviews and prepares the references for posting in the Zoom chat, and then searches in real-time for references mentioned by subject matter experts during discussion. We began implementing the role in four of our core COVID-response programs with international audiences. With an overwhelmingly positive response from participants and experts, we expanded the role to begin serving more traditional, long-standing ECHO programs. We track the programs, sessions, and number of participants served; the number of references posted; and the participant feedback on regular post-session surveys. This Information about the ECHO Librarian role is published to a dashboard that demonstrates the impact of our service.

Conclusion: Since piloting the role in Spring 2020 through the end of 2022, our ECHO Librarians have provided support to 32 unique ECHO programs, serving over 100,000 participants on 680 sessions and posting over 8,000 references. We currently support 16 ongoing ECHO programs, covering topics such as COVID-19, Behavioral Health, Climate Change and Human Health, Dermatology, Endocrinology, Hepatitis C, HIV, Infectious Diseases, Palliative Care, Post-COVID, Reproductive Health, and Vaccine Confidence. The role has been featured in several recent peer-reviewed journal articles. We have found that the role is most effective with ECHO programs that are anchored by a strong community of practice and that include research-rich didactics.

Authorship or Acknowledgement? A Complicated Question!

Track: Information Services | On Demand

Jennifer Deberg, OT, MLS - User Services Librarian, University of Iowa, Iowa City, Iowa

Background: For librarians supporting systematic reviews, determining whether to request authorship, acknowledgement, or neither can be a conundrum. This talk will present an overview of the factors surrounding these decisions including a few case examples from my experience supporting reviews. The aim is to stimulate ongoing critical thought, discussion, and ideas to provide improved guidance. With the rise in publication of systematic reviews, awareness of the need for librarian expertise has grown, supported by reputable organizations such as the Cochrane Collaboration. Recently, the Medical Library Association released an authorship statement to provide additional support for co-authorship in publication. There is potential for this statement, important as it is, to be misinterpreted as a standard of practice or a mandate.

Description: Decision challenges are most likely to arise when considering accountability for research integrity. For example, when the practices of the research team do not seem conducive to accuracy/integrity, authorship does not seem to be the best choice. In these types of situations, is it better to be acknowledged for sloppy work than not recognized at all? Also, due to the volume of requests coupled with communication challenges, it can be difficult to keep up with progress of supported reviews. This may result in the librarian not seeing the final manuscript prior to submission, which is an obvious problem

Conclusion: It would be unwise to interpret the recent authorship statement mentioned as a directive to always seek authorship for supported reviews. Although the evidence correlating librarian co-authorship with more complete and transparent methodology should be communicated to systematic review teams, it should viewed as support for sustained librarian involvement on review teams. A case by case approach using sound professional judgment should guide decisions regarding attribution.

Beyond Freud: Decentering the Patriarchy, Improving Accessibility, and Expanding Usership in a Psychoanalytic Research Archive

Track: Information Services | On Demand

Christine Morse - Library and Archival Services Coordinator, New Center for Psychoanalysis

Background: There is a growing shift within psychoanalysis, as the field takes concrete steps towards expanding access to both its training and treatment. This is reflected in the diverse student body of the author’s place of work, a psychoanalytic training and research institute. In 2017 the center invested in establishing an educational and research archive centered around documenting the history of psychoanalysis in Los Angeles. Since the author stepped into their role as Library Services Coordinator in 2022, the archive has received little use. How might the archive position itself as a resource that is welcoming, inclusive, engaging, and in which its users see representations of themselves?

Description: Although this project is in an embryonic stage, support from leadership is strong. An initial meeting resulted in identifying both immediate action items and longer arching issues to address. Immediate items to implement include rewriting the home page for the archive, focusing on welcoming and orienting visitors to the collection (launch date February 5th, 2023), and marketing its materials in the institute’s bi-monthly newsletter, with the intent on featuring images and other content that reflect gender, educational, and racial diversity in the field. The importance of engaging in outreach efforts directed at faculty, making them aware of the archive and welcoming them to make use of its primary source material in their course content was also discussed and identified as an immediate action to take, as courses are offered year-round. Longer arching projects include generating a stronger presence of search terms in the archival software finding aids meant to yield access to primary source material in which users from a range of backgrounds and experiences might see themselves reflected.

Conclusion: Motivated to move away from the homogenous tone and website presence of the archive, and

focusing efforts organized around promoting its contents in a way that reaches a broader audience within the institute and the community at large, the author predicts usership to increase, particularly due to the promotional material that will be feature in the bi-monthly newsletters and in making connections with faculty, drawing attention to their option to use the archive materials in their syllabus content. Measuring the outcome of the project will be done by coordinating with the institute’s information technology (IT) team to capture website-use numbers, and by manually recording the (expected) increase of in-person users.

Centering Community in Maternal Health Equity: Creating Opportunities for Conversations

Track: Information Services | On Demand

Ophelia T. Morey - Associate Librarian, Health Sciences Library Services, Buffalo, New York

Background: Relative to other racial and ethnic groups, Black women are at a significantly higher risk for pregnancy-related death and post-birth complications. Community members express a need to understand why Black women have worse pregnancy outcomes, and ask for action to address this disparity and racial inequities. Based on feedback from the community, local departments of health recommend community-level strategies, such as support for community-based programs and services, increased awareness for health care services, and educational programming. Therefore, this project aims to increase awareness, and to educate the community by amplifying the voices of community members around these maternal health issues.

Description: This project will aim to increase awareness of maternal health issues by implementing three events: 1) a webinar, 2) an in-person screening of a documentary about Black maternal health, and 3) an in-person community unconference where attendees will have the opportunity to participate in community proposed sessions. These events will also feature consumer health resources. Community members will have the opportunity to participate in all three events as attendees or speakers. In addition, community members will be invited to propose and vote on proposed conference topics and activities. Evaluation of project objectives will happen at the end of each community event. The success of each event and the overall project will be indicated by the number of attendees, responses to surveys, and by recording observations.

Conclusion: The expected outcomes are that each event will be well attended, with active participation from community members where they indicate increased awareness and education about maternal health topics and consumer health resources. This talk will highlight the outcomes of the project activities.

ChatGPT Generated PubMed Search Strings

Track: Information Services | On Demand

Breck Turner - Informationist, Welch Medical Library, Owings Mills, Maryland

Background: ChatGPT is an artificial intelligence-powered chat bot. It is trained on data collected from the internet. So far, it has been popularly used to answer questions and create summaries based on user input. ChatGPT is also able to generate search strings for PubMed based on user queries. The goal is to determine whether ChatGPT is consistently capable of generating search strings that are usable and/or worth using based on user queries.

Description: ChatGPT will be tested to determine functionality in creating PubMed search strings based on user queries. These tests will be on criteria such as: How many concepts can it handle combining in one search? Can it properly utilize field codes? Can it determine appropriate MeSH terms and use them correctly in the search? Are there significant logical or syntax errors that would keep search strings from being used or from running correctly? How specific do users have to in their queries to ChatGPT in order to generate usable search strings?

Conclusion: Outcomes that I expect to measure include the quality of the PubMed search strings generated by ChatGPT and the feasibility of users being able to input queries that will generate the desired search strings. These outcomes will help inform whether or not ChatGPT is worth including in a medical librarian's toolkit and determine what potential uses it might have in this context.

Collaborative Approaches to Improving MeSH

Track: Information Management | On Demand

Violet Fox, MLIS - Cataloging & Metadata Librarian, Galter Health Sciences Library, Northwestern University

Kelleen Maluski, MLS - Student Success and Engagement Librarian, Health Sciences Library & Informatics Center, University of New Mexico

Background: In June 2022, a group of medical librarians published an open letter to the National Library of Medicine (NLM) sharing concerns about the terminology used within NLM’s Medical Subject Headings (MeSH). The letter described problems with the opacity of the review process for new and revised terms in MeSH. NLM responded with a letter partially addressing concerns and announcing a series of listening sessions to facilitate communication about the updating of MeSH. In response to the increased interest in MeSH among medical librarians and NLM’s efforts in transparency and partnership, a new program has started: the Medical Subject Funnel. The Medical Subject Funnel is an informal structure for library workers to collaborate on proposals for additions or revisions to MeSH.

Description: The members of the Medical Subject Funnel work together with an aim of accurate, up-to-date, culturally sensitive, and respectful terminology within MeSH, in the service of enhancing access to library resources. The funnel co-chairs coordinated with the Program for Cooperative Cataloging and the National Library of Medicine to create the new program, which is open to any library worker interested in medical terms in controlled vocabularies. Training sessions and monthly meetings were established to create opportunities for learning and discussion about potential improvements and how revisions might impact searching in PubMed and library catalogs.

Conclusion: Evaluation of the program will consist of periodic surveys which will assess members’ learning about the MeSH revision process and confidence in making proposals for change. Along with the revision of subject headings, this program is being created with the intention of acting as a welcoming space for all library workers (especially those new to libraries) to share information and ideas; survey questions will also ask participants about the collegial aspects of the Medical Subject Funnel as a space for peer-to-peer learning and shared creation. In addition, the program will be appraised for its ability to make substantive changes in MeSH terminology by recording if and how members’ concerns were addressed within MeSH after the NLM yearly review process.

Consider the Fully Remote Option: Advice from a Fully Remote Academic Medical Librarian

Track: Professionalism & Leadership | On Demand

Jaena Manson - Scholarly Communications Librarian, Boxer Library at Rosalind Franklin University of Medicine and Science, St. Paul, Minnesota

In August 2022, I was hired as a fully remote Scholarly Communications Librarian at Rosalind Franklin University of Medicine and Science (RFUMS). Even after the COVID-19 pandemic’s reshaping of the hybrid/remote job landscape, fully remote academic librarian positions like mine remain rare. This is despite data from Pew Research, that shows that, of those who have jobs which can be done remotely, 60% would like to work from home all or most of the time. Libraries and academic institutions in general have not traditionally been thought of as options for remote workers, but I argue that libraries should consider this option to stay competitive in our current (and future) workplace environment. In this lightning talk I will discuss the benefits of offering fully remote librarian positions to both the employee and the library, as well as what to consider before deciding if a fully remote position will be appropriate.

Correcting Misinformation in the Clinic: A New Course for Medical Students

Track: Education | On Demand

Ian Roberts - Acquisitions and Resource Sharing Librarian, Himmelfarb Health Sciences Library, Washington, District of Columbia

Rachel Brill - Reference and Instructional Librarian, Himmelfarb Health Sciences Library, Washington, District of Columbia

Stacy Brody, n/a - Reference and Instructional Librarian, Himmelfarb Health Sciences Library, Washington, District of Columbia

Background: Librarians provide instruction to first-and second-year medical students on information literacy. The rise of misinformation, its impact on the medical profession, and the development of related sessions across other courses inspired the authors to develop an instructional session on misinformation and the SIFT framework. SIFT (Stop. Investigate. Find. Trace.) is one evaluative framework that has been applied to online health information. Librarian instructors delivered the approximately 80 minute instructional session to small groups of eight first-year medical students. In this lightning talk, the authors describe the development of the session, its implementation, and future directions.

Description: The authors used the P-HLET (pre-work, hook, link, engagement, and transfer) instructional design framework to plan the session and identified examples of online health claims. Prior to running the session, the authors’ colleagues tested the SIFT framework on pre-selected articles. A brief PowerPoint slide deck framed the session and introduced definitions of misinformation and disinformation. Most of the session time was devoted to a small group activity. In groups of 2-3, students applied the SIFT framework to examples of online health claims. Librarian instructors purposefully selected articles from a variety of sources, largely popular news sites, such as CNN and “wellness blogs,” and identified examples with connections to specific issues in research, for instance, retractions. The goal was to practice appraising information that might be presented by a patient in a clinical setting. A research guide about correcting misinformation, which described communication techniques, SIFT, and other resources, was introduced to students during this session for further learning. No formal evaluation was conducted. The authors gathered

informal feedback from colleagues and reflected on their experiences delivering the session

Conclusion: Librarian instructors reported high engagement. Groups discussed various information literacy and scholarly communication topics. In the future, the authors hope to expand the session to encompass communication skills, providing role play scenarios for students to communicate with patients presenting misinformation claims. This session also empowered the authors to discuss additional instruction opportunities across the medical school curriculum, especially as other instructors have expressed interest in the topic of misinformation. Weaving this session content into different parts of the MD curriculum would help instructors and students integrate key skills, attitudes, and behaviors. The authors hope to adapt this session to other audiences and to continue collecting example articles, along with SIFTing reports.

Crafting Research Impact Statements: Helping Faculty Demonstrate Their Value

Track: Innovation & Research Practice | On Demand

Judy Smith - Librarian, Health Sciences, University Library, Ann Arbor, Michigan

Rebecca Welzenbach, MSI - Research Impact and Information Science Librarian, University of Michigan Libraries, YPSILANTI, Michigan

Sara Samuel - Informationist, Taubman Health Sciences Library, University of Michigan, Ann Arbor, Michigan

Tyler Nix, MSLS - Assistant Director (Interim), Research & Informatics, Taubman Health Sciences Library, University of Michigan, Ann Arbor, Michigan

Background: Library colleagues at a large academic institution have formed teams within the health sciences library and the central library to address research impact needs of faculty, students, and staff. Our team strives to educate the campus on using bibliometric indicators effectively within broader evaluation frameworks. Our primary mission is education and training, including individual consultations, and close partnership with our Offices of Research and a cross-campus Research Development Community of Practice. The team often presents tools containing bibliometric indicators that can be used for multiple assessment purposes, such as grant proposals, promotion and tenure, or awards.

Description: The team wanted to evolve our instruction from pointing to tools and providing definitions of bibliometric indicators, to giving our users practical applications. However, developing a relevant, pragmatic workshop on writing research impact statements was more challenging than anticipated. For example, we knew we wanted to demonstrate how bibliometrics might be used in grant review processes, but we could not find clear evidence of their impact. Nevertheless, we moved forward with how researchers could use bibliometric tools to enhance funding applications, with workable suggestions on reaching out to program officers. We also modified our idea from a very focused workshop on research impact statements for the National Institutes of Health to a workshop that could address many disciplines. Ultimately, we created an all-campus session where attendees could better understand how to use both subscription-based and free tools to write research impact statements. We provided an overview of research impact frameworks, such as the Translational Science Benefits Model, with the focus of the session being sample statements and best practices for integrating evidence. Evaluation involved a brief survey after the session, and we will also be hosting a lunchtime research development networking event sponsored by the research development community of practice.

Conclusion: The first round of our presentation resulted in increased interest on campus, and we plan to continue to refine the content and present again this spring based on user feedback and instructor reflections. Additionally, there have been requests for more tailored sessions, such as an upcoming career

development office hour for early and mid career health services and policy researchers. We anticipate more requests for customized sessions, as our first session tried to accomodate all disciplines. Finally, we hope to learn more about how faculty and staff are using these tools for their research impact statement needs after attending our sessions, and how successful these research impact statements have been in achieving their goals.

Current Mood? Polling in Instruction Sessions

Track: Education | On Demand

Stacey Greenwell - Coordinator of Educational Services, University of Kentucky Libraries, Lexington, Kentucky

Background: The presenter for this session has the MLS and a doctorate in instructional design plus over 20 years experience in libraries and technology. The presenter has been teaching both library instruction sessions and semester-long courses. Given this background in educational technology and libraries, the presenter can provide attendees with useful tools for creating their own instructional content.

Description: Based in educational research, interactivity in instruction is key for learning, particularly with college/university students and potentially other adult learners. This lightning talk will inspire you to interact with students via polling software. The presenter will demonstrate several examples including topic development, keyword generation, source characteristics, and more. These quick examples will give you ideas to create your own interactive instruction elements.

Conclusion: Recently the presenter developed a series of interactive exercises for library instruction sessions and shared them as part of the EDUCAUSE/Penn State Instructional Designer to Instructional Designer program. The presenter would like to share these exercises with the MLA/SLA community in a lightning talk at the 2023 Annual Conference.

Demystifying Peer Review for Information Professionals

Track: Innovation & Research Practice | On Demand

Margaret Graton - Research & Education Intern, Duke University Medical Center Library

Leila S. Ledbetter, AHIP - Research and Education Librarian, Liaison to the School of Nursing, Duke University Medical Center Library, Durham, North Carolina

Samantha Kaplan - Research & Education Librarian, Liaison to the School of Medicine, Duke University Medical Center Library, Durham, North Carolina

Sarah Cantrell, AHIP - Associate Director for Research and Education, Liaison to Graduate Medical Education, Duke University Medical Center Library, Durham, North Carolina

Background: Poorly conducted systematic reviews (SRs) are making it through the peer review process and into the published literature. This stems from the journal editor’s or reviewers’ lack of knowledge of SR methodology and an inability to recognize poorly conducted SRs. Librarians play an important role in producing quality SRs, and therefore should be included in the peer review process for SRs. Despite being SR experts, many qualified librarians do not feel confident enough to complete peer reviews or to approach journal editors to offer peer review services. We designed an online, synchronous educational intervention to help medical librarians learn the skills and confidence necessary to complete peer reviews for SRs.

Description: We designed a peer review class, based on the authors' professional experience in academic publishing and peer reviewing for academic journals. The peer review class covers topics like journal roles, the parts of a peer review, tips for completing peer reviews, special considerations for SR peer reviews, and how to receive recognition for peer reviews. We developed a pre- and post-educational intervention survey to gauge librarians' experience and comfort levels with completing peer reviews for academic journals. We initially plan to assess librarians’ knowledge of, and comfort level with, peer review, and then gauge if their comfort level and perceptions change after the educational intervention. The class will be offered twice, is one-hour in length, and is offered online through a synchronous Zoom presentation. We will advertise these two opportunities to health sciences librarians via several Medical Library Association listservs and groups, namely the Scholarly Communications Caucus, Systematic Review Caucus, and Expert Searching lists. In an effort to increase survey completion, people interested in taking the class will receive the Zoom link upon completion of the pre-intervention survey. The initial evaluation of the intervention includes analysis of the pre and post-intervention surveys.

Conclusion: We will use the pre- and post-intervention surveys to evaluate and assess the impacts of the project. Ideally, the peer review class will give librarians the skills to seek out peer reviewing opportunities and the ability to confidently review articles. It will also help build a pool of seasoned peer reviewers who can then mentor their colleagues and help them become peer reviewers earlier in their career.

The DMPTool NIH template project: Policy analysis, practical implementation

Track: Information Management | On Demand

Nina Exner, PhD, MLS - Research Data Librarian, Virginia Commonwealth University Libraries, Richmond, Virginia

Katy Smith, PhD, MLIS - Health Sciences Reference Librarian, Saint Louis University Medical Center Library, St Louis, Missouri

Matthew Covey - University Librarian, The Rita & Frits Markus Library, The Rockefeller University, New York, New York

Genevieve Milliken, MA, MSLIS - Data Services Librarian, NYU Health Sciences Library / Data Services, Wallkill, New York

Background: After the NIH’s new Data Management and Sharing (DMS) Policy was announced, researchers needed guidance to help make DMS Plans. Members of the Editorial board of DMPTool, the data management planning and plan templating tool, formed a team of librarians to work on an NIH DMS Plan template. Because there was not one canonical policy document to harness, the template project team had a complex policy analysis job. The team combined librarians who had extensive DMP-writing experience but no NIH experience, with librarians who knew health and the NIH but not the DMPTool. Together this team of librarians worked to synthesize and curate NIH policy guidance in preparation for this year’s DMS Plan rollout.

Description: Over the course of different additions to guidance from varying routes, the Template team strove to reach consensus on what the most useful parts of the Policy and related content would be. In several cases, our attempts to guess the future turned out incorrect and we had to backtrack changes to match new understandings. Throughout, the committee members read NIH policy statements closely for what was said and what was not said in the DMS Policy and related guidance. What was not specified often came into discussions as much as what was specified. In addition, past experiences and current use cases were discussed to try to test the usefulness of what was included in the Template. Furthermore, popular

demand encouraged the addition of “example answers” to help researchers and librarians see how one might address the DMS Plan elements. However, there is not one answer that can cover most or even a good range of data scenarios. The committee members therefore worked on providing a range of possible example answers that would encourage, rather than limit, researcher thinning through how different data might need different answers to the Plan elements.

Conclusion: The DMPTool NIH-GEN DMSP template has been frequently updated to keep up with evolving text and guidance. In addition, content has been added in response to community input. By bringing multiple perspectives together, we enriched the conversations and built greater expertise. The Template reflects best known practices based on evolving and uncertain guidance, to help support everyone with the new Plan. In this session we will show the Template and touch on key policy documents that were incorporated, to help attendees understand how policy and practice come together in the DMPTool NIH template for data management and sharing.

DMPTool Review

Track: Information Management | On Demand

Damiana Fortenberry - Instructor, UAB Libraries, Helena, Alabama

Background: A few academic health science librarians have set out on a research project that will result in supporting public librarians dealing with health-related reference questions. Although the group's project was not funded by NIH, they set out to create a data management plan using the DMPTool. As this was the group's first experience with data management or any of the tools associated with creating a data plan, it feels important to discuss what the experience was like and the data management concepts it encouraged us to consider.

Description: One person was placed in charge of selecting a data management tool and gathering the thoughts, opinions, and preferences of the other members of the group in order to integrate all perspectives during the process. Although many other data management planning tools are available, the group decided on using DMPTool for its ease of use and adaptability. One notable advantage for those needing a data management plan that met the requirements of certain funding organizations could search for acceptable data management plan templates within the tool. As this wasn't necessary for our particular project, we could forgo that service and follow a basic template with a simple outline. Once completed, our data management plan resulted in 5 pages, but would likely expand to 6 or 7 pages given we had not decided on a long-term data storage plan at the time it was completed.

Conclusion: The group ended up with a data management plan using the DMPTool that was sufficient for our purposes. Even without the need to ensure our funding was maintained, the tool asked questions we hadn't considered and probably wouldn't have thought to consider without the tool's prompting. For example, our project's foundation was started 20 years ago by a now-retired academic health sciences librarian. A recent discovery of the surveys they used was very helpful, and when the DMPTool queried us on our long-term storage plans amongst librarians at different academic institutions, we had to consider our data storage. In this way, I think a data management plan should become a best practice for any research project, regardless of funding status.

Earn While You Learn

Track: Clinical Support | On Demand

Elizabeth Frakes, MSIS, AHIP - Assistant Librarian for Clinical Services, Spencer S. Eccles Health Sciences Library, Salt Lake City, Utah

Shawn Steidinger, AHIP - Associate Librarian for Clinical Services, Spencer S. Eccles Health Sciences Library, Salt Lake City, Utah

Background: At a PAC-12 university, clinical librarians have embedded their expert evidence consult service into the health system’s electronic health record (EHR). The service allows clinicians to seamlessly request literature to evidentiate both research and patient-specific clinical questions (e.g. treatment plan, medication choice), while utilizing the EHR. Consequently, clinical workflow is not interrupted allowing for a better patient experience and questions are answered as they arise. To increase usage of the consult service, the librarians teamed with the Graduate Medical Education Office to grant CME credits for use of the EPIC service. The objectives of this partnership were threefold: increasing awareness of clinical library services, increasing the utilization of the consult service, and ascertaining if the evidence provided changed clinical practice.

Description: The expert consult service debuted in EPIC in the Fall of 2020. Various marketing pushes have been implemented to varying success. The expert consult service employs the EHR message system to request librarian help. An emailed alert notifies the team of a submitted query. Clinical librarians with EHR access (granted after EPIC training and permission from system administrators) can log in to view the message which includes clinical question, patient record number, and priority level. This EHR access permits the librarians to view the relevant patient record, enabling a patient-specific tailored response that includes a summary of the literature, attached articles, and/or links to collections of PubMed citations. Additionally, the response includes the link to participate in a 5 question Learner Survey to earn 0.5 CME hours for having read the supplied packet and articles. The survey collects information such as change in practice, effectiveness and usefulness, and satisfaction with information and service. The librarians hope to use the data collected to provide evidence of impact on patient outcomes and clinician knowledge.

Conclusion: The partnership with the GME Office was implemented in March 2022. Data will be collected annually starting March 2023 to calculate the number of awarded credits. Additionally, analysis will be conducted on the responses to the survey questions. A data assessment will be conducted to determine the change in number of clinical questions asked the year prior to the GME partnership as compared to the year afterwards. Collected data will be used to justify the continued provision of GME credits through the consult service as the financial support through the GME office is expected to cease. The data may also be used to identify potential knowledge gaps to be addressed in the medical school curriculum and GME offerings.

Enhancing Collection Equity with the Diverse Voices Toolkit

Track: Health Equity & Global Health | On Demand

Kristi Torp - Project Specialist, NNLM Region 5, University of Washington Libraries

Background: This lightning talk describes the process for creating the Diverse Voices in Health & Medicine Collection Development Toolkit developed by a grant-funding organization. The Toolkit’s purpose is to assist libraries in expanding diverse voices in their collections by sharing resources from regional members selected to enhance collection equity and support health literacy. Twenty-seven member organizations in the region, including public libraries, research and academic libraries, public health agencies, and community organizations, participated in the project.

Description: The grant funding organization funded 27 collection equity awards to member organizations in the region that used the grants to purchase materials that supported the collection equity goals submitted in

their applications. Some examples of these goals are: expanding eBook resources on health-related social justice & diversity, equity, and inclusion topics, materials for emotional and social aspects of health, medical issues in underrepresented young adult populations, highlighting women physicians, and the LGBTQIA+ experience. As a requirement of the award, each member organization submitted a bibliography of purchased materials using a template provided by the grant funding organization. Staff then compiled, deduplicated, and arranged the materials by subject, resulting in over 1400 unique resources from multiple genres and formats. The Toolkit was divided into four collections (Adult, Young Adult, Children’s, and Zines), free to download as searchable pdf files. To determine the impact of the Diverse Voices Toolkit, staff collected quantitative data showing over 1200 downloads between the launch date of September 28, 2022, and March 28, 2023. Staff also compiled and compared the number of materials per subject to evaluate which topics are most in demand in the region.

Conclusion: Requiring bibliographies from member libraries that received the collection equity awards enabled the grant funding organization to compile a toolkit of resources unique to the health needs of their regional member organizations. The collection data revealed that resources about mental health and health topics for people of color are most in demand in this region. This freely available Toolkit equips users to see the materials that reflect the voices of the communities served, illuminate health issues facing underserved populations, and discover previously unknown resources.

Ensuring Digital Accessibility of Subject Guides

Track: Education | On Demand

Anna Biszaha - Assistant Professor/Research & Education Librarian, The Ohio State University Health Sciences Library, Columbus, Ohio

Background: Confirming that library websites and electronic resources are digitally accessible is of vital importance to ensuring that our services and materials are available to all users. The LibGuides system from Springshare allows library staff to create quick and easy subject guide resources for our patrons with minimal website design knowledge, and they have many accessibility features either baked in or available for use. However, many of these tools are ultimately reliant on content creator awareness, uptake, and implementation. As such, it is important to evaluate subject guides with an accessibility lens to ensure they are maximizing these features and are accessible to all.

Description: The Health Sciences Library at The Ohio State University maintains more than 50 published subject guides. These guides are created by a variety of librarians and library staff. In an effort to ensure that these guides meet basic digital accessibility standards, I will be undertaking a standardized review of all published guides. The rubric used to assess the guides will be focused on those digital accessibility pieces which are reliant on the content creators. Examples of rubric items will include the use of alt text for images, descriptive text for links, and appropriate use of headings within rich text boxes. After completion of the initial assessment, issues discovered will then be remediated to improve the digital accessibility of the guides. In an effort to prevent guides from falling out of digital accessibility in the future, a small-scale training program is also planned to be embedded within our monthly department meetings. This lightning talk will describe the overall technical and administrative approach used for the review and remediation process, as well as a status update on the current progress made and lessons learned.

Conclusion: This project is currently ongoing. The rubric elements have been identified and refined, and an online intake tool has been created as a LibWizard Form. Initial guide reviews have highlighted several system-wide or administrative issues that can be corrected across the platform, and the most commonly seen problems so far have been insufficient alt text for images and using layout tables. By creating the review instrument as an online form, it is hoped that the information collected will be consistent, well-

organized, and easily exported for analysis and remediation. Additionally, the form will allow other staff members to potentially review guides in a standardized fashion in the future.

Establishing Organizational Identity: Collaboratively Creating a Library Mission and Values Statement

Track: Professionalism & Leadership | On Demand

Margaret Ansell, MLIS, AHIP - Nursing & Consumer Health Liaison Librarian, University of Florida, Health Science Center Libraries, Gainesville, Florida

Jane Morgan-Daniel, MLIS, MA, AHIP - Community Engagement and Health Literacy Librarian, University of Florida Health Science Center Libraries, Gainesville, Florida

Hao Ye, PhD - Reproducibility Librarian, University of Florida, Academic Research Consulting & Services (ARCS)

Hannah F. Norton, MS, AHIP - Chair, Health Science Center Library - Gainesville, University of Florida, Health Science Center Libraries, Gainesville, Florida

Melissa L. Rethlefsen, MSLS, AHIP - Executive Director & Professor, University of New Mexico, Health Sciences Library and Informatics Center

Background: In a year-long participatory process that involved all library employees, an academic health sciences library developed a Mission and Values Statement to formally express the library’s guiding principles. Leadership of the university libraries had asked each departmental unit to identify shared values in order to inform the creation of a broader values statement for the university library system. With this prompting, the [library name’s] statement was developed in collaboration with the library’s Diversity, Equity, and Inclusion (DEI) team.

Description: In late 2020, the library director constructed and disseminated a survey to all health sciences library employees, asking for the top three values they thought represented the library. This was followed by an all-staff virtual meeting where employees participated in breakout rooms to organize the suggested values from the survey into larger themes. In a second meeting, the agreed-upon themes were then discussed and further refined in different breakout groups, and draft descriptions were written for each theme. Working from these, members of the DEI team drafted a mission statement to articulate the overall purpose of the library, as well as the individual values that guide library employees in pursuing that mission. The draft statement was shared at an all-staff meeting and all employees were invited to provide written or verbal follow-up feedback.

Conclusion: The finalized statement was added to the library’s website. The five values identified were: Accuracy and Integrity, Community and Collaborations, Inclusion and Accessibility, Equity and Justice, and Lifelong Learning and Innovation. The overarching mission touched on each of these values, stating that “our mission is to improve health education and research by facilitating access to information and empowering individuals to critically assess evidence. As active partners in the education, research, training and clinical needs of our communities, we are committed to promoting equity, diversity, inclusion, and justice in healthcare.”

Forging Ahead Together: Facilitating Research Partnerships through an Annual Systematic Review Education Series

Track: Education | On Demand

Carrie Baldwin-SoRelle - Health Sciences Librarian & Liaison to Public Health, University of North Carolina at Chapel Hill

Rebecca Carlson, AHIP - Health Sciences Librarian and Liaison to the Eshelman School of Pharmacy, University of North Carolina at Chapel Hill, Chapel Hill, North Carolina

Background: Health science librarians see an ongoing need for systematic review (SR) skills among new and established researchers. To help meet the demand for review support, librarians at one institution created a two-day virtual workshop for researchers at the beginning stages of an SR to get training and hands-on assistance with reviews. This synchronous event met internal goals of efficiently using staff time while unifying a range of content under an SR skills umbrella. It also addressed external-facing goals of promoting the library’s expertise in SR projects, conveying the scope and methodological rigor of SR projects to researchers planning or beginning a review, and providing a home for SR researchers to network across a large campus.

Description: Liaison librarians at an academic health sciences library developed and iterated an annual summer workshop to train graduate students, post-docs, residents, and faculty on systematic review (SR) best practices. For both workshop occurrences, hosting the events on Zoom minimized costs and allowed for flexible scheduling, as researchers off-campus for the summer could still attend. The presentations by librarians and graduate assistants included processes, tools, and breakout sessions for questions and consults. Librarians with a range of SR experience contributed to the workshop. Staff newer to SRs presented on tools like citation managers and Covidence, and more experienced librarians presented on PRISMA guidelines, review methodologies, and automation tools. Interactive components of the presentations offered instant feedback. The best-reviewed addition to the second annual workshop was a panel discussion with experienced SR researchers discussing aspects of the SR process that aren’t usually under the purview of librarians. A graduate student, researcher, and two faculty members all discussed their experiences conducting and teaching systematic reviews and, of particular value, highlighted the benefits of partnering with librarians on the SR process and the value of an interprofessional research team. Overall, the workshop met staff and attendee goals for a large-scale introduction to systematic reviews.

Conclusion: The workshop was well-received, as evidenced by attendee feedback and subsequent higher attendance at repeated events. A major benefit for library staff was delivering content to 79 attendees simultaneously instead of one-on-one, saving at least 35 hours of staff time. Survey responses and attendance tracking from the first year informed the content prioritized for the second workshop, yielded a mini version of the workshop during the school year, and offered feedback on a library guide that was undergoing revision. Librarians who also teach course-incorporated review content adapted their materials and teaching strategies from the workshop for the classroom. Future opportunities for expansion include a weekly workshop series, multiple tracks for researchers with different experience levels, and facilitating networking opportunities for attendees.

From Ad-Hoc to Streamlined: Establishing an Infrastructure of Documentation and Support for Systematic Review Services

Track: Information Services | On Demand

Amelia Brunskill, MSIS - Assistant Professor & Information Services and Liaison Librarian, University of Illinois Chicago, Chicago, Illinois

Rosie Hanneke, MLS, AHIP - Associate Professor & Head, Information Services and Research, Library of the Health Sciences, University of Illinois Chicago, Chicago, Illinois

Background: Working with faculty and students on systematic reviews, we have found that users come to us with widely varying levels of experience with conducting reviews. Their expectations about the level of the support that we’ll be able to provide is similarly wide-ranging. As such, we decided that it was important to provide more explicit documentation for the review process in order to limit opportunities for miscommunication and provide a more streamlined approach, thereby improving the process for all parties involved.

Description: We, as the co-chairs of the library’s Systematic Reviews Working Group, teamed together to create language and tools to provide more clarity about the support we can provide, a clear snapshot of the researcher’s plan for the review, and tools users can adopt after an initial consultation. We outlined two tiers of support for evidence synthesis projects, the first a consulting model and the second an in-depth collaboration model. We outlined these on our library’s systematic reviews LibGuides page. For users interested in the second-level tier support, an online intake form was created, adapted from another library’s Creative Commons-licensed form, that asked users to provide detailed information about their planned review. Upon completion, this form would be automatically sent to the larger working group for dissemination to the relevant liaison librarian. We also created a spreadsheet for documentation of the search strategy creation and testing process, with embedded instruction about how to use the different sheets. The larger working group, and a group of health science liaison librarians, were consulted for feedback along the process for all created materials.

Conclusion: The language on the website has gone live and a link to the intake form has been added. Liaisons have begun to use the form with faculty and graduate students in their liaison departments. Initial reports are that both users and librarians are pleased with having this information collected and presented in an initial document. The spreadsheet has also been provided to users, who’ve expressed appreciation for having clear next steps outlined for them to follow. We feel that this work represents clear positive progress towards a comprehensive system of documentation and support resources, benefiting librarians and review teams alike.

Getting Evidence into Clinical Practice: A New Role for Librarians

Track: Clinical Support | On Demand

Lara Handler - Clinical Librarian, American Board of Family Medicine, Carrboro, North Carolina

Background: The American Board of Family Medicine is one of 24 specialty medical boards in the United States. The Board’s mission is to improve the health of the public through Board Certification, Residency Training, Research, Leadership Development, and promotion of the specialty. As part of the certification process for over 100,000 physicians, various activities are offered. A National Journal Club (NJC) was launched in 2021 to help physicians earn certification points, stay current with medical literature, support shared decision-making, and advocate for their patients with subspecialists, health systems, and payers. The Clinical Librarian played a unique role in the creation and evaluation of the NJC, showing that librarians can contribute in new and innovative ways alongside physicians and project management.

Description: Reviewed and curated by a national committee of practicing family physicians with expertise in evidence, the pilot was launched in summer 2021 and initially featured 45 articles that were evaluated and

ranked according to relevancy, methodological rigor, and impact on practice. The Clinical Librarian was a key part of the team that designed, planned, and coordinated the creation of the NJC. This new project incorporated several novel aspects for a journal club, such as providing free full-text PDFs to all participants, having a multi-tiered system of selecting articles, and including a Clinical Librarian with evidence-based medicine and critical appraisal expertise. The Clinical Librarian’s role was integral to the project, from helping develop a system to identify articles, ranking the articles based on pre-selected criteria, managing copyright and publication issues, and participating in evaluation and improvement of the project.

Conclusion: After rapid uptake- over 125,000 participants in the first 15 months- and overwhelmingly positive feedback, the Board decided to continue the NJC, which remains available to all members seeking certification or to enhance their own knowledge/skills. Two years after the pilot, there are four releases, including 200+ highly-ranked articles. In feedback collected, 95% of participants say they feel more comfortable reviewing journal articles, and 66% of participants intend to or are considering making changes in their clinical practice. The Clinical Librarian played a unique role serving on the leadership committee to launch this new and innovative way of supporting physician access to the latest evidence-based articles that are methodologically rigorous, highly relevant, and have the potential to improve practice.

The Hobgoblins of Our Massive Minds: The Survey on LIS Curriculum from the 2022 UNYOC Conference. The Panel, Their Conclusions, and Our Next Steps

Track: Education | On Demand

Jaimi McLean - Librarian, Miner Library, Rochester, New York

Background: In October 2022, a panel discussion was held at UNYOC's regional conference regarding MLIS education content, and its relevance and applicability to health sciences librarianship. The panelists included an early career librarian (recent graduate or ≤2 years in the field) and a veteran librarian (≥5 years of experience in the field). This presentation will revisit the questions presented in October and propose a course of action for further research to support MLIS curriculum development.<

Description: After multiple water cooler chats with colleagues and two years in the profession, I wanted to assess our mandatory education empirically. A survey was sent to various MLA listservs inquiring about our undergraduate background, graduate coursework, mentorship, continuing education credits, skill development post-grad, and practical skill transition.

Conclusion: The results revealed a great majority of medical librarians with a background in the liberal arts and a consensus on mandatory skills that are regularly developed after graduate studies on the job. The intention is to bring awareness to this survey and encourage participation in another survey in Fall 2023. A goal would be to present these findings to the MLA and ALA to encourage refinement of the MLIS curriculum and development of curriculum for a health science specialty and/or certificate to ensure consistency and empirical standards for our emerging health science librarians.

How Did a Caribbean Library Association Bring the Caribbean Region and North America Together?

Track: Health Equity & Global Health | On Demand

Ayaana Alleyne Cumberbatch - Librarian, The University of the West Indies Medical Sciences Library, St Augustine campus, Trinidad and Tobago

Background: In 2018, The Association of Caribbean University, Research, and Institutional Libraries, (ACURIL) ’s Virtual Health Libraries Content Area roundtable special interest group was established to create a forum to share and discuss issues and create projects related to Virtual Health Libraries located in the Caribbean. Since the inception of this special interest group, many pre-conference sessions and workshops were held inviting presenters and attracting participants from Latin America and the Caribbean region. At these sessions, a representative from the Pan American Health Organization (PAHO) would present and discuss ideas with members. On May 16th, 2022, the pre-conference session welcomed for the first time a presenter from the National Library of Medicine (NLM) who presented alongside two representatives from PAHO.

Description: The chair of ACURIL ’s Virtual Health Libraries Content Area roundtable, approached the NLM’s then Chief in the Office of Engagement and Training (OET) and invited her to speak on ACURIL’s theme: Change Management and Resilience: Proactive actions in Libraries, Museums and Archives. In addition to agreeing to present at the pre-conference session, the NLM provided the technical facilities and support to make the online session possible. The presenter spoke on ACURIL’s theme, with her presentation entitled: Anticipating the 3rd century of the National Library of Medicine: Pivoting internally and evolving the network of the National Library of Medicine to achieve the 2036 vision. The chair’s invitation to the PAHO was also accepted and they agreed to offer two presentations. The first presenter from PAHO was the Director of the Department of Evidence and Intelligence for Action in Health PAHO and World Health Organization and the acting director of Latin America and Caribbean center on Health Sciences Information (BIREME- Portuguese acronym) PAHO / WHO. Following his presentation was the Coordinator of Information Technology infrastructure at the Latin America and Caribbean center on Health Sciences Information (BIREME- Portuguese acronym).

Conclusion: The pre–conference session was 100% free and available online to interested persons. The session was advertised online and attracted a total of fifty-six people attracting members and non-ACURIL members from Caribbean region, Latin, and North America. Attendees who would not have had the chance to attend or participate in previous sessions had the chance to join and benefit from this collaborative event. It was also an opportunity to bring together regional and international medical and health information professionals at an event hosted in the Caribbean region. Finally, it demonstrated the chair’s leadership skills to coordinate, innovate and network with colleagues, locally, regionally, and internationally by providing and hosting an event which encouraged the sharing of experiences and ideas.

Integration of a Librarian within Three Clinical Clerkships for Evidence-Based Medicine: Reflections and Future Directions

Track: Education | On Demand

Sa'ad Laws - Librarian, Education & Research, Health Sciences Library, Doha, Ad Dawhah, Qatar

Mai Mahmoud, M.D. - Associate Professor of Teaching in Medicine / Assistant Dean for Faculty Affairs, Medical Education

Moune Jabre - Assistant Professor of Clinical Obstetrics and Gynecology, Medical Education

Amal Khidir, MBBS - Associate Professor of Pediatrics, Medical Education

Ziyad Mahfoud, Ph.D. - Professor of Research in Population Health Sciences, Public Health

Background: Evidence-based medicine (EBM) has been identified as a critical component of medical education, integrating question formulation, information retrieval, appraisal, and application. The emphasis on information literacy makes EBM a natural area for librarian integration in medical curriculum; however, there is little consensus on how and where to do this. In this example, one librarian and three clerkship directors determined that the existing EBM curriculum was not extensive enough to afford students practical instruction in several areas, such as experience with multiple question types and appraisal of different study types. This lightning talk will explore how a librarian worked with clinical faculty to incorporate EBM into clinical clerkship courses, what occurred along the way, significant successes, and areas for improvement/future directions.

Description: Beginning with the Internal Medicine clerkship and then extending to Pediatrics and Ob/Gyn, the librarian and clerkship directors formulated a longitudinal curriculum. Students start by completing initial eLearning modules to introduce and refresh essential EBM knowledge. Further, students are required to submit initial iterations of EBM case projects drawn from their clinical experience. Both librarian and clerkship director supply formative assessment of skills such as PICO formulation and searching for evidence. Students are ultimately required to complete a summative assessment such as a written paper or group presentation.

Conclusion: Increased inclusion of a librarian within the EBM curriculum has produced beneficial effects, both instructionally and professionally. Students now gain greater EBM experience, which has improved their abilities to address different question types, medical specialties, study types, and other variations. Informal evaluations indicate that students find value in receiving formative feedback prior to summative assessment. Additionally, increased contact between librarian and faculty has improved faculty perceptions of librarians and resulted in additional collaborations in areas beyond EBM instruction.

Journal Assessment is Not All About Publisher Supplies Statistics!!

Track: Information Management | On Demand

Lutishoor Salisbury - Distinguished Professor, University of Arkansas Libraries, Arkansas

Background: Making collection management decisions of resources in academic libraries is important and challenging. These often occur in circumstances where the operating collection budgets are flat, and the costs of science resources are constantly increasing. Because of this, librarians undertake regular evaluations of their subscribed resources in order to provide the best resources to meet their patron needs. This presentation will identify and discuss factors beyond vendor supplied data and citation counts that should be considered when retention/subscription decisions are made for journals. Individuals will learn how to think about journal evaluation projects; appreciate the various uses that are made of current journals; and apply quantitative and qualitative information in assessments.

Description: This presentation reports on a project undertaken to identify the extent the University of Arkansas faculty are publishing in and citing journals from top vendors, to what extent they cite and publish in the same journals; provide a comparison of publisher data versus those titles that the researchers publish in and cite and identify the titles that are heavily cited but not in the radar for subscription.

Conclusion: In addition to providing information on the types of material and the top journals that the cite and use, the findings from this study will also provide data for evidenced based collection development beyond only COUNTER data. It will inform decisions in validating how well the library’s collections are meeting the research and instruction needs of their campus users.

Just Add a Librarian: Scholarly Communication Instruction Integrated into an Armenian Informatics Training Program

Track: Health Equity & Global Health | On Demand

Lynn Kysh, MLIS, MPP - Clinical & Research Librarian, Children's Hospital Los Angeles, Los Angeles, California

Tamara Galoyan - Assistant Research Porfessor, Drexel University

James Dickhoner

Armine Lulejian, EdD, MPH, CHES - Assistant Professor, Keck School of Medicine at the University of Southern California

Background: Health information technologies including electronic health record systems and telehealth services are being widely implemented and have become essential in the wake of the COVID-19 pandemic. Many low- and middle-income countries (LMICs) are in urgent need for training of health informaticians to support best practices and innovation in the domains of clinical care, public policy, translational research, and health informatics. With a national commitment, Republic of Armenia is poised to leapfrog the systems in the United States and Europe if there is a workforce to build the necessary infrastructure.

Description: A librarian joined a team in developing and running the first health informatics training program in Armenia and in the Caucasus region. The training program consisted of four educational pillars: a bootcamp, an individualized training program, a capstone, and a scholarly project. In this inaugural cohort, four fellows consisting of physicians and computer engineers completed three months of coursework followed by guided mentorship. Librarian instruction included lectures, discussions, and consultations to guide participants through seeking informatics literature and disseminating the results of their scholarly project. Each session of the bootcamp was evaluated for knowledge impact, content, organization, increase of confidence and skills, among others. Qualitative interviews informed understanding of gaps and provided needs assessment.

Conclusion: Overall, bootcamp increased knowledge in most of the modules. Confidence and skills were positively associated with knowledge gained. Qualitative interviews revealed the value of mentorship through a capstone and scholarly project. Librarian instruction was well received and effectively connected participants with scholarly communication opportunities. Lessons learned for improvement include building effective discussions with participants whose primary language is not English and building in dissemination instruction earlier in the coursework to meet potential submission deadlines.

Learning On the Job: Using Artificial Intelligence and Natural Language Processing to Support Rapid Review Methods

Track: Innovation & Research Practice | On Demand

Leah Hagerman - Research Coordinator, National Collaborating Centre for Methods and Tools, Hamilton, Ontario, Canada

Sarah E. Neil-Sztramko - Knowledge Translation Advisor, National Collaborating Centre for Methods and Tools

Maureen Dobbins, PhD - Scientific Director, National Collaborating Centre for Methods and Tools, Hamilton, Ontario

Background: The Rapid Evidence Service (RES) responds to the needs of public health decision-makers by answering priority questions using rapid review methods. The vast quantity of literature available makes it challenging to identify relevant research using manual screening under rapid review timelines. Our centre has integrated Artificial Intelligence (AI) to support screening for rapid reviews.

Description: Four AI features offered by DistillerSR have been integrated into our screening methods: DAISY Rank, which uses Natural Language Processing to learn manual screening patterns and apply those to the remaining references to be screened, thereby predicting which studies will be most relevant; Re-Rank Report, which predicts the total number of included studies based on previous screening patterns; AI Screening, which automatically screens studies based on prediction scores; and Check for Screening Errors, which identifies studies that were potentially falsely excluded.

Conclusion: We have used DAISY Rank on 30 rapid reviews on 18 topics, allowing full text screening and data extraction to proceed by identifying the most relevant studies early. Use of the Re-Rank Report and Check for Screening Errors functions have informed decisions to move to AI Screening. To date, we have integrated AI Screening into five rapid reviews, including three living reviews. In one rapid review update, AI Screening automatically excluded 5,744 of 7,196 studies, thus saving time in manual screening. Integrating AI into rapid review methods has saved time spent on manual screening, allowing reviews to progress faster and for staff to be re-allocated to other tasks. Our methods are transferable across most RES topics.

Leveling up Researcher Profiles: Changing Platforms, Changing Practice

Track: Information Management | On Demand

Heather J. Martin, MISt, AHIP - Director, System Library Services, Portland, Oregon

Amanda Schwartz - Digital Asset Librarian, System Library Services, Providence, Missoula, Montana

Background: When essential work-flow functionalities were sunsetting in an existing Researcher Profile platform, a large multi-state US health system Library looked for alternatives and decided to transition to a different platform with automatic upload capabilities and a more intuitive user interface. Due to the size and complexity of the health system enterprise, varied branding and naming conventions, and competing stakeholder needs, the Library faced challenges in planning, migrating, and implementation that may be unique in contrast to a traditional academic or stand-alone research center environment.

Description: Due to the increased cost of the new platform, the Library needed to find a funding partner and secure buy-in from stakeholder leadership. The Digital Asset Librarian and the Library Director reached out to Research leadership explaining the need for a replacement product and proposing a possible vendor solution. A representative from the Research department joined the project team and planning and negotiations quickly began in earnest. The team identified shared goals and where needs and priorities differed between the two teams. Careful evaluation of the product was carried out, and when it was determined the best-fit solution the team secured funding approval from leadership, initiated contract negotiations and an agreement eventually signed. Currently in the implementation phase, focus has been on building out the product in a way that meets the unique needs of complex health system, creating an original organizational architecture and taxonomy, and identifying researchers for inclusion in the profile platform. Challenges have arisen around technology requirements, consensus building amongst multiple stakeholders, and slow approval processes.

Conclusion: The contracting/implementation processes were slow due to the shared nature of the project.

However, it resulted in broader buy-in and positive relationship-building. Both groups are eager to pivot practices to release the new platform. The new platform will be launched in Q2 of 2023 with a focus on prioritizing prolific authors in order to create the most dynamic launch possible. The primary goal of the Library is to find a replacement profile platform with semi-automated scholarly record population. The primary goal of the Research department is to give researchers a way to search, discover and connect to Providence researchers. We anticipate that the launch will meet those objectives. Post-launch will focus on streamlining workflow and promoting engagement.

Librarians Facilitating Community Health Worker Certifications: Creating Partnerships with Academic Faculty and Healthcare Professionals

Track: Education

Meghan Davitt - Health Sciences Librarian, Shenandoah University, Winchester, Virginia

Background: Community health workers are health professions who facilitate access to services and improve the quality and cultural competence of service delivery. A recent change to Medicaid rules allows for the possibility of reimbursement for preventive services offered by community health workers, which may prompt further movement toward developing training and credentialing standards. As community health workers become more common in healthcare settings, health librarians should look for opportunities to collaborate with academic departments and healthcare professionals to ensure community health workers are trained in health and information literacies.

Description: An academic health sciences librarian and a nursing faculty who teaches a Community Health class began to collaborate on a Community Health Worker project with the regional health system. The health system had previously partnered with the Community Health class to provide some experiential education to students. With a new state certification for Community Health Workers available and an expansion of the Medicaid reimbursement rules, there is an opportunity for the university to implement a new program and the health system to gain certified employees. Meetings were held to discuss what requirements there were for each institution. To learn more about the challenges we might face, we then reached out to other organizations in multiple states that were beginning to start their own programs. Talk has now begun with our university administration on how to best move forward with this new program. The librarian’s role as faculty and liaison to multiple departments helps to create the bridges needed for this multi-disciplinary program.

Conclusion: We anticipate being able to offer this program next year. In the meantime, we will continue to develop the curriculum and collaborate with the regional health system to set plans in place. This includes creating a curriculum, building the framework for utilizing our online and in person classrooms, and developing the simulation lab experiences. This program has the possibility to create connections between multiple academic departments: the library, nursing, public health, physician assistants, pharmacy, and others. We hope it will also act as a “feeder” program for some community members who begin their health care careers with a Community Health Worker certificate and decide to continue in one of our degree programs.

Librarians' Electronic Resource Reviews Network (LERRN): A Free Citation Database for Resource Reviews

Track: Information Services | On Demand

Louisa Verma, AHIP - Reference Librarian, Portland Cement Association, California

Background: Reviews of electronic resources are a valuable way to identify potential databases and service platforms for libraries. Resource-focused articles assist librarians in learning about updates or useful features, help train others, and market to potential user groups. Resources of general interest to many types of libraries may be published in disparate journals and are not well collocated. Further, small or solo-staffed libraries may not subscribe to library and information sciences databases or journals that contain reviews of electronic resources. With this in mind, the main aim is to create a freely available citation database that brings together electronic resource reviews, overviews, comparisons, projects, or other evaluatory content in one place using free or low-cost means.

Description: To create the database, a list of journals was created from known journals, library association-related publications, DOAJ, and by searching on the JournalTOCs (UK) website for library and information science-related titles. Citations are initially gathered into a Zotero folder through imported RSS feeds. Relevant citations are given broad topic tags (e.g. science, technology, engineering, mathematics, medicine and general) and other tags and imported into Librarika online catalog records via a CSV template. The current database includes citations back to 2019 and focuses on electronic resources in the areas of science, technology, engineering, mathematics, and medicine. Prior to importing, citations are given four tag types to assist with browsability in the Librarika database (year, resource, topic, article type). Content is selected based on its value for assisting with electronic resource purchasing, training, marketing, or use. In addition to reviews, article types also include overviews, comparisons, projects, search tips, book reviews and general electronic resource management (ERM) articles. The database is updated quarterly in January, April, July and October. A website (https://www.lerrndb.com) was created to highlight the rationale, scope, and update frequency of the database. A list of journals indexed is also available on both the website and Librarika (https://lerrn.librarika.com/search).

Conclusion: The LERRN database is still in its early stages of development and, as of June 2022, has only been introduced to a handful of librarians. From June 2022 to mid-December 2022, the database has garnered nearly 4000 OPAC page views and includes over 500 articles (a majority published from 2019 to the present.) It is the author's expectation that as content continues to be added to the database and more librarians become aware of it, it will become a useful tool for librarians who are looking to learn about new resources for potential acquisition or update themselves on resources they currently own in order to more readily market to and provide training to their users.

Librarians’ Role in Developing International Pediatric Clinical Guidelines

Track: Information Management

Hannah J. Craven - Research & Scholarly Communications Librarian, Ruth Lilly Medical Library, Indianapolis, Indiana

Elizabeth C. Whipple, MLS, AHIP - Assistant Director of Research and Translational Sciences, Ruth Lilly Medical Library, Indianapolis, Indiana

Background: Due to a library’s liaison program, a pediatrician had an established working relationship with two librarians already and utilized our evidence synthesis expertise. In fall of 2019, the liaison librarians joined an international team of physicians, respiratory therapists, and nurses to establish the first international clinical practice guidelines for pediatric ventilator liberation. One of the aims of this project included establishing consensus-based recommendations for methods to assess when a pediatric patient is

ready to be liberated from mechanical ventilation. When consensus was not reached, a systematic review needed to be conducted. This project demonstrated the importance of librarians in the development of practice guidelines and furthers the practical impact librarian expertise brings to clinical care.

Description: Upon joining the team, the librarians helped develop workflows and data management for the different research questions. Eight of the PICO questions failed to reach consensus and each required a systematic review. The librarians created two reusable filters that repeated across all PICO questions (pediatrics and ventilation). The searches for the unique PICO concepts were created by working closely with smaller groups of specialists. The librarians divided the searches amongst them, while also working closely with one another to build and review the searches. Typical evidence synthesis services were provided as well such as retrieving full text and citation management. As an integral part of the team, the librarians attended recurring administrative meetings, international conferences, and worked directly with the principal investigators when challenges arose.

Conclusion: Including librarians on the team showed many medical professionals the skills required for a truly exhaustive search. A portion of salary savings for the library was a benefit of this grant-funded project. Three papers and counting have been published (executive summary, definitions, and a meta-analysis) with the librarians as coauthors. The executive summary received laudatory comments from the journal editor, praising its importance to the field and unique methodology. Due to the successful working relationship, the librarians have been asked to participate in another clinical guidelines project. The librarians are in the process of archiving the searches in SearchRxiv to ensure digital preservation of their searches, disseminate their work, normalize sharing of searches, and highlight the importance of high-quality searches.

Literally Healing: Evaluation of Therapeutic Titles in a Pediatric Hospital

Track: Clinical Support | On Demand

Kyle Horne - Program Manager, Literally Healing / Children's Hospital Los Angeles, Los Angeles, California

Lynn Kysh, MLIS, MPP - Clinical & Research Librarian, Children's Hospital Los Angeles, Los Angeles, California

Background: Pediatric clinicians will likely encounter therapeutic children’s books with topics ranging from childhood development, particular diagnoses, and difficult events including death and dying. Studies have demonstrated that such therapeutic titles counter pervasive clinical problems including low health literacy, anxiety, and limited time between clinicians and patients. The current consensus is that therapeutic books for young people have value and should be incorporated into all clinical practice. However, not all books are created equal. There will always be variation in the quality of published books and while there are many existing awards that can point clinicians to titles based on literary merit there is no such award or national committee focused on determining the therapeutic value of titles.

Description: “Literally Healing,” the book gifting program at Children’s Hospital Los Angeles, recognized a need to have a panel of reviewers determine if a title would be appropriate for use in its therapeutic library. As a result, the interprofessional Therapeutic Review Committee was formed to read, rate, and discuss all therapeutic books and then determines if we will accept, accept with conditions, or decline them. When reviewing titles each committee member will read the book and provide a score of 0 to 3 within four sections with a total of 12 subsections. The four sections are illustration, story, therapeutic/medical accuracy, and provenance. Some of the subsections include inclusive illustrations, child-friendly therapeutic content, and

story length (per developmental age). Notes are then written by each clinician. While these points serve as a guide in then discussing the titles as a committee, they do not always guarantee that a book is recommended or not. This is because different weights are given to each category. The result of this review process is a database of therapeutic titles that includes both ranking and relevant metadata that can be accessed by hospital staff when consulting with clinicians, patients, and families.

Conclusion: Over the 2022 fiscal year, the committee reviewed 152 titles of which 63 were accepted, 65 were declined, and 24 were accepted with conditions. As a result, Literally Healing supported over 75 clinicians across the organization, gifted 2,800 therapeutic books, and completed 400 consultations. The next step for this database is through collaboration with the health sciences librarian and Literally Healing to create a beta version for a national audience that will allow for searching, browsing, and filtering by subject, age, use, language, etc. This will be designed and tested locally for a population of users that will include clinicians, educators, counselors, librarians, etc.

Machine Learning-Assisted Screening Increases Efficiency Of Systematic Review

Track: Information Management | On Demand

Eitan Agai - Founder, headquarter, St Petersburg, Florida

Riaz Qureshi, n/a - Assistant Professor, University of Colorado Anschutz Medical Campus

Background: Problem statement: Conventional systematic review (SR) methods are time-consuming and highly resource intensive. Artificial intelligence (AI), machine learning, and deep learning can help reviewers complete these tasks in less time and with fewer resources.

Description: Description: PICO Portal (PP) prioritizes articles for review using natural language processing and active learning to monitor user decisions on eligibility during title/abstract screening. To predict eligibility, PP uses several algorithms including both decision tree (e.g., SVM, Random Forest Naïve Bayes) and deep learning (e.g., Bidirectional Encoder Representations (BERT)) models. We tested the system on eight completed conventional SRs (i.e., two independent screeners for title/abstract and full-text eligibility) with a total of 55,812 records (range: 4,204 to 14,188), and on a range of topics from social to biomedical sciences. For each case, we simulated the screening using 100 articles to train and build predictions for eligibility, re-ranking the next 100 articles, and continuing through the records comparing the predicted eligibility with the actual results. We found that with the active AI predictions, reviewers needed to screen only 9-40% of title/abstracts to capture 95% of eligible articles (Figure).

Conclusion: Lessons learned: Using five SRs as case studies, we show that 40-60% of screening effort can be saved using PICO Portal, an AI-assisted, web-based, SR platform. In one SR, reviewers screened 3600 records to achieve 95% of the predicted abstract includes, but when compared to the predictions and the adjudicated decisions, they had identified 95% of included articles after screening only 600 title/abstracts (16%). Future research should examine the impact of missing the final 5% of articles on review conclusions and assess the resource-benefit ratio.

Managing Systematic Review Projects: A Guide for Librarians

Track: Information Services | On Demand

Dani LaPreze - Clinical Librarian, Kornhauser Health Sciences Library, Louisville

Ansley Stuart - Clinical Librarian, Kornhauser Health Sciences Library

Background: This presentation and guide will give librarians the tools they need to help corral research projects that are too large for small research teams, while maintaining adherence to review guidelines. Attendees will leave this presentation with answers to questions commonly asked by researchers regarding why and how advanced long-term review projects are performed the way that they are, and a roadmap to help guide these projects.

Description: There is a growing interest among researchers and academics to publish systematic reviews. Often researchers will set out to publish a systematic review without having previously worked with a long-term review team or having a firm grasp on what all the review process entails. Helping to guide these novice researchers and manage these types of review projects is a growing role for librarians. The role of the librarian in these types of projects is multi-faceted, where they regularly act as facilitators, organizers, managers, voices of reason, and more. In this presentation, a guide will be provided for librarians that they can use to mentor inexperienced authors over the multi-month review process. This guide provides instruction on taking researchers through the entire review process from project organization and developing a fully formed research question to publication. This guide also discusses the stages of managing long-term review projects, as well as how to guide researchers to alternative reviews that could better suit the project. The presentation recommend specific entities for review registration, guidelines to follow, databases for literature searching, software to use in the review process, citation management programs, and places to publish, based on the generalized topic of the systematic review.

Conclusion: Measurable outcomes for this project include heightened understanding of the review process by researchers, enhanced ability to guide researchers by librarians, and overall improved collaboration and understanding of different roles played during these projects between research teams and librarians.

Mental Health First Aid (MHFA): Why and How?

Track: Professionalism & Leadership | On Demand

Mary Margaret Thomas - Clinical Education Librarian, Hardin Library for the Health Sciences, Iowa City, Iowa

Background: Because the COVID-19 Pandemic exacerbated behavioral health conditions and highlighted the need for more behavioral health advocacy/interventions, the workplace training program Mental Health First Aid (MHFA) may be of interest to health sciences libraries. MHFA training can help libraries educate staff, destigmatize mental illness, and provide de-escalation techniques to better meet their community’s needs. This talk will provide a detailed description of what MHFA training is, how it can be implemented at your library, and the benefits of the training.

Description: MHFA is a standardized curriculum that teaches lay people how to respond to mental health crises and provides background education regarding mental illnesses. It is an eight-hour educational workplace training program taught by certified instructors. Just as CPR training teaches participants how to assist an individual having a heart attack, MHFA teaches participants how to assist someone experiencing a mental health or substance use-related crisis. During MHFA, participants learn risk factors and warning signs for mental health and addiction concerns, de-escalation strategies for both crisis and non-crisis situations, and where to turn for help. MHFA includes many interactive learning opportunities that make applying the skills learned in a real-life situation easier such as role-playing and scenarios. Anyone can be certified as an MHFA instructor, by completing the instructor training course. Once certified, they can begin training others.

Conclusion: MHFA is a natural fit for libraries since a large part of the training is devoted to teaching participants how to provide reliable mental health information, resources, and appropriate behavioral health treatment options. It is relevant and appropriate for all library staff, especially public-facing staff since it gives them skills to better cope with challenging public interactions. Additionally, there is a wide range of specialty MHFA trainings, depending on the target audience; for instance, there is one for Higher Education.

The MLA Style Guide: Looking Back to Move Forward

Track: Information Management | On Demand

Katie Arnold, Copy and Production Editor, Medical Library Association

Ellen Aaronson, MLS, AHIP - Librarian, Mayo Clinic Libraries, Rochester, Minnesota

Skye Bickett, AHIP - Reference and Education Librarian, Philadelphia College of Osteopathic Medicine - Georgia Campus, Suwanee, Georgia

Michelle Kraft, AHIP, FMLA - Library Director, Cleveland Clinic, Cleveland, Ohio

Jenessa M. McElfresh, AHIP - Systematic Review Service Coordinator / Senior Research & Learning Services Librarian, University of Tennessee Health Science Center Health Sciences Library, Memphis, Tennessee

Beverly Murphy, MLS, AHIP, FMLA - Assistant Director, Web Content & Development; DUHS Hospital Nursing Liaison: Watts CON Liaison, Duke Medical Center Library & Archives, Durham, North Carolina

Background: The MLA Style Guide is considered the primary resource for publishing guidelines for MLA print and electronic material. The goal of this revision is to better reflect currency, sensitivity, publication styles, authors' voice, and be more accessible to users. Following discussions with the JMLA Editorial Board in late 2020, it was suggested that a group be created to evaluate how the guide (then called "manual") could be updated for the modern era. The last revision was in 2019.

Description: In January 2021, the MLA Style Guide Work Group convened with members from the JMLA Editorial Board and the MLA publications teams. A survey was created and administered to those involved with JMLA including editors, peer-reviewers, and all publishing arms of MLA. The Work Group sought data on awareness of the guide, barriers to usage, and suggestions for improvement. The Work Group met virtually twice a month, evaluating each section of the existing guide, and making recommendations for revisions. The JMLA Equity Work Group was consulted to ensure that the guide was representative of current language and DEI practices.

Conclusion: Survey results revealed accessibility and currency issues, a knowledge gap in the existence of the guide, and a need for respect and sensitivity in language. When completed, the revised guide will become a dynamic and interactive resource accessible through MLANET and reviewed on a regular basis to ensure currency.

Nonbinary Genders in Taxonomy and Research Data

Track: Information Management | On Demand

Ari Gofman Fishman - Research & Content Management Librarian, Analog Devices Inc Research Library, Wilmington, Massachusetts

Nina Exner, PhD, MLS - Research Data Librarian, Virginia Commonwealth University Libraries, Richmond, Virginia

Sam Leif - Assistant Director, University of Nevada, Las Vegas

Background: Existing studies estimate that between 0.3% and 2% of adults in the U.S. (between 900,000 and 2.6 million in 2020) identify as a nonbinary gender or otherwise gender nonconforming. In response to the theme of “Looking back, forging ahead”, this lightning talk examines the existing strategies for representing nonbinary people in datasets and suggests options for how researchers, librarians and administrators can approach the curation of this data at each stage of the research lifecycle moving forward. This talk is scoped to discuss research data administration and taxonomy practices, not clinical encounters and medical records.

Description: In this session, we examine some of the known challenges of gender inclusion in datasets and summarize some solutions underway. Using a critical lens, we examine the difference between current practice and inclusive practice in gender representation, describing inclusive practices at each stage of the research lifecycle from writing a data management plan to sharing data. Results: Data structures that limit gender to “male” and “female” or ontological structures that use mapping to collapse gender demographics to binary values exclude nonbinary and gender diverse populations. Some data collection instruments attempt inclusivity by adding the gender category of “other,” but using the “other” gender category labels nonbinary persons as intrinsically alien. Inclusive change must go farther, to move from alienation to inclusive categories. We describe several techniques for inclusively representing gender in data, from the data management planning stage, to collecting data, cleaning data, and sharing data. To facilitate better sharing of gender data, repositories must also allow mapping that includes nonbinary genders explicitly and allow for ontological mapping for long-term representation of diverse gender identities.

Conclusion: Conclusions: A good practice during research design is to consider two levels of critique in the data collection plan. First, consider the research question at hand and remove unnecessary gendering from the data. Secondly, if the research question needs gender, make sure to include nonbinary genders explicitly. Allies must take on this problem without leaving it to those who are most affected by it. Further, more voices calling for inclusionary practices surrounding data rises to a crescendo that cannot be ignored.

NOT-NOT? Yes!: A Visualization Tool for an Advanced Search Technique

Track: Education | On Demand

Basia Delawska-Elliott, MLS, AHIP - Medical Librarian, Providence System Library Services, Portland, Oregon

Melinda C. Davies - Associate Research Informationist, Kaiser Permanente Center for Health Research Evidence-based Practice Center, Portland, Oregon

Background: The objective of this program is an in-depth slides-based presentation of how the NOT-NOT device works, how to assess if the device is appropriate for a searcher's need, and guidance on how to use it. The presentation will serve as a reference tool that viewers can return to when they need to consider the NOT-NOT device. Used in systematic searching to reduce recall and improve specificity, NOT-NOT is effective at eliminating specific population groups without excluding desired groups that overlap. Although NOT-NOT has been explained in handouts and webinars, the device can be confusing. The NOT-NOT device employs a nuanced use of Boolean operators and concept nesting. Using it requires knowledge of searching, and a correct technical application.

Description: We have observed through conversation and within expert search focused training that many searchers struggle to understand the device. Furthermore, there is an unease about using the device when the training we receive advises against using NOT in comprehensive searching. The device is applied in very limited circumstances, primarily to exclude non-human studies or to target specific age groups. There could potentially be a much wider use of the device if it were better understood. Studies show that visualization enhances learning. In this project we combined visualization and a precise search example to illustrate the effectiveness of NOT-NOT and increase searchers’ confidence. To deliver an effective presentation, we explored a number of approaches for describing the device and how it works. This work included testing several search strategies before selecting one that fit the criteria and would allow for a simple explanation. With the strategy in hand, we built a visualization including a diagram and a narrative legend (an infographic). We are implementing the program by offering the training as a lightning talk to an audience of experienced literature searchers. We hypothesized that learning and confidence would be enhanced with the right visualization including a search and an infographic.

Conclusion: The infographic developed as a result of our project is meant to be a teaching tool. Our goal is to launch the tool as a presentation and deploy it after as a survey. The efficacy will be evaluated with a pre- and post-test measuring searchers’ level of comfort with and understanding of the NOT-NOT device, and reported when the survey is concluded.

Opportunities for Resource Sharing in the Era of Mandated Data Management

Track: Information Services | On Demand

Kevin O'Brien, MLS - Head, Access to Resources Department, UIC Library of the Health Sciences, Chicago, Illinois

Background: The goal of this lightning talk is to introduction the topic of the challenges presented to resource sharing practitioners by the increasing prevalence of supplementary files, including data sets, accompanying biomedical journal literature to a large audience of biomedical librarians.

Description: The traditional interlibrary loan workflow excels at sharing returnable items and discreet non-returnable items, like journal articles and book chapters. However, both the workflow and the legacy resource sharing applications used by practitioners are not adequate to the task of managing requests for supplementary files included with version of record published articles. A move to a repository model standard for supplementary data availability, similar to the new NIH data management mandate, would facilitate sharing by making it easier for the borrower to obtain files outside of the resource sharing process. Librarians interested in improving resource sharing will benefit by raising awareness of this issue among peers and library users. This talk will contribute to that task by reviewing library literature on the topic and introducing this discussion to a large audience of biomedical librarians.

Conclusion: Heightened awareness of the challenges presented to resource sharing practitioners by the increasing prevalence of supplementary files will contribute to fruitful discussions of potential solutions to the problem by potentially influencing librarians, library users, publishers, and resource sharing application developers.

Partnering for Growth: How Medical Librarians Joined Special and Academic Librarians to Plan a Joint Conference

Track: Professionalism & Leadership | On Demand

Rebecca J. Morgan - Health Sciences Librarian, University of Kentucky Medical Center Library, Lexington, Kentucky

Stephanie M. Henderson - Nursing Liaison Librarian, University of Kentucky Medical Center Library, Lexington, Kentucky

Gina Genova - Clinical Librarian, University of Louisville Kornhauser Library, Louisville, Kentucky

Background: After struggling with declining membership and decreased networking opportunities, the Kentucky Medical Library Association (KMLA) joined the Kentucky Library Association Special Libraries Roundtable, the Kentucky Association of College and Research Libraries, and the Special Libraries Association - Kentucky, to plan a joint conference. The goal of KMLA's participation was to boost medical librarian involvement in state library initiatives, improve organizational finances, and improve collaboration between state medical librarians and librarians of other specialties.

Description: Two volunteers from KMLA were involved in all conference planning processes which included attending meetings and site visits and selecting and booking a keynote speaker. We considered KMLA involvement a success if 1) there was significant involvement and attendance by KMLA members, 2) our participation generated more money than we spent on the keynote speaker, and 3) our members reported that they benefited from participation.

Conclusion: Ultimately, our participation was a success. Nearly 50% of KMLA members attended the conference and presented lightning talks. We also earned a profit of approximately $800. Most importantly, members reported that they made connections with colleagues with whom they might not otherwise interact. Not only were our members able to apply what they learned at the conference to their own work, but members were also able to advocate on behalf of health sciences libraries and demonstrate commonalities between our work and the work of other librarians. As a result of this successful collaboration, KMLA is continuing to help plan the next joint conference in March 2023 and this presentation will report on the outcome of this second conference, as well.

Patents and Market Research: Librarians Partnering to Assist Bioengineering Senior Design Teams

Track: Education | On Demand

Meredith Futral - Business Librarian, R. M. Cooper Library, Clemson University, Clemson, South Carolina

Background: Business and engineering librarians have partnered to create a two-step, efficient process to assist Bioengineering students in understanding patents, patent searching and market research. The team approach was used to provide instruction and consultation meetings with these students. A two-semester Bioengineering Senior Design program matches teams of students with regional clinicians to develop biomedical devices that they research, design, prototype, and test. In the first semester of Bioengineering Design Theory, teams conduct a market analysis and identify competitors. They then search for existing related patents and reflect on how prior art could impact patentability of their devices.

Description: The business and engineering librarians were invited to lead a workshop on market analysis and patent searching for bioengineering students. The business librarian provided instruction in market research and the engineering librarian focused on patents. The business and engineering librarians visited two sections of the bioengineering class during the fall and spring semesters. A BioE 4010: Senior Design course guide with patents and market research pages served as the starting point for instruction. Following the in-class lectures, student teams were required to meet with both the business and engineering librarian to receive more tailored assistance with their market research and patentability assignments. These consultations were done independently. Students scheduled appointments using scheduling software. These meetings took place online or in person. If the teams did not include a brief synopsis of their devices when scheduling their meetings, the librarians reached out to the teams to request this information, to be better prepared for the team consultations.

Conclusion: Project Outcome was used to assess the initial whole class lecture sessions. There was positive feedback from the students and faculty. The students indicated that they learned something new to help succeed in their class and felt more confident about completing their assignment. The bioengineering professor noted that it was evident that the instruction and consultations improved the student’s learning and content outcomes. The business and engineering librarians would like to use what they have learned to continue to be involved with the bioengineering students and to become more involved with other senior design programs in the future.

Personal Statements: Supporting Med Students Through Workshops and Consultations

Track: Education | On Demand

Elliott M. Freeman - Research and Writing Librarian, LSUHS Library, Shreveport, Louisiana

Background: While medical schools often lack a dedicated writing center, medical students can still benefit from academic support in their development as writers. In recognizing an unfulfilled need, our library began to offer writing support, at first on an ad-hoc basis from librarians with a writing background and more recently through the hiring of a dedicated writing specialist. One of the largest needs identified was support for medical students composing personal statements for residency applications; these services have been by far the most utilized components of writing support.

Description: Our support program for personal statements is two-pronged, offering both an introductory workshop and one-on-one consultations. In 2021-2022, these services were transitioned to a dedicated writing specialist and recalibrated, drawing on writing center pedagogy. While both workshops and consultations offered guidance for surface-level features (grammar, formatting, etc.), they primarily emphasized higher-order concerns such as understanding the intended audience, developing a clear narrative, and illustrating points through salient storytelling. The workshop was offered multiple times throughout the 2021-2022 school year, with early-bird sessions in April and later options throughout August and early September in preparation for the opening of ERAS residency applications. Third year students were surveyed to determine optimal times; sessions were held in the evening, alternating between in-person and online workshops to maximize availability. Individual writing consultations were likewise available online and in-person. Consultations were offered from either the Research and Writing Librarian or an external scientific editor.

Conclusion: We saw a total of 25 students throughout our workshop sessions (17% of the class of 2023), and exit surveys universally rated the workshop as helpful. More students utilized the individual consultations, with a total of 68 students (~46% of the class of 2023); many were seen multiple times as they

continued to draft and refine their personal statements. Three topics emerged as particularly common and high-value areas to explore with students during drafts: articulating their interest in a specific specialty, developing clear examples to support their points, and understanding that they were applying to be both a trainee and a colleague.

Preparing Librarians and Researchers for the NIH Data Management and Sharing Policy

Track: Information Management | On Demand

Katy Smith, PhD, MLIS - Health Sciences Reference Librarian, Saint Louis University Medical Center Library, St Louis, Missouri

Dani LaPreze - Clinical Librarian, Kornhauser Health Sciences Library, Louisville

Nina Exner, PhD, MLS - Research Data Librarian, Virginia Commonwealth University Libraries, Richmond, Virginia

Background: In October 2020, the National Institutes of Health released the Final NIH Policy for Data Management and Sharing (DMSP), effective January 25, 2023. In June 2022, a group of concerned librarians and data scientists from across the United States joined together and created a virtual working group to share knowledge, expertise, and enthusiasm and to produce open access guidance and educational materials for both librarians and researchers. This program will introduce the working group, outline the group’s self-determined charge and goals, and present an overview of the group’s outputs/final products.

Description: Over 50 stakeholders answered the initial call to action with the idea of reviewing the NIH DMSP and related resources to provide guidance for both researchers and research support staff. While some stakeholders expressed interest but elected to remain “listening” members, 26 stakeholders actively contributed. The contributing stakeholders determined to meet weekly with rotating days and times to accommodate varied and busy schedules, with members taking turns facilitating the weekly meetings. The contributing stakeholders also divided into seven committees, each tasked with a different product to support librarians--especially those with little to no data management knowledge and experience--and researchers. As the committees worked and the products took shape, two committees merged and others refined their targets with the final products including: a glossary of data management terminology for librarians, a DMSP checklist for researchers, a policy readiness checklist for librarians, a collection of example plans (good and bad), an FAQ on the policy, and a tool for finding repositories. In addition to creating these resources, contributing stakeholders provided peer evaluation and advice and publicized the group’s efforts and products.

Conclusion: The products of the working group are now freely accessible via the Open Science Framework and linked from the NNLM NCDS Toolkit. The resources have been well-received: the folder is on track for over 1000 unique visits in the month of January 2023 and over 175 downloads have been completed. While the charge of the working group has been accomplished, the working group is aware that situations may change as the policy officially goes into effect. The group plans to continue to meet, albeit less frequently, to ensure that support for stakeholders, particularly librarians, remains current and correct.

Redesigning LibGuides for Better Student Support

Track: Education | On Demand

Lynn Warner - Assistant Librarian, Research and Health Sciences, Harrison Health Sciences Library, University of Cincinnati

Background: LibGuides are an essential resource, especially for nursing students, who often have significant research needs. Upon starting a new role as a Health Sciences Librarian at a large Midwestern university with high research activity, it became apparent to the author that the Nursing LibGuide, while heavily used as a portal to library resources, had become unwieldy, difficult to navigate, and full of unused information. This presentation will describe the process of overhauling a messy LibGuide and creating a streamlined, efficient new resource.

Description: After conducting a literature review on LibGuide user experience and best practices, the author utilized the ADDIE (Analyze, Design, Develop, Implement, and Evaluate) instructional design model to reorganize the Nursing Resources LibGuide. This presentation will describe the process of turning a bloated information source into a structured resource for logical student use.

Conclusion: This project is currently in the evaluation phase. Already the streamlined guide has become more simple to maintain, reducing librarian workload. Statistics are showing increased use of the guide, and user experience studies are planned to fully evaluate the updated resource. User feedback will also guide future updates, in order to keep the LibGuide up to date.

A Service to Assist Researchers in Finding Funding

Track: Information Services | On Demand

Merle N. Rosenzweig, MALS - Informationist, Taubman Health Sciences Library, University of Michigan, Ann Arbor, Michigan

Paul Barrow, MLIS - Foundations and Grants Librarian, University Library, Ann Arbor, Michigan

Paul Grochowski, MLS - Senior Associate Librarian, Science and Engineering, University Library, Ann Arbor, Michigan

Jacqueline Freeman, MLIS - Informationist, Taubman Health Sciences Library, Michigan

Judy Smith - Librarian, Health Sciences, University Library, Ann Arbor, Michigan

Background: A core value of academic libraries is supporting the clinical and research needs of the members of its institution. Librarians and informationists routinely engage in collaborative efforts across academic units that further the research enterprise. Literature searches, presenting workshops, and supporting grant applications are some of the traditional manifestations of that engagement. Our librarians and informationists collaborated with our Office of Research (OoR) to develop a new service to support the research enterprise.

Description: Our Office of Research (OoR) conducted a survey asking researchers to identify their most pressing issue in moving their research forward. The majority of those responding expressed frustration with identifying funding relevant to their research. Armed with this information, OoR reached out to librarians and informationists to work towards a remedy to this situation. The collaborative effort landed on a service to assist institutional members with finding funding. The service is situated in the Library, and it is accessed via a Research and Funding Guide with a simple Contact Us form. Requests are sent to five members of the Library’s Grants Triage Team, and the team member with the greatest subject knowledge responds to the inquiry. Consultations may draw in other experts from within the Library, or sometimes from the campus.

Conclusion: The Research and Funding Guide has seen a steady increase in activity over its seven years.

The Library Grants Triage Team has changed membership over the years, but diverse subject expertise has been a hallmark of the group throughout. The Consultation serve through the Research and Funding Guide has also seen a steady increase in activity over the seven years since the guide was launched and is well received by the research community. Above all, the service is fully integrated into the research enterprise across the institution.

Student Engagement Interest Group - Preparing Students for the Library and Health Information Workforce

Track: Education | On Demand

Heather N. Holmes, AHIP - Associate Director of Libraries, MUSC Libraries, Charleston, South Carolina

Samantha Nunn - Project Manager, Network of the National Library of Medicine, Region 4, Salt Lake City, Utah

Background: The purpose of the Network of the National Library of Medicine’s (NNLM) Student Engagement Interest Group (SEIG) is to advance the mission of NNLM by sharing participating regional offices and center's (ROCs) student engagement-related activities and prioritizing those appropriate for national coordination. Additionally, the SEIG prioritizes students, high school age and older, from traditionally marginalized communities and those underrepresented in biomedical research when conducting student engagement activities. Since the creation of the SEIG, the group has explored how NNLM can support students in acquiring experience in a professional setting, gaining access to mentors who can guide them in preparation to enter the library and information workforce and develop knowledge and skills to become better versed in health information resources.

Description: The SEIG consists of 10 members that meet once a month to identify and develop NNLM-wide strategies and metrics to advance student engagement, implement and evaluate strategies in increasing student engagement across the country, including those from underrepresented populations in biomedical research, and guide the development of programs that enhance skills of students in NLM resources and interest within the health professions, including health sciences librarianship. Some examples the SEIG has discussed for promoting student engagement are sharing project ideas that would benefit the intern or practicum student’s overall experience and support their areas of interest, methods to engage with LIS and iSchool programs to recruit potential students for practicums and internships, and providing opportunities to acquire knowledge and tools for students that may not be able to obtain in their program. Recently, an NLM fellow partnered with SEIG to further develop a page on the NNLM website that lists opportunities available within each of the ROCs, along with contact information for students to access when looking for potential sites to do a project or internship.

Conclusion: The SEIG made great progress in supporting LIS/iSchool students in acquiring knowledge and experiences to better prepare them for the library and information field. This progress led to hosting practicums and internships across several ROCs. For instance, the group collaborated with Region 1 to create a plan on how NNLM can support the University of Kentucky students participating in the Alternative Spring Break. The committee also produced a draft of the student engagement handbook that NNLM ROCs can use as a guide to effectively recruit, host, and provide quality mentorship experiences for students choosing to spend their practicum or internship time with the NNLM program. The handbook is anticipated to be finalized and implemented in the coming year.

Supply and Demand: An Expanded Statistical Approach to Collection Assessment and Targeted Outreach

Track: Information Management | On Demand

Nicole Askin - Liaison Librarian, WRHA Virtual Library, Canada

Background: Usage statistics and patron demand are traditional means of assessing the coverage of a health sciences collection and identifying new titles for potential purchase. However, a much wider variety of library statistics can contribute to an understanding of the supply of resources on a particular subject, and the potential demand for resources on that subject. This project sought to bring together a broad set of statistics to get a holistic view on the allocation of collection resources. Simultaneously, these statistics provide insight into potential targeting of outreach initiatives to usergroups that have not previously been vocal in advocating for library resources.

Description: This project considered a range of statistical measures that could speak to two areas of interest. The first was the allocation of subjects within the collection (supply). This category included not only a breakdown of titles and collections spending by subject, but also considered comparisons to external bibliographies as well as venues in which patron research was being published. The second was the interest (real or potential) from library patrons on that subject (demand). This category comprised occupational measures of the library's patron base, their research activity, viewership measures for subject-specific portions of the library website, and interactions with library services - reference questions, literature search requests - according to subject. These varied statistics were compiled and analyzed for two purposes: to assess the supply of resources per subject as compared to demand, and to assess opportunities for specific patron groups to be targeted for general or service-specific outreach initiatives.

Conclusion: The analysis revealed several subject collections that would benefit from increased investment of collections spending, and several where existing supply was disproportionate to demand. This analysis can be considered in concert with other factors when deciding how to allocate collection dollars going forward. We also identified some anomalies around our website and our collections policy that are being addressed. Finally, we identified some specific patron groups that are good candidates for targeted outreach, and are including them in an ongoing outreach initiative.

Supporting Future Medical Authors and Scholars: Launching a Writing Center from an Academic Health Sciences Library

Track: Education | On Demand

Alison Rollins - Supervisory Librarian, James A Zimble Learning Resource Center, Bethesda, Maryland

Rhonda J. Allard, MLIS - Supervisory Librarian, James A Zimble Learning Resource Center, Bethesda, Maryland

Elizabeth Steinbach, PhD - Academic Writing Specialist, James A Zimble Learning Resource Center, Bethesda, Maryland

Background: For more than a decade, the Learning Resource Center had received requests for writing support. However, without training in writing pedagogy, librarians could not meet the demand for comprehensive writing support. As a result, in July 2021, with support of deans from each school, the university president approved a pilot program for an Academic Writing Specialist (AWS). A timeline for

implementation and measures of performance were defined and candidate interviews were conducted. In January 2022, a PhD in applied linguistics, with experience working in writing centers and teaching writing in undergraduate settings, was hired to spearhead the writing center pilot.

Description: With only one year guaranteed for the pilot program, and executive decisions required half way through the year in order to convert the program to permanent status, a quick launch was imperative to the success of the writing center pilot. Librarians leveraged existing relationships with university stakeholders to connect the AWS with subject matter experts who could inform the development and deployment of needs-based services, most notably instructional support, workshops, and consultations. Librarians also used their existing partnerships with faculty and subject matter experts to recommend writing workshops and programs. Existing library software was configured to implement writing center services including online consultation bookings, managing workshops, and collecting feedback surveys.

Conclusion: The program’s success, including consultation services, seminars, curriculum integration, user feedback, and workshops, exceeded initial goals. In August 2022 the AWS pilot program was converted to permanent status. Since launching, the writing center has a closer partnership with library outreach and instruction. Opportunities for cooperation include classes on literature searching and manuscript writing to meet curriculum requirements; referral of patrons; custom classes to support academic scholarship; cross-referrals; joint marketing; and supporting university Diversity & Inclusion efforts. The AWS is able to speak on some topics, e.g. ChatGPT discussions, with a writing professional’s insights, while librarians offer assistance with citations and copyright. This partnership has not come without challenges such as a learning curve for all stakeholders and increased workloads.

Teaching Systematic Review Methodologies: Practicum Development for Library Science Students

Track: Innovation & Research Practice | On Demand

Cayla M. Robinson - Health Sciences Librarian, University of Kentucky Medical Center Library

Background: The aim of this practicum is to teach a graduate library student the methodology of a systematic review and how to conduct a research project. The student will be listed as a co-author on a methodology review of systematic reviews in collaboration with the librarian. One of the more difficult aspects of librarianship is feeling comfortable and confident working on advanced review methodologies and conducting research. A practicum of this nature is important for early exposure to both research practices and the day-to-day functions of an academic librarian. While it is crucial that the student learns the basics of reviews and the librarian’s role, it is even more important that the student sees research as a creative process.

Description: This practicum was developed in collaboration with a first-year library science student. Each week, the student will be exposed to different aspects of review methodologies and librarian roles within them. Simultaneously, the student will be working with the supervising librarian on constructing their first robust systematic search for a methodology review. The practicum experience simulates what it is like for a librarian to engage with a team on a review. In this instance, the supervising librarian and colleague would be the review team and the practicum student would be the team’s librarian. To ensure the success of this endeavor, the librarian has mapped out weekly lecture topics and tasks to engage the student as well as created visual materials for learning. Additionally, the librarian must fill multiple roles for the student. They would be the primary investigator of the research project, the point of contact for the library student about the project, and a librarian mentor to the student.

Conclusion: While the practicum experience is still ongoing, there are already promising strides being

made. Naturally, the overarching goal is to expose the student to research in medical libraries. However, once this goal has been completed, the librarian hopes to use the framework of the practicum and the materials prepared for the student to generalize to other library science students - whether this be for a credit-bearing course or for another practicum experience. The main outcome of this experience is to demonstrate that it is possible to create an engaging and informative systematic review practicum experience.

Use of RRIDs in Publications to Capture Research Core Facility Usage

Track: Information Services | On Demand

Robyn B. Reed, AHIP - Biomedical Informatics and Emerging Technologies Librarian, Harrell Health Sciences Library, Hershey, Pennsylvania

Background: The use of persistent identifiers in research has been on the rise. One application is using Research Resource Identifiers, or RRIDs, for citing biomedical resources in the literature to help with identification of equipment and research reproducibility. This pilot project describes how our team of a medical librarian, a marketing staff member, a core facility manager and research administrators employ our research networking platform to identify and showcase outcomes of core facility equipment.

Description: Tracking publications that describe research utilizing institution-funded research cores and facilities to generate research data can be challenging. Relying on researchers to self-report publication acceptance is limiting and contacting researchers for updates is time consuming. The Penn State College of Medicine Biomedical Research Core Facilities request the use of RRIDs in publications by researchers who use the services. The goal of this approach is to make tracking of publications easier and provide more accurate information for return-on-investment analysis. The medical librarian created search strategies with RRIDs, core facility names and institution to query Scopus and Dimensions databases. Results were shared with the core facility manager and relevant publications were assigned to their appropriate core facility in the research networking system for public display. This lightning talk will highlight the outcomes from a facility as well as describe the role the librarian has in this project.

Conclusion: Our team-based approach to identifying relevant publications has improved our records of core facility usage and demonstrated that not all researchers use the assigned RRIDs in publications. The Director of the Biomedical Research Core Facilities will implement strategies to increase the usage of the identifiers in future publications. Additionally, our efforts have improved internal return on investment analysis while publicly demonstrating applications from core usage.

A Visit from the Local Library: Bringing the Public Library to Graduate Health Sciences Students

Track: Information Services | On Demand

Kristen Sheridan - Education and Information Services Librarian, Boston University's Alumni Medical Library, Boston, Massachusetts

Background: How can we provide e-books, audiobooks and streaming media to our students to promote wellness when our institution does not have access to resources like OverDrive? Numerous studies have

suggested that leisure reading is a valued self-care activity and beneficial to overall student wellness. However, finding the time to read and accessing preferred digital formats are not as readily available from an academic health sciences library. With this in mind, we sought to provide resources that support leisure reading that are available in our community. Because of our student's rigorous academic programs, they have little time for activities like leisure reading. In order to break this barrier, we partnered with our local public library to bring these resources to them.

Description: Our institution is across town from our main campus, and there are limited spaces for students to gather for outreach events and programs. While the library is one of the student-centered spaces, we are on the twelfth floor of the main instructional building and do not have a large group space for programming. In order to reach as many patrons as possible, we reserved a table in the lobby of our building to promote this pilot program and were accompanied by two librarians from the public library. The public librarians brought branded library swag like pens, stickers, bookmarks etc. for patrons to take when they walked by or after they signed up for a library card. We were positioned in an advantageous spot by the front door in the lobby and were able to capture patrons as they were coming into the building. Because of this location, we had many patrons stop to learn more about resources available to them if they signed up for an eCard, and we also had patrons stop talk to us even if they already had a library card and were actively using e-resources like Libby, Hoopla and Kanopy.

Conclusion: We were pleasantly surprised with the positive outcome that came from this pilot program. We were busy for the full two hours of the program and concluded with signing up 42 patrons with a library eCard. This was a valuable partnership with our public library, and a great way to strengthen ties within our community. We were the first university that our public library did an eCard signup program with, and this gives us hope that we will collaborate on a variety of projects in the future. We plan to run this program again in 2023, but will try a different weekday and time frame in the hopes of pursuing new patrons with different work and class schedules.

Why Do Journal Websites Look Like That? Teaching with Print Science Journals

Track: Education | On Demand

Kayleigh Bohemier - Science Research Support Librarian, Marx Science and Social Science Library, Yale University Library, New Haven, Connecticut

Background: This lightning talk highlights an active learning exercise that has been added to some literature review sessions for students in science disciplines (astronomy, geosciences, and physics) in order to improve student awareness of the continuity between print journals and the online resources that they use every day. The primary goal is to give students an experiential sense of what they are interacting with when they are selecting fields to search or reviewing an electronic table of contents.

Description: Many students in the sciences have never touched a print journal, which has an impact on how they navigate our online systems, ranging from requesting books to using advanced database features. Adding an exploration of print journals from the 19th and 20th centuries was originally done for a summer library enrichment session as a way to break up the time through an active learning session. It has since become a valuable tool, serving both as an icebreaker in longer information literacy sessions and as a way to prime students in these sessions to think more deeply about the history of science communication and how it has led to what they are seeing in their online searches.

Conclusion: The initial experience using this active learning exercise as a way of giving students hands-on experience with print resources has been positive, and there are opportunities to integrate it into more formal

assessment through pitching it as a library session module to faculty and by implementing a future pre- and post-session assessment to measure changes in levels of student confidence and knowledge.

LIGHTNING TALK PRESENTATIONS: SPECIAL LIBRARY ABSTRACTS

We have sorted lightning talk presentation abstracts in this section by title in alphabetical order and note presentations available to conference attendees on-demand.

Evolving & Re-Imaging Physical Library Space in Health Care Settings

Track: Special Library | On Demand

Alice Anderson - Director, Library Services, Monash Health, Clayton, Victoria, Australia

Objectives: Over the last 25 years, health library collections and working practices have shifted in response to an increasingly digital world. There is a need to examine the continuing role of physical library space, and the transition from outmoded models that emphasizes collection storage. This presentation will advocate for developing spaces that support multi-function areas, social infrastructure, and place-centered activities. It will also report on key findings from a research investigation into the enduring role of physical library space in health care environments, which was supported by ALIA HLA (Australian Health Libraries Association, Health Libraries Australia) in 2020/21.

Methods: An international literature review and survey of health libraries was conducted from Melbourne, Australia (one of the world's most locked down cities) to explore the enduring requirement for health library spaces. This lightning talk reports on the findings from two publications that arose from the study. The first shared insights from the Australian health care sector, based on seven interviews with library leaders. The second included a literature review and through a survey it revealed the resilience of library space in the pandemic despite lockdown challenges. Together these two articles support a call for health services to take into account the importance of library space for health professionals knowledge, development, education and wellbeing.

Results: In short: • Decades of change have laid the groundwork for re-imagined spaces. • Digitisation and reduced print collections provide a catalyst for change. • Library space must serve its patrons, not the past. • There is a significant need for flexibility and adaptability. • Library space is associated with facilitating use information technologies and digital literacy. • A variety of zones should accommodate a range of uses in library space. • Reducing footprints and funding are key challenges for library space.

Conclusions: The shift towards digital technology combined with the impact of the COVID-19 pandemic, presents opportunities for lasting change in physical library spaces. If we take the words of Arundhati Roy for inspiration "...we can walk through lightly, with little luggage, ready to imagine another world. And ready to fight for it." - Arundhati Roy, ‘The pandemic is a portal’ How will we reimagine library space? What luggage will we take and what will we fight for? Thinking about these questions will guide the evolution of library space and ensure that it endures long into the future.

Libraries in the Field: Information Use at a Remote Field Station and the Libraries’ Role in Field Science Education

Track: Special Library | On Demand

Thomas E. Gerrish - Engineering Librarian, Field Librarian, Purdue University Libraries; University of Michigan Libraries, West Lafayette, Indiana

Scott Martin - Biological Sciences Librarian, University of Michigan, Ann Arbor, Michigan

Objectives: Field station libraries are an understudied aspect of the library profession. This presentation will first discuss what is a field library. Then, we will summarize how students, faculty, and researchers at a remote field station use information pursuant to their work and studies in field ecology, geology, and environmental science. Lastly, we will discuss the role of the library in field science education and research.

Methods: During the in-person Summer 2022 session, the information needs were surveyed at a remote field station. We collected syllabi, assignments, and conducted semi-structured qualitative interviews with field station faculty, researchers, and staff. The interview included: (1) Which resources were used in class (2) How subject specific information literacy was taught (3) Observed problems using information in teaching and research (4) Use of library resources in constructing teaching materials (5) Use of GIS, spatial data, and maps (6) Use of information resources in planning course based research and personal research (7) Use of specific software such as R, SPSS, or GIS (8) Use of data in courses and research These interviews were recorded and later keyed. In the field library, a physical gate count was collected in addition to the visits to the field station's LibGuide. All reference questions were recorded with an assigned a READ score. Additional usage data from the previous virtual session was collected to compare virtual and in-person library services at the field station.

Results: This work is currently in progress. The initial results suggest that the instruction at the station relies heavily on the databases like Web of Science, Zoological Record, and PubMed. Instructors and researchers made use of maps and GIS, but were uncomfortable with using the software and used the library to locate maps and GIS data. Surprisingly, the university institutional repository was repeatedly mentioned as a valuable source of information on site locations, historic species locations, and previous research methods. Older printed materials were also used. In-person library interaction at the field station was strongly preferred to virtual by all parties.

Conclusions: This is currently work in progress. Field stations have specific information needs including literature, GIS data, maps, historic ecology information including undigitized works from the 19th-20th centuries. Field stations are regionally focused and the collection should include the local area such as student papers, pilot studies, unpublished works, and dark data sets within FERPA/privacy. Interestingly, field science instructors, researchers, and students rely heavily on data and GIS analysis, but the presence of these skills in the discipline is lagging, which could constitute an opportunity for library services. The information needs and geographic specificity of field station with an education mission show a field station library constitutes a under described type of academic library with specific collections and librarian skill sets.

Plain Language. In Plan Language.

Track: Special Library | On Demand

Cara Marcus - Knowledge and Resource Manager, Resource Center, Woburn, Massachusetts

Objectives: According to the federal Plain Language website (plainlanguage.gov) plain language is “communication your audience can understand the first time they read or hear it.” This lightning talk will provide Special Librarians who write customer-facing publications, including resource abstracts, newsletters, website content, etc., with the tools and techniques they need to assure that their writing is clear and understandable to their users, including those whose first language is not English or those who have learning or other disabilities.

Methods: National Rural Transit Assistance Program (National RTAP) is a program of the Federal Transit Administration whose mission is to address the training and technical assistance needs of rural, and tribal transit operators across the nation, and to support the State RTAPs through a comprehensive set of free technical assistance programs and resources. Our peer-reviewed Plain Language for Rural Transit: Writing for Readability and Clarity technical brief, written by Knowledge and Resource Manager Cara Marcus, is based on tenants of the Tufts University Plain Language for Health (PL4H) course she completed, with additional content provided for transportation librarians. Research included a literature review, case examples of transit materials written in plain language, and tactical best practices recommendations.

Results: The technical brief has been viewed 241 times and downloaded 171 times since its publication. The print brief has also been ordered by numerous transit agencies and displayed at many regional and national conferences, reaching additional users. The brief's takeaways have also been used for various research publications, such as Division of Planning Research on Call (ROC) 2020-14 Task 5: Effective EJ and LEP Engagement Strategies and Methods for Statewide Plans. In 2021, the National Transportation Library was putting together a bibliography of important works on transportation equity and justice, and this brief was identified.

Conclusions: Plain language skills and techniques learned from this technical brief and proposed lightning talk can ensure that materials written by special librarians can understood by people with low literacy, or people whose first language is not English. Transportation organizations that receive federal funding are expected to comply with Title VI regulations for low-literacy populations, so the lessons learned can also help maintain compliance with the law.

SLA EDUCATION SESSION ABSTRACTS

We have sorted education session presentation abstracts in this section by title in alphabetical order and note presentations available to conference attendees on-demand.

Algorithm Bias: Detection and Perspectives of Undergraduate Students

Track: In Person

Wei Zakharov - Associate Professor & Engineering Information Specialist, Purdue Libraries & School of Information Studies

Education Session Description: There have already been numerous reported computer algorithm biases that systematically discriminate against certain content, individuals, or groups and that have had serious impacts on society. This session examines an information literacy component focused on algorithm bias in the presenter’s undergraduate-level course on data management. The course meets the university’s requirements for the certificate “Applications in Data Science.” The detected cases, and perspectives of undergraduate students in regard to privacy, fairness, and ethics will be shared and discussed.

Auto-tagging Documents Using the ICS-10, CPT, and HCPCS Code Sets in All Document Types

Track: On Demand

Marjorie M K Hlava - Founder/Chairman/President, Access Innovations, Albuquerque, New Mexico

Education Session Description: Autotagging for EHR and patient encounter records including clinical protocols, techniques, etc., using the ICS-10, CPT and HCPCS code sets usually used in billing applications within the Electronic Health Record for submission to Medicare and other agencies for reimbursement is expanding. These codes are being increasingly used to tag clinical guidelines, journal articles, areas of clinical expertise, audio visuals of everything from conference presentations to surgical techniques, etc. Although within the library world we think primarily of coding or tagging / indexing records with the Medical Subject Headings (MeSH) and other National Library of Medicine resources the increasing connection between what is allowed for billing and the recommended guidelines for care have spiked a demand that more information is coded against these traditional invoicing codes for broader access to pertinent information held in additional resources.

Beyond the Impact Factor: How Do You Show Success Beyond a Single Number?

Track: In Person

Lance Utley

Education Session Description: What do you mean my journal doesn’t have an impact factor? Why am I not seeing my item in Web of Science, how do I show its impact? Many of us hear such examples from users who need to show how their work has impact, but the traditional citation counts and impact factors are not always sufficient - or paint only a partial picture. Find out how to paint more of that picture for your users and help them understand alternative metrics options. Speakers: Rebecca A. Welzenbach, University of Michigan, Danny Dotson, The Ohio State University.

Biomedical and Life Sciences Community: Vendor Roundtable

Track: In Person

Darra R. Ballance - Assistant Professor, Robert B. Greenblatt MD Library, Augusta, Georgia

Donna S. Gibson - Director, Library Services, Memorial Sloan Kettering Library, New York, New York

Andrew Dunn - Customer Success Manager, CAS

Kathi M. Grainger - Manager, Wolters Kluwer, Cedar Rapids, Iowa

Shamika Shah - Customer Success Manager, Springer Nature

Education Session Description: Join the Bio Community and three to four of our vendor partners for this informative session with Q&A. Each 10-minute vendor talk will highlight new and innovative resources and services. Our theme this year will be: Teaching and supporting next-generation researchers; understanding the best ways to uncover scholarly journal literature. The focus would be on bibliographic databases/products that have new enhancements and features for narrowing search results and finding the most relevant work on a topic of interest.

Book Discussions in Your Medical Library—How to Enhance Outreach through Books

Track: In Person

Claudia Schuchardt-Peet, MS, MLIS - Medical Librarian, Education Resource Center, Chambersburg Hospital, Pennsylvania

Georgeanna Ledgerwood - Medical Librarian/CME Medical Education Specialist, WellSpan Health Good Samaritan Hospital Library, Lebanon, Pennsylvania

Meredith I. Solomon, AHIP - Senior Outreach Officer, Countway Library of Medicine/Outreach Office, Quincy, Massachusetts

Awareness of library resources is paramount to library usage. In this session you will learn how libraries can utilize books and book discussions to market library services across all sectors of healthcare. Books not only connect clinicians, but also give voice to the greater health community. Learn how book discussions enhance patient and family centered care while supporting clinician engagement and retention. Book discussions help librarians build relationships with users, crucial to leveraging support for library services. You will walk out of this session with a book discussion title list, know how to apply for book discussion CME credits, and more. This is an interactive session where we will divide you into small groups to discuss

marketing ideas, what attendees have done in the past, and any other ideas which come to mind. Join us for some bibliographic fun!

The Breakfast Club: Leveraging Cross-Disciplinary Expertise to Create Strong Interprofessional Teams

Track: In Person

DeAnn Brame - Assistant Director, Network of the National Library of Medicine, Web Services Office

Education Session Description: Studies have shown that there are immense benefits to having diverse teams in the workplace--from increased productivity to creative breakthroughs--but what is it really like to work on one? While our time spent in the library is not for detention like in the 80’s classic film, we do spend our days working together to find common ground. Join four professionals, with diverse backgrounds ranging from grassroots organizing to public health to arts non-profits to library administration, as they discuss working on an interdisciplinary start-up team, housed within an academic health sciences library. In this session, we will discuss our backgrounds and how our unique expertise has contributed to forming a cohesive and dynamic work relationship. Learn how we’ve built a culture filled with empathy, trust, healthy conflict, and lots of humor. Explore how to maximize diverse skill sets, work experiences, and both formal and informal education, to create cohesive workplace teams. Attendees will reflect on ways to integrate their own unique career experiences into their current or future work environments.

Census Data for Research: Learn About the Census and the American Community Survey

Track: In Person

Michael Arthur, Geographer, Geographic Customer Service Branch, Geography Division

Ellisa Johnson

Sam Patton

Education Session Description: Students, researchers, and a variety of organizations use data from the American Community Survey (ACS) to answer questions and make decisions. Join this session for an overview of Census and the ACS, ACS data, how organizations have used ACS data to understand the health and well-being of their communities, and how you can use ACS data to research and better understand your community. This session will also feature a live demonstration on how to access the latest ACS and 2020 Census tables on data.census.gov. We show how to create maps on the site and also highlight the latest updates over the past year. Attendees will also learn the basics of geography hierarchies, relationships, and easy-to-use geography reference tools that enable you to visualize boundary changes over time and find geographies for a particular address.

Cybercrime and Information Content—the Role of the Information Professional

Track: In Person

Helen B. Josephine - Principal, HBJ Associates, CONCORD, California

Emily J. Glenn, AHIP - Interim Dean, McGoogan Health Sciences Library, Omaha, Nebraska

J.Wolfgang Goerlich

Emily Singley - Vice President, North American Library Relations, Elsevier, Massachusetts

Education Session Description: Information content in libraries is under attack by unauthorized users and hackers. Recent research suggests that cyberattacks in higher education and in the public and private sector are on the rise while libraries' competency in addressing cybersecurity is mixed. Mitigating these risks can be especially challenging for libraries with minimal support from internal IT departments. This program will present practical steps to mitigate risk and proactively protect against cybersecurity threats. The panel will include Stacey Ruel, a representative of SNSI (Scholarly Networks Security Initiative), J.Wolfgang Goerlich, a Chief Information Security Officer (CISO), Emily Singley VP of North America Library Relations, Elsevier, and Emily Glenn, Interim Dean, McGoogan Health Sciences Library University of Nebraska Medical Center. This panel of experts will offer practical strategies and best practices for libraries and librarians to better protect themselves and their digital collections from the increasing threat of cybercrime. The program will include interactive exercises so participants can test their own risk exposure on the open web. Tips for how to proactively protect against threats will also be shared with attendees. A robust question and answer session will also be included.

Data Literacy for Info Pros

Track: In Person

Marydee Ojala - Editor, Online Searcher, Indianapolis, Indiana

Education Session Description: Putting words into search boxes is the default behavior of information professionals but statistics, datasets, and numerical information can be even more vital to a research project than the words describing the numbers. Data literacy is rapidly becoming a core competency for information professionals. Ojala helps you figure out reputable sources for numeric information, their credibility and validity, and potential for manipulation. Evaluating not only sources but also data presented in sources helps winnow out false or simply misunderstood information.

Develop a Product Dashboard that tracks relevant literature (patent and non-patent) to the Product

Track: On Demand

Christine Geluk

Education Session Description: The session will focus on the mechanics of building a "product dashboard with Microsoft Power BI. While the example used will be a drug product, the principles and techniques described in the session can be applicable to building any product dashboard for any other industry. Free and open resources to build the dashboard like from National Center of Biotechnology Information (NCBI), World Intellectual Property Organization (WIPO), European Patent Office and more. Key Take-aways: Learn

the techniques on the resource selection for building a product dashboard, understand how to leverage Power BI with building such a dashboard, personalize the dashboard for your own needs.

Digital Tools To Empower Your Collections Strategy (And Stretch Your Budget)

Track: In Person

Jessica Clemons, MLS - Director of Development, North America, Technology from SAGE

Tony Landolt - Head of Academic Business Development, Reprints Desk, Walnut Creek, California

Tara Ghaemi

Education Session Description: Connecting patrons with relevant content at the ‘point of need’ is a fundamental role of librarians everywhere. This process is growing more complex as libraries and librarians (and budgets) are stretched to the max. What if there was an innovative and collaborative solution? We will explore the benefits of demand-driven acquisition and how to provide your patrons with instant access to journal articles, eBooks, open access versions, and more. We will also share tips for improving the patron user experience using success stories from several institutions. Attendees will learn how simple, yet powerful, digital tools can work together to enhance your services, save you time and frustration, and work hand-in-hand with users, wherever they are searching.

Equity & Leadership in Specialized Libraries

Track: On Demand

Nabi Hasan - Librarian & Head, Central Library, New Delhi, Delhi, India

Arnetta Giradeau - Repository Manager, North Carolina Central University Law Library, MEBANE, North Carolina

Nastasha Johnson - Associate Professor of Library Science, Purdue University, Indiana

Education Session Description: Specialized library leaders face a choice in the modern workplace: develop a culture of equitable practices for workers or face irrelevancy. As a leader, you are accountable for ensuring that all staff feel free from inequities. But how do managers and directors in the specialized library field effectively communicate equitable relationships with staff? In this session, participants will learn about policies and practices that are inclusive and give a voice to employees who need it the most. They will also gain key insights into communication methods and opportunities that may enhance the experience for all workers.

The Future of Faculty OER Outreach

Track: In Person

Christine Andresen - Associate Professor/Research and Education Informationist, Medical University of South Carolina, Charleston, South Carolina

Jeanne Hoover - Head of Scholarly Communication, Academic Library Services, East Carolina University, Greenville, North Carolina

Education Session Description: Over the past two decades, Open Educational Resources (OER) have been utilized in all types of education across all disciplines. In addition to saving learners money, OER offers educators the flexibility to customize accessible instructional materials that are better aligned with an individual’s teaching style to best meet learning outcomes. Libraries are well-positioned to support affordable learning initiatives by leveraging institutional subscription resources as alternatives to traditional instructional materials, and as librarians, we have an opportunity to be proactive in encouraging the use and adoption of OER which can be challenging. In this interactive session, utilizing polling technology and small/large group brainstorming in multiple modalities, we will share ways to engage all stakeholders with OER and alternative affordable learning resources. Successful stakeholder engagement models will be discussed, and attendees will leave the session with concrete ideas for OER outreach at their own institutions and libraries.

How to Make Research Data Available and Ensure Compliance with the OSTP Memo

Track: In Person

Andrew Mckenna-Foster - Product Specialist, Figshare

Education Session Description: In August 2022, the Whitehouse Office of Science and Technology Policy announced new requirements from federal agencies “Ensuring Free, Immediate, and Equitable Access to Federally Funded Research.” Namely, this requires executive agencies and departments to: 1. Update their public access policies as soon as possible, and no later than December 31st, 2025, to make publications and their supporting data resulting from federally funded research publicly accessible without an embargo on their free and public release; 2. Establish transparent procedures that ensure scientific and research integrity is maintained in public access policies; and, 3. Coordinate with OSTP to ensure equitable delivery of federally funded research results and data.

In 2019, we saw the number of open access academic papers published globally, passing 50% of all papers published. There are also many new business models and routes to support open access as the push has steadily gained momentum since it was first conceived in the 1980s. Authors and funders have a plethora of green and gold open access publication routes, although managing the cost implications of the latter is an ongoing discussion. Open academic data is largely a new concept to researchers and funders alike. Whilst several fields have been benefiting from making data available for some time, for example, genomics and astrophysics—many communities are struggling with the concept of where and how to make their research data accessible. Several are struggling even with the concept of what ‘data’ means to their field.

The State of Open Data report from Digital Science surveys researchers on their attitudes and concerns with regards to open academic data. Since 2016, we have monitored levels of data sharing and usage. Over the years, we have had 25,000 responses from researchers worldwide providing unparalleled insight into their motivations, challenges, perceptions, and behaviors toward open data. The State of Open Data is a critical piece of information that enables us to identify the barriers to open data from a researcher perspective, laying the foundation for future action. This year’s report also includes guest articles from open data experts at the National Institutes of Health (NIH), the White House Office of Science and Technology Policy (OSTP),

the Computer Network Information Center, Chinese Academy of Sciences (CNIC, CAS), publishers and universities.

A separate Digital Science report led by Ripeta is titled The State of Trust & Integrity in Research has insights into how researchers are actually acting in practice when it comes to open research data. It demonstrates that improved data sharing policies will boost public trust in research. In addition to its detailed analysis of the five major funders, Ripeta found that with regard to 62 key funders worldwide: ● 71% of funders required data management plans ● 68% of funders covered expenses of data management and sharing ● 66% specifically mentioned data sharing repositories as a mechanism of making data publicly accessible ● 26% of policies mentioned how long data should be retained. Locating funding agency policies proved to be particularly difficult, with many not surfacing through web searches. There was also significant policy variability between funding agencies, with many policies having differing requirements for implementation. Utilizing Ripeta’s services, funders and government agencies have the ability to analyze and monitor compliance within established open science or data management and sharing policies, while also identifying areas of good institutional research practice. Exclusive analysis by Ripeta in one section of the report compares the policies and practices of five major world funders: the Bill and Melinda Gates Foundation, the European Commission (EC), the National Institutes of Health (NIH) in the United States, the National Natural Science Foundation of China (NSFC), and the German Federal Ministry of Education and Research (BMBF).

The analysis shows that although each of the funders have their own policies in place to support data sharing, there are discrepancies between those policies and how they translate into practice. For example, while the number of papers with data availability statements is increasing, “Data available upon request” statements are becoming more prevalent and may not perfectly align with funder policies. Best practice is to use a repository and reference that location in the data availability statement but researchers may avoid this option for a variety of reasons as noted in the State of Open Data report. This session will discuss what we know about how to encourage researchers to make their data available and how we can ensure compliance with the OSTP memo going forward.

Impact of Changing Diagnostic Nomenclature for Mental Disorders on Taxonomy Maintenance and Information Retrieval

Track: On Demand

Marisa R. Hughes - Taxonomist, APA Publishing, Herndon, Virginia

Education Session Description: Following a review of historical terms within the APA Thesaurus of Psychological Index Terms, Marisa Hughes will discuss the history of early classification systems for mental disorders and symptoms, and will trace the evolution and current status of diagnostic nomenclature within present traditional systems, including the Research Diagnostic Criteria (RDC), DSM-5-TR (published just this last February) and ICD-11 (effective 1/22), as well as empirically-based research frameworks, such as the Research Domain Criteria (RDoC) and Hierarchical Taxonomy of Psychopathology (HiTOP). She will then discuss the role of these nomenclature changes in the maintenance of existing controlled vocabularies that include or incorporate diagnostic terminology, as well as the impact on indexing accuracy, search, and information discovery.

Information Leadership in 2030: What’s Next for Info Pros

Track: In Person

Daniel Bostrom - Member Engagement Manager, RAILS, Burr Ridge, Illinois

Leslie J. Reynolds - Senior Associate Dean, University of Colorado Boulder

Stephanie Rollins - Library Director, Air University Library, Maxwell Air Force Base, Alabama

Education Session Description: We know that the pandemic threw a lot of management teams for a loop in 2020. But what will 2024 look like? And what is beyond? How will leadership teams need to shift in terms of style and tone? Join our team of panelists to look into the crystal ball in terms of information leadership. What are the trends and how will leaders need to adapt? In this presentation, panelists will address topics like content buying, marketing, managing teams, and investing in sustainability.

Introduction to Knowledge Graphs

Track: On Demand

Heather Hedden - Knowledge Manager, Semantic Web Company, Boston, Massachusetts

Education Session Description: There is a growing interest in knowledge graphs to organize information and make it findable in organizations with large amounts of data and content. Unlike technologies involving big data or federated search, a knowledge graph has a structure that is typically based on a taxonomy and ontology, and thus the creation of a knowledge graph, should involve information professionals. Knowledge graphs also provide more benefits than information findability, including discovery, analysis, and recommendation. Knowledge graphs bring together content and data. This session explains what a knowledge graph is and how it is built, with a particular focus on the ontology component, and it also presents examples and standards.

"Kill A Stupid Rule" for Process Improvement, Transparency Across Functions and Engagement on Your Team

Track: In Person

Karen Whitney - Library Resources Manager, Perkins Coie, LLP - Library, Seattle, Washington

Education Session Description: Learn how a law firm library department used the “Kill A Stupid Rule” process improvement activity to streamline processes, facilitate innovation, break down silos and promote engagement on our team. This session will cover the basics of the process review activity, provide best practices of how to implement it on your team, and identify the benefits of this type of activity beyond process improvements.

Leveraging User Data to Increase & Improve Engagement

Track: In Person

Jill Strand - Director, Research & Knowledge Services, Fish & Richardson, P.C., Minneapolis, Minnesota

Education Session Description: All libraries face the challenges of time, management pushback, and budget constraints. This session is a case study of how one firm leveraged the technology available to them to plan, start, and maintain an outreach effort to gain deeper insights into the needs of their internal clients. This work has strengthened the Research & Knowledge Services department's position as a strategic partner in their organization's practice and business development efforts.

Maintaining and Managing a Changing Taxonomy: Taxonomy Governance Fundamentals

Track: On Demand

Heather Hedden - Knowledge Manager, Semantic Web Company, Boston, Massachusetts

Education Session Description: Abstract: Taxonomies provide a link between users and content that enhances findability. Taxonomies are also flexible, and they can and should change as content, systems, or user objectives evolve. However, since taxonomies are controlled vocabularies, changing them also needs to be controlled. Managing change in taxonomies is the main concern of taxonomy governance. This session presents a thorough overview of taxonomy governance including planning, scope, roles and responsibilities, policies and procedures, and types of governance documents. How taxonomy management software and taxonomy standards support governance and where they need to be supplemented by a governance policy is also discussed.

Mindfulness and Mental Health Improvement Practices for an Effective Work-Life Balance (WLB) in Librarianship

Track: On Demand

Jay Bhatt - Engineering Librarian, Scholarly Connections, Drexel University Libraries, Philadelphia, Pennsylvania

Poonam Bharti, PGDLAN, MLS & UGC-NET - Librarian, IILM University, Greater Noida Campus, India, Greater Noida, India

Meenal Oak, MES Institute of Management & Career Courses - Librarian(Professor- Level 14) & Coordinator, MES Institute of Management & Career Courses, Library, Pune, Maharashtra, India

Education Session Description: Stress, burnout, anxiety, we all need help to manage and improve our well-being. Join this session for a discussion of techniques you can use to reduce your stress, increase resiliency in the workplace, and help generate ideas for implementing stimulating programs to inspire library

users. Poonam will present on how being a little mindful instead of “mind full” can lead to improved mental health and lifestyle. Jay will share the in-person and virtual programs created at Drexel University Libraries to promote intellectual wellness and reduce stress for staff and students. Meenal will share the conceptual ideology of Work-Life Balance (WLB), factors affecting WLB using case examples, and giving the best performance at work. We will have time for questions. Please share your wellness program ideas with us.

Moving Beyond Checklists: Centering The Importance of Onboarding

Track: In Person

DeAnn Brame - Assistant Director, Network of the National Library of Medicine, Web Services Office

Abby Dowd - Instructional Designer, Eccles Health Sciences Library, University of Utah / NNLM NTO

Jamia Williams - Consumer Health Program Specialist, University of Utah/The Network of the National Library of Medicine/Training Office, Rochester, New York

Meredith Gozo, MA, MLIS - Rare Books & Manuscripts Librarian, University of Rochester Medical Center

Charlotte Beyer, MSIS - Associate Vice President, Boxer Library and Information Collaboration, Boxer Library, North Chicago, Illinois

Education Session Description: The Bureau of Labor Statistics predicts that over the next decade, there will be 15,200 library job openings annually (2022) due to “workers who transfer to different occupations or exit the labor force.” So how will we ensure that these new employees are adequately onboarded? Onboarding is a vital part of a new employee’s experience. In this session, we hope to have a candid discussion about onboarding in libraries, with a particular focus on health sciences and special libraries. Our panelists will share their most recent experiences and how they are working to improve onboarding at their organizations. We want to provide a space to engage and share best practices around onboarding within libraries. New employees can be left without proper direction on navigating their job or organizational culture without adequate preparation. Onboarding goes beyond paperwork and checklists.

Participants will have the opportunity to think critically about onboarding practices at their institutions and what changes could be made to improve the experience. We will engage with attendees in the conversation via polls and small group discussions. This will be an opportunity for attendees to participate actively and provide insight into this crucial conversation. They will leave the session with some takeaway strategies for creating and revising their institution’s onboarding practices. Our hope is that this will lead to actionable steps for all levels to make onboarding more effective for new employees.

New Day, New Project: Juggling Multiple Projects in the Information Field

Track: In Person

Daniel Bostrom - Member Engagement Manager, RAILS, Burr Ridge, Illinois

Janel Kinlaw - Manager, Refining Workflow LLC

Education Session Description: Just when you thought you were getting into the groove of a project, a new one gets dumped in your lap! The thing that they don’t tell you in library school is how many projects you’ll be juggling at one time! So what are the keys to managing multiple projects in an information professional role? How are information professionals supposed to prioritize and keep new projects moving? This session will build off of popular LMD project management sessions from past conferences. Participants will gain key insights into tips like managing project timelines, delegating responsibilities, and tracking progress. The session will also cover organizational skills and tools to support your project management approach.

The New Rules for Job Searching in the Information Field

Track: On Demand

Daniel Bostrom - Member Engagement Manager, RAILS, Burr Ridge, Illinois

Lisa Dubler

Education Session Description: Remote work? New terminology? Group interviews? You need a lot more than a resume to get a job these days: you need a plan! There are a lot of jobs out there, but they all they look different and the hiring process feels like an endless set of speed bumps. Job seekers in the information field have so much to navigate! In this session, participants will get the new rules for job searching. Explore tips on building your network, creating a professional statement, and rehearsing your interview answers. Learn what it takes to get your dream job in the modern information field.

Panel Discussion: Advanced Taxonomy Issues: The Response of Controlled Vocabularies to the Evolving Domains of Health and Healthcare

Track: On Demand

Marjorie M K Hlava - Founder/Chairman/President, Access Innovations, Albuquerque, New Mexico

Marisa R. Hughes - Taxonomist, APA Publishing, Herndon, Virginia

Education Session Description: This session will be a panel discussion comprised of 3-4 information professionals who work with taxonomy in some role, discussing advanced topics within their taxonomy work over the past year relating to evolving information and coding in health and healthcare domains. Examples of specific topics include: the impact of ICD-11 coding and terminology, and improved tagging and onsite discovery using secondary taxonomies for health research and CME. Each panelist will discuss their advanced use case, any future applications, and commentary and feedback by the panel will follow, with time for Q&A.

Power of Collaboration in Special Libraries of Asia

Track: On Demand

Parveen Babbar - Deputy Librarian, Dr B R Ambedkar Central Library, Jawaharlal Nehru University, Delhi, Delhi, India

Praveen K. Jain - Librarian, Library, Delhi, Delhi, India

Joseph M. Yap - PhD Student, Institute of Library and Information Science, Eotvos Lorand University, Budapest, Hungary

Dilara Begum - Associate Professor and Chairperson, Department of Information Studies & Librarian (Acting) East West University Dhaka, Bangladesh, Dhaka, Dhaka, Bangladesh

Labibah Zain, n/a - Head of Central Library, State Islamic University (UIN) Sunan Kalijaga, Jl. Laksda Adisucipto Yogyakarta Indonesia 55221, Yogyakarta, Yogyakarta, Indonesia

Education Session Description: How collaboration and collaborative work is impacting the development in library and information centers of Asian Countries. How special libraries are exploring the entrepreneurship and bringing new vision, energy to the librarianship. The session relates to special libraries of Asia, demonstrating how Asian libraries are building a Strong and Vibrant Community with connecting people and ideas to have the knowledge management practices in libraries. The diversification of Asia itself exhibits the community identities and enriches strong community’s unity. The libraries of Asia are practicing enrichment by personal learning and recreation, have the sense of ownership of unique collections and managing Library Services for equity. The inter collaboration within various regions of Asia encourage and promote cooperation in various domains including science, technology and innovation through joint research activity, and development on cross-sectoral areas not limited to health, communicable and emerging infectious diseases, environmental management, climate change adaptation and mitigation measures, agricultural technologies, alternative energy, biodiversity, food processing, advanced materials for development of value-added products, and space technology and applications. Various projects are already running in Asia and libraries are supporting these projects cross nations. Some studies to exhibit how libraries can be vibrant to make these collaborations successful. The session will be of interest to audience unaware about the collaborations in Asia with relation to scientific collaborative projects and libraries supporting these projects.

Researching Information for Smart Cities and Global Sustainable Growth in Communities

Track: On Demand

Jay Bhatt - Engineering Librarian, Scholarly Connections, Drexel University Libraries, Philadelphia, Pennsylvania

Poonam Bharti, PGDLAN, MLS & UGC-NET - Librarian, IILM University, Greater Noida Campus, India, Greater Noida, India

Education Session Description: As new academic research initiatives become increasingly interdisciplinary, it is imperative that librarians worldwide develop and implement new approaches to the dissemination of various research tools and techniques. Many institutions and organizations have evolved, with tremendous potential for future growth, in response to the continued increase in demand for emerging technologies for sustained community growth. Smart Cities initiatives are being envisioned and implemented globally requiring the need for acquiring information on topics such as Information and Communication Technologies, Human Computer Interaction, IT and Information Security, Cryptography, Cybersecurity, Outsourcing, and Entrepreneurship, etc. Students, researchers and entrepreneurs will need to become aware of a wide array of different online resources available through their libraries, corporations, and

organizations. Various strategies of gathering, connecting, evaluating, and using information efficiently will be addressed during this talk. Using these approaches can be very useful for students as they continue to envision innovative Smart Cities related projects. It is hoped that this will also contribute to life learning that can be transferred in future professional and academic activities to continue to contribute towards economic and sustained growth of global communities.

Sci-Tech 101: Introduction to Publication Metrics

Track: In Person

Mary Frances Lembo - Research Librarian - Senior, Pacific Northwest National Laboratory, East Grand Rapids, Michigan

James E. Manasco - Librarian, Manasco Acres, Kentucky

Education Session Description: In this session, the two speakers will provide an introduction to five standard publication metrics (Scholarly Output, Citation Count, h-index, Collaboration and Journal Impact), how they can be helpful in looking at trends and for benchmarking, and will also discuss the limitations and ethical considerations for these metrics. There are also times when metrics “go bad” – just as there are predatory journals, there are predatory or made-up metrics. The speakers will discuss these as well.

SEC Documents: How to Read Some Common Forms and How to Get the Most Information from Them!

Track: In Person

Barbara Coffey - Finance Research Librarian, Princeton University

Education Session Description: Before being the Finance Research Librarian at Princeton University, I worked on Wall Street doing financial analysis. This experience taught me to read 10-Ks, 10-Qs, Proxy Statements, 8-Ks, S-1s and other filings carefully. While full of legalese, these documents hold troves of information. Most of us are familiar with the numbers these documents hold, this session will touch on the numeric content and will focus on the content found in the text. This will discuss the more common SEC filings. Ideally, attendees will learn about using these filing for company research and hopefully the session will demystify these government documents. As for skills, I believe that having familiarity with these documents and being able to use them for company research is a skill. This is a unique offering as this class will be being taught by someone who was a finance professional and practitioner.

Service-Learning Projects Complement Evidence-Based Educational Programs that Build Resilience to Health Misinformation in Academic and Community Settings

Track: In Person

Bethany S. McGowan, AHIP - Associate Professor, Libraries and School of Information Studies, West Lafayette, Indiana

Education Session Description: In this presentation, I advocate for librarian leadership of service-learning projects to strengthen and scale evidence-based educational programs that build resilience to health misinformation. When designed considering community-based participatory research practices, service-learning projects encourage a partnership approach equitably involving community members, students, organizational representatives, researchers, and other stakeholders in the instruction and research process. All partners contribute expertise in decision-making and ownership. I discuss my experience designing and teaching a service-learning course whose learning outcomes raise students' consciousness of and sensitivity to authentic community health information needs by establishing mutually beneficial partnerships between grassroots communities and students and provide local, historically marginalized, and under-empowered communities with a low overhead entry-point into academic partnerships.

After this presentation, attendees will learn to critique service-learning projects as an approach to:
1. Strengthen and scale the use of evidence-based educational programs that build resilience to misinformation in academic and community settings;
2. Respond to and deploy interventions that mitigate and protect against the infodemic and its harmful effects.

The *World Health Organization's Public Health Research Agenda for Managing Infodemics* and *Confronting Health Misinformation: The U.S. Surgeon General's Advisory on Building a Healthy Information Environment* were used to frame the research for this project. Both publications highlight high-priority research needs in the field of infodemiology, including the creation of health literacy interventions that build resilience to and protect against misinformation.

This presentation is possible because of funding from the Institute for Museum and Library Services, for the project ‘Understanding the Health Information Seeking Behaviors of Black Americans: A Cultural Perspective’.

Solo Corporate Librarian Success Stories

Track: In Person

Jamie Hullinger - Senior Coporate Research Librarian, Zimmer Biomet

Lisa Weinberger, MSc - Advanced Corporate Librarian, Med-El Medical Electronics/Clinical Research

Gabriele Hysong - Information Operative/Librarian, Library & Knowledge Services Center/ Rolls-Royce Corp, Indianapolis, Indiana

Jamie Hullinger - Senior Coporate Research Librarian, Zimmer Biomet

Education Session Description: This will be a 3 personal panel sharing success stories from experienced solo librarians in different corporate environments. Attendees will hear real stories from the field. Attendees will be able to take elements of the success stories shared and apply elements to their own libraries. Attendees will be able to see that it is possible to have success as a solo librarian. They will learn examples of what has worked in other library situations. They will hear about success stories in three different industry libraries. Industry and corporate libraries do not always get a lot of exposure within SLA and MLA so it would be an opportunity for their experiences to have a place in the conference; he same is true for Solo Librarians.

Standards Update: What's New with Standards?

Track: In Person

Kim Beets - Global Library Manager, Black & Veatch

Vince Price - Executive Director, Partner Relations, Accuris (IHS), Michigan

William (Bill) Nara - Director, ASCE, Reston, Virginia

Warren Adams - Global Director, ASME

Osvaldo (Oz) Lugo - Account Manager, ASTM

Christine W. McCarty - Product Marketing Director, Copyright Clearance Center (CCC), San Diego, California

Gerri Brady - Sales Specialist, SAE International

Nate Partington – Techstreet

Education Session Description: Get up-to-date information on new standards and specifications as well as platform enhancements from key publishers and aggregators. Specs and standards are critical resources for design and compliance, yet costs and licensing may present challenges to libraries and information centers. Attend this long-standing annual session to optimize your knowledge about these core materials in the engineering, transportation and science disciplines.

Strategizing for Leadership in the Information Field

Track: On Demand

Susan Klopper - Director, Goizueta Business Library, Emory University

Education Session Description: You don't lead by pointing and telling people some place to go. You lead by going to that place and making a case. —Ken Kesey Having a vision matters! Visionary leaders can help transform an organization. Managers who present a strategic vision for their employees can make their organizations a better, more exciting place to work. How are you sharpening your strategic leadership skills? Attendees will learn tips on strategizing for leadership. This session will help you develop a strategic plan for offering exciting products and services that make a difference.

Successful Info Pros in a Data-Driven Enterprise

Track: In Person

Mary Ellen Bates - principal, Bates Information Services Inc., Longmont, Colorado

Education Session Description: In an increasingly complex and interconnected information environment, finding and delivering intelligence to library clients requires a different set of skills. Info pros are using new tools and strategies to extract critical insights from online resources, provide strategic data management services, and bring semantic enrichment and meaningful structure to text and data mining projects. Mary

Ellen Bates interviewed a number of experienced info pros about their approaches in working collaboratively with data professionals within their organization. She will share best practices, biggest challenges and advice for building the value of an information center in a data-driven enterprise. Learn what these experts wish they had known when they started working with data scientists, and what they learned the hard way so you don't have to.

Sustainable Mobility

Track: In Person

Stephanie Sheldon - Librarian, Lockheed Martin

Education Session Description: Join us for a fireside chat with publishers and industry experts as we discuss the emerging field of Sustainable Mobility whose goal is to secure a net zero emissions transportation future. Focus areas include: Electric Vehicles & EV Infrastructure, Battery Lifecycle, and Long-Distance Transportation that incorporates alternative fuels including hydrogen, Sustainable Aviation Fuels (SAF), biofuels, etc. Impacts for library and information professionals serving this emerging field include: new sources of content; new information needs, especially around tech pubs, training, and certifications; and changes with technical publishing and what we need to know about NextGen research products.

Top Ten Tips for Running a Stem Library: Both Evergreen and Evolving

Track: In Person

Sara R. Tompson - Retired Library Manager; Independent Engineering Researcher, Research Solutions, Lawrence, Kansas

Kim Beets - Global Library Manager, Black & Veatch

Education Session Description: Kim and Sara, multidisciplinary engineering librarians who have served in corporate, academic, and public settings, will share their top ten tips for running a successful STEM library, noting what points have held true over time, and what they have seen change. Insights about best practices will be shared including collection management, physical vs virtual spaces, and marketing of services. Some things have gotten more complex, and some have gotten easier. An example of the latter: we used to have to very stealthily weed our collections, since our engineers and scientists typically hated to see any books in a recycling bin, but now when we weed out unused electronic resources, no one notices! Audience members will be invited to participate in the second half of the session, sharing their own tips, and what they have seen evolve over their careers.

The Transformational Technology of Search

Track: In Person

Marydee Ojala - Editor, Online Searcher, Indianapolis, Indiana

Education Session Description: Intelligent technologies, particularly generative AI, are transforming search. We’ve moved beyond keyword matching to semantic search. Algorithms determine the relevance of our results. Machine learning dictates what our searches find for us and what is deemed uninteresting to us. Natural Language Processing (NLP) adds new dimensions to search possibilities. Cognitive computing helps determine the intent of a search, why a query is being asked. Voice assistants and chatbots free us from the search query box. Searches routinely return results that include images, videos, and numeric data. How can information professionals capitalize on these transformational search technologies and enhance our value to our users and our management?

Unique Teaching Ventures: Making Inroads in New Areas

Track: In Person

Lance Utley

Education Session Description: Why is this librarian showing us cartoons? What are Latex (with or without Overleaf) and JupyterLab? Can my librarian help with data visualization and story maps? Why are these library course guides linked in my class? See how librarians are using different methods to reach users and how these methods move beyond the traditional search, evaluate, and cite one-shot session. Presenters will share successful strategies for teaching excellence including non-information literacy opportunities to pursue in the classroom and breaking the instructor mold by teaching in new ways. Attendees will also learn about opportunities librarians have in the classroom to promote principles of diversity, equity and inclusion. Speakers: Sam Hansen, University of Michigan, Sarah Siddiqui, University of Rochester, Barbara Wetzel, Pacific Northwest National Laboratory.

Using Social Media to Manage Your Career

Track: In Person

Daniel Bostrom - Member Engagement Manager, RAILS, Burr Ridge, Illinois

Leigh Bush - Children's Librarian, Free Library of Philadelphia, Philadelphia, Pennsylvania

Education Session Description: What does social media have to do with your career in the information field? A lot more than you’d think! Your Facebook, Twitter, and LinkedIn profiles tell a story about you as a professional. They can also send a signal to your current employers, future employers, and coworkers. Make sure you’re managing your profiles effectively. In this session, attendees will learn how to use social media to create their personal brand. This session will cover the best ways to build your network, find and follow potential employers, and strike a balance between personal and professional.

Welcome to the Endemic: Librarianship in a Post-Pandemic World

Track: In Person

Lance Utley

Education Session Description: We made quick adaptations to new ways of librarianship during the most intense times of the COVID-19 pandemic. Now that the virus is becoming endemic, are these adaptations also around for the long term? You will hear stories of how librarians responded to the needs of users in new and creative ways as many transitioned to remote work in 2020 and how some of these methods and services have permanently changed how we work in 2023. Speakers: Lauren Gala, Science and Digital Initiatives Librarian at University of Pennsylvania and Stan Smith, Regional Sales Manager at IOP Publishing, Inc.

What Are Publishers' Favorite OA Success Stories?

Track: In Person

Lance Utley

Education Session Description: Are citations up 200% for a journal that went Open Access (OA) only? Do you have 100 read-to-publish agreements with universities? Hear some OA success stories from publisher representatives and see what’s next on the horizon. Panelist presenters will also respond to a series of moderated questions and engage in question and answers with attendees. Speakers: Keith Layson from Annual Reviews, Patricia Hartner from American Physical Society, Robert Harrington from American Mathematical Society, Kevin Steiner from American Institute of Physics

What Info Pros Want R&D Scientists to Know

Track: In Person

Mary Ellen Bates - principal, Bates Information Services Inc., Longmont, Colorado

Education Session Description: Librarians and info pros have always been involved in bringing insight and information to their users. The explosion of new information sources, more open-source content and an increased focus on data analytics means that info pros need new ways to communicate their value to R&D professionals, who often assume that their information needs cannot be addressed by the library. Mary Ellen Bates has interviewed a number of tech info pros and will share their best approaches for proactively engaging R&D scientists and effectively communicating the roles info pros can play in knowledge management and support of product innovation. Expect to come away with fresh ideas and strategies for reimagining existing roles to address R&D professionals’ changing needs.

What's New & Exciting in Health Data Privacy Law

Track: In Person

Jill Strand - Director, Research & Knowledge Services, Fish & Richardson, P.C., Minneapolis MN

Sarah Pappas - Senior Content Specialist (Health Law), Bloomberg Law, Leesburg, Virginia

Education Session Description: Healthcare Trends to Watch for 2023: 1. Digital and Virtual Healthcare (Includes Telehealth/Telepsychiatry Expansion/Remote Patient Monitoring) 2. Healthcare Artificial Intelligence & Machine Learning (Includes Predictive Analytics/Nanomedicine) 3. Retail Healthcare Expansion (ex. Amazon Clinic, Walmart Health) 4. Increased M&A and Joint Venture Activity 5. Abortion Law Post-Dobbs.

When Industrial Standards Become Law: The Use of Incorporation by Reference in Federal and State Regulations

Track: In Person

Bobbi Weaver, JD, MS - Reference Librarian Foreign and International Law, California Western School of Law

Kelly Bunting - Senior Research and Content Management Librarian, Analog Devices, Inc., Boston, Massachusetts

Alan Stout - U.S. EPA Office of Transportation and Air Quality

Leena Lalwani - Associate Director HS-STEM/Engineering Librarian, University of Michigan, Ann Arbor, Michigan

Education Session Description: Incorporation by reference is a way for agencies to streamline the regulation process by adopting other rules or standards rather than rewriting them in regulations. The Code of Federal Regulations is published online and free to the public. Incorporation by reference is also used in state regulations. Michigan uses the term “adoption by reference” in its regulations. For example, Michigan regulations on ground water quality and well construction adopt by reference several industrial standards. (See, Mich. Admin. Code R 325.1610). Title 24 of the California Code of Regulations is comprised of various copyright-protected building standards. But, what happens when agencies do not write the regulations and incorporate copyright-protected industrial standards instead? The standards are available at centralized locations in the Washington, DC area (for federal regulations) or state capitals, but members of the public outside of these areas may have to resort to purchasing these standards which have become part of federal and law. This program will look at the role of the information professional in facilitating access to these standards that have become part of our laws.

The Wonderfully Complicated World of Standards: An Open Discussion for Information Professionals on the Ins and Outs of Standards Management

Track: In Person

Jamie Hullinger - Senior Coporate Research Librarian, Zimmer Biomet

Education Session Description: This session is meant to be a moderated yet lively and open discussion on managing industry standards. While there is a yearly session provided by the Engineering Community it is limited to vendor presentations. The focus on salable features does not allow discussion on the management aspect for new librarians-- there is a lot to working with standards, managing packages, and finding the best fit for your team's needs. This complicated environment is best navigated with the help of

experience. A collective and open discussion can open avenues for management tools and strategies that will truly help YOU in your role. Share your questions, frustrations, and experience with our community! This would be moderated by myself possibly with a few additional panelists. This will allow for structure, but the overall goal is to allow questions from the audience to be answered by either the identified panel or those in attendance. This will be an interactive session. Attendees will have the opportunity to ask pointed questions with the benefit of having a broad expertise pool in attendance to answer. They will expand on their existing knowledge on the topic of standards. Be able to understand the nuances between BSI/ISO/ASTM/SAE/IEEE/DIN/ISTA/IEC.

COLLECTION DEVELOPMENT SYMPOSIUM ABSTRACTS

We have sorted collection development symposium presentation abstracts in this section by title in alphabetical order and note presentations available to conference attendees livestreamed/on-demand.

Cruising in the "Non-Traditional" Collections Lane with Your MAAP: Challenges and Solutions

Track: In Person

Evan Erlichman - Northeast Director of Sales at Ovid Technologies, Inc.

Ramune K. Kubilius, MALS, AHIP - Collection Development / Special Projects Librarian, Northwestern University Feinberg School of Medicine, Evanston, Illinois

Beth Callahan, MLS - Head, UL Research Institutes

Bart Davis, MLIS - Head, Galter Health Sciences Library & Learning Center, Northwestern University

Rebecca Snyder, MLIS - Customer Success Manager, Wolters Kluwer

Requests to acquire unique, non-traditional resources, such as medical Spanish online training programs, human gene mutation databases, data sets, and decision-making tools, are among the most challenging requests a librarian can receive. If you’ve faced such requests or want to be prepared when you do, join three experienced collections and resource-savvy librarians to get a MAAP for addressing these requests in ways that serve your institution and your patrons.

The presenters will discuss their challenges and offer you a range of solutions, best practices, techniques, and tips that address all aspects of non-traditional resource management and enable you to navigate your way through the challenges to sound acquisition decisions using MAAP: -Monitor the landscape of resources and needs of your stakeholders -Acquire resources only after evaluating risks and challenges -Assess usage when traditional standards or statistics are not available -Partner with stakeholders in creative solutions for acquiring and managing resources. With your MAAP in hand, you'll be able to cruise through the non-Traditional" collections lane!

This session was developed by the 2023 Collection Development and Resource Sharing Special Program Committee members Stella Hillegrass, Regional Account Manager- NYC, Long Island, & Westchester County, Ovid, and Ramune Kubilius, AHIP, Collection Development/Special Projects Librarian, Northwestern University.

Embracing Healthy Habits to Create Sustainable Library-Vendor Relationships: A Panel Discussion for Librarians, Publishers, & Vendors

Track: In Person

Jodi Hetzel - Vice President, EBSCO

Jean Gudenas, AHIP - Director of Information Resources and Collection Services, Medical University of South Carolina, Charleston

Irene M. Lubker, AHIP - Medical University of South Carolina, Charleston, South Carolina

Matthew Ragucci, MLIS - Associate Director, Wiley

Emily Singley - Vice President, North American Library Relations, Elsevier, Massachusetts

If you are in a library-vendor or library-publisher relationship, you won’t want to miss this panel of experienced librarians and vendor/publisher representatives as they share their wisdom and practical guidance on embracing healthy habits to create an effective, sustainable, and mutually rewarding relationship that serves the shared goal of providing information to users. Library-vendor relationships, because they are both transactional and personal, can be especially tricky to navigate. Baggage from previous relationships, staff changes, personality differences, cultural differences between academia and industry, and more can get in the way. The panelists will share their insights and tips on how you can address these and other challenges as they arise, repair broken relationships, and start off right to build sustainable partnerships. There will be time for audience questions and contributions.

This session was developed by the 2023 Collection Development and Resource Sharing Special Program Committee members Shannon Gordon, Customer Consultant, ELSEVIER, and Jean Gudenas, AHIP, Director of Information Resources and Collection Services, Medical University of South Carolina.

From Manual to Automatic: Improving the Accessibility of Library Electronic Resources

Track: In Person

JJ Pionke - Applied Health Sciences Librarian, University of Illinois Urbana Champaign, Champaign, Illinois

Cheryl Firestone - Senior Manager, American Academy of Pediatrics

Bill Kasdorf - Principal, Kasdorf & Associates, LLC

Heidi M. Schroeder, AHIP - Accessibility Coordinator, Michigan State University Libraries, East Lansing, Michigan

Emily Singley - Vice President, North American Library Relations, Elsevier, Massachusetts

If you work with your library’s electronic resources, you know that making them accessible to all your patrons is both an ethical and practical concern. You won’t want to miss learning how a select group of publishers and librarians are making progress in improving the accessibility of electronic resources and how you can get involved. A representative from a large publisher and one from a medical society will discuss what is new with their current platforms and the plans they have for the future. Two librarian representatives will present on how they are working with publishers and educating communities to provide equal access to information and developing repositories to share accessible educational materials and remediated texts. This moderated panel discussion will include time for audience questions, comments, and idea sharing. You’ll leave the session with a deeper understanding of the current state of electronic resource accessibility, knowledge of ways libraries can improve the accessibility of their resources, and fresh enthusiasm and inspiration for making your electronic resources more accessible.

This session was developed by the 2023 Collection Development and Resource Sharing Special Program Committee members Amy Bleich, Manager, Global Institutional Licensing, American Academy of Pediatrics (AAP), and Steven Douglas, AHIP, Head, Collection Strategies and Management, University of Maryland.

The Great Debate on Controlled Digital Lending: And the Checkered Flag Comes Down on. . .

Track: In Person | Livestreamed

Claire Woodcock - Independent Journalist & Digital Ownership Fellow, NYU Law's Engelberg Center

Dave Hansen, MSLS, JD - Executive Director and Copyright Attorney, Authors Alliance

Devlin Hartline, JD - Legal Fellow, Hudson Institute’s Forum for Intellectual Property

More and more libraries are digitizing and lending library materials, while publishers and experts are divided on whether copyright law and fair use allow it. In this “can’t miss” debate on the controversial topic of Controlled Digital Lending (CDL), a copyright lawyer and an intellectual property lawyer go head-to-head in a debate on the legality of CDL. Dave Hansen, the Executive Director at Authors Alliance and a copyright attorney takes the “pro” side. Devlin Hartline, a legal fellow at Hudson Institute’s Forum for Intellectual Property, takes the “con” side. Claire Woodcock, a Fellow of NYU’s Engelberg Center on Innovation Law & Policy and a journalist whose byline has appeared in Vice and National Public Radio, moderates.

You’ll vote on which side the checkered flag comes down on. There will be plenty of time for audience questions and comments. You’ll leave the session with an understanding of CDL and the arguments on both sides that will enable you to see where you stand on this issue.

This session was developed by the 2023 Collection Development and Resource Sharing Special Program Committee members Sarah McClung, Head of Collection Development, University of California, San Francisco, and Andrea Spector, Biomedical Regional Sales Manager, EBSCO. This session will be livestreamed for virtual attendees and available post-conference on demand.

To Merge into the Resource Cost Sharing Lane or Not: Evaluating and Developing an Approach to Resource Sharing

Track: In Person

J. Michael Lindsay, MSIS, MBA, AHIP - Head, University of Tennessee Graduate School of Medicine, Knoxville, Tennessee

Jen Ostrosky, MS - Assistant Director, JoVE

Diane Campagnes - Regional Sales Manager, Wolters Kluwer / Ovid

Susan K. Kendall, AHIP - Health Sciences Coordinator, Michigan State University Libraries, East Lansing, Michigan

Evan Simpson - Associate Dean, Northeastern University Library

Joe Swanson, Jr., AHIP - Library Director, M. Delmar Edwards, MD Library, Atlanta, Georgia

Your users want more resources than your administration wants to fund, or you are approached by a user, another department, or an external institution looking for ways to acquire a needed resource. You think a resource cost sharing partnership may be the solution for the parties involved, but you’re not sure what partnerships are or what other ways of sharing resources might work. If you want to learn how you can develop, implement, and evaluate a cost sharing partnership, you won’t want to miss this session! A library director, a library dean, a head of library collections, and a publisher representative will share their resource cost sharing experiences and offer tips and guidance on matters ranging from addressing partnership invitations, evaluating funding sources, analyzing the risks and benefits of a partnership, as well as managing and developing strong relationships that will payoff for all.

This session was developed by the 2023 Collection Development and Resource Sharing Special Program Committee members J. Michael Lindsay, AHIP, Head of Collections & Access Services, Preston Medical Library, University of Tennessee, and Jen Ostrosky, Assistant Director of Sales, Key Accounts, JoVE.

On the Road to Transformative Agreements: National and international Perspectives

Track: In Person | Livestreamed

Arthur Boston - Assistant Professor and Scholarly Communication Librarian, Murray State University

Lisa Janicke Hinchliffe - Professor/Coordinator, University of Illinois, Urbana-Champaign

Lieuwe Kool - Director, Amsterdam UMC Academic Medical Centre

Maria Lopes, PhD - Vice President, Springer Nature

James Picken - Business Development Director, Elsevier

Join an exceptional panel of experts in publishing and academia whose work spans the Americas and Europe as they share their experience and insights on the challenges and benefits of negotiating transformative agreements. You’ll gain an understanding of the basic concepts of transformative agreements, increased clarity about whether a transformative agreement is right for your institution, and insights, considerations, and strategies that can help you negotiate agreements. If you decide a transformative agreement is the right road to take, the information you learn at this session will help to pave your way.

This session was developed by the 2023 Collection Development and Resource Sharing Special Program Committee members Donna Gibson, Director, Library Services, Memorial Sloan Kettering Cancer Center, and Rob McKinney, Global Sales Director, NEJM Group. This session will be livestreamed for virtual attendees and available post-conference on demand.

LEADERSHIP & MANAGEMENT SYMPOSIUM ABSTRACTS

We have sorted leadership & management presentation abstracts in this section by title in alphabetical order and note presentations available to conference attendees on-demand.

Connecting with Stakeholders and Communicating your Library's Value Proposition

Track: In Person | Livestreamed

Jill Strand - Director, Research & Knowledge Services, Fish & Richardson, P.C., Minneapolis MN

Mary Joan (MJ) Tooey, AHIP, FMLA - AVP Academic Affairs and Executive Director, Health Sciences and Human Services Library, U of Maryland/Balitmore, Baltimore, Maryland

Jean P. Shipman, AHIP, FMLA - Elsevier, Cottonwood Heights, Utah

Chris Shaffer, AHIP - University Librarian and Assistant Vice Chancellor, University of California, San Francisco

This session will be livestreamed for virtual attendees and available post-conference on demand.

Forging Ahead: Libraries as Engines of Innovation

Track: In Person

Mark Berendsen, MLIS - Deputy Director, Northwestern University Feinberg School of Medicine, Chicago, Illinois

Karen Gutzman - Head, Research Assessment and Communications, Galter Health Sciences Library & Learning Center, Chicago, Illinois

Librarians and information professionals are well-positioned to step into leadership roles in many novel areas of innovation, such as artificial intelligence, data management and repositories, data visualization, makerspaces and virtual reality. At this highly interactive breakout discussion session, you will engage in meaningful conversations, generate fresh ideas, and forge a better understanding of what it takes to lead novel areas of innovation. This session will help you tackle questions, such as: • How can you leverage information competencies in areas of innovation? • What are strategies to launch cutting-edge services? • What knowledge and skills are needed to lead new initiatives? • Where do you start? Whether you have already taken the deep dive and are leading your organization in one of these areas or if you are currently contemplating strategies for getting started, this session is for you!

Improv and Librarianship

Track: In Person

PJ Jacokes - Co-owner, Go Comedy! Improv Theater

Ann Cullen

Elizabeth Downie

Can methods used by Improv professionals in comedy and theatre be applied to the day-to-day work of librarians? Yes they can! In this program learn how these practices can prove valuable in being able to respond and adapt quickly to unfamiliar situations and provide ways to spark new insights in approaching our work as librarians and leaders. The session will start with an overview of research and case studies on how librarians have applied improv techniques followed by a demonstration from PJ Jacokes, co-owner of Go Comedy! Improv Theater, a seasoned improviser and instructor in Michigan's Metro Detroit area.

It’s Not Them, It’s Us: Understanding and Addressing the Factors that Negatively Impact the Recruitment, Hiring, and Retention of BIPOC Librarians

Track: In Person

Kawanna Bright, PhD - Assistant Professor, East Carolina University, College of Education

Kelsa Bartley, AHIP - Education & Outreach Librarian / Learning, Research, and Clinical Information Services, Louis Calder Memorial Library / University of Miami Miller School of Medicine, Miami, Florida

This session will focus on recruitment, hiring, and retention of Black, Indigenous, and People of Color (BIPOC), specifically what library leaders can do to address issues with recruitment, hiring, and retention of BIPOC employees in their organizations and how to implement inclusivity and belonging practices in the workplace. Within the session, participants will learn more about the issues that negatively impact recruitment and retention of BIPOC employees and actions that can be taken to address those issues.

Re-thinking the Definition of Community and Connections in a Virtually Connected World

Track: In Person | Livestreamed

Mari Anne Snow - CEO, Sophaya + Remote Nation Institute

Elizabeth Downie

Thanks to technology advances and new social norms, most of us have easy access to cost-effective connection options at our fingertips. Yet, true engagement can feel elusive. Loneliness and disconnection are very real. By actively pursuing new strategies that leverage what is possible now, we can work together to re-define community by learning new interpersonal skills that create engaged relationships and extend our community impact.

Work & Lead more Effectively: Understand and Adapt Your Preferred Style

Track: In Person

Silke Janz - Executive Coach and Owner, Three Birches LLC

Ann Cullen

Laura Walesby

In this highly interactive session, you will explore your preferred work/leadership style, and how to adapt your style to more effectively work with others and meet situational needs. You will learn to see your and others’ styles through the lens of the Michigan Model of Leadership, developed at the University of Michigan, based on 50+ years of research. The session is led by Silke Janz, a certified Executive Coach and leadership development expert with 20+ years of experience. Silke is affiliated with the Sanger Leadership Center at the Ross School of Business, University of Michigan, and is the Owner of Three Birches LLC, a leadership coaching business.
