# Supplementary material for: 123rd Annual Meeting Medical Library Association, Inc. Detroit, MI May 16-19, 2023
Source: J Med Libr Assoc. 2024 May 22;112(2):E1–E22. doi: 10.5195/jmla.2024.1872 (PMC11305459; doi:10.5195/jmla.2024.1872)
Supplement: Supplementary file 2 — Appendix: MLA ‘23 Poster Session Abstracts [file jmla-112-2-E1-s02.pdf]

Medical Library Association
MLA | SLA ’23 Hybrid Conference & Exhibits

Poster Abstracts

Abstracts for poster sessions are reviewed by members of the Medical Library Association National Program Committee (NPC) and volunteer peer reviewers.

Information is as provided by abstract submitters.

POSTERS: RESEARCH ABSTRACTS

Although poster numbers are included, content in this section is sorted by title in alphabetical order.

Assessing Collection Diversity: Building Inclusive Care (17)

Track: Health Equity & Global Health

Bianca Brillant, DHSc, MLIS - Librarian, Education, St. Cloud, Minnesota

Anne Snieg - Library Technician - Serials, Diversity Council Chairperson, LRC-Library, Milwaukee, Wisconsin

Jennifer J. Jones - Medical Librarian, Fargo VA Health Care System, Fargo, North Dakota

Thomas Keeler, MLIS - Librarian, Education, Minneapolis, Minnesota

Mimi Ray Guessferd - Clinical Librarian, VA New England Health Care System, Manchester, New Hampshire

Objectives: A hospital library must address the needs of the patients served across a spectrum, providing inclusive collections to improve health care providers’ access to appropriate and accurate information. Research has demonstrated that health disparities exist between gender, race, sexual orientation, religious belief, age, and socioeconomic status. Hospital library collections have not always been equitably inclusive of diverse communities. To address this issue, the authors developed a survey tool to identify gaps in hospital library collections and guide librarians in developing more diversified collections to better serve the health information needs of medical providers and their patients.

Methods: The survey tool developed was based off of a diversity audit created by a school librarian in 2020. The librarians were interested in learning about collection items that spoke to the age, gender, race, religion, and sexual orientation of population groups. When creating questions for the survey, the librarians grouped terms of identity into separate questions and used synonyms where appropriate, such as ‘Racial’ OR ‘Ethnicity’ OR ‘Racial Groups’, based on a controlled vocabulary of medical terms. The librarians settled on a list of 56 questions and used a publicly available online tool to administer the survey. The questions and the survey were tested first on the authors’ local library collections, before being sent off to a wider audience. Invitations to conduct the survey were sent out via both a listserv message and an email request. Responses were anonymous and the data was calculated in a spreadsheet from the thirty-five participants that were used as a sample population.

Results: The collection assessments, completed by 16 participants, were based on the following subjects: age, disability, religion, gender, and sexual orientation. The responses revealed “child or children” was the largest age group indexed and represented in 18% (11,458,292) of the 111,932,701 titles, 3.35% (3,917,645) of the holdings covered disability and impairment, 8.09% (9,055,355) of the records included racial terms, 0.37% (414,151) of the collections contained phrases related to cultural awareness, 0.31% (346,991) of titles were classified using religious terminology, gender terms were used to classify 29% (32,460,483) of the titles, and terms related to sexual orientation accounted for 1% (1,119,327).

Conclusions: Libraries serve diverse communities with a variety of informational needs. If library collections do not represent the populations served, then it becomes increasingly difficult for those needs to be met. The statistical results of this project, which focused on medical library holdings, reveal how under-representative health science collections currently are. Findings show several contributing problems to this under-representation, including: some subjectivity in the diversity survey tool’s development, a lack of consistency among medical libraries cataloging practices, and issues surrounding controlled vocabularies’ use of inaccurate terminology. Despite these issues, and the potential limitations of the survey tool, one of

the main findings is that continual assessment of a collection’s diversity is essential to improve awareness of diversity, equity, and inclusion concerns.

Assessment of Learning Modules Promoting Team Science Practices to the Translational Scientist (63)

Track: Innovation & Research Practice

Bart Ragon - Director, University of Virginia Health Sciences Library, Charlottesville, Virginia

Belinda E. Hernandez

Wendy M. Novicoff - Professor, University of Virginia

Morgan Given

Catherine P. Bradshaw

Objectives: The overarching objective is to assess the value of promoting team science practices across a diverse clinical translational science community through the development of learning modules. We aim to share lessons learned to help inform best practices for CTSA hubs interested in promoting team science.

Methods: We recently created a series of self-paced learning modules focused on the science of team science, which include a variety of text and multimedia content. A preliminary assessment was conducted to determine the perceived value of six video team science Learning Shots and to identify areas for improvement. Significant content revisions are underway based on respondent feedback. In early 2022, a follow up survey will solicit feedback from a larger sample of researchers to reassess the learning modules and to ensure that desired improvements were achieved. We will incorporate continuous improvement cycles to gather future feedback, track improvements, and identify potential future direction for new content.

Results: The preliminary assessment identified the most effective aspects of the modules to be the variety and knowledge of speakers, diversity of topics, organization of the content, and appropriateness of length. Least effective aspects included a desire for more information in some content areas and not enough focus on the challenges of team science for junior faculty. Suggested areas for improvement include a desire for supplemental descriptive text, links to productivity tools, and additional examples from researchers. The follow up study is expected to yield more detailed information on the impacts of the improvements and the overall effectiveness of the modules

Conclusions: This project provides insights for libraries and CTSA Hubs interested in promoting team science and best practices when developing learning modules. Results contribute to what is known about researchers’ interest in learning about team science and the effectiveness of using online formats for delivery.

Attitudes About Learning and Teaching Critical Appraisal Among Health Sciences Librarians Over Time: A Qualitative Analysis (60)

Track: Professionalism & Leadership

Marie T. Ascher - Lillian Hetrick Huber Endowed Director, Phillip Capozzi, M.D. Library / New York Medical College, Valhalla, New York

Objectives: The purpose of this systematic review is to explore attitudes of health sciences librarians toward professional development in the field of Critical Appraisal. This is an area of opportunity for health sciences

librarians. Evidence-based practice requires the use of information throughout a cycle of ask, acquire, appraise, apply, and assess. Librarians have traditionally been trained in asking focused clinical questions, developing search strategies, and acquiring information, but only have rudimentary skills related to critical appraisal of the health sciences literature. This study seeks to identify, track, and assess attitudinal readiness for professional development in the realm of critical appraisal.

Methods: Method: Systematic Review. A systematic review using PRISMA guidelines will seek to answer the question, "Have librarian attitudes about developing critical appraisal skills changed in the last 30 years?" The authors will identify articles which discuss librarian attitudes toward professional development in the areas of critical appraisal, biostatistics, and clinical epidemiology. PubMed, EMBASE, Scopus, and Library Literature Databases will be searched using a comprehensive search strategy in each. Two reviewers will scan results particularly identifying articles which describe attitude states. Hand-searching will be completed in the Journal of the Medical Library Association and Medical Reference Services Quarterly. An analysis of trends and themes will be developed.

Results: Forthcoming. The timeline for this project will be tight but because there is not that much literature specifically on this aspect related to "attitudes," it should be easily completed. The results may be preliminary, however.

Conclusions:

Can Students Learn Too Much About Evidence-Based Medicine? Analysis of Longitudinal EBM Curriculum in Three Clinical Clerkships (12)

Track: Education

Sa'ad Laws - Librarian, Education & Research, Health Sciences Library, Doha, Ad Dawhah, Qatar

Mai Mahmoud, M.D. - Associate Professor of Teaching in Medicine / Assistant Dean for Faculty Affairs, Medical Education

Moune Jabre - Assistant Professor of Clinical Obstetrics and Gynecology, Medical Education

Amal Khidir, MBBS - Associate Professor of Pediatrics, Medical Education

Ziyad Mahfoud, Ph.D. - Professor of Research in Population Health Sciences, Public Health

Objectives: Despite a history of librarian integration in Evidence-based medicine (EBM) instruction, there is little evidence of preferred methods or repetitions to give maximum instructional benefit. A librarian and three clerkship directors worked over several years to develop a longitudinal EBM curriculum that allows students to experience multiple iterations of EBM question types, medical specialties, and analysis of different study types. Anecdotal evidence from students indicated that they valued and appreciated the librarian’s involvement, formative feedback, and varied formats of summative assessment. Despite this initial praise, instructors wanted to know if the curricular developments produced any quantitative improvements in student performance.

Methods: First-year medical students receive introductory instruction in EBM; however, they don’t have an opportunity to use EBM in a clinical setting until they reach third-year clinical clerkships. To aid student recall, each clerkship begins with an EBM e-learning module to refresh essential concepts. Each clerkship has a summative EBM assessment in the form of a written assignment or group presentation. Before this assessment, students need to submit an EBM proposal for approval. Both the project proposal and summative projects require students to incorporate different facets of EBM, including a PICO based on their chosen case and search. The librarian and clerkship directors offer formative feedback to students through

written and in-person meetings to address any gaps or deficiencies in knowledge.

Results: Both the mid-clerkship EBM project proposals and summative assessments for PICO formation and searching were assessed within clerkships and between clerkships. Analysis indicated that students improved in all clerkships between mid-clerkship and summative assessment. Students also improved longitudinally with EBM between their first and second clerkships, but not always in the third clerkship.

Conclusions: Varied and repeated exposure to EBM does have a positive effect on student performance outcomes. Students continually performed better after receiving feedback on projects. This may seem like an obvious outcome, but one that should be done more often given the effect. When considering the number of exposures students have to EBM, there appears to be a limit to the improvements that students will have. This may be due to a plateauing effect on student performance.

A Case Study of the Citation Advantage of the Open Access Papers Published by Taipei Medical University Researchers (15)

Track: Health Equity & Global Health

Shu- Yuan Siao - Head, Division of Knowledge Services, Taipei Medical University Library, Taipei

Tzu-Heng Chiu - Professor & University Librarian, Taipei Medical University, Taipei, Taiwan

Objectives: Nearly 60% of the journal papers of [institution] in 2022 are open access articles. The research purpose of the poster is to analyze the journal papers of [institution] indexed in WoS database in the period 2017-2021, intending to understand the citations impact of journal papers and the difference in the average number of citations between open access (OA) and non-OA journal papers. It is authors’ hope that research results will serve as reference for paper submission of [institution] researchers and promotion programs of [institution] in the future.

Methods: This study was based on a bibliometric analysis to 10,586 TMU papers published from 2017 to 2021 in the WoS database. The data was collected on May 11, 2022, and the Affiliation "Taipei Medical University" was searched in the Web of Science Core Collection database (including Science Citation Index Expanded, SCI-EXPANDED & Social Sciences Citation Index, SSCI). There are 6 types of OA classification (Table 1) in the WoS database, each article may receive multiple OA classifications. Authors used Excel to calculate the average number of citations of papers based on the classifications of Open Access and the quartiles of journal impact coefficients (2020 JIF).

Results: It was found that the number of papers in Open Access journals of Taipei Medical University accounted for 57.79% of the total number of papers, and the Gold OA papers accounted for 47.85% (Table 2). These 10,586 articles were cited 109,159 times, with an average citation of 10.31, while the average citation of OA journal papers was 12.46, and the average citation of papers in non-OA journals was 6.33; the average citation times of papers ranked by OA journal article type were green accepted(130.16), hybrid(77.37) and green submitted(24.08) (Figure 1). The average citation of papers in Q1 OA journals was 5 times higher than that of Q4 OA journals (20.21 vs. 4.17); The average citation of “Green Accepted” papers in Q1 journals was 186.74, the average citation number of papers in “Gold Hybrid” in Q1 journals was 119.48, and the average citation number of papers in “Green Submitted” papers in Q1 journals was 47.18 (Figure 2).

Conclusions: The bibliographic data collected in this study are limited to journal articles indexed by the WOS database of Taipei Medical University and differences in subject areas are ignored. Therefore, the results cannot apply to other institutions. In conclusion, OA journals have higher citations on average than non-OA journals. The average citation times of papers in Q1 OA journals are better than those of Q2, Q3

and Q4 journals. Green Accepted journal papers have the highest average citations. Therefore, the authors recommend that researchers of Taipei Medical University should aim for JIF Q1 and Gold Hybrid journals when submitting journal papers in the future and try to self-archive or institutionally archive their manuscripts as much as possible to increase research visibility and increase citations.

COVID-19 Publication Types on PubMed: A Bibliometric Study (65)

Track: Innovation & Research Practice

Leah Everitt - Associate Fellow, National Library of Medicine, Cincinnati, Ohio

Paul Fontelo, MD, MPH - Staff Scientist, National Library of Medicine Applied Clinical Informatics Branch, Bethesda, Maryland

Objectives: This bibliometric study will focus on COVID-19 publication types on PubMed: clinical trials, meta-analyses, systematic reviews, and randomized controlled trials. Additionally, data on the PubMed Central preprint pilot including estimated percentages of preprints eventually published in peer-reviewed journals. As academic literature retractions have become an issue the retraction rate for all PubMed publications will be compared to the retraction rate of COVID-19 publications. This study will help health science librarians understand how COVID-19 publications have affected the biomedical citation landscape and will be of particular interest to librarians who work with clinicians and researchers working on COVID-19 issues.

Methods: PubMed was searched in November 2022 for publications from 2018-2022 using a comprehensive search strategy developed by the author and reviewed by expert searchers aimed at retrieving articles pertaining to COVID-19 and SARS-CoV-2. Additional searches were used to determine how many articles were published annually, the different types of articles published, and the number of retracted publications. Then publication counts for each of the listed categories were visualized as bar graphs and tables using Microsoft Excel. Percentages of different publication types and COVID-19 literature were calculated using Excel and included in visualizations. Additionally, the percent increase in publications from year to year was also calculated when relevant.

Results: The number of PubMed citations increased from 2018 to 2021 with a 4.8%, 15.5%, and 8.6% increase each respective year. The number of COVID-19 citations also increased, in 2019 there were only 51 but by 2020 there were 91,830 citations; from 2020 to 2021 there was a 50.0% increase. Most PubMed publication types had increased numbers of citations from 2018 to 2021, the trend also occurred in COVID-19. Annual retraction rates from the past 10 years shows < 1% of citations are retracted from PubMed yearly peaking in 2019 at 0.09%. COVID-19 had fewer retractions with 0.04% in 2020 and 2021.

Conclusions: The increases in COVID-19 citations in PubMed annually were higher compared to the total citation annual increases, although not high enough to account for the trend of increasing number of citations annually. Most PubMed publication types have increasing numbers of citations until 2021. The number of COVID-19 preprints did not increase from 2020 to 2021 on PubMed. Based on estimates many preprints go on to be published as peer-reviewed articles. Over the past ten years there is an overall trend of increasing numbers of retracted publications, however the percentages of retracted publications are very low. There is not a higher percent of retracted papers in the COVID-19 subject area.

Classifying Data Management Plan Guidance using DMPTool (114)

Track: Information Management

Lucy C. Jones - Research Data & Scholarly Communications Librarian, Claude Moore Health Sciences Library, University of Virginia, Charlottesville, Virginia

Lauren Phegley - Research Data Engineer, University of Pennsylvania

Objectives: To understand the kinds of guidance R1 universities give researchers for the development of data management plans by analyzing the themed guidance text they provide in DMPTool.

Methods: DMPTool is a popular open-source online application for writing data management plans using generic and funder-specific templates. Institutions can become members of DMPTool, allowing them to provide their students, faculty, and staff with an institutional login, the ability to request feedback on their plans, and access to institution-specific guidance. 92% of R1 universities are members of DMPTool and 48% provide guidance for at least one of the 14 data management themes available in DMPTool, such as data format or preservation. This makes DMPTool a convenient source to examine the data management guidance research-intensive American universities are providing their constituents, which would otherwise likely be limited to their respective websites. For this study, themed guidance text provided by 70 R1 universities was copied from DMPTool into Excel. Each link in the text blocks was then coded according to which of nine destination types it pointed to, such as a library website, university research website, or an outside resource. The average text length and number of links were compiled, as well as the most popular guidance themes and the most common linked resources.

Results: The analysis of 490 themed guidance text blocks from 70 R1 institutions showed that guidance text length ranged from 2 words to 2,259 words, and the largest number of links provided in one text block was 31. Universities provided guidance on an average of 6.93 out of the 14 possible themes. Data sharing was the most common theme, while data collection was the least common. The three most common link destinations were the university’s library website, an independent outside resource, and a university research website, and the three most shared outside resources were re3data, the journal *Nature*, and DataOne.

Conclusions: This study found a wide variety of approaches to advising researchers on writing data management plans, from minimal to detailed guidance. Although best practices for research data management exist, there is no standard for how to deliver this guidance to researchers, especially in an interactive environment like DMPTool which presents unique challenges for information delivery. The assembled text corpus offers opportunities for further research utilizing topic analysis to evaluate the text on a more granular level. Additionally, this dataset could be compared to a second snapshot taken at a later date to see how data management plan guidance shifts over time or compared to guidance provided by a different set of institutions.

Did You Test the Search First? Analyzing the Prevalence of a Pilot Search in Expert Searching Publications (39)

Track: Information Services

Rachel Lane Walden - Health Sciences Informationist, Annette and Irwin Eskind Family Biomedical Library and Learning Center, Nashville, Tennessee

Kayce D. Gill - Health Sciences Collections Librarian, Annette and Irwin Eskind Family Biomedical Library and Learning Center

Heather E. Laferriere - Health Sciences Informationist, Annette and Irwin Eskind Family Biomedical Library and Learning Center, Nashville, Tennessee

Camille L. Ivey - Health Sciences Informationist, Annette and Irwin Eskind Family Biomedical Library and Learning Center, Hermitage, Tennessee

Rachel Davis, MLIS - Document Delivery Services Assistant, Annette and Irwin Eskind Family Biomedical Library and Learning Center

Philip D. Walker - Director, Annette and Irwin Eskind Family Biomedical Library and Learning Center, Nashville, Tennessee

Objectives: While our library does not have a formal Expert Searching Service, the Information Services Team is constantly asked to collaborate on systematic reviews, scoping reviews, and meta-analyses (SR2MAs). As a form of professional development, the team occasionally meets to share information on new expert searching tools, standards, and trends, and dissect publications. These meetings led to the development of a search standard for our library. One standard item is performing an initial or pilot search prior to searching all appropriate databases. In reviewing the literature, we discovered this essential first step is rarely mentioned, thus prompting the need for further evaluation.

Methods: In the Fall of 2022, we searched PubMed (NCBI) to find SR2MAs authored by researchers from our institution from 2017–2022. The search strings and results were evaluated and revised until we reached consensus. We developed inclusion and exclusion criteria with the main objective of identifying if the author(s) mentioned performing an initial or pilot search prior to searching all appropriate databases. We further analyzed the presence of an information professional, internal or external, on the research team as a co-author or acknowledgement. We utilized Covidence [1] to evaluate the results and extract data which met our criteria.

Results: The mention of an initial search was found in only 18 of the 631 analyzed reviews. Reviews that reported working with an information professional (25% of included reviews) had a higher percentage mentioning an initial search, 10 out of the 18. Also of interest is the varying degrees of attribution: 53 mentioned an information professional in the text only, 52 included an information professional by name in the acknowledgements section, and 51 included an information professional as an author. The most used guidelines/frameworks were PRISMA, MOOSE, Cochrane Handbook, and Arksey and O'Malley Framework for Scoping Reviews. A total of 28% of the reviews did not list the use of any guidelines/frameworks when designing or performing their review.

Conclusions: We identified a lack of standard documentation and reporting of the search process for SR2MAs. Khan’s book on systematic reviews [2] discussed how comprehensive literature searches are iterative and strategies will need refinement. He stated, “for transparency, it is essential to be explicit about the modifications and indicate which questions were posed a priori and which were generated during the review work” [p 17]. Several research reports account for improved search reproducibility and lower risk of bias when a librarian is involved with evidence synthesis publications [3,4]. The Cochrane Handbook, 2011 guidelines from the National Academy of Medicine, and the PRISMA Extension on Reporting Literature Searches [5-7] also recommend including a librarian in the review process. The ICJME guidelines on defining authorship and contributors [8] and the COPE authorship and contributorship guidance [9] go a step further in advocating for librarians to be recognized as authors or included in the acknowledgements depending on their levels of contribution provided to the final publication. Based on our findings, our next steps are to develop a standard statement about the pilot search process to include in the methods section of manuscripts and offering workshops on review types to increase awareness of appropriate methods.

Discoverability of supporting research materials for U.S. federally funded COVID-19 clinical studies registered in ClinicalTrials.gov (129)

Track: Innovation & Research Practice

Paije Wilson - Health Sciences Librarian, Ebling Library for the Health Sciences (University of Wisconsin-Madison), Cottage Grove, Wisconsin

Vojtech Huser, MD, PhD - Director, Medical Informatics & Data Management Lead, Pfize

Objectives: Access to supporting research materials increases the transparency and utility of clinical study research. The objective of this study was to evaluate the discoverability of supporting research materials in U.S. federally funded COVID-19 ClinicalTrials.gov (CTG) records with at least 200 participants. More specifically, the study evaluated the records for discoverability of: (1) supporting documents, including protocols, informed consent forms, and statistical analysis plans; (2) information relating to whether and how outside researchers may access full, deidentified individual participant data (IPD); and (3) associated publications.

Methods: To isolate federally funded COVID-19 studies in CTG, the COVID-19 and the Funder Type filters were applied in CTG. Results were exported as a CSV, and Excel was used to filter out records containing fewer than 200 participants, resulting in 206 records. Using the "Study Documents" column of the Excel sheet, data were collected on whether CTG records provided links to protocols, informed consent forms, and/or statistical analysis plans. In the "Individual Participant Data (IPD) Sharing Statement" section of each CTG record, data were collected on the researchers' intentions to share and, when applicable, how they planned to share IPD. In the "More Information" section of each CTG record, data were collected on the presence of linked publication records. Using a decision tree, publications were also evaluated for whether they were full, original results publications for their associated CTG record. Additional data were collected on whether the publication record linked back to the CTG record, and whether the publication record could be retrieved by searching the study's NCT number in PubMed (using [si] and [tw] field tags).

Results: Of the 206 CTG records, only 9% linked to protocols, 8% to informed consent forms, and 9% to statistical analysis plans. 55 (27%) indicated they planned to share IPD, with just 25 providing sufficient information to access IPD. The majority of records (61%) didn't link out to any publication records, and only 43 (21%) linked out to at least 1 results publication. Of the 479 links to publications, the majority (86%) were to non-results publications. Most results publications were automatically linked (92%), and were interspersed with non-results publications, complicating their differentiation. All publications linked back to their CTG records, and most results publications (97%) were retrievable by searching NCT number in PubMed, with lower retrieval for non-results publications (10%).

Conclusions: With the release of the Office of Science and Technology Policy's 2022 memorandum, "Ensuring free, immediate, and equitable access to federally funded research", which calls for the public sharing of outputs deriving from federally funded research, considerations relating to the discoverability of supporting research materials has never been more vital. This research provides insight into the landscape of current sharing practices for supporting research materials from federally funded COVID-19 clinical research in CTG, and aids in the identification of shortcomings that may need to be addressed through policy and/or educational measures. This will not only help researchers and librarians to be better informed and prepared for federal sharing policies, but will help researchers to make their findings more discoverable, transparent, and utilizable.

Evaluation of Awareness and Approach of Medical Sciences Postgraduate students in Qazvin about Feasibility of Cloud Computing Development in academic Libraries (117)

Track: Information Management

Shahrzad Gholami, n/a - Director of Medical Information and scientific Resource Department, Qazvin University of Medical Sciences, Qazvin, Qazvin, Iran

Masumeh Karbala Aghaei Kamran, n/a - Assistant Professor & Director of Information Science and Knowledge Department, Library and information science Department, Educational Sciences and Psychology faculty, Tehran, Tehran, Iran

Objectives: Universities have started adopting cloud computing technology as cost-effective tool for storing, accessing and sharing data the. the aim of this study was to evaluate postgraduate student’s awareness and satisfaction about cloud computing in the XXX University of Medical Sciences, XXX.

Methods: This applied research was carried out using a descriptive survey method to evaluate of rate of awareness and satisfaction of cloud computing in medical libraries was used a questionnaire. The questionnaire’s reliability was confirmed at the Cronbach’s alpha of 0.914. The data were analyzed by SPSS software ver.20 and using Spearman correlation coefficient and Kruskal Wallis test.

Results: Among 53 participants in the study, 75% of them were female and 25% male. Based on result the 50% of participants did not have any information about the cloud computing function. The 62.3% of them were believed that the use of cloud space among the information services of medical libraries can be very useful and efficient.

Conclusions: According to the medical sciences postgraduate students' opinion, using the capacities of cloud space in university libraries can be very useful and efficient; Because, based on the structure of the cloud space, the cloud computing techniques and methods applied to libraries and academic environment not only can improve the quality of services and utilization of resources, but also it can provide the sharing of software, improvement of data storage and management and facilitate the use of information resources from anywhere. According to the findings of research on the feasibility of using more cloud space, there are still significant platforms and technological infrastructure and data protection protocols in XXX medical academic libraries that have not been addressed and need more research.

Evaluation of Child Injury Surveillance System in Nigeria Hospital (130)

Track: Innovation & Research Practice

Moyosore Adegboye - Phd student, School of computing and information, Pittsburgh, Pennsylvania

Sneha Anil Kumar Vaidhyam, n/a - Phd student, School of computing and information

Objectives: The purpose of child injury surveillance systems is to collect data on injuries among children, identify risk factors associated with those injuries, and develop interventions to reduce child mortality rates. This paper evaluated the current child injury surveillance system in a hospital in Nigeria as it relates to data collection, and the accuracy of the information collected, and directly examined outcomes achieved using this system.

Methods: A qualitative study was designed and conducted to evaluate the child injury surveillance system used in a rural hospital in Nigeria. Data was gathered through an interview with 20 experts working in the

hospital using an unstructured questionnaire. Interview transcripts were analyzed using the content analysis method on NVivo.

Results: The content analysis identified four items in the current system: the lack of proper data collection and analysis resulted in a significant gap between what is known about child injuries, inadequate monitoring which revealed that there is a lack of reliable data to identify high-risk areas, limited resources in infrastructure leading to many cases going unreported or misclassified and financial constraints for medical professionals.

Conclusions: Investing more resources into building an effective child injury surveillance system so accurate that data can be collected regularly which will then allow us to target specific high-risk groups/areas accordingly while also increasing public education campaigns around safety practices as well as training health workers on appropriate protocols when dealing with pediatric trauma patients. Therefore, only then can we hope to see any significant improvement outcomes related to reducing the child incidence rate in Nigeria.

Existence of a Library and Degreed Librarian at ANCC Magnet Recognized Hospitals (121)

Track: Information Services

Louisa Verma, AHIP - Reference Librarian, Portland Cement Association, California

Objectives: The primary objective of this study is to look at the library landscape of Magnet-recognized hospitals, particularly with respect to the existence of an onsite library and degreed librarian. This preliminary examination hopes to define any distinctive features of Magnet hospital libraries and librarians and to be a point of reference for future studies advocating that librarian support is crucial to the support of nurses and evidence-based practice in Magnet hospitals.

Methods: The ANCC Magnet Recognition website provides a downloadable spreadsheet listing of current and former designated Magnet facilities. Using this spreadsheet along with the NNLM Member Directory, hospital/health system websites, LinkedIn, the MLA Directory, and posting to the MLA Hospital Librarians mailing list, the author gathered the following data points for the Magnet facilities listed: a) existence of onsite library, b) library employs at least one degreed information professional, c) facility affiliated with an academic library, and d) facility has access to a librarian as part of a health system.

Results: This survey of 659 Magnet certified/prior-certified hospitals found that over 80% provided an onsite or virtual library, over 77% were staffed by one or more Master's degreed librarians, almost 30% were affiliated with an Academic institution and almost 68% were affiliated with an umbrella health system.

Conclusions: This exploration aims to shed light on the previously unstudied correlation between Magnet-recognized initiatives for nursing research/evidence-based practice and the informational professionals who work to support these initiatives. It is the author's hope that this research project will provide new evidence to the growing literature on the value of hospital libraries, so that hospital librarians can continue to advocate for, market, and communicate their supportive role in their institutions.

Exploring Links in the Library Value Chain: Does Collection Use Lead to Scholarly Output? (67)

Track: Innovation & Research Practice

J. Michael Lindsay, AHIP - Head of Collections & Access Services / Associate Professor, Preston Medical Library, University of Tennessee, Knoxville, Tennessee

Martha Earl, AHIP - Director & Professor, University of Tennessee, Preston Medical Library, Knoxville, Tennessee

Objectives: Value chain analysis is a business tool used to determine what activities in a process add to or take away from the customer's perception of the value of a product or service. For one academic library in a hospital setting, regular analysis of usage has been done for many years to determine what serials and databases are being used and which are not. To evolve and expand the library's view of the value collections provide, this study was initiated to explore correlations between library serials subscription usage in general and specialty areas and the school's scholarly output in these areas.

Methods: The library has prepared cost/use studies on serials subscriptions for the past 14 years, retaining the data from these studies. Focusing on the pre-COVID period (2017-2018), and the peri-COVID period (2020-2021), the study began by comparing total serials use for these periods, and then serials use changes in specialty areas, including Surgery, Critical Care, Pulmonology and Family Medicine. Following this, a search of scholarly publications for the institution's faculty members was conducted for the two periods in PubMed. The results were then reviewed and classified according to medical specialty and institutional department. These datasets were then analyzed for correlations by a statistician.

Results: Initial results showed that serials in critical care saw a substantial increase in use over the two periods. In terms of scholarly output, there was a noticeable increase in publications, with almost all departments increasing their output. Regression analysis showed statistically significant correlations between serials use and scholarly publications for the time periods and medical specialties examined.

Conclusions: Libraries spend as much as 50% of their budgets on resources, reflecting in part the importance of serials to patrons. By correlating serials use with scholarly productivity, librarians gain more evidence of contribution to the scholarly enterprise.

A Field Normalized Exploration of Awoken Papers (61)

Track: Innovation & Research Practice

Sam Hansen - Mathematics & Statistics Librarian, University of Michigan, Ann Arbor

Objectives: In citation analysis there is a type of paper that holds particular interest: these papers receive little to no citations for years, after which scholars start citing them at a high rate. These papers have traditionally been referred to as “sleeping beauties,” a name problematic for its gendered implications and its exclusionary Eurocentric nature. For these reasons, I refer to them as “awoken papers.” Previous studies of awoken papers have been based off raw counts, but in this poster I explore the impact field normalization and limiting to highly cited papers has on the rates of awoken papers in different disciplines.

Methods: I computed sleeper coefficients using the method described in Ke, Ferrara, Radicchi, and Flammini's 2015 paper, Defining and identifying Sleeping Beauties in science, for all of the articles in Clarivate's Web of Science from 1900-2017. Similarly to Ke et. al. I then defined awoken papers as those with the highest 0.1% coefficients. Then I normalized the number of awoken papers per subject by the total number of articles indexed in Web of Science. I also investigated the impact number of citations has on awoken papers by looking at the sets of papers with at least 25, 100, 300, and 500 citations.

Results: Normalizing the number of awoken papers by the number of papers published in the different subject areas drastically changes the areas which rank as the highest producers of awoken papers. The top 10 changes from large, multidisciplinary science and medical disciplines to more mathematical and social

sciences disciplines. Limiting by citation counts continues to show the very large prevalence of awoken papers within the mathematical article corpus. For instance mathematical papers with at least 100 citations, over 11% of them at awoken papers, which is an order of magnitude higher than any other subject.

Conclusions: The results of this exploratory analysis show that using raw counts of awoken papers has a strong effect on which subjects are likely to create them, Specifically it appears that the reason scientific and medical fields are thought of as producing many of them is more related to the glut of publications in those subjects and not that they are actually more likely to create work that will go unnoticed before becoming important. In particular it appears that an article being in an area related to the mathematical sciences is the most likely predictor that an article may become an awoken paper.

Focus on Health Information Support from Library Collaborations in Support of Their Communities – a Literature Review (109)

Track: Health Equity & Global Health

Marlowe Bogino - Clinical and Reference Librarian, CMSRU Library/ Rowan University, Wilmington, Delaware

Objectives: What is in the current literature surrounding collaborations between different types of libraries who focus on outreach to health information support for communities?

Methods: Searching the databases of Proquest’s Social Science database and Library Science database, PubMed and Ebsco’s Academic Search complete databases using a combination of keywords and controlled vocabulary for published articles focused on the topic. Limited findings to the English language and included articles that specifically focused on different library types i.e., public academic or hospital libraries which worked together to serve communities to support health. Prepared poster using the key findings from the literature review.

Results: From my search, I found a relatively small number of articles with the specific focus of collaboration between libraries. Though the number of articles was few, there was a great amount of information on tips to consider when engaging in partnerships with communities such as funding, selecting partnerships and community needs assessments. There were also many examples of programs and initiatives that occurred from the shared knowledge and strengths that various types of libraries brought to community outreach initiatives. The examples provided excellent ideas for future endeavors for creating partnerships with fellow librarians.

Conclusions: Specifically interested in the partnerships that exists between various types of libraries such as hospital or health or academic libraries who aim to support health focused initiatives for their communities and community members, I conducted a literature search to find out what existed within the published literature on this topic. The findings of the search yielded a small number of articles with this particular focus, but I gained key take aways from the articles which included tips for participating within community partnerships and examples of programs and initiatives to help guide future collaborations.

Forging Ahead Towards the UN’s Sustainable Development Goals: A Visual Analysis of Publications on Gender Equality in Biomedicine (23)

Track: Health Equity & Global Health

Patricia Smith - Product Specialist, Digital Science

Shannon O'Reilly, MPS - Product Specialist, Digital Science

Carlos Areia - Data Scientist, Digital Science

Objectives: The UN’s Sustainable Development Goals (SDGs) are part of a global effort to, “improve health and education, reduce inequality, and spur economic growth,” through 17 focus areas. Our aim is to demonstrate the value of Open Access (OA) and how it can help achieve the UN’s goals, with a focus on SDG 5 Gender Equality in the field of Biomedical and Clinical Science. We will evaluate publications classified under SDG 5 and analyze their OA status and Altmetric data; we will report on the top institutions and researchers contributing to this goal and their respective openness.

Methods: Using Dimensions and Altmetric Explorer as data sources, publications were limited to years 2016-2023, and by institutions in the United States and Canada. Publications were filtered under SDG 5 Gender Equality, and Fields of Research classification was used to limit publications to the field of “Biomedical and Clinical Science.” A bespoke dataset containing Dimensions and Altmetric data was created in Google BigQuery and results were analyzed in Google Data Studio and Tableau through interactive dashboards. Descriptive statistics were explored by mean and standard deviation comparisons. Visualizations, such as choropleth maps, radar charts, and bar and line graphs, will be highlighted on the poster, along with the relevant datasets.

Results: The institutions producing the highest volume of publications (Harvard, Johns Hopkins, and UCSF) were not necessarily the institutions with the most open publications (UCSD, Columbia, UCSF). The most prolific individuals were from UCSD, University of Michigan, and Vancouver General Hospital. Broadly, these publications were popular with the masses—75% of publications received online attention tracked by Altmetric. While a slim majority (56%) of the publications were OA, 44% of publications were closed access, revealing that much of the research is not accessible to many. Altmetric data reveal that OA publications received much more attention than their closed access counterparts.

Conclusions: Gender equality in healthcare is a widespread issue, with publication topics ranging from domestic violence to a lack of representation in medical education. These publications are popular with the public, and receive widespread news, policy, and social media attention tracked by Altmetric, with OA publications receiving more attention overall. However, a large percentage (44%) of publications are closed access. The aim of SDGs is to improve health and reduce inequality, and the closed nature of many of these publications is in opposition to that goal. This, along with the more prolific dissemination and online attention of OA research, can serve as another call to action to push towards open access.

Forging a Connection Between East Tennessee Rural Communities and Health Information Using Little Free Libraries (110)

Track: Health Equity & Global Health

Alexandria Q. Wilson, AHIP - Assistant Professor/Research & Learning Services Librarian, Preston Medical Library & Health Information Center, Knoxville, Tennessee

Kelsey L. Grabeel, AHIP - Associate Professor/Assistant Director, Preston Medical Library & Health Information Center, Knoxville, Tennessee

Objectives: To discuss how medical librarians at Preston Medical Library used already established Little Free Libraries (LFL) as outreach opportunities to provide consumer health books to rural locations in East Tennessee’s Appalachia region and connect the community to their local consumer health library. Success of the project was measured based on how many books had been taken from the LFLs and consumer health

information requests from the corresponding zip codes.

Methods: Researchers analyzed regional counties and zip codes by reviewing the population’s literacy levels, the Index of Medical Underservice scores, and the availability of already established LFLs. Twenty-two already established LFL locations were selected and 1 new LFL was built for a community eye clinic. Eleven health books on pertinent health topics were purchased for each established LFL, and 33 books were purchased for the new LFL. A bookmark with information about the local consumer health library and health information service was included with each book. Researchers went back to each location to examine books taken five months after initial delivery and pulled data from an internal consumer health information request database that collects zip codes for analysis.

Results: In total, 231 books were delivered to already established LFLs, and 90% (n=208) of the books were taken from those locations at time of follow-up. 25 consumer health requests from the LFL zip codes have been received as of December 2022. The new LFL was successfully built at the eye clinic and the optometrist provides regular updates about usage.

Conclusions: By distributing health books to already established LFLs, researchers brought relevant health information books to rural and medically underserved communities and increased awareness of the consumer health library as seen from the quantity of books taken and the zip code information from the consumer health information database. The new LFL allowed for a partnership between the library and a rural eye clinic. Given the global reach of LFLs, this project could be replicated within other communities.

Giving It All You’ve Got: The Value of Extending Hours in an Academic Health Sciences Library (41)

Track: Information Services

G.J. Corey Harmon, AHIP - Head of Access Services/Library Assistant Professor, Laupus Health Sciences Library, East Carolina University, Greenville, North Carolina

Kerry Sewell - Research Librarian/Library Associate Professor, Laupus Health Sciences Library, East Carolina University, Greenville, North Carolina

Objectives: Students regularly state strong preference for increased library operational hours to accommodate learning needs. While many academic libraries provide extended hours, academic health sciences libraries appear to adopt such models less frequently. Budgetary constraints and lower gate counts in health sciences libraries may drive this, yet student preference remains strong. In response to student preferences for increased hours, our institution shifted its budget to provide an additional 10 weekend hours (Friday through Sunday). This study assessed changes in physical use of the library over weekend days, elucidating the relationship between stated preference and actual use.

Methods: This study utilized physical library usage data [gate counts, room reservations, and circulation transactions] for the time period January 2018-January 2020, restricted to Fridays, Saturdays, and Sundays. The date range allowed for comparison of weekend physical use statistics during one complete year pre-extended hours with one complete year post-extended hours data. For the two time periods, for all periods except exams, we ran descriptive statistics to assess the percent increase in physical library usage, with sub-analyses by patron type whenever data allowed.

Results: Overall, we saw increases in all three areas of physical usage data studied. Our gate count increased by 17% on the days that we extended our hours, with entrances during extended hours accounting for 21%, 11% and 8% of total daily entrances on Sunday, Saturday, and Friday, respectively. Room reservations during the extended hours in 2019 accounted for 17.42%, 20.98%, and 18.92% of all reservations during the Spring, Fall, and Summer Semesters respectively. Following the increase in hours,

our circulation increased by 32.92% with Saturdays seeing the biggest jump (39.66%) followed by Sundays (34.66%), and Fridays (22.86%).

Conclusions: Based on this data, we determined that the expressed student preference for increased operational hours was matched by actual increased physical use of library spaces and resources during extended hours.

Health Sciences Faculty Publication Patterns and Related Information Seeking Behavior (69)

Track: Innovation & Research Practice

Sandra L. De Groote, AHIP - Head, Assessment & Scholarly Communications & Professor, UIC University Library/ Assessment & Scholarly Communications, Chicago, Illinois

Jung Mi Scoulas - Assessment Coordinator & Assistant Professor, UIC University Library/ Assessment & Scholarly Communications, Chicago, Illinois

Objectives: Since 2017, the University Library at a large Urban University has surveyed faculty every two years to follow their changing needs and perspectives on library resources, services, and roles. In addition, a long-term retrospective study explores how faculty publication patterns have changed over the years. Using these two data sources, this study explores how health sciences researchers’ publication patterns have changed over time, the relationship between productivity and the use of literature in journal articles, and how health sciences faculty productivity relates to their information seeking behavior and perception of the academic library.

Methods: To explore how faculty publication patterns have changed over time and examine the relationship between literature use and productivity, faculty publications were examined. The citations of publication of Health Sciences faculty who had been at the institution for at least 15 were obtained from Scopus. In addition, the number of references and the number of co-authors for each publication was captured. Faculty productivity was calculated by tabulating total published articles for each author. To examine faculty’s perceptions on their use of and the importance of library resources, a faculty survey was distributed in Spring 2022. The 12-question survey included open-ended and multiple-choice questions on the use and importance of library resources and services related to faculty research. Demographics (college, rank, years at institution) and publication data from the past 5 years (number of articles, conference proceedings, books, and book chapters) was also captured.

Results: Overall, publications per author, references per publication and the co-authors per publication increased over time. Less productive faculty (under 20 articles) used less references in their publications; prolific authors (over 52 articles) included less references in their publications compared to productive authors. Survey results indicate almost all health sciences faculty reported using online journals at least once a month and ranked them very important to their research. Most faculty use A&I databases at least once a month and value them as very important to their research. The importance and use of other library resources varied among the disciplines.

Conclusions: Faculty’s productivity varies by health sciences discipline, as does their use of the literature. The increase in availability of online and open access journals and online databases has likely played a role in the increase the literature included in publications. Literature use also varied by faculty productivity discipline. This study demonstrates that the library continues to play a critical role in supporting faculty research.

Improving PubMed for the Novice at the Expense of the Expert: Surveying Librarians 3 Years Post-New PubMed (38)

Track: Information Services

Tim Kenny - Medical Librarian, MaineHealth, Portland, Maine

Heather Kemp - Medical Librarian, MaineHealth, Portland, Maine

Objectives: To examine and analyze current attitudes and opinions of health science library professionals regarding PubMed three years after the 2019-2020 overhaul. Following the update of PubMed, anecdotal discussions across medical library listservs, professional meetings, and additional informal communications indicated a degree of dissatisfaction with changes made to the PubMed interface as well as the back-end search customization mechanisms. Concerns include but are not limited to: loss of precision searching options, reliability of reproducible search results, increasing appearance of predatory titles within results, and valuing appearance over functionality.

Methods: Using a mixed methods approach, health science library professionals will provide feedback on the new PubMed via a web-based survey. Survey questions will include multiple choice, Likert scale, as well as open response formats. The survey is anticipated to take no more than five to ten minutes to complete. Questions will be worded in a neutral fashion to avoid any leading of the responses. Participants will be given three weeks to respond to the survey. Those surveyed will include graduate degreed librarians and other library professionals who regularly engage with PubMed. Reponses will be analyzed quantitatively and qualitatively through thematic analysis of open-ended answers.

Results: Results will provide insight into the view of health science library professionals on the current iteration of PubMed. Both positives and negatives gathered from the results will be discussed along with any gaps or areas for improvement within the platform and interface. Results will be presented in both narrative and visual formats best aligned for a poster presentation.

Conclusions: PubMed is a central and critical tool for health science library professionals as well as the wider biomedical community. Given anecdotal observations and feedback, there appears to be lingering underlying dissatisfaction with certain aspects of the new PubMed three years after its debut. A formal examination of areas of dissatisfaction, gaps, and avenues for improvement may aid in future improvements and development of PubMed. Further surveys of non-library biomedical professionals may prove a useful focus for further research on the new PubMed.

Mapping Diversity, Equity, and Inclusion Research in Medical Education Journals: An Exploratory Bibliometric Analysis, 2018-2022 (111)

Track: Health Equity & Global Health

Mirian Ramirez - Research Metrics Librarian, Ruth Lilly Medical Library, Indianapolis, Indiana

Levi Dolan, MLIS - Data Services Librarian, Ruth Lilly Medical Library, Indianapolis, Indiana

Objectives: More medical schools are incorporating Diversity, Equity, and Inclusion (DEI) competencies into their institutional practices as the academic medicine community support programs and projects designed to build cultural humility in medical education, clinical care, and research. This study aims to identify and analyze items covering DEI-related topics published in medical education journals in the last five years (2018-2022). We performed a bibliometric analysis investigating the overall patterns of published articles, including the annual publication distribution, distribution by journal, and analysis of keywords. This approach

aims to contribute to a better understanding of the characteristics of DEI-related research in medical education.

Methods: We conducted a bibliometric analysis on articles published in a set of 56 core medical education journals indexed in Pubmed from 2018 to 2022. The searches were conducted in April 2023. To retrieve and gather citations for published articles, we used the Pubmed database. First, we used the NLM catalog to search and identify the subset of journals that are referenced in NCBI database records classified with “Education, Medical”[Mesh] OR "education"[MeSH Subheading]) in the English language. Next, we developed and executed a comprehensive search to find articles that contained terms related to DEI; we used the Association of American Medical Colleges (AAMC) "Diversity, Equity, and Inclusion Competencies Across the Learning Continuum" (https://store.aamc.org/downloadable/download/sample/sample_id/512/) as a guide to identifying the terminology to use in the search. We used Excel to aggregate, clean up and analyze the data, and VOSviewer software was utilized to generate the athor keyword co-occurrence analysis and generate the visualization map.

Results: For the 2018-2022 time frame, 3,107 (out of 32,898, 9.4%) articles covering DEI topics were published in the selected journals in the field of medical education. Publication distribution pattern of the articles published on DEI topics has maintained a steady pattern in publishing research on DEI (average of 9.4% per year); 621 documents on the topic were published on average per year. Academic Medicine, Health Policy, and BMC Medical Education, published the most in DEI topics (32%), with more than 240 publications per journal. Author keywords will be extracted for co-occurrence analysis to identify topic trends.

Conclusions: This poster shows an examination of articles about DEI subjects written in medical education journals over the previous five years and indexed in Pubmed. We outline the characteristics of the top journals that published the research and topic trends of articles. The findings may support researchers and faculty in the health sciences disciplines when making decisions for developing a publication and research strategy. Researchers who seek DEI pathways to meet their promotion and tenure criteria will also benefit from understanding the results of this analysis.

Mapping Labor and Delivery or Birthing Centers and Neonatal Services Associated with Hospital Services in Seven States (9)

Track: Clinical Support

Julia M. Esparza, AHIP, FMLA - Associate Director/Professor, LSUHS Library, Shreveport, Louisiana

Elliott M. Freeman - Research and Writing Librarian, LSUHS Library, Shreveport, Louisiana

Sarah P. Jackson - Education Librarian, LSUHS Library, Shreveport, Louisiana

Erin Ware, MLIS - Reference Librarian, LSUHS Library, Shreveport, Louisiana

Dymaund Farmer, n/a - Student Researcher, LSUHS Library

Abigail Green, n/a - Medical Student Researcher, LSUHS Library, Shreveport, Louisiana

Kerrington Jones, n/a - Student Researcher, LSUHS Library, Shreveport, Louisiana

Christopher Schmoutz, Ph.D. - Assistant Professor, Department of Pharmacology, Toxicology and Neuroscience, Shreveport, Louisiana

Objectives: The US has increased maternal morbidity (https://bit.ly/3gSvbyA). Literature points to a lack of access to labor and delivery and neonatal care as key drivers of this issue (https://doi.org/10.26099/411v-9255). This study identifies facilities that provide labor and delivery or birthing center services and of those, which supply neonatal care, in Arkansas, Kansas, Louisiana, Missouri, Nebraska, Oklahoma, and Texas. The goal is to map this data with census populations to create geospatial service maps in these states.

Methods: This is a minimal risk, anonymous (no individual names collected), cross-sectional study. After obtaining IRB exemption, the team obtained lists of licensed healthcare facilities from state government websites or hospital associations. The researchers examined websites, obtained state lists and contacted healthcare facilities and birthing centers for whether they had labor and delivery departments or a birthing center, whether they handled high-risk deliveries, and whether they had a neonatal unit. Healthcare facilities supplying high risk delivery services and neonatal care were considered comprehensive care centers. If information was not found on websites, facilities were called to try to obtain the information. Afterwards, population data was obtained from the U.S. Census. Both data sets were converted to latitude and longitude using geocodio. Using QGIS (3.26 Buenos Aires version), both the population data and the zip codes of facilities were geospatially mapped.

Results: A total of 1,332 healthcare facilities were evaluated. Facilities providing labor and delivery or birthing services totaled as follows: Arkansas 37, Kansas 60, Louisiana 41, Missouri 60, Nebraska 49, Oklahoma 41, and Texas 217. The number of facilities providing comprehensive care services were: Arkansas 20, Kansas 21, Louisiana 31, Missouri 28, Nebraska 15, Oklahoma 14, and Texas 132. Overall, 38% (505) of the facilities provided general labor and delivery or birthing services, and only 20% (261) provided comprehensive care.

Conclusions: The heat map allows us to compare population density with availability of these services. While underserved areas are often in low-population regions, each state has areas which show significant population but minimal or no services available without a long drive. Due to data collection issues with inconsistent website designs and unresponsive or non-participatory facilities, these results may not fully reflect the availability of services. The data may also misrepresent actual availability; of those facilities that supply labor and delivery services, some may have only 1-3 beds.

Mapping the Literature of Nursing Education: An Update (45)

Track: Information Services

Carolyn Ching Dennison, AHIP - Librarian, University of Hawaii at Manoa Library, Hawaii

Objectives: Since the 1997-1999 publication of articles analyzed by Margaret (Peg) Allen, Melody M. Allison, and Sheryl Stevens’s study "Mapping the Literature of Nursing Education,” two significant changes have occurred in nursing education: the growth in the number of Doctor of Nursing Practice (DNP) programs and the effort to increase the number of nurses with undergraduate and graduate degrees. The goal of this study is to update the previous study by mapping the literature of nursing education in order to identify its core journals and databases.

Methods: This study utilizes the mapping protocol developed by NAHRS and updated in December 2017. Five core nursing education journals were selected as source journals. The original study’s three source journals were incorporated into this study: *Journal of Continuing Education in Nursing*, *Journal of Nursing Education*, and *Nurse Educator*. Two additional source journals were included for this update based on suggestions mentioned in the original study: *Journal of Professional Nursing* and *Nursing Education Perspectives*. An analysis of the citations of the journals' articles from 2016 to 2018 was done along with an analysis of database access to the journals that were cited the most.

Results: A total of 29,991 citations appeared in the five source journals. Most of the citations (69.6%) were from journals with the remaining coming from books (13.5%), government documents (3.4%), the Internet (2.2%) and miscellaneous sources (11.3%). Almost half of citations were published between 2011 and 2015 (48.0%) and a third between 2001 and 2010. Journal article citations came from 2,679 journals. Eight core journals were the source of one third of the journal citations. Another third came from 91 journals. The

remaining third were from 2,580 journals. CINAHL, PubMed/Medline and Scopus covered all of the core journals, followed by Science Citation Index and Social Science Citation Index covering seven titles.

Conclusions: Like its predecessor, the findings of this study will be useful to nurse educators and researchers, librarians supporting nursing education programs, and database providers. The study demonstrated that citation patterns in the late 1990s are relatively the same in the mid-2010s. Journals remain as the most cited format. Authors primarily cite items published within the last one to 15 years. The core journals used in the late 1990s continue to be highly cited in 2016-2018. Since 1999, new resources have gained importance in the area of nursing education, notably the journals *Nurse Education in Practice* and *Clinical Simulation in Nursing* and the database Scopus.

Reference
Allen MP, Allison MM, & Stevens, S. Mapping the literature of nursing education. J Med Libr Assoc. 2006 Apr;94(2 Suppl):E122–7.

Mind the Gap: Understanding Coverage Breaks of Newly-Launched Journals of Engineering and Computer Science in Various Databases (123)

Track: Information Services

Yuening Zhang - STEM librarian, Kent State University Libraries, Ohio

Dylan Yu, n/a - Student, Oberlin College

Objectives: This project examines how quickly Google Scholar and three other major multidisciplinary databases (Web of Science, Scopus, and Academic Search Complete) index newly added journals in engineering and computer science.

Methods: The newly added journals launched in 2020, 2021 and 2022 will be from the Association for Computing Machinery (ACM) and the Institute of Electrical and Electronics Engineers (IEEE). The number of articles published (including early access articles) will be identified for each journal. The number of articles and percentages indexed by each databases will be calculated and compared.

Results: Among these databases, Google Scholar is expected to demonstrate the highest coverage of published and early access articles, while Web of Science the least.

Conclusions: The result will be helpful to information professionals when evaluating databases or teaching users how to use them.

Open Access Viewpoints and Self-Archiving Decisions of Public Health Faculty (73)

Track: Innovation & Research Practice

Kayla M. Del Biondo - Simbonis Librarian for Public Health, Harvey Cushing/John Hay Whitney Medical Library, Yale University, New Haven, Connecticut

Holly K. Grossetta Nardini - Associate Director, Harvey Cushing/John Hay Whitney Medical Library, Yale University, New Haven, Connecticut

Kate Nyhan - Research and education librarian for public health, Harvey Cushing/John Hay Whitney Medical Library, Yale University, New Haven, Connecticut

Objectives: This study explored what faculty at a school of public health know about open access and whether they self-archive a version of their publications ("green open access"). Our secondary objectives were to uncover barriers to self-archiving for public health researchers, inquire about the information-seeking behavior of primary faculty when they need to find a particular journal’s open access fees or policies, determine whether or not faculty members have conversations with students about open access, and gather faculty members' ideas for a reimagined institutional repository.

Methods: To conduct this qualitative research study, two medical librarians surveyed 102 primary faculty from a school of public health about their understanding of open access in the fall of 2022. The team developed an eleven-question survey in Qualtrics and administered it via email and sent a reminder to complete the survey two weeks after the initial launch date, as well as four weeks later. The librarians approached a Dean at the school of public health who agreed to email the group of 102 primary faculty to encourage them to complete the survey. The survey had a 40% response rate, and statistical analyses were generated in Qualtrics for the closed-ended questions. All 41 survey respondents had the opportunity to opt-in to a follow-up interview, of which nine individuals agreed. Eight of the nine respondents followed through with the interviews. Two interviewees met with a librarian in person to respond to the semi-structured interview questions, and six were interviewed virtually on Zoom. In both cases, the audio from the interviews was recorded and auto-transcribed via Zoom, and the transcripts were coded using the NVivo software.

Results: 78% of survey respondents strongly agreed that there is value in open access, however, only 5% strongly agreed that their publications were already available to those who need to access them. 61% of respondents consider whether their work will be freely available when deciding where to submit a manuscript. During interviews, five of the eight interviewees believed that sharing results is a part of the scientific method, and several interviewees self-archive in a preprint server. For faculty who do not self-archive, they feared they’d receive backlash from a journal or that the pre-peer review version might contain errors.

Conclusions: This study highlights motivational factors driving faculty publishing decisions and gaps in communication about open access incentives with public health faculty at one institution. Nearly all interviewees said regular communications from library staff regarding read-and-publish deals that the university has with publishers would be extremely helpful. These authors seek information about APC waivers and discounts. We hypothesize that more conversations about open access and self-archiving might take place in certain departments over others. The medical librarians will use the results of this study as a basis for developing targeted instruction and outreach to empower authors to find information and make informed decisions about disseminating their scholarly output.

Open but Hidden: Open Access Gaps in National Science Foundation Funded Publications as Posted Online in the NSF Public Access Repository (36)

Track: Information Management

Kimberly R. Powell - Research Impact Informationist, Emory University Woodruff Health Sciences Center Library, Atlanta, Georgia

Jenny Townes - Open Access Librarian, Emory University Woodruff Library

Fred Rascoe - Scholarly Communication Librarian, Georgia Tech Library

Objectives: In August of 2022, the White House Office of Science and Technology Policy released a memo updating its 2013 guidance for making publicly funded research open and available. However, there are thousands of publications that should have been made available by now via the terms of the 2013 memo. Yet, access to some of those works may remain behind paywalls. This project looks at National Science Foundation funded research conducted at two R1 Universities. Preliminary findings suggest that some publications identified as available through the NSF Public Access Repository remain only available as the copyrighted version of record behind paywalls.

Methods: The project team searched for papers published from two R1 Universities and funded by the NSF, using the NSF’s own Public Access Repository (PAR). Searches were limited to 2017-2020 where the 2013 OSTP memo was in effect, and all embargoes allowed by the policy would have expired. Records were reviewed to determine if the PAR held a copy, or if it directed users to an open version elsewhere (usually a publisher site). In cases where the PAR directed users with a link, two authors evaluated whether or not an open access version was actually available at the link provided, or if it was available openly elsewhere online. In addition to tracking the number of papers that should have been available but weren’t as a percentage of the total, the project team also tracked and compared similarities and differences in publications produced by the two schools and compared journal/publisher information to see if any trends emerged. The project team will consider how this study can be expanded to a wider range of publication output, and how the agnostic position on copyright in the policy may leave some research closed, when it should instead be open and accessible to the public.

Results:

Conclusions:

Open Science and Wikimedia: What Medical Libraries Can Do with Wikipedia? (27)

Track: Health Equity & Global Health

Kubra Zayim Gedik - Lecturer, Bezmialem Vakif University/Vocational School of Health Services, Istanbul, Turkey

Nurgul Kilic, PhD - Librarian, Ankara University Main Library, Ankara, Turkey

Objectives: International Wikimedia organizations have much in common with libraries. Libraries and Wikimedia projects can be considered partners in the access to knowledge ecosystem. Wikipedia, the oldest and largest Wikimedia project, is a free and multilingual internet encyclopedia. Wikidata, a different project, is a platform where all data is accessible and provides access to open data sources in line with the principles of open science. This study aims to raise awareness about Wikimedia projects. Librarians came together with various user groups to define their role in the creation of high-evidence information on platforms with high access potential.

Methods: As a provider of trusted information resources, libraries are similar to Wikipedia in that they both strive to create digital infrastructure facilities. Open science efforts aim to provide access to reliable information sources for everyone. Accordingly, all types of users access health information on the Wikipedia platform with high click-through rates per day. This can lead to the rapid spread of misinformation or misinterpretation of medical facts. A group called WikiProjectMed was established in 2004 to coordinate medical content.The working group aims to improve health entries on the Wikipedia platform. The Wikidata project, which also supports open science, maintains a database that serves as a repository for data from Wikimedia projects.The Wikidata platform applies the FAIR principles. Within the scope of this study, a health literacy workshop on Wikipedia/Wikidata will be organized with Wikimedia volunteers at a university in Turkiye that provides education in the field of medicine and health sciences.The poster will present the

perspectives and impressions of medical and health sciences students on Wikimedia in line with the survey questions asked to the students at the workshop.Students will be encouraged to develop Wikipedia content together with faculty members and librarians and will be introduced to the Wikidata platform.

Results: The study will evaluate the Wikimedia experiences of students of medicine, dentistry, pharmacy, nursing, audiology, occupational therapy, physiotherapy, and other health departments in groups. Interdisciplinary groups will be formed while providing general information. During the content development phase, they will be transformed into separate groups according to the discipline. Students' knowledge of Wikimedia and open science will be determined through questionnaires administered before and after the workshop. The survey questions will be developed within the scope of open science, Wikimedia, and application examples and the awareness level of the students will be determined.

Conclusions: Medical and health sciences libraries can improve Wikimedia projects to collaborate with their users on scientific communication, open science, networking, co-learning, diversity, equality, and inclusion, and leverage Wikipedia to better serve their communities.

Potential for Partnership: Investigating Public Library Professionals' Experiences with Health Reference (49)

Track: Information Services

Gina Genova - Clinical Librarian, Kornhauser Health Sciences Library, Louisville, Kentucky

Dani LaPreze - Clinical Librarian, Kornhauser Health Sciences Library, Kentucky

Objectives: Public libraries often serve as sources of health information. Partnerships between public and academic health sciences libraries are a potential way to improve a community’s access to and understanding of quality health information, but these partnerships must be tailored to the needs and interests of both public library staff and the patrons they serve. Two clinical librarians at a four-year public university are undertaking a research project to better understand the experiences of public librarians in their state with health information with the ultimate goal of expanding their library’s outreach work with public libraries.

Methods: This study is being conducted with IRB approval by two clinical librarians at a four-year public university. Public library professionals working in the state where the investigators are located are invited via email lists and personal contacts to participate in a semi-structured interview lasting roughly thirty minutes. Interviewees need not be MLIS-degreed librarians, so long as they handle reference work as part of their job duties. Interviews can be conducted either over Zoom or in-person during a site visit by the lead investigator. They are recorded and transcribed, then inductively coded for themes. Interview questions cover several aspects of public librarians’ experience with navigating their patrons’ health and wellness questions. This includes how often they receive health-related queries; which topics they encounter most frequently; what resources they use to answer health-related questions; and what barriers, if any, they experience in assisting their patrons with health information. For the purpose of this study, interviewees are asked to consider health and wellness broadly, to encompass subjects like nutrition, exercise, and health insurance in addition to medical conditions.

Results: Twelve interviews have been conducted in urban, suburban, and rural communities, mostly within an hour’s drive of the clinical librarians’ institution. All reported working with health-related information, with frequencies varying from “seldom” to “almost daily.” None expressed any major barriers in assisting patrons with these queries. Common topics included fitness and diet, diabetes, and the logistics of accessing healthcare. Some were familiar with MedlinePlus, but most primarily used books, often due to patron preference. Misinformation related to COVID or vaccines was not mentioned by any participants, but several expressed concerns about fad diets.

Conclusions: These interviews have provided valuable insight into public librarians’ experiences with health information. Public librarians encounter health- and wellness-related questions with varying frequency, but they are comfortable addressing these requests. Additionally, they are more likely to be asked for materials or information related to healthy living or accessing health services rather than purely medical topics. There are opportunities for academic health sciences librarians to provide support to public librarians. For example, a number of participants were not familiar with MedlinePlus, and one librarian who expressed a desire for system-wide training on the resource. As such, MedlinePlus training may be one opportunity, and, since many patrons continue to prefer books, there may also be use for suggested booklists.

Racism Or Culture: Examining Trends in Health Disparities Literature Indexed for MEDLINE (29)

Track: Health Equity & Global Health

John Pell, MLIS/MSEd/AHIP - Associate Professor-Librarian, Hunter College Libraries, New York City, New York

Ishrat Jahan, n/a - Research Assistant, Hunter College

Geraldyn Gomez, n/a - Research Assistant, Hunter College

Objectives: Research on health in Asian American populations often invokes culture as an explanation for variance in health outcomes or utilization of care. Some researchers have suggested that there may be an over reliance on cultural explanations; however, they do not reference research that provides quantification of this possible over reliance in the context of research trends with other populations. Our research contributes to this discourse by quantifying the association between "Asian American" and "culture" indexing terms in the literature in MEDLINE indexed with either the "health status disparities" or "health care disparities" Medical Subject Terms.

Methods: We conducted a series of Boolean searches on MEDLINE records indexed with either the "health status disparities" or "health care disparities" Medical Subset Heading (MeSH) term. The searches were designed to test the association between the six major MeSH categories in the "population groups" family ("Hispanic or Latino", "American Native Continental Ancestry Group", "Asians", "African Americans", "Native Hawaiian or Other Pacific Islander", and "Whites") with the MeSH terms "racism" or "culture." We conducted a qualitative analysis exploring the concepts of racism and culture within the results of the "Asian Americans" subset search.

Results: We found articles indexed exclusively with the "Asians" population term were more strongly associated with the "culture" MeSH term than they were with the "racism" term when the article contained either "health status disparities" or "health care disparities" MeSH terms. This is opposite the trend observed with the articles indexed exclusively with the "African Americans" population term. Qualitative analysis of the Asian Americans subset found the "culture" term was consistent with the focus of the article. Bias toward cultural explanations for health disparities in Asian American populations seems a feature of the literature, not just an artifact of indexing.

Conclusions: Racism is under-utilized as an explanatory framework in articles about health or health care disparities and Asian populations.

Rural Health Promotion and Engagement: A Scoping Review (126)

Track: Information Services

Stefani D. Gafford - Health Sciences Informationist, U. of Arkansas for Medical Sciences Library, Little Rock, Arkansas

Lauren S. Tong - Clinical Librarian/Instructor, University of Arkansas for Medical Sciences Library, Little Rock, Arkansas

Andrea M. Hayes - Outreach Librarian, University of Arkansas for Medical Sciences, Little Rock, Arkansas

Objective: Researchers aim to identify methods used to promote health information, resources, and services to rural populations. General searches of the literature revealed great disparities in results depending on the database and search functionalities of the database used. A scoping review utilizing structured reporting and analysis will provide a more reliable overview of the existing literature.

Methods: An in-depth search strategy was devised and searching began in early-January 2023. Expert search techniques being utilized included the use of MeSH and other thesauri. Synonyms, truncation and nesting are being utilized where appropriate. Databases include: MEDLINE (including In Process & Daily Update records) via PubMed as well as CINAHL Complete and PsycINFO (both via EBSCO), ERIC and LISA via ProQuest and ProQuest Central, EMBASE, JSTOR, Scopus, Associated Press Collections Online, Consumer Health Complete, and Web of Science. The researchers are following accepted scoping review practices to analyze results for trends and themes.

Results: From the final included result set of 13, themes were coded as appropriate to the citation. The most common methods of contact were printed posters, flyers, and brochures that were placed in non-healthcare settings. These methods were each mentioned in (53.8%) and (46.2%) of the articles, respectively. Newspaper ads and postal mail were the next most utilized methods at (38.5%) and (30.8%) respectively. Radio ads and printed materials in health care settings were each mentioned (23.1%) in the articles. Kentucky was the most common state noted in the final set of articles, appearing in 4 out of 13 articles. Some articles received more than one code based on topic, if multiple methods of outreach were noted.

Conclusions: Preliminary analysis of the final set revealed limited publications on the topic. Discussions of methodologies of information dissemination were lacking. The majority of the methods utilized to reach rural communities are printed materials, and the use of mass media such as newspapers and radio. The results showed limited social media utilization. Additional research in this area is needed with greater focus on methods of health communication to rural communities.

Search Strategies for Chronic Diseases/Conditions: Initial Findings (51)

Track: Information Services

Megan Jaskowiak - Health and Social Sciences Librarian, Miami University Libraries, Oxford, Ohio

Objectives: The Centers for Disease Control and Prevention defines chronic diseases as “conditions that last 1 year or more and require ongoing medical attention or limit activities of daily living or both.” The Centers for Medicare and Medicaid Services collect beneficiary data on 21 chronic conditions. Because of the lack of comprehensive subject headings which cover all of these conditions, designing a comprehensive search strategy is difficult. The objective of this study is to investigate the search strategies from published evidence syntheses that examined populations that have chronic diseases (conditions) to see how researchers handled this dilemma.

Methods: A search was conducted using PubMed, PsychInfo, CINAHL, and Web of Science for systematic

reviews and scoping reviews published within the last five years, and whose populations were persons with chronic conditions. Reviews included in this study did not limit their just one chronic disease or condition. Extraction data includes search terms, databases searched, and inclusion/exclusion criteria for the populations.

Results: Upon acceptance of this abstract for a poster, this section will be updated with our preliminary survey results.

Conclusions: The results from this study will be used as the basis for a validated search hedge for chronic diseases (conditions).

A Space for Marketing the Library: A Scoping Review of Promotional Endeavors in Academic Health Sciences Libraries (133)

Track: Professionalism & Leadership

Stefani D. Gafford - Health Sciences Informationist, U. of Arkansas for Medical Sciences Library, Little Rock, Arkansas

Susan C. Steelman, MLIS AHIP - Head of Education & Research Services, U. of Arkansas for Medical Sciences Library, Little Rock, Arkansas

Caraline R. Annichiarico, MA MLIS - Academic Health Sciences Librarian, U. of Arkansas for Medical Sciences Library, Little Rock, Arkansas

Objectives: Researchers identified marketing strategies based on proven techniques used by academic health sciences libraries (AHSL). General searches of the literature revealed great disparities in results depending on the database and search functionalities of the database used. A scoping review utilizing structured reporting and analysis provided a more reliable overview of the existing literature.

Methods: An in-depth search strategy was devised and expert searching techniques were utilized including the use of MeSH and other thesauri. Synonyms, truncation, and nesting were utilized where appropriate. Databases included: MEDLINE (including In Process & Daily Update records) via OVID, CINAHL Complete and PsycINFO (both via EBSCO), ERIC and LISA via ProQuest and ProQuest Central, EMBASE and Web of Science. EndNote was used for citation management and de-dupping of results. The unique set was imported into Covidence for screening. Excel and Covidence were used during full text analysis. The researchers followed accepted scoping review practices to analyze results for trends and themes. A statistical analysis of the quantitative and qualitative data collected from surveys and questionnaires will reveal marketing methods within AHSLs as well as successes and barriers of implementation.

Results: Database searches resulted in 1114 records. Screening identified 55 records that met inclusion criteria. During full text analysis data points for the final unique set of 55 citations were coded and themes were identified. Results to be shared include: citations by year and by marketing method, marketing of specific services, library resource being promoted, and social media usage. Professional journal titles were also coded and listed by frequency of publications on themes and subject areas.

Conclusions: Analysis of the final set indicates an increase in publications regarding marketing efforts within AHSLs. However, discussions on social media were usually in a format specific to an individual university or as part of a guidance document with vague generalities covered. When discussed in conjunction with specific projects, details on the publicity aspects were often skimmed over with little details provided. Specifics on marketing strategies were ambiguous. Additional research in this area is needed with greater attention to detailed practices in academic health sciences libraries’ publicity and marketing endeavors.

Stronger Together: Collaborating on Collection Development with Subject Niche Liaisons Librarians (48)

Track: Information Management

Rachel Whitney, AHIP - Research & Education Informationist, MUSC Libraries, Charleston, South Carolina

Irene M. Lubker, AHIP - Medical University of South Carolina, Charleston, South Carolina

Jean Gudenas, AHIP - Director of Information Resources and Collection Services, Medical University of South Carolina, Charleston

Objectives: A strong collection in a subject niche that supports the research and educational needs of your users can be complicated. Whether you are the liaison librarian or just working with the subject, it is important to know the current collection so that you can anticipate the needs of your users. In 2017, after doing an initial broad collections assessment, the Director of Information Resources and Collections Services (DIRCS) identified subject areas for additional development. The objective was to see how collaborating with liaison librarians during the collection development process affected the resources within the areas of pharmacy and dental medicine.

Methods: A weighted binary classification grid was created which identified the core electronic collection first for pharmacy, then later for dental medicine. Utilizing a collaborative approach, the Director of Information Resources and Collections Services partnered with the liaison librarians for pharmacy and dental medicine. Information was shared about potential resources requested by faculty to complement the core collection. Librarians conducted a search of the collection development literature in several databases (PubMed, Academic Search Complete, CINAHL, PsychInfo) to find articles describing best practices for niche areas. Citations were collected using EndNote and uploaded into Covidence for screening. COUNTER usage statistics of pharmacy and dental medicine resources were collected and analyzed. Additionally, Cost Per Use (CPU) data for both collections is being analyzed.

Results: Results are forthcoming based on completion of data analysis.

Conclusions: Preliminary data analysis indicates that collaborating with liaison librarians for resources that were identified as Core Collections within pharmacy and dental medicine subjects has initially shown a better Cost Per Use (CPU) than when compared with resources that were identified and obtained, but not promoted through collaboration.

Trends in Biomedical and Scientific Grants Peer Review - What Librarians Need to Know (55)

Track: Information Services

Tiffani R. Conner, AHIP - Proposal Manager, Business Development, Tampa, Florida

Laura A. Murray, AHIP - Graduate Medical Education Librarian, University of South Florida, Morsani College of Medicine, USF Health Libraries, Tampa, Florida

Objectives: The objective of this rapid scoping review was to identify domestic and international artifacts concerning peer review of funding proposals to uncover future trends for evaluation of future funding proposals. To be included the resources had to discuss peer review of funding opportunities rather than journal articles. The search focused on dates between 2019-2022 to build upon previous completed work that was published in 2018. Included artifacts were: 1) significant materials identified via citations back to 2017, 2) articles concerning journal peer review if broader than just journals, and 3) government grey

literature such as blog posts, newsletters, manuals, etc.

Methods: We utilized a rapid scoping review, following the protocol developed by Tricco et al (2017) to discover the literature (both published and grey) pertaining to peer review. To identify artifacts, we conducted searches in electronic databases including Gale Databases, PubMed, Google Scholar, Scopus; the Internet; government/funding agency’s websites; and professional organizations’ websites and journals. To maintain an international perspective, we included US agencies (National Science Foundation, Code of Federal Regulations, Departments of Energy, Education, Justice, and Veteran’s Affairs), International agencies (Canada, United Kingdom, Netherlands, and Australia), and Professional organizations (Embassy of Good Science, Center for Open Science, Foster Open Science (EU), European Network of Research Integrity Offices (ENRIO), African Research Integrity Network), and the International Congress on Peer Review and Scientific Publication. The protocol followed included an initial search result saved and housed in Mendeley. Upon completion of initial searches, a down-select process was conducted in two stages: 1) review of titles and abstracts, and 2) full-text articles. Any discrepancies were handled through consensus discussions. Qualitative coding was used to group articles and determine major themes from the literature.

Results: Of 3,812 identified candidates, 90 items identified six peer review trends and six literature themes. Six trends being pursued by funders or discussed in the literature were identified including tools to make reviewing easier, recruiting subject matter experts (SME), using machine learning to assist with peer review, outreach to targeted SMEs, COI and research integrity, and training/ support for reviewers. The themes focused on the topics and types of artifacts. The six themes uncovered were information, instruction, alternatives, statements, elements of bias, and changes to criteria or program.

Conclusions: One of the key motivators and improvements for peer review are tools that make the process easier and streamlined. Identifying and securing qualified SMEs was noted as a critically difficult issue facing and one top suggestion was creating a more diverse group of reviewers. Developing algorithms and utilizing machine learning to address grants peer review was a relatively frequent topic in the literature. Conflict of interest (COI) confirmation and prevention is not new to peer review but was discussed with regularity in the literature. Required and targeted training was a dominant issue. Specific training topics included general training for Minority Serving Institutions (MSI) scientists, properly reviewing grants, effective and ineffective interventions, scoring criteria, and facilitation.

Using Curriculum Mapping as a Tool to Align the Information Literacy and Evidence-Based Research Learning Outcomes in Nursing Curriculum (108)

Track: Education

Jane Wu - Digital & Web Services Librarian, Courtright Memorial Library, Westerville, Ohio

Sara Ross, MLIS Student - MLIS Intern, Kent State University

Objectives: This research investigated how to use curriculum mapping as a curriculum-integration tool to identify the components of information literacy (IL) and evidence-based practice (EBP) in the nursing curriculum for the future systematic incorporation of information literacy components in teaching EBP. The primary objective is to strengthen information literacy and evidence-based research competency. Using curriculum mapping as a tool helps discover courses and specific places within the curriculum where IL and EBP content could be integrated within the program sequence. It can also help to provide an opportunity to explore valid and reliable assessment tools to measure student IL competencies.

Methods: The ACRL information literacy framework in Higher Education, Information Literacy Competency Standards for Nursing, EBP Competencies, and the EBP research model was used to provide structure to create a map and crosswalk of information literacy and EBP student learning outcomes. We obtained 59

copies of the nursing undergraduate and graduate course syllabi to perform content analysis for each course. Content analysis was performed on each syllabus to identify existing or potential learning outcomes and assignments related to EBP and IL. We then developed a codebook to guide the identification and coding of competency categories. We used this codebook to independently code the types of the included IL and EBP competencies in fifty-nine syllabi from both undergraduate and graduate nursing programs. A survey and follow-up interview also helped to gain information on EBP teaching and curriculum learning support needs from the library. The curriculum map well represented the components of information literacy and evidence-based research competencies. It thus provided an opportunity to seamlessly align the assessment of IL learning outcomes with the EBP learning outcomes in the nursing curriculum. It also built up the basis for the future creation of valid and reliable assessment tools to measure IL and EBP competencies.

Results: ACRL Framework, Standard & EBP are well represented in Syllabi from low 69.5% to high 93.2%. It helps us to understand where and to what extent the nursing curriculum addresses specific student learning outcomes and identifies gaps between and opportunities for enhancing the scholarly expectations of undergraduate and graduate EBP education and the building blocks of information literacy (IL) education. The curriculum map will offer fully embedded librarian–faculty partnership opportunities to evaluate courses for IL and EBP learning outcomes together. The research is a start to establish a baseline of library engagement in the nursing curriculum.

Conclusions: The representation of the competencies of the ACRL Framework for Information Literacy and information literacy standards for nursing aligns with evidence-based research competencies. Yet many of the competencies could only be taught and assessed by the nursing faculty. Collaborating between the faculty member and the librarian to improve the SLO assessment requires innovative planning and a good curriculum roadmap. It would be beneficial to know if students can demonstrate a higher level of information literacy competency at the completion of a course and degree when collaborating with the curriculum faculty in the assessment. Successful collaboration necessitated understanding each other's discipline-specific approach to information and developing a shared language.

Using Retrospective Evidence to Evaluate Publication Hedge Impact in Pathology-Related Guideline Development Projects (131)

Track: Innovation & Research Practice

Carol F. Colasacco, AHIP - Medical Librarian Specialist, College of American Pathologists, Northfield, Illinois

Marisol Hernandez - Medical Librarian Specialist, College of American Pathologists, Northfield, Illinois

Objectives: Systematic reviews play a critical role in clinical practice guideline development, and published evidence has increased dramatically in recent years. We routinely exclude low-level evidence (e.g., letters, editorials, commentaries, case reports) in literature searches for College of American Pathologists (CAP)-developed guidelines and may apply a more comprehensive publication hedge when a broad scope requires additional focus. A retrospective review was designed to determine whether this strategy would exclude articles that had been included as evidence in previously published CAP guidelines. Results will be used to evaluate search methods and strategize efforts to balance precision and recall without negatively impacting guideline quality.

Methods: A total of 22 different publication hedges were evaluated; four for guidelines, seven for systematic reviews/meta-analyses (SRs/MAs), seven for randomized controlled trials (RCTs), and four for observational studies. Four hedges had been developed internally; the remaining were selected from external sources. Control sets of papers were generated for each publication category from articles included as evidence in

prior CAP-developed guidelines. Each hedge was run in Ovid MEDLINE and checked with its control set. Results were documented, and a closer examination of control articles not captured was completed, including a review of indexing terms and keywords. All publication hedges were carefully reviewed and edited, and a comprehensive publication hedge was constructed that covered all four publication types. This hedge was evaluated against the combined control set to ensure that it performed at least as well as the highest-performing hedge in each publication category. All control articles that had been missed were again examined for additional controlled vocabulary and keywords. Further investigation of the impact of this comprehensive publication hedge on the literature search results of two current guideline projects is ongoing.

Results: In initial tests, the CAP hedge for guidelines captured 46/47 control articles (97.9%), greater than or equal to the other hedges evaluated. For SRs/MAs, the CAP hedge captured 60/64 control articles (93.8%), one fewer than the highest-performing hedge. For RCTs, the CAP hedge captured 30/34 control articles (88.2%), more than others evaluated. For observational studies, the CAP hedge performed better than others but captured only 77/94 of the control articles (81.9%). After edits, the comprehensive hedge containing all publication types captured 228/239 control articles (95.4%). When the new hedge was applied to a current guideline’s literature search, the total hits increased by nearly 23%. Further investigation is ongoing.

Conclusions: Publication hedges provide an opportunity to increase precision and reduce recall, but not all potentially relevant articles are appropriately indexed. Keywords help to counter this challenge, but identifying every relevant article may not be possible when using a publication hedge. Reviewing a guideline project’s scope and inclusion/exclusion criteria, estimating the length of time that will be required to complete a guideline project, and considering how best to balance the precision and recall of a search strategy may all influence the decision of whether to include a publication hedge. Given the evolving pace of evidence-based medicine, approaches to systematic review literature searching, and updates in medical terminology, guideline developers must appreciate and balance myriad factors when establishing guidance documents.

Using a Semi-Automated Approach to Update Clinical Genomics Evidence Summaries (13)

Track: Clinical Support

Mallory N. Blasingame - Information Scientist and Assistant Director for Evidence Provision, Center for Knowledge Management, Vanderbilt University Medical Center, Nashville, Tennessee

Jing Su - Senior Information Scientist, Center for Knowledge Management, Vanderbilt University Medical Center, NASHVILLE, Tennessee

Jerry Zhao - Senior Application Developer, Center for Knowledge Management, Vanderbilt University Medical Center, Nashville, Tennessee

John D. Clark - Senior Application Developer, Center for Knowledge Management, Vanderbilt University Medical Center, Nashville, Tennessee

Taneya Y. Koonce - Deputy Director, Center for Knowledge Management, Vanderbilt University Medical Center, Nashville, Tennessee

Nunzia B. Giuse, FMLA - Vice President for Knowledge Management | Professor Biomedical Informatics, Medicine, Center for Knowledge Management, Vanderbilt University Medical Center

Objectives: Recognizing the rapid expansion of genetic content knowledge due to the rate of ongoing discovery, our team developed a semi-automated approach to aid with the scalability of maintaining its supporting evidence. An analysis of the manual work process identified two distinct and complementary opportunities for automation: 1) a rules-based algorithm, which ranks citations based on pre-defined

metadata indicating an article’s likelihood of relevance, and 2) an open-source natural language processing (NLP) tool, ASReview, which further sorts citations using active learning. Our team conducted a prospective analysis to assess this semi-automated approach for identifying the most relevant citations for evidence updates.

Methods: Through a series of retrospective evaluations, the team worked with developers to assess and refine our established process. The rules-based algorithm initially ranks citations based on how "critical factors" necessary for answering the question are matched in each record. The top (maximum of 20%) and bottom (maximum of 5%) ranked articles are imported into ASReview as "prior knowledge" candidates and screened to "train the model"; then the remaining articles are imported and screened in order of relevance determined by the active learning model. Although the evaluations initially conducted greatly improved both the algorithm and metadata design, they still left us uncertain on whether all critical evidence was included. Thus, we implemented a prospective assessment comparing the effectiveness of replacing our current manual approach with a semi-automated one. Information scientists manually reviewed all new citations for 10 topics and conducted semi-automated review using pre-defined stopping rules on the same citation sets. Citations were screened for relevance and inclusion in the evidence summary. We analyzed these data, including number of citations reviewed and Relevant References Found (RRF), to assess time savings, effectiveness, and the possibility of any evidence loss.

Results: A total of 1,837 citations were retrieved from 19 search strategies underlying 10 topics. Of 54 articles manually selected for inclusion, three (6%) were missed by semi-automated review. One was a duplicate screened in another search strategy, one supported existing knowledge, and one added new non-critical knowledge. Semi-automated screening reduced the average number of articles screened per topic from 184 to 88. The average percentage of Relevant References Found (RRF) per topic by ASReview was 61% for the top-ranked articles included in the “prior knowledge” candidate set, and 55% for the next 10% of ranked references.

Conclusions: The prospective analysis demonstrated the ability of the current iteration of our semi-automated review process to identify many of the most relevant citations, guided by prior knowledge provided by information scientists with deep familiarity with each topic. A positive impact on time needed for evidence summary updates was also shown, as semi-automated review using stopping rules reduced by half the average number of citations screened. These results also highlight opportunities for further refinement to bring us closer to an optimal result. Additional studies are needed to confirm the ability of what we believe is a novel approach to address the complex problem of scalability for ongoing maintenance of evidence required by the rapidly advancing field of precision medicine.

Visual Exploration of Literature (132)

Track: Innovation & Research Practice

Chaeyeon Kim - Digital Initiatives Archivist, Upstate Medical University / Health Sciences Library / Archives and Special Collections

Objectives: This poster presents an overview of bibliometric analysis and visualization by exploring and analyzing bibliographic data of 22,638 review articles on Coronaviruses, COVID-19, and its associated research. Its application in library services focusing on bibliometrics and research impact is relatively new and underdeveloped. This poster will be a useful resource for gaining insights on science mapping techniques and procedures for performing research using bibliometric analysis to present emerging trends of a research topic and obtain insights into the development of the research domain.

Methods: After consulting the literature, a search term (‘COVID-19’) was identified to find review articles (n=22,638) to warrant bibliometric analysis. Bibliographic data that include titles and keywords were collected from the Web of Science in the TXT and XLS format. After data cleaning, co-authorship and co-

occurrence analysis was conducted using VOSviewer to map journal networks of the scientific literature as well as maps of frequently occurring terms in the dataset to summarize the bibliometric structure of scientific literature. Finally, a summary and visualizations were curated to discuss the findings along with their implications.

Results: A keyword analysis is conducted using VOSviewer to present the most frequently used keywords in the title and abstract of global research so far published on coronavirus disease. The terms ‘sars-cov-2’, ‘vaccine’, ‘inflammation’, ‘pandemic’, ‘mortality’, and ‘mental health’ appear to be the most frequently used terms in the title and abstracts of review articles on COVID-19. Secondly, the co-authorship network of countries showed that 134 of 183 countries have participated in publishing at least five review articles.

Conclusions: Due to the usefulness of bibliometric software and databases that ease the acquisition and assessment of large volumes of bibliographic data in research, the bibliometric methodology has gained popularity in recent times. Nonetheless, the science mapping technique is an effective method of summarizing large bibliographic datasets, it is not without limitations. The bibliographic data from databases are not produced exclusively for bibliometric analysis and can contain errors. To reduce errors, researchers should clean the bibliographic data which includes removing duplicates and erroneous entries.

POSTERS: PROGRAM DESCRIPTION ABSTRACTS

Although poster numbers are included, content in this section is sorted by title in alphabetical order.

Analyzing Our Systematic Review Service Data: Do We Collect What Matters? (56)

Track: Information Management

Johanna Goldberg, MSLIS - Research Informationist, Memorial Sloan Kettering Cancer Center Library, New York, New York

Kendra Godwin, MLIS - Research Informationist, Memorial Sloan Kettering Cancer Center Library, New York, New York

Celine Soudant - Research Informationist, Memorial Sloan Kettering Cancer Center Library, New York, New York

Lindsay M. Boyce, MLIS - Research Informationist, Memorial Sloan Kettering Cancer Center Library, New York, New York

Robin O'Hanlon - Associate Librarian, User Services, Memorial Sloan Kettering Cancer Center Library, Astoria, New York

Background: Our hospital library began a formal systematic review (SR) service in 2010, wherein staff provide crucial guidance and support to SR teams [1]. Since then, the need for accuracy and standardization of SR project tracking data has become clear both in our service and in the larger SR field. In one outside study of 326 SR protocols registered in PROSPERO in 2011–2012, 26% had not been published by 2017. Time to publication was a median 16.3 months/495.79 days [2]. One aim of our data collection project is to track our institution’s time from request to publication, and to identify factors that may lead researchers to publish or cancel the project. Collecting additional data may result in service improvements.

Description: Library staff changes and requests for search updates motivated our data collection adjustments. By 2019, all original SR service staff members had left the institution. At that time, data collection included the requestor name and department, topic, date request received, date search needed, assigned staff member, time spent on different tasks, search date, and publication date. In 2020, we added additional information to our tracking spreadsheet going forward, including the names of known SR team members, the SR requestor’s job title, email address, and whether the project was cancelled, with the reason for cancellation listed if known. We also added tabs for work done on SRs requested in previous years. Data was located on multiple spreadsheets and drives, with some early SRs not found in shared tracking documents. In 2022, spreadsheets from 2018 and onwards were consolidated and moved to Microsoft Teams, where the data became centralized and accessible on the cloud.

Conclusion: Staff marked 32 SRs requested 2019–2022 as cancelled, with 25 records listing reasons for cancellation. See Figure 2 for a breakdown of cancellation reasons. Eighteen SRs requested 2019–2022 were published, taking a median 524.5 days, with a range of 154–1,071 days. In the future, we will collect information on protocol registration, number of citations to screen, number of screeners, and stage at cancellation. We will also list contact information for multiple SR team members. Finally, we will assign a staff member to check the consistency of the data tracked. The additional data will inform education and intake efforts.

Works Cited:

1. Jewell S, Gibson DS. Nine steps to a systematic review service. *MLA News*. 2014 Feb;54(2):11.
2. Tsujimoto H, Tsujimoto Y, Kataoka Y. Unpublished systematic reviews and financial support: a meta-epidemiological study. *BMC Res Notes*. 2017 Dec 6;10(1):703. DOI: http://dx.doi.org/10.1186/s13104-017-3043-5.

The Benefits of Being in “Print”: Getting Involved with Practical Academic Librarianship (134)

Track: Professionalism & Leadership

Stacey Greenwell - Coordinator of Educational Services, University of Kentucky Libraries, Lexington, Kentucky

Jennifer A. Bartlett - Oral History Librarian, University of Kentucky, Lexington, Kentucky

Background: *Practical Academic Librarianship* is one of two peer-reviewed journals published under the auspices of the Special Libraries Association Academic & Education Community, and has a published history of over 10 years. After a brief hiatus, the two new co-editors have revitalized the journal and are in the fourth year of building out the journal’s focus, audience, and readership. Publishing in a journal like PAL is a key ingredient in bringing important and emerging projects and research to the attention of the professional community and can be a useful career boost. This poster aims to introduce new and established library professionals to PAL and discuss the advantages of getting involved in library publishing.

Description: Interested in sharing your research? Want to get more involved in your professional community? An important aspect of any information professional’s career portfolio is research and publication, but finding a good fit for your work can be a challenge. One of two peer-reviewed journals published by the SLA Academic & Education Community, Practical Academic Librarianship (PAL) is a longstanding venue for writing from new and established library professionals in a variety of library and information institutional settings, and SLA membership is not required for publication. This poster will describe the focus of the journal, showcase previous content, and outline not only the process for submission, but also other ways to help with the journal’s publication process.

Conclusion: The submission process for the journal is open and ongoing, and the co-editors will consider expressions of interest to review at any time. We hope this poster will describe the benefits of publishing in library and information science, bring more exposure to the journal itself, and encourage conference attendees to consider getting involved with the journal and publishing in some way.

Bite-Sized Appreciative Inquiry for Strategic Planning in Small Libraries (62)

Track: Professionalism & Leadership

Carrie D. Adams, MA, LIS; MA, IDT - Program Director, University of Florida Health Science Center Libraries, Borland Library, Jacksonville

Background: Health Science and Special Libraries often have smaller staffing numbers, making it difficult or impossible to conduct long strategic planning sessions, as this may take away from frontline services. The library has this issue, with a team consisting of four members in total. However, the most recent strategic plan was extremely outdated and many years old, and the new program director determined it needed to be updated. In order to avoid affecting library services, it was decided to apply an innovative method of several shorter sessions using the appreciative inquiry framework during team meetings.

Description: Appreciative Inquiry is a framework for strategic planning that embraces the use of positive determinants as part of the planning process, including strengths and past successes. As with other forms of strategic planning, it can take several day-long sessions to complete. Because of the low staff numbers at the library, the framework was divided instead into many shorter sessions, to be presented during regularly scheduled team meetings. Each session is designed to be completed in 20-30 minutes and uses the appreciative inquiry framework. The program director determined how to distribute each session in order to provide continuity in the appreciative inquiry process and avoid interrupting productive conversations related to planning. The process is designed to take several months, using monthly inquiry and planning sessions. Using team meetings for this time allows for limited interruption to workflows and provides a comfortable environment for team sharing and discussions.

Conclusion: This project is not fully completed, but results will be available at MLA 2023, and will include an example of a completed strategic plan based on the methods described above as well as qualitative data of staff and faculty impressions and responses to the bite-size method of implementing strategic planning and the use of appreciative inquiry to do so. Other outcomes will be a discussion of the possible use of appreciative inquiry as one tool to help improve internal culture.

Books? In a Library?! (33)

Track: Information Services

Brandon Patterson - Technology Engagement Librarian, Eccles Health Sciences Library, Salt Lake City, Utah

Christy Jarvis, MLIS, AHIP - Associate Director, Scholarly Communications, Eccles Health Sciences Library, Salt Lake City, Utah

Donna Baluchi - Equity, Diversity, & Inclusion Librarian, Eccles Health Sciences Library, Salt Lake City, Utah

Background: In 2013, EHSL de-accessioned 90% of its existing print collection in order to accommodate an anticipated incoming campus partner. This action freed up 16,000 square feet and eliminated 24,000 linear feet of shelving. When the space remained unclaimed by our campus associates, the library had the opportunity to explore alternative use case scenarios that would address outstanding patron requests, including the creation of a casual reading collection. In November 2020, a large bookshelf that used to sit empty began to be filled with books, inviting passersby to browse and select a book that might provide them with a mental break, help them learn a new skill, or complement their studies in some way.

Description: The Browsing Collection encourages casual reading by students, staff, and faculty from five schools and colleges in the health sciences. Over 380 titles are centered on popular materials that include self-help books, novels, magazines, cookbooks, and popular nonfiction, among others. A committee of librarians makes suggestions to add to the collection on a quarterly basis based on recommendations from stakeholders and partnerships with offices in the health sciences. To highlight the collection, we’ve partnered with the medical humanities program on campus, had exhibits on specific themes, and have asked key faculty and deans to have select titles they’d recommend others to read (with a nice note transcribed inside the book about why they enjoyed it). The space holding the collection also provides a physical display space and materials to engage passersby with social justice topics, from Hispanic Heritage Month to Trauma-informed Healthcare to indigenous health practices.

Conclusion: The collection has created an atmosphere expected of a library. They line a shelf close to our entrance and are enjoyed and used by our patrons. The shelves with books also absorb sound for students wanting a quiet area for study. To date, the collection has 383 titles costing approximately $6,000. The number of requests/holds and loan transactions from our collection is 635 titles. These “causal reading’ titles

are the most frequently requested and borrowed items in the library’s print collection.

Building a Architecture for Pre-Publication Data in an Institutional Data Catalog (113)

Track: Information Management

Ummea Urmi - Data Catalog Coordinator, NYU Health Sciences Library, New York, New York

Ian Lamb - Senior Solutions Developer, NYU Health Sciences Library, New York, New York

Nicole Contaxis, MLIS, MA - Data Librarian and Lead of Data Discovery, NYU Health Sciences Library, New York, New York

Background: A medical library developed a data catalog, where descriptions of research data are available to the public, even if a user needs to apply in order to access certain protected datasets. Previously, very few researchers chose to share pre-publication data. There were concerns about descriptions of pre-publication data being made broadly available to individuals outside of the institution. Therefore, to address these concerns and encourage pre-publication data sharing within the institution, the data catalog was updated to create an ‘institution only’ view, where researchers could share descriptions of their pre-publication data with only their colleagues at the institution.

Description: To create this new functionality, the library collaborated with the institution’s Information Technology (IT) department as well as the Genomic Technology Center (GTC), a shared scientific facility interested in assisting researchers share pre-publication genomic data. The IT team performed user interviews with researchers at the institution, focusing on the information they need to locate data, what prevents them from sharing their own data, and what tools they would want in a data sharing platform. Using this information, project personnel developed new functionality for the catalog, including the ‘institution only’ view for the sharing of pre-publication data. Additionally, the team created workflows for the request, approval, and transfer of pre-publication data. Outreach about this new functionality and to further encourage pre-publication data sharing within the institution is ongoing.

Conclusion: The development of the new functionality is complete. Descriptions of pre-publication datasets are now made available to institutional faculty and staff only, and access to the data is only granted with the principal investigator's consent. While the new functionality has only been available for one month, we will measure the following pieces of information to assess the success of our program: the number of pre-publication datasets described in the catalog, the number of pre-publication datasets requested, and the number of pre-publication datasets successfully shared. This assessment will help guide outreach efforts as the team continues to encourage pre-publication data sharing within the institution.

Calling Dr. Google (105)

Track: Education

Joanna Anderson - Medical Librarian, Dunbar Library/Boonshoft School of Medicine, Springfield, Ohio

Background: LCME standards require that medical students learn to be self-directed, life-long learners, this also includes using critical judgement and problem-solving skills (Standards 6.3 & 7.4). These skills are crucial to understanding the Five A’s (Assess; Ask, Acquire; Appraise; Apply) of evidence based medicine (EBM). How can medical librarians help medical students master the process of EBM? The author worked with Undergraduate Medical Education to implement an innovative way to aid students in self-directed learning. The role of ”Dr. Evidence” was added as a student role in problem-based learning (PBL) cases in three systems-based modules in the Foundations curriculum (Years 1&2).

Description: Students have an orientation session with the medical librarian on search strategies. During the first integrated systems-based module focusing on cardiology, pulmonary and renal, Dr. Evidence is provided faculty-generated background questions for the first few PBL cases. By the end of the first module, students in their PBL groups generate background questions for Dr. Evidence. Dr. Evidence completes the required worksheet after the PBL cases opens and submits their answer to the medical librarian at case closure for review. Faculty facilitators give students oral feedback on their Dr. Evidence searches at case closure. A medical librarian provides written feedback on their Dr. Evidence worksheet, including the quality of the background or foreground questions, the credibility of information sources and the question answer. As part of the worksheet, students describe their search strategies.

Conclusion: The addition of a Dr. Evidence role can be incorporated by any medical school with the help of the medical librarian to expand students’ understanding of EBM.

Cutting UpToDate at a Small, Private, Health Sciences University (106)

Track: Education

Ekaterini P. Papadopoulou, AHIP - Health Science Librarian, Seattle University

Background: At a small, private health sciences university with two teaching clinics, and after 10 years of increasing usage, overreliance on UpToDate came under scrutiny. Data from the reps suggested it was being used “too much”; reports from department heads indicated it was being relied on too heavily; reports from alumni indicated that their skills were not transferable after graduation without continued access to UpToDate; budgetary restrictions resulted in the need to reduce the multiple point-of-care options available through the library. In order to address these three issues, we embarked on a multi-step process to ease the reliance on UpToDate before ending the institutional subscription.

Description: From Fall 2019 to June 2022, the library instigated a series of steps to move away from extreme reliance on UpToDate as a campus and clinic resource. This included an assessment of the LMS to identify use of embedded content, outreach to stake-holders, an awareness campaign, an embedded curricular component, a stepped technological pull-back, and the promotion of alternative/replacement resources.

Conclusion: In June of 2022, the subscription to UpToDate expired. Initial assessment of library satisfaction surveys in the Fall of 2022 indicated minimal impact on student satisfaction with library services. Additional data is awaited from faculty and alumni surveys to assess for any trending changes in satisfaction with library resources since the end of the UpToDate subscription. From June to October 2022, fewer than 10 questions about continued access to UpToDate were logged, indicating a successful awareness campaign and transition to alternative resources.

Canines and Cookies: Outcomes and Lessons Learned from Planning a Grand Opening Party for Students at Our New Health Sciences Library Location (120)

Track: Information Services

Caitlin Maloy, AHIP - School of Nursing and Research Services Librarian, University of Washington Health Sciences Library, Everett, Washington

Leah DeSantis - Public Health and Research Services Librarian, University of Washington Health Sciences Library, Seattle, Washington

Background: Our health sciences research library serves multiple health sciences schools and hospitals. We recently opened a second library location to provide a space for interprofessional education and collaboration. Our task force of librarians and paraprofessionals planned a grand opening celebration to engage students and stakeholders in this new library. Our goals included outreach to new students and potential users while connecting them to resources and services. This was our first in-person library event for students since COVID closures in 2020, so we had a strong incentive to succeed. Our planning resulted in a popular event with a large student and faculty turnout, a greater awareness of the library and library services, and it laid the groundwork to host future events.

Description: Our grand opening task force was charged with planning and hosting a celebration of our new auxiliary library space. We began planning in April 2022 with an opening date that September. With our short timeline and strict budget, we divided tasks among our committee members and held frequent meetings online to share status updates. We coordinated our print and virtual marketing campaign with the annual campus-wide student welcome week to reach a larger audience. We chose attractions that students would enjoy most by using input from student employees, offered catering with cookies, custom-ordered giveaways and decorations for students, and hired a photographer and the university’s live husky mascot for photos. Attendees had their photos taken with the dog both by a professional photographer and on their personal devices which gave the library additional exposure on social media. We invited the local representatives of several library vendors to attend and provide informational sessions about their products. The health sciences librarians also attended to answer questions from students and faculty about the library space and services available to users. New students especially were interested in what the library offers, and some instructors expressed an interest in holding library informational sessions in classes.

Conclusion: The library’s grand opening party on September 28th was a huge success with over 350 attendees who enjoyed cookies and giveaways, which were among the most popular items. The university mascot had the biggest appeal with long lines of attendees waiting for photos with the husky. After the grand opening’s success, the library hosted a National First-Generation College Celebration that November. Using student feedback and our observations from the grand opening helped us plan another popular event with at least 75 students and four health sciences schools attending. Understanding what students want and enjoy as they return to in-person learning will help us create more impactful events and marketing strategies for future engagements, ultimately bringing more people to the libraries.

Chart Your Path to CHIS (19)

Track: Health Equity & Global Health

Jamia J. Williams - Consumer Health Program Specialist, Eccles Health Sciences Library, University of Utah/NNLM NTO, Rochester, New York

Jessi Van Der Volgen, AHIP - Associate Director NNLM NTO, Eccles Health Sciences Library, University of Utah, Salt Lake City, Utah

Molly Knapp, AHIP - Training Development Manager, Eccles Health Sciences Library, University of Utah / NNLM NTO, Houston, Texas

Background: We created “Chart Your Path to CHIS” to increase awareness of NNLM’s free Consumer Health Information Specialization (CHIS) classes and CHIS application fee sponsorship program.

Description: Our aims were to clarify the steps in obtaining the specialization and encourage more library staff to complete the requirements for the specialization. We designed two promotional items: an interactive web-based infographic and a one-page printable flyer. We shared promotional materials across multiple organizations and media types through a trackable URL.

Conclusion: Over a three-month period, we tracked clicks, enrollment in advertised classes over the preceding three months, and requests for CHIS application fee sponsorship. These efforts resulted in a 213% increase in NNLM class enrollments and 40 public library workers earning their CHIS.

Creating a Better Doctor: Introducing the Medical Humanities to an HBCU Health Sciences Community (14)

Track: Education

Jeremy Gunnoe - Assistant Director and Medical Librarian, Louis Stokes Health Sciences Library - Howard University, Washington, District of Columbia

Background: In the 19th century, Edward Livingston Trudeau said that medical practitioners need, “To cure sometimes, to relieve often, to comfort always.” This quote is often used to recommend the use of the humanities in medical education. The medical humanities is a pedagogy that integrates history, literature, and the arts with medical education. Its implementation promotes the understanding of the patient experience among medical practitioners to build empathy, and become better observers, communicators, and team members. Both the AAMC and the AMA are committed to supporting the integration of humanities into health sciences programs and many universities have already added this material to their curricula. However, there is currently little evidence of the use of the medical humanities at HBCU’s.

Description: I will begin to introduce the medical humanities to the patrons of our Health Sciences Library through a limited print library collection and display area and will start to promote this topic and the library resources available. Outreach will include book clubs, faculty development presentations, fact sheets, and suggested reading lists throughout the spring 2023 semester. This topic has been introduced by adding a monthly themed digital medical humanities “bookshelf” to our website beginning mid-2022. These web pages, with links to digital materials, have received over 250 visits since July 2022. To continue this process, the library has identified and purchased a small selection of narrative and graphic medicine print materials to add to the library collection. As an HBCU, initial choices consider the experiences and backgrounds of our university community, commitment to health equity, and combating disparities in healthcare. Through both quantitative and qualitative analysis and reporting at the end of the trial period, the information and data obtained from this project will be analyzed to determine if the topic should be introduced to the various curricula committees and to determine if a permanent collection development strategy for this material should be put into place at our library.

Conclusion: The Medical Humanities LibGuide (with themed digital bookshelves) was the sixth most viewed guide this past year out of 50+ guides. Additionally, 40+ print books were purchased for a new Medical Humanities collection and have been extremely popular with patrons. Unfortunately, due to a delay in shipment, the book club has been moved to Fall 2023 and a presentation scheduled to the Health Sciences colleges will be done this summer. While working on this project Howard and Georgetown University announced plans to launch a joint Medical Humanities program and has asked the library to be involved and to assist with collection development, which offers us an ongoing opportunity to continue to share information on this topic with our campus community.

Creating a Community of Medical and Public Librarians through a Web-Based Collaborative Book Selection System (16)

Track: Education

Yumi Yamashita - Librarian / Ph.D student, Kyoto Prefectural Library / Department of Humanities and Social Sciences, Keio University, Kyoto, Japan

Chie Suga, MLIS - Assistant Professor, Library Science Course, Jissen Women’s University

Nozomi Ikeya - Professor, Department of Humanities and Social Sciences, Keio University

Background: Public librarians in Japan have found it difficult to select books and provide reference services while also promulgating health and medical information to the public. Librarians across library types have collaborated to address this issue, and through their efforts, lists of reliable books have been composed to reduce the burden on each librarian in the book selection process. At the same time, as the creation of a reliable book list itself is labor intensive and it is not easy to continuously update a public list, some lists have been made public, but others have been available only to members.

Description: Having reviewed past efforts that have been made on this issue, we decided to create a community of medical librarians and public librarians through the creation of a cooperative book selection system that creates an ongoing web-based knowledge community where librarians can bring books on health and medicine, introduce them, and evaluate them. Members can easily register books in the system by entering the ISBNs. They can write a description of each book and add recommendations where warranted. The Web-based Collaborative Book Selection System currently has over 30 librarian members participating and sharing a list of over 200 books. Members register their information when they have a book recommendation. The list of books contributed by members can be used by other librarians as a reference for book selection. The members also created material for exhibition such as a book list and brochures with the National Cancer Institute at libraries around the country. In addition, there is a mailing list in which members highlight various issues and exchange tips for health information services, including information about book selections and recommended events.

Conclusion: This system allows members to provide information without feeling burdened. Since only members can see the shared list, they can freely exchange information. On the other hand, if the size of the group does not increase, it will be difficult to collect information and thus to use the system as a reference for general book selection. Moreover, the small number of members may result in biased information . We are still seeking better ways to deal with these issues, in part through collaboration with the Health Information Committee of the Japan Library Association.

The Data Services Continuing Professional Education (DSCPE) Program: Creating Communities of Practice for Data Services Librarianship (30)

Track: Education

Sara N. Hoover - Metadata and Scholarly Publishing Librarian, Himmelfarb Health Sciences Library, George Washington University, Washington, District of Columbia

Background: Federal mandates such as the 2023 NIH Data Management and Sharing Policy (NOT-OD-21-013) have created a heightened need for research data management services offered by academic libraries. Due to these policies, medical librarians are likely to see an uptick in researcher questions about compliance, creating data management plans, and locating appropriate data repositories. For many librarians, the ability to fully engage with data management related questions will require additional training. In the fall of 2022, the Data Services Continuing Professional Education (DSCPE) program sought to address this need via an eight-week intensive online learning experience. The objective of this poster is to highlight the role of programs like DSCPE in creating communities of practice for data librarianship.

Description: The Data Services Continuing Professional Education (DSCPE) program was developed by members of the Harvard Medical School’s Countway Library and the Simmons University School of Library

and Information Science with the aim of addressing a known skill gap among librarians. The DSCPE pilot program was an eight-week course that ran from early October 2022 through the end of November. Early and mid-career librarians needing to learn more about the research data management ecosystem applied for the program during the summer of 2022 and the first cohort consisted of fifteen librarians from both medical and non-medical libraries around the United States. Funding for the Fall 2022 cohort tuition was provided by a gift from Elsevier. Program participants gained experience with research data management through structured instruction, independent learning, and a seventy-hour paired capstone project with a mentor site. Program activities aimed to build research data management knowledge among practicing librarians and allowed them to apply learning to a specific project. In this poster I describe the structure of the program and my experience as a participant in the first cohort. Program content addressed research data management needs arising from the 2023 NIH DMS Policy, but also created space for broader application.

Conclusion: Programs like the Data Services Continuing Professional Education (DSCPE) program create unique professional development opportunities for early and mid-career academic librarians in need of specialized training. The DSCPE pilot program offered instruction related to the collaboration skills needed to develop research data management services across an organization. Program activities such as a library director’s panel and a supported RDMLA curriculum gave students insights into the work being done by other institutions. Through the DSCPE capstone process students gained increased familiarity with research data management services outside their own organization and expanded communities of practice. While some structural elements may be further developed in future iterations, programs such as DSCPE ultimately offer a successful model for continued training for academic librarians.

Describing Basic Science Data: Updating the Metadata of an Institutional Data Catalog (115)

Track: Information Management

Ummea Urmi - Data Catalog Coordinator, NYU Health Sciences Library, New York, New York

Michelle Yee - Senior Project Coordinator, NYU Health Sciences Library, New York, New York

Ian Lamb - Senior Solutions Developer, NYU Health Sciences Library, New York, New York

Nicole Contaxis, MLIS, MA - Data Librarian and Lead of Data Discovery, NYU Health Sciences Library, New York, New York

Background: A medical library at an academic medical center developed and runs a data catalog supported by Clinical and Translational Science Awards (CTSA). It is a data discovery platform designed to help researchers locate datasets for re-use and collaboration. Previously, data in the data catalog focused on clinical and population health research, and the author was hired to improve the representation of basic science research in the data catalog. Part of these efforts included updating the data catalog metadata in order to better accommodate the needs of the basic science researchers.

Description: In order to improve data discovery of basic science datasets in the data catalog, the author investigated several metadata elements to update: subject of study and software. Each of these metadata elements are crucial for basic science research as they help describe the subject and methods of each dataset. For each of these fields, she investigated data standards and ontologies to better understand their scope and recommendations. For example, for subject of study, she reviewed the American Type Culture Collection (ATCC) and Jackson Laboratory. Additionally, she reviewed citation guides, like the Force11 Software Citation Principles, for recommendations to adapt to the software metadata field. Both subject of study and software were presented to librarians at other institutions for feedback and critique before being implemented in the institutional data catalog.

Conclusion: Changes to the subject of study and software fields have been implemented in the catalog. Previously, the subject of study only contained two fields: subject of study and species or strain. Now, it is more descriptive and includes five fields: common name, species, strain, tissue, and cell line. For software, versioning is now consistently cataloged to better describe the tools used to access, manipulate, and collect data. 87 basic science datasets were cataloged with these new metadata fields. A future survey is planned to request feedback on these metadata additions. Future plans include investigating the equipment and software metadata elements and considering how to best catalog reagents for basic science data.

Desired Future in Turbulent Times: Strategic Planning on a Dime! (64)

Track: Professionalism & Leadership

Maud Mundava - Campus Head/Assistant University Librarian, A.T. Still Memorial Library, Kirksville, Missouri

Hal S. Bright, IV, MLS/AHIP - University Library Director, A. T. Still Memorial Library, Phoenix, Arizona

Background: The ever-changing information landscape combined with the effects of COVID-19 pandemic in health sciences and higher education are increasingly causing academic libraries to constantly rethink their value and repurpose their spaces. Other challenges involve how to remain relevant and be responsive to stakeholders’ needs, while addressing staffing issues, expanding resources, and redesigning services. This poster will define what strategic planning is by highlighting how the planning process was used as a framework to address some of the issues above while aligning the library’s mission, vision, and goals to that of the institution. The poster will: 1.Describe the process that was used to create a strategic plan for an academic library within a six-month period. 2.Demonstrate how the library used an in-house talent and an inclusive employee input circle method to develop an inspiring vision while aligning the mission and goals to that of the institution. 3. Establish why strategic plan is important and how it can be used as team building exercise and boost employee morale while advocating and engaging internal and external stakeholders.

Description: This poster will define and describe the process that was used to create a strategic plan for the academic library within a six-month period. The strategy development process started with the formation of a task-force representing key library functions. The taskforce identified key priorities/ themes from the University strategic plan and used them as a guide in formulating goals and objectives of the library strategy. In addition, the taskforce carried out an environmental scan of the health sciences academic libraries, and higher education landscape to benchmark best practices. Other higher-level activities involved engaging external stakeholders for input and evaluating libraries’ performance against key performance metrics. To ensure success, the library also used in-house talent and a staff input circle method to encourage employee buy-in at every stage of the plan. The SWOT analysis and survey was used to help identify opportunities, and key focus areas. Communication, timeline, flexibility, and celebrating previous accomplishments were key to the completion of the strategy.

Conclusion: The strategic plan was completed and is now in the execution phase, monitoring progress and adjusting as needed. The process was highly collaborative, enhanced teamwork, and really energized team morale. Additionally, it has set the framework for strengthening campus-wide partnerships and continued customization and alignment of resources and services to the diverse needs of its stakeholders. The Library looks forward to the future!

Developing an Interactive Map of Regional Reproductive Health and Substance Use Treatment Resources in a Rural US Area to Advance Access, Transitions, and Continuity of Care (21)

Track: Health Equity & Global Health

Emily S. Mazure, AHIP - Clinical & Research Librarian, MAHEC, North Carolina

Joan L. Colburn - Director of Library Science, MAHEC Library, Asheville, North Carolina

Katlyn Moss, BSN, RN - Perinatal Nurse Champion, MAHEC OB/GYN Specialists

Abigail V. Earley, BS '23 - Project Manager, UNC Health Sciences at MAHEC

Ana Cabello-De la Garza, MSW, MPH - Director of Maternal and Reproductive Health, UNC Health Sciences at MAHEC

Background: In rural areas, access to healthcare is challenged by widespread reduction in healthcare services, transportation issues, travel distance, geographical barriers, and lack of reliable broadband connection. Static maps of regional prenatal care have proven the value of visually displaying services. Further assessment indicated a need for more robust data and interactive maps. Our goal was to develop methods for collecting and curating data to map the spatial distribution of reproductive health and substance use disorder resources within our 18-county region. The map and accompanying dashboard are intended to enhance access, transitions, and continuity of care at the micro, mezzo, and macro levels.

Description: A multi-disciplinary team operationalized an innovative tool to increase access to and visually highlight regional health and healthcare resources. We gathered data about regional resources including prenatal, primary care and pediatric practices, substance use disorder treatment locations, hospitals, harm reduction services, and safety net services. To build this database of resources, we pulled data from the American Medical Association, the Substance Abuse and Mental Health Services Administration, local medical organizations, Google, publicly available listings, and internal clinical referral lists. Data collected for each location included name, address, contact information, and services offered (i.e. offers telemedicine, welcomes LGBTQIA+, treats opioid use disorder). Creation of a data dictionary ensured consistent and rigorous coding during data collection. Missing data was manually searched for on the location’s websites or through direct contact. To ensure the validity and reliability of the data, continuous quality assurance was conducted. An external consultant developed the map and dashboard in ArcGIS. Stakeholders have repeatedly provided feedback. To ensure sustainability, structured review of the database will occur yearly. Opportunities for feedback and updates have also been incorporated into the map.

Conclusion: Continuous quality improvement activities have been conducted to refine the user experience, functionality, and usability of the map viewer and dashboard. Through internal focus groups, opportunities for improvement and expansion have been identified and, when possible, implemented. Future plans include gathering usage data and feedback from end users. Lessons learned and methodology developed throughout this process has been documented for adaptation and application in developing similar tools for additional regional resources.

Developing Research Support Services in an Academic Medical Library (35)

Track: Information Services

Faith McKoy-Johnson - Librarian, Medical Branch Library, The University of the West Indies Mona, Kingston 7, Jamaica

Background: Academic libraries in low- and middle-income countries (LMICs) face financial and technological barriers to providing research support in the e-research environment. Research lifecycle workflows are undergoing significant disruptions due to various factors. These include technological advancements, the data explosion, the research reproducibility crisis, unethical publishing practices and new mandates from grant donors. This poster follows the journey of a medical library’s research support program developed to build capacity to support the processes of inquiry used by faculty and postgraduate researchers. The program has added value at the different points of the research lifecycle.

Description: Traditional library services, while useful, are not enough to meet the changing needs of researchers. Librarians have therefore had to branch out into roles that include providing e-research services. Librarians in LMICs have had to use various strategies to acquire the necessary knowledge and skills to support students and faculty as they try to overcome the increasing challenges faced by the changing research environment. These include keeping abreast of trends and issues in health sciences librarianship by strategic use of social media, listservs, and conference and webinar attendance and participation. Another key strategy for librarian upskilling is listening to patrons’ queries and pain points. Librarians have had to market the library’s services through interactions with key stakeholders such as leaders and individual lecturers at the medical school and through targeted events. As a result, strategic partnerships have developed between the library and the medical school leading to collaboration for the development of more relevant library services to faculty and student researchers.

Conclusion: Medical libraries can build on traditional information literacy and reference services to provide research support services on all the points of the research lifecycle. With the importance of e-research in today’s higher education landscape, there are plans to create additional services related to e-research and new requirements for research. The following represent some developments soon: new position of Scholarly Communication Librarian, research Data Services (Current service involves awareness-building and staff training), and data repository for faculty and postgraduate researchers.

Development of an Automated Notification System for the National Institutes of Health's Public Access Policy Non-Compliance (37)

Track: Information Services

Beth Blackwood - Research & Education Librarian, Duke University Medical Center Library & Archives, North Carolina

Nathan Dunn, MLIS - Digital Projects Analyst, Information Technology

Lesley Skalla, Ph.D., MLIS - Research & Education Librarian, Research & Education

Background: Medical librarians, including those at our institution, regularly provide guidance on compliance issues related to the NIH Public Access Policy, which requires that all peer-reviewed publications supported by NIH funding be submitted to PubMed Central within ninety days of publication. In order to limit the amount of staff time dedicated to notifying author teams of non-compliant articles and to increase overall compliance, library staff built and socialized an automated notification system using only existent resources and systems.

Description: The project required two forms of development: one technical and one social. On the technical side, the automated system was built by creating a data pipeline that integrates and transforms datasets pulled from the NIH Public Access Compliance Monitor (PACM) and the university's existing scholarly profiles platform, Symplectic Elements. The data was then stored in a Microsoft Access database. The code was written in python using primarily the Pandas library and will be made available in the poster via QR

code. Microsoft’s Mail Merge was used to send dynamic emails based on the final data set. To socialize the research community to the new program, library staff members developed an outreach strategy that involved introducing the tool prior to its launch in blog posts, campus newsletters, library instructional sessions, and directed emails. The program was evaluated by measuring the policy compliance rate, pulled from PACM using log files logging the changes to the database, at regular intervals.

Conclusion: After several months of development and testing, the system launched in January 2023, notifying several hundred authors of their compliance issues. While evaluation is ongoing, the system has been well received and most author teams have been pleased with the reminder and information provided in the automated emails. The poster will outline several lessons learned, especially regarding large NIH grants where principal investigators are often far removed from the publications that their grants support.

Development of a Foundational Competencies Curriculum Framework with an Information Literacy Component for Nurses (66)

Track: Professionalism & Leadership

Aida Marissa Smith, MLIS, AHIP - Clinical Informationist/Medical Librarian, Ascension, St. Louis, Missouri

Cynthia LaFond, PhD, RN, CCRN-K - Research Scientist Director, Research and EBP, Ascension

Victoria Boyce, MSN, AHN-BC - APRN Clinical Nurse Spec-Acute, Ascension

Judy Dusek, DNP, M.Ed., APRN-CNS, CMSRN, ACNS-BC - Consultant

Jonathan Hecht, MSN APRN ACNS-BC CCRN CNRN - RN Director of Nursing, Ascension

Jennifer L. Smith, DNP, RN - RN Director of Nursing, Ascension, Florida

Elizabeth Yorks, MSN, RN - RN-Educator, Ascension

Background: This project describes a collaborative effort between two Ascension National Nurse Affinity (ANNA) groups at a large multi-state medical system, Evidence-Based Practice/Implementation Science (ANNA-EPIS) and Nursing Research (ANNA-NR), to develop a Foundational Competencies Curriculum Framework. Identification of foundational competencies was recognized as an important step to ensure the ANNA groups could reach their respective goals through professional development of their members. Information literacy was recognized as an important component of the curriculum framework from the onset for both groups.

Description: In September 2022, the Professional Development Planning Team formed with members from both ANNAs and two mentors, a nurse researcher and a clinical informationist. The meetings occurred virtually every two weeks for three months. The aim was to jointly define and develop foundational competencies for members, identifying areas of alignment for coordination. The team focused on leadership, research methods/evidence-based practice processes, and information literacy. The mentors supported the team’s work of identifying key concepts, on which to build associated learning objectives, content, and learning activities. The process included review of professional source documents for critical competencies, brainstorming, and discussion. Collectively, five key concepts pertaining to information literacy were identified as foundational: evidence-based organizational decision-making advocacy, availability of medical library resources and services, literature search strategies, information management, and appraisal & synthesis of information sources. The concepts aligned closely with elements found within ACRL’s Framework for Information Literacy for Higher Education, one of the professional source documents. The resulting information literacy objectives and content for the groups was the same, but variation was needed in the associated learning activities. The team will seek endorsement of the competencies by the respective

ANNA leadership and members, and begin implementing the professional development opportunities.

Conclusion: The ANNA-EPIS and ANNA-NR groups recognized the value of providing information literacy professional development for their member nurses by: *Including a clinical informationist as one of two mentors of the Professional Development Planning Team. *Identifying information literacy as one of the three focus areas of the ANNA Professional Development Foundational Competencies Curriculum Framework. *Acknowledging the applicability of the ACRL information literacy framework for higher education in nursing evidence-based practice/implementation science and nursing research in acute care hospital settings. Performance and evaluation measures will be used to assess the impact of the ANNA Professional Development Foundational Competencies Curriculum Framework. We anticipate that the content will be of interest and use to our nursing community outside of the ANNA groups.

Diverse Cultural Heritage Professionals Chronicling the History of the Health Sciences (68)

Track: Professionalism & Leadership

Shannon Bohle, MLIS, MA, CDS-PhD, AHIP - Owner / President, Archivopedia LLC, Lima, Ohio

Jennifer Ulrich, MLIS - Technical Services Archivist, Augustus C. Long Health Sciences Library, Columbia University Irving Medical Center, New York, New York

Stephen Greenberg, MSLS, PhD, AHIP - Retired, Silver Spring, Maryland

Background: Diversity is an important consideration in the library and archival sciences professions. Yet a gap continues to exist in the percentage of individuals who identify themselves as members of non-dominant religions, races, ethnicities, sexualities, and gender identities, or are differently abled. These include, but are not limited to individuals of African American, Native American, Alaska Native, Hispanic, Asian American, Pacific Islander, or multiheritage descent, identify as being non-heterosexual / GLBTQ status, or as having a disability.

Description: The poster, a contribution of the History of the Health Sciences Caucus, will highlight the contributions of diverse individuals who helped preserve health sciences historical collections. Examples include Naomi Broering, Su-Shan Chin, Maria Cunningham, Jensen Fisher, Chaeyeon Kim, Yolanda Retter, and Tomaro Taylor. Members of the MLA's History of the Health Sciences Caucus were invited to participate in the poster's content development prior to the annual meeting. The caucus is free to join for all MLA members.

Conclusion: The poster presents the diverse faces of individuals who served by example and, with a description next to each photo, highlights their professional activities and leadership achievements. The objective of the poster is to foster a greater culture of inclusivity for current and next-generation professional librarians and archivists who would like to preserve the history of the health sciences.

DOCLINE Spring Cleaning for Librarians (116)

Track: Information Management

Jill (Tarabula) Daby, AHIP - Regional Medical Librarian, Northern New York Regional Medical Library, Sebastian, Florida

Margot G. Malachowski, AHIP - Education and Outreach Coordinator, Network of the National Library of Medicine, Region 7, Worcester, Massachusetts

Background: DOCLINE’s success relies solely on regular upkeep of a library’s journal holdings and schedule, which must be maintained by each participating library. Failure to do so can prevent timely filling of information requests, which ultimately can have an impact on patient outcomes. In accordance with the DOCLINE Comprehensive User Manual, we recommend annual Spring Cleaning for DOCLINE librarians. This includes an annual review of the library’s profile, journal holdings and routing tables. Further, when a library closes, even temporarily as was seen during the pandemic, library staff should follow best practices to ensure document delivery services at other libraries aren’t negatively impacted by outdated library information. This poster will highlight recommended best practices for libraries using the DOCLINE ILL system.

Description: A solo librarian at the Northern New York Regional Medical Library (NNYRML) serves three hospitals (one critical access) with 400 providers and 3200 employees in a rural upstate tri-county area near the Canadian border. Document delivery is the most heavily utilized service. From January 2021-December 2022, the NNYRML submitted 519 DOCLINE requests, of which 473 (81%) were receipted, and 381 (73%) filled. However, of those 519 requests, 46 were never receipted and 92 went unfilled. What causes DOCLINE requests to go unreceipted/unfilled? According to Harrow et al., there was a 30% decrease in registered DOCLINE libraries from 2007 to 2017 (n=3166 to 2140). A 2022 report reveals there are now 1591 active DOCLINE libraries. When a library decides to terminate participation in DOCLINE, are they following the proper steps for closure? The DOCLINE Comprehensive User Manual recommends annual reviews and best practices with regard to library profile, routing tables, and journal holdings records. The manual also provides step-by-step guidance for temporary and permanent closure. This poster highlights current issues faced by participating libraries, and will provide a checklist for DOCLINE Spring Cleaning with screenshots of sample tasks to encourage proper maintenance and closure if applicable.

Conclusion: Librarians are encouraged to review their eligibility and commitment to participate in DOCLINE as stated in the Comprehensive User Manual including collection size, reciprocity and service levels. For those faced with temporary or permanent closure or cessation of DOCLINE participation, the checklist and user manual will guide appropriate actions. This DOCLINE refresher should lead to improved fill rates and turnaround times, as well as more accurate library profiles and directory records.

Exploring Physical Space Using H5P Image Hotpot: Telehealth Service Models for Libraries (18)

Track: Education

Abby Dowd - Instructional Designer, Eccles Health Sciences Library, University of Utah / NNLM NTO

Katie Pierce-Farrier - Data Science Strategist, University of North Texas Health Science Center, NNLM Region 3, Fort WOrth, Texas

George Strawley - Library Engagement Specialist, Network of the National Library of Medicine, Salt Lake City, Utah

Faith Steele, AHIP - Outreach and Education Librarian, Network of the National Library of Medicine

Background: When COVID-19 was at its peak, a need for telehealth services emerged for communities across the United States. Since then, libraries across the country have shown increased interest in providing telehealth spaces in their own communities. With this increased interest came an increased need for support in implementing these services. In response, We developed a course for an audience of public library workers about telehealth. Libraries were starting to provide physical telehealth spaces, and our learning audience wanted to know how to set up a space of their own.

Description: We utilized the H5P image hotspot in Moodle 3.9 to give librarians taking this course a detailed view of telehealth models. This activity allows them to choose how to explore the key components of a

successful telehealth space in a physical library through images and text. We created two hotspot image tours, highlighting different formats to allow librarians to visually recognize how each model may implement these components differently, and to encourage them to think critically about how these could be implemented in their own library.

Conclusion: These hotspots have been created; however, the class they were developed for has not been rolled out yet. As more and more libraries see this option for exploring physical spaces it is our hope that they begin to consider other ways hot spots could be utilized in their classes.

Forging Ahead with Publishing and Presenting: Tips and Insights from the Field (70)

Track: Professionalism & Leadership

Adela V. Justice, AHIP - Senior Librarian, MD Anderson Cancer Center, Houston, Texas

Background: It can be challenging for librarians interested in publishing and presenting to get started in these professional endeavors. There are so many decisions involved, such as where to find topics to publish or present on, plus if a topic is best presented as a conference poster, or as a paper presentation. There’s also the consideration about turning a topic into an article and getting it published, which leads to deciding on which journal to submit a manuscript. Although this author has years of experience in publishing and presenting, she decided to host a one-hour round table at her 2022 SCC/MLA Chapter meeting in order to garner others’ tips and insights on how they approach publishing and presenting.

Description: The round table agenda was organized into several sections: Where to find topics/ideas to publish/present on; Format, e.g., Should it be a conference poster or paper presentation or lightning talk, or is there enough here for a published paper, or could it even be a webinar?; Writing an article to be published and where to submit your manuscript; and Final thoughts/last takeaways. Nine participants, with diverse years of work experience as well as publishing and presenting experience, attended the round table. Each participant shared their own questions, challenges, discoveries, and successes they’d experienced on the road to publishing/presenting. Having such a mix of experience resulted in a lively and productive discussion. The discussion resulted in many valuable insights and practical tips such as good library journals to submit to, things to consider when submitting to conferences, how to find collaborators and co-authors, and much more.

Conclusion: Despite this author’s many conference presentations and journal article publications she still garnered much wisdom and ideas from the round table participants. The participants expressed this same sentiment. This robust, resulting list of tips and insights were shared with the participants’ emails after the chapter meeting ended, however the information is so valuable that this author thinks a wider sharing of it is warranted. This author doesn’t recall the topics of publishing and presenting being discussed in library school and came to it on the job; therefore, she believes there is room for librarians at all stages in their career to learn more about the creative and decision processes that go along with these professional endeavors.

Forging Collaborations: EBP and Information Literacy in the Allied Health (107)

Track: Education

Alessia Zanin-Yost, AHIP - Health Science Librarian, Bailey Library/ SRU, Slippery Rock, Pennsylvania

Marilia Antunez, AHIP - Life & Allied Health Sciences Librarian, University of Akron, Akron, Ohio

Background: Collaboration between faculty and librarian is essential to support lifelong learning and best practices after graduation. However, information literacy (IL) concepts and EBP skills are seldom taught together, deemphasizing their natural connection. Students assume IL and EBP need different skills and, in the long run, do not see how these notions connect to their future careers. The authors will illustrate how they integrated EBP and IL at their institutions in undergraduate courses in the allied health professions. The aim of the poster is to: Learn how the EBP process connects to IL. Determine the benefits of integrating IL and EBP into the curriculum. Collaborate with faculty to emphasize lifelong learning in students.

Description: The presenters discussed how the training in EBP at their institutions was disconnected from the instruction of information literacy. They planned similar instructions to determine how it could improve students learning and reinforce good research practices after graduation. The presenters used the ACRL Framework to provide best practices combining IL knowledge to execute EBP research. In the planning stage, a list of common skills was created. Collaboration with faculty and coordination of assignments were critical in ensuring success. From this experience, the presenters have collected feedback and insight. This study adds to the gap found in EBP and IL by providing practical examples of the impact that faculty-librarian collaborations can have in teaching EBP through information literacy for undergraduate education. These collaborations can extend beyond the librarian offering one library instruction session to reinforce students’ confidence in conducting research. The presenters offer practical examples of assignments developed from their partnerships with faculty, their discussions, and the academic needs of students in the health and allied health professions. The combination of practice, prior and new knowledge, and clinical applications demonstrate that EBP skills should not be taught in isolation but integrated into the context of lifelong learning.

Conclusion: The study aimed to identify the following: How information literacy notions and practices can support EBP instruction. How knowledge of information literacy in the allied health professions influences the understanding of EBP. How to collaborate with faculty to integrate EBP and information literacy in the curriculum. The information was grouped into a list and then reduced to the most impactful (from feedback received) experiences. The connection between information literacy, EBP, and practice demonstrates that undergraduate students need exposure to both information literacy and EBP early in their academic process.

Free, FAIR, and Fabulous: Five Tools for Data Management and Sharing (40)

Track: Information Management

Katie Pierce-Farrier - Data Science Strategist, University of North Texas Health Science Center, NNLM Region 3, Fort Worth, Texas

Christine Nieman - Data Management Librarian, University of Maryland, Baltimore, Network of the National Library of Medicine Region 1, Baltimore, Maryland

Sean Corning, MLIS - Education and Outreach Coordinator, Lamar Soutter Library

Elizabeth Roth - Research and Data Science Strategist, NNLM Region 2, Charleston, South Carolina

Background: With more publishers and funders requiring data management and sharing plans, it is important for librarians, researchers, and support staff to be aware of the tools available to help them skillfully manage research data. Librarians and researchers will identify freely available tools for data management, wrangling, and sharing that improve workflows in accordance with new NIH Data Management and Sharing Policy (DMSP) guidelines. Researchers can select tools to support data management throughout the research lifecycle, regardless of budgetary support or restrictions.

Description: There are a wide variety of free and paid data management tools available, but selecting an affordable, accessible tool can be a barrier to use. This poster will highlight five data tools: DMPTool, NIH Common Data Elements Repository, NLM Scrubber, OpenRefine, and Open Science Framework. These tools were selected because they are freely available, have help documentation, and each one supports different, or multiple, stages of the research lifecycle. Each tool is accompanied by a product guide handout that librarians and instructors can use to create instructional programming at their institution. We will discuss unique features, usability considerations, and potential applications of each tool in complying with the NIH DMSP and how they can be incorporated into the research workflow.

Conclusion: These five tools support open, FAIR, NIH DMSP compliant data practices across the research lifecycle. The health information community and the science community at large are moving to more open science practices and policies. Information and research professionals can use freely available tools to help them skillfully manage research data and facilitate open data practices.

From Digital Preservation to Open Educational Repository: How a Photo Digitization Project Transformed into a Library-Developed Open Educational Repository (20)

Track: Education

Bryan E. Hull - Head, Digital Publishing, Eccles Health Sciences Library, University of Utah, Salt Lake City, Utah

Carmin Smoot - Digital Publishing Coordinator, Spencer S. Eccles Health Sciences Library, University of Utah

Julia Curtis - Assistant Professor, Department of Dermatology, University of Utah, Murray, Utah

Background: DERM (Dermatology Education Resources & Modules) is a collaborative effort between a university department of dermatology and an academic health sciences library to produce a robust open educational platform for the discipline of dermatology. The project began as an effort to digitize a collection of 14,000 Kodachrome slides depicting clinical presentations of dermatological conditions taken and donated to the academic health sciences library by a former faculty member of the department. The project soon morphed into developing a custom-made platform to explore selections of the images using diagnoses, clinical features, and textbook references.

Description: The development of the project has been broken up into three phases. The first phase was the digitization of the donated Kodachrome slides into an Internet ready format. The second phase of the project included developing a metadata structure based on diagnosis and clinical features as well as an item-level description strategy to describe every image in the collection by the dermatology department's faculty and residents. Simultaneously, prototyping and user experience design was completed in preparation for the development phase. The third phase was the development phase, where the customized platform was developed in-house by the library's web programming and design team, incorporating the system and features outlined in the design phase.

Conclusion: The web-based platform is currently in the alpha-release stage for the department of dermatology and partner organizations for user feedback and testing while the complete collection of slides are ingested into the system. Once the complete collection of images are ingested into the system a wider beta-release will be made publicly available for use as an educational tool by medical students, residents, fellows, and faculty in the discipline of dermatology. The next iteration of the project will include mechanisms for accepting image submissions from users and a peer-review process for vetting submitted images to the collection.

From Student to Practitioner: Building Health Science Librarian Skills as an ILS Student Through Co-Creating an InterProfessional Education Activity (22)

Track: Education

Sarah Lopez, MLS (Spring 2023) - Library Science Student, Indiana University Bloomington

Amy Minix - Neuro-Health Sciences Librarian, Indiana University, Bloomington, Indiana

The Interprofessional Practice and Education (IPE) Center was established at Indiana University in 2014, with the goal to extend a culture of collaboration across the state and build stronger and better healthcare teams for the future. In 2021, a new Health Sciences Building (HSB) was opened on the Bloomington campus, which fostered closer collaboration between these formerly disparate departments and schools. A newly established library outpost within the HSB opened fresh conversations about how librarians could support the health sciences. Librarians began meeting with IPE folks to brainstorm an activity that involved literature searching based on a standardized patient’s cursory “research” of their health concerns, as well as looking for evidence-based practice articles to inform or dispel health information. The development of the literature review activity was a joint effort between a neuro-health sciences librarian and a library science student participating in the Funk Fellowship. The Funk Fellowship was established as an outlet for ILS students to gain knowledge and experience as a health information professional. The inaugural fellow worked to create materials that would guide IPE students through a literature search based on timely and personal health concerns from a patient’s perspective. Topics such as using hydroxychloroquine to treat COVID and holistic treatments for periodontal disease were included. Students would search the literature with their respective disciplines in mind (E.g. nursing students would use CINAHL or PubMed to search for relevant literature based on the patient’s concerns); students representing the various intersections of healthcare (optometry, nursing, social work, etc.) would meet as a group along with a faculty facilitator to discuss and share their findings from the literature and talk about how they would address these concerns with the standardized patient. Then students would talk as a group with the standardized patient about the problems outlined in the pre-work. Finally, students would debrief with the facilitator to discuss what went well and what could be improved. The first fellow and the coordinating librarian were able to attend the IPE activity and take notes about the experience. Students had varying degrees of searching, evaluating, and referencing the literature to share with the group; this was one area we quickly identified as needing more scaffolding. Overall, the feedback from the students and facilitators was positive. Students found the exercise to be reflective of a real-life scenario. The IPE curriculum committee is compiling the feedback and will share that with the librarian attached to the project. The second fellow began this Spring 2023 semester and will begin adapting the materials to be reflective of current health concerns and practices.

A Hospital Library Efficiently Meeting Clinicians’ Information Needs: A Virtual Usability Study (1)

Track: Clinical Support

Michele L. Mason-Coles, MLS, CHIS - Clinical Librarian, Darnall Medical Library, Walter Reed National Military Medical Center, Bethesda, Maryland

Ann M. Holman - Library Director, Darnall Medical Library, Walter Reed National Military Medical Center, Bethesda, Maryland

Sarah C. Clarke, MSLS, AHIP - Medical Reference Librarian, Darnall Medical Library, Walter Reed National Military Medical Center, Bethesda, Maryland

Background: The Darnall Medical Library (DML) aims to ensure that the content management system (CMS) for our library aligns with the needs/preferences of our clinicians in accessing resources and information. During COVID, our CMS was reformatted to include all information resources we believed our many clinicians could need given the lack of physical library staff presence. This ongoing project seeks to determine how well our CMS currently meets the needs of our clinicians in their independent attempts to efficiently locate and access the medical evidence when a librarian is not immediately available for assistance. This project is a priority for our library since locating the medical evidence is a vital step in information literacy and in the process of evidence-based medicine.

Description: A web-based survey was developed to identify challenges our clinicians experience when using our CMS. The survey was open from Aug – Sept 2022 and disseminated by email blasts to strategic user groups with a link to the survey. A total of 69 responses were received. Based on the challenges identified in the survey responses, a web-based usability study (US) was developed. The US assessed our clinician experiences as they attempted to complete specific tasks in nine different categories. The US ran from Oct – Nov 2022. During our “Visit the Library Week”, in-person and virtual usability testing (UT) was offered on the first day of the US. Afterwards, participants could complete the UT virtually.

Conclusion: Our virtual US allowed for 100% remote participation. No clinicians came into the physical library to participate in the US despite rigorous marketing. A total of 13 users completed UT virtually. Traditionally, in-person UT requires hours of time, participant coordination, space, equipment, and observers to run the study and record responses. In a busy hospital environment, it is unrealistic to expect clinicians to come into the library to participate in a traditional UT. Virtual UT provides a flexible format to obtain granular feedback and data with which we can improve the efficient and effective use of our CMS for clinicians. We hope this method of virtual UT encourages other libraries to conduct US in our increasingly virtual world.

Disclaimer: The views expressed in this abstract are those of the author(s) and do not necessarily reflect the official policy of the Department of Defense or the U.S. Government.

How One Medical Library Provides Services to Four Hospitals within a New Jersey Health System (3)

Track: Clinical Support

Brittany Haliani, AHIP - Director, Cooperman Barnabas Medical Center, Livingston, New Jersey

Abril Berry - Medical Librarian, Cooperman Barnabas Medical Center

Background: 1. Describe how Cooperman Barnabas Medical Center Medical Library developed a working collaboration with four hospitals within the RWJBarnabas Health system in New Jersey 2. Demonstrate how the Medical Librarians communicate, educate, and disseminate information to medical staff of all four hospitals 3. Describe how the Medical Librarians support nursing and Magnet designation at each hospital

Description: RWJBarnabas Health system includes thirteen hospitals within New Jersey. Not all of these hospitals have access to medical librarians or a medical library. The hospitals are strong teaching hospitals that have robust residency programs funneled from Rutgers Medical School. The majority of our hospitals are seeking or have obtained Magnet Designation. In March 2021, the Director of the Medical Library at Cooperman Barnabas Medical Center (CBMC) was approached by the Chief Medical Officer at that time, to inquire if the Medical Library would be interested in providing Medical Library services to Jersey City Medical Center (JCMC) within the system. The collaboration started with the Medical Library Director working with the Jersey City Director of Graduate Medical Education to establish what services the Medical Library would provide. A legal contract was formally signed by the Graduate Medical Education Directors at both hospitals. The Library Director scheduled an onsite visit to JCMC to establish relationships with key hospital members. Two other RWJBarnabas Health hospitals then asked to be a part of the collaboration: RWJ University

Hospital Rahway in October 2021 and Community Medical Center in September 2022. Both the Library Director and the Medical Librarian are embedded at all four hospitals.

Conclusion: Since March 2021, the success of the collaboration resulted in two other hospitals signing legal contracts for our Medical Library services. In addition, we have been measuring outcomes through statistics. See below for recent outcomes as of December 2022.

Statistics: 2021 Literature Searches Jan.-Dec. 2021: 630; Article Retrieval Jan.-Dec. 2021: 2,900; Reference Questions Jan-Dec. 2021: 2,500. 2022 Literature Searches as of Dec. 2022: 900 (43% increase); Article Retrieval as of Dec. 2022: 3,800 (31% increase); Reference Questions as of Dec. 2022: 4,000 (60% increase).

Improving Outreach Practices with Basic Science Researchers for an Institutional Data Catalog (118)

Track: Information Management

Ummea Urmi - Data Catalog Coordinator, NYU Health Sciences Library, New York, New York

Nicole Contaxis, MLIS, MA - Data Librarian and Lead of Data Discovery, NYU Health Sciences Library, New York, New York

Background: A medical library at an academic medical center developed a data catalog to help researchers locate and share data for re-use and collaboration. Previously, outreach focused on the clinical and population health communities, with 347 datasets cataloged over the 7 years, but very few of these datasets focused on the basic sciences. Therefore, to address the lack of representation of basic science research in the data catalog, the author was hired to improve outreach on the data catalog to the basic science research community at the institution.

Description: The author took several steps to better basic science representation in the data catalog. With a background as a research lab technician, she first needed to familiarize herself with library science research and the data catalog by reviewing relevant literature. Then, to make inroads with the basic science community, she set up meetings with researchers who were publishing research through journals indexed in PubMed but not sharing their data or including Data Availability Statements. Initially, the author began emailing 5 researchers per week with follow-up emails as necessary. After an enthusiastic response to the initial outreach initiative, the author adjusted her process to be able to accommodate the large number of outreach meetings. Because of her efforts, datasets from researchers in the Department of Medicine, Biochemistry and Molecular Pharmacology, Cell Biology, Microbiology, Neurology, and Pathology are represented in the data catalog.

Conclusion: Previously, there were 18 basic science dataset records in the data catalog before the author started targeted basic science outreach. From March to November 2022, the author conducted 40 outreach meetings with the basic sciences researchers. She created 87 basic science dataset records of which 34% were the result of the new outreach initiatives with researchers. Additionally, presentations were given to faculty members in the Department of Biochemistry and Molecular Pharmacology and the Department of Neuroscience and Physiology. In the future, she plans to partner with the Genomic Technology Center (GTC) and Experimental Pathology Research Laboratory (ExPath), shared scientific resources that generate genomic and microscopy data at the institution, to get more leads on microscopy data and pre-publication genomic data.

Increasing Access to Biomedical knowledge at the Bahir Dar University College of Medicine, Bahir Dar, Ethiopia (25)

Track: Health Equity & Global Health

Susan Keller - Librarian, Children's National Medical Center, Washington, District of Columbia

Alemayehu Bisrat, MSC - Assistant Professor, Health Informatics Director, St. Paul’s Hospital Millennium Medical College, Addis Ababa, Adis Abeba, Ethiopia

Eiman Abdulrahman, Md,MPH - Assistant Profession Pediatrics, George Washington University, Emergency Department, Washington

Background: Background • Ethiopian clinicians and researchers have limited access to high-quality biomedical information to help them treat patients and conduct research • Research 4 Life provide free/low cost access to high-quality, leading scientific journals • Many health care providers and librarians in Ethiopia are not aware of the Research 4 Life resources • Training in how to use and access these resources is necessary to overcome this knowledge gap • Other barriers encountered during this project include war, the COVID-19 pandemic, and unreliable electric power Objective: Increase the knowledge, skills and comfort level of resident physicians and librarians these areas: • Accessing biomedical resources • Assessing evidence • Using reference management software programs Setting: Bahir Dar University College of Medicine.

Description: A local expert with an extensive background in the Research 4 Life databases was recruited to teach the classes and he traveled to the campus of the medical school at Bahir Dar University in Bahir Dar, Ethiopia. In Bahir Dar, he trained 20 library staff members and 33 resident physicians during a five-day period. Topics covered included how to navigate biomedical databases such as PubMed, practice in using the Research 4 Life Unified Content Portal, and the use of reference management systems. Both groups completed pre-and post-training questionnaires that included both quantitative and qualitative items.

Conclusion: Among the resident physicians, questionnaire results indicate that comfort/experience searching PubMed, accessing Research 4 Life databases, and the use of reference management programs increased after the classes. However, the residents did not demonstrate a good understanding of the use of the Boolean operator AND. Among the librarians, questionnaire results indicated an increase in comfort/experience searching the biomedical literature with PubMed, accessing the Research 4 Life databases, accessing point of care databases, and using reference management tools. The librarians also demonstrated a good understanding of the use of the Boolean operators OR AND. A three-month post-survey of the resident physicians is planned. Despite many difficulties, the project resulted in increased knowledge/comfort among the participants.

Initial Review of a Demand-Driven Acquisition E-book Pilot Program at an Academic Health Sciences Library (42)

Track: Information Management

Elizabeth Jenkins - Head of User Services, Boston University Alumni Medical Library, Boston, Massachusetts

A'Llyn Ettien, AHIP - Head of Resource Sharing and Discovery, Boston University Alumni Medical Library, Boston, Massachusetts

Background: In September of 2022, an academic health science library at an R1 research institution began a demand-driven acquisition ebook pilot program. Our goal was to cost-effectively increase the number of

biomedical ebooks available to library patrons. DDA provides an acquisition model that allows patrons to participate in the selection process, has the potential to save staff time, ensures that newly acquired materials are used, and provides instant access to new titles. We hope to use this pilot program to determine if DDA is a sustainable acquisition model for our institution. If so, the pilot will help us establish a realistic budget for DDA going forward and allow us to identify the optimal trigger thresholds for titles being loaned and/or purchased.

Description: To implement this program, we created a team of key stakeholders at the library. They were charged with creating a budget, selecting titles, developing workflows, and assessing the outcomes of the pilot program. The team worked with the vendor to select 9,342 biomedical-related titles to add to a DDA collection. MARC records were added to the library catalog, which allowed patrons to browse the expanded selection of ebooks. Our team opted for an access model that allowed for one short-term loan prior to triggering an ebook purchase. To evaluate this program, our team will conduct the following assessments: review the total cost of ebooks loaned and purchased; assess the access model to see if any cost savings were associated with implementing short-term loans; review the breadth of titles activated to see if they can help us identify gaps in our current collection; and review any unexpected challenges faced when implementing our new DDA workflows. Evaluations will be conducted at the 6th and 12th-month marks of the program. Data for this assessment will come from the vendor's collection management site, invoices, and discussions with members of the implementation team.

Conclusion: Our team will review the total spent on ebooks during the pilot. This data will inform revisions to the existing budget and forecasting for the next fiscal year. We will count how many ebooks were purchased or lent. Access models will be adjusted for the best cost savings. The team will review challenges encountered and modify internal workflow as necessary. We will review the subject areas of the ebooks used and see if they provide any insights into our collection’s strengths and weaknesses. We will provide recommendations to the library director as to the suitability of DDA at our institution. Additionally, we hope to provide guidance and identify potential pitfalls other institutions could face when beginning their own DDA program.

Integrating EBM and the Basic Sciences: A Valuable Medical School Assignment (5)

Track: Clinical Support

Deborah A. Crooke - Associate Director and Assistant Professor, Phillip Capozzi, M.D. Health Sciences Library, New York Medical College, Valhalla, New York

Elizabeth Drugge, PhD, MPH - Assistant Professor, Epidemiology Division, Director ECARS, Adjunct Professor of Pharmacology, NYMC School of Health Sciences and Practice, Department of Public Health and NYMC School of Medicine

Background: Medical residency directors report that medical students entering residency need to be better prepared to practice evidence-based medicine (EBM). Even with increased EBM content and librarian involvement in the curriculum, students have been graduating without the competence in biostatistics, epidemiology and critical appraisal that makes evidence-based practice second nature. An ongoing curriculum redesign provided the opportunity to meet our objective to integrate foundational basic sciences with EBM in the first two months, of the first year, of medical school.

A librarian and an epidemiologist, who had trained as a pharmacologist, designed an assignment where students critically appraised a phase III drug trial concerning erythropoiesis, a basic science topic. This poster will outline the content of the assignment and session, explaining how we achieved our objective, the challenges involved, and the way forward.

Description: Medical students report dissatisfaction with a curriculum taught via lectures. So, with only short preparatory classes in EBM and biostatistics, students worked in small groups to critically appraise a phase III drug trial. The two-hour session consisted of three exercises: 1) a search for drug information, where students were required to use resources such as PubMed, PubChem, and Embase to gather pre and post-marketing information on luspatercept; 2) an exploration of study design & methods where students had to identify phases, statistical tests, and techniques of a clinical trial and 3) results & study significance where students had to demonstrate an understanding of a randomized controlled trial by calculating odds ratios, explaining the use of intention to treat analysis, stratification, Kaplan-Meier curves, and more.

Students were divided among nine modules of six groups each. Each module was facilitated by 2-3 faculty members: an EBM expert (a librarian where available) and two basic science professors, working together. Groups were assigned portions of one of three exercises and given 30 minutes to work. In the remaining 1.5 hours, groups presented their answers. Training was provided for facilitators before the session, but most faculty found the material challenging. There were not enough faculty familiar with EBM.

Conclusion: We were able to integrate EBM with basic science in a way we had never done before. Students reacted positively. They liked the hands-on, interactive work better than passively listening to an EBM lecture; their understanding of basic sciences gained a clinical application; and they practiced presentation skills. However, EBM experts were hard to find and most science faculty did not have the EBM skills necessary to carry the session. Although training was provided to faculty before the session, the gap in EBM knowledge was too great to remedy in a short time. Faculty, including librarians, need to develop a greater understanding of critical appraisal to better integrate EBM throughout the curriculum, and engage students in active practice.

Introducing the Data Policy Finder: Helping Researchers Navigate Publication Requirements (44)

Track: Information Management

Donna S. Gibson - Director, Library Services, Memorial Sloan Kettering Library, New York, New York

Anthony J. Dellureficio - Associate Librarian, Data Management, Memorial Sloan Kettering Library, New York, New York

Background: Based on internal conversations with researchers about their publication challenges, a new resource was designed to help researchers navigate the growing body of policies and information by publishers/publications regarding data, code, and other supplemental materials. We identified a critical area within the scholarly publishing process where we could support researchers by identifying data requirements, often buried deep within interior pages on the publishers’ or providers’ journal websites. There is a role for data management librarians to play supporting authors, as well as research quality, and transparency. Researchers can save time using the Data Policy Finder and this resource provides another access point for them to connect with data management services.

Description: In developing this open-source application, information was gathered regarding the scholarly research needs of our authors and the top fifty journals they most frequently publish in as the seed data to populate this resource at product launch. The entries are librarian-reviewed and curated with the intention of adding new content and updating existing information. Attention was given to designing and developing an intuitive tool that required little to no instruction to use. An agile approach was used to develop the interface which started with user stories. We then identified functional requirements, normalized the database structure, and applied user experience design principles. Extensive testing was done with the intended users. Also important was the ability to embed this resource into library webpages and share the application with other interested libraries. By designing a user interface based on API connections and presenting it through a LibGuide, this tool can be more broadly included in other institutions’ platforms. Instructions were

included on the About page for libraries to embed the application within their LibGuides.

Conclusion: The Data Policy Finder is a searchable database containing information, links, direct quotes from the relevant sections of the policies, and notes to help the researcher search, verify, and plan for their publication data requirements. The user can search for data sharing and management policies by publisher or publication and can send an email within the application if they are unable to retrieve the information they seek. Library staff review the policies and curate the database. We hope by providing instructions for other libraries to embed the application, we will encourage collaboration and help with populating the database. Site usage metrics, user survey feedback, and inquiries from other libraries will help us determine resource value and future enhancements.

An Introvert’s Guide to Building Workplace Camaraderie in an Academic Library (58)

Track: Professionalism & Leadership

Christiana M. Keinath - Health Sciences Librarian, Charles C. Sherrod Library, Johnson City, Tennessee

Background: As with so many libraries in recent years, in 2021 employees at my library found ourselves seeking methods to strengthen working relationships and increase feelings of belongingness, after multiple staffing changes and months of work from home due to the Covid-19 pandemic. To achieve these goals, and at the suggestion of my supervisor, I founded the Training and Development (T&D) Committee to plan casual social events and organize professional development opportunities for library employees. Activities have included origami, book discussions, financially supported workshop attendance, forest walks, potlucks, and a pet photo contest. Most events last approximately one hour and require minimal cost, planning, and little to no preparation from attendees.

Description: A small group was formed to plan at least one event per month, as well as three birthday parties annually. In choosing events, we focused on adding an introvert-friendly and gentle level of structure, such as a shared activity or common interest. This helps spark conversations for those who don’t know each other well or are not as comfortable socializing. For example, in spring 2022, we created a shared library Spotify playlist. Then, we held two virtual music hangouts, during which we played the playlist and discussed music. Through experimenting with a variety of formats and topics, we found success by choosing low-commitment activities, creating opportunities to share food and drink, and drawing on free local resources and internal talents. Challenges include choosing times that work with various schedules and accommodating different preferences and needs for virtual vs in person activities. Through anecdotal feedback, I’ve learned that these events have helped people feel more comfortable with one another and learn about their coworkers’ interests and hidden talents. In addition, employees have shared that they enjoy the opportunity to interact with those they don’t often encounter due to working in different departments or different locations in our large building.

Conclusion: One caveat for those wishing to implement a similar program is that social events and workshops are unlikely to be adequate to repair deep-rooted, systemic causes of low morale in libraries, such as low pay, lack of funding, or workplace discrimination. Nevertheless, the response to the T&D committee activities has been very positive, with employees attending regularly and expressing their desire for popular events to be repeated in the future. Our activities have even sparked ideas for new student programming. A survey will be conducted to gather more information on the preferred formats and types of activities, as well as to assess employees’ perceptions of committee activity influences on their sense of belongingness at work and relationships with coworkers.

Leveraging Biological Relationships to More Effectively Search Scientific Literature (71)

Track: Innovation & Research Practice

Sherry L. Winter - Director Biology and Biomedical Solutions, Elsevier, Amsterdam, Netherlands

Carlos Rodriguez del Rio - Product Manager, Elsevier

Ricardo Moreira, n/a - Senior Research Manager, Elsevier

Background: Current literature searching methods are not designed to support biology-focused searches; they lack functionality that allows researchers to pinpoint the most relevant information quickly. Information is ‘lost in the noise’, costing time and increasing the potential to miss important insights. We have designed an intuitive search solution that leverages millions of biological relationships mined from literature and clinical trials – allowing scientists to gain a complete view of what’s known for their area of research and to quickly focus a search, find relevant results and discover new information.

Description: An online survey of 478 published authors/researchers in biology-related fields was conducted, along with over 50 qualitative interviews of researchers working in universities and pharmaceutical companies, to understand current approaches to searching literature, key pain points, and if a biology-relationship-focused approach to searching literature could address challenges. Questions included what tools respondents currently use and time spent searching, along with their level of satisfaction and specific areas seen as good/bad about current approaches. A short video (in the case of online surveys) or live demonstration was shown, of a new approach for searching literature that leverages biological relationships to visualize and filter results. This was followed by questions on what respondents liked/disliked most and how they thought it could help with current pain points. Results were collated and used to make decisions on whether to further develop the new solution.

Conclusion: Researchers search biomedical literature on at least a weekly basis using mostly PubMed and Google and that existing tools are easy to use and provide a good level of information coverage and currency. However, relevancy of search results, ease to identify relevant content from the search results and ease to focus search results using filters/limits scored poorly and can cost time or increase the risk of missing important information that can have a significant impact on the success of a project. Leveraging biological relationships, together with intuitive visuals and filtering options based on these relationships, allowed researchers to find answers more efficiently. Including a visual representation of relationships for the search term provides a global view of what’s already known.

Librarian-Curated Mobile App for Military Hospital with Graduate Medical Education Programs (7)

Track: Clinical Support

Sarah C. Clarke, MSLS, AHIP - Medical Reference Librarian, Darnall Medical Library, Walter Reed National Military Medical Center, Bethesda, Maryland

Ann M. Holman - Library Director, Darnall Medical Library, Walter Reed National Military Medical Center, Bethesda, Maryland

Michele L. Mason-Coles, MLS, CHIS - Clinical Librarian, Darnall Medical Library, Walter Reed National Military Medical Center, Bethesda, Maryland

Background: MilMed is a mobile app specially designed for Walter Reed National Military Medical Center (WRNMMC) staff to quickly access curated program, service, or department content – including house

handbooks, protocols, standard operating procedures (SOPs), and contact lists. It also includes a phone list for the entire hospital. The Darnall Medical Library (DML) inherited the maintenance of MilMed in 2019.

Description: Each June, General Medical Education (GME) programs are contacted via e-mail and provided instructions allowing them to elect to add their specific program year content to be updated and/or uploaded into MilMed. Resources that can be found within the app can include PDFs, images, linkouts to websites, and (limited) messaging capabilities. MilMed installation information is made available during department orientations, mobile apps classes, and our LibGuide. It is available for download on both iOS and Android operating systems. MilMed is also useful to provide information during current events. For example, at the beginning of the COVID-19 pandemic, quick links were added to assist users with point of care needs, and information specific to military health was made easily findable and accessible. Currently, there is major construction at the hospital, and the addition of visitor maps has been helpful with navigation. Feedback for the app can be submitted directly from the app, or to library staff via e-mail. Feedback received directly from the app tends to be requests for updated phone numbers. Feedback received via e-mail tends to be requests for additional resources to be included and/or updated.

Conclusion: MilMed is an important resource to our staff as it allows immediate access to materials, with or without internet access, that may be needed when moving throughout the hospital (i.e. rounding). As of January 25, 2023, we have 1,787 users; 13 have used the app in the last 24 hours, 57 have used the app in the past week, and 147 have used the app in the past month. In the last year, the app received 10,928 pings (application items viewed). Our library is continuously trying to find additional way to connect with patrons to provide them with the information they need at the moment they need it; MilMed has helped further our goal.

Morehouse School of Medicine: Community-borne Bacterial Infections, Spatial-geographic Mapping, and National Library of Medicine (NLM) Resources During the Pandemic (24)

Track: Education

Roland B. Welmaker, Sr. - Library Manager - Technical Services, M. Delmar Edwards, MD Library, Atlanta, Georgia

Joe Swanson, Jr., AHIP - Library Director, M. Delmar Edwards, MD Library, Atlanta, Georgia

Monica Riley, n/a - Serials Librarian, M. Delmar Edwards, MD Library, Atlanta, Georgia

Lynne Simpson, n/a - Library Manager - Information Services, M. Delmar Edwards, MD Library, Atlanta, Georgia

Background: Methicillin-resistant Staphylococcus aureus infections, MRSA, on the decline in recent years, are generally found in hospitals. With community-borne cases, MRSA accounts for most skin and soft tissue infections in outpatient settings in our metropolitan area and nationwide. Local studies indicate racial disparities in MRSA incidence and a lack of knowledge about NLM health information resources. Project objectives are to: 1. identify at risk communities and engage community stakeholders in identifying community-level risks; 2. develop toolkits about preventing MRSA; 3. develop workshops about NLM information resources on MRSA infections and other health topics for healthcare professionals, librarians, and community members; 4. determine the effect of the workshop on participants’ intent to use NLM resources for health-related information.

Description: Librarians identified communities with the highest incidence of community-borne MRSA and the various libraries within those communities and developed a presentation module related to MRSA that could be used by academic, public, and school librarians, health professionals, and community members to acquaint healthcare providers and community members with NLM resources. Within the module was also instruction on meaningful ways to assess the value of websites. Focus group sessions with community

members helped to identify MRSA risk factors in those communities that led to the design of toolkits and of workshops to instruct healthcare professionals, community members, and librarians in the use of pertinent NLM resources. Local healthcare/library professionals were trained on the toolkit developed for teaching clients about risk factors related to community-borne MRSA. Qualitative analysis of focus group sessions NVIVO identified themes and rankings of risk factors. Pretests, posttests, and follow-up instruments were designed to measure participants' baseline, short-term, and long-term intervention knowledge of MRSA, NLM resources, information search practices, and future intentions and actual health information search resources used. SPSS statistical software was used to compare pre- and post-responses and for descriptive analysis of the participants.

Conclusion: Results. 1. Communities with high concentrations of MRSA infections, along with the academic, public, and school librarians and healthcare practitioners within those areas were identified. Desired outcomes (measured using questionnaires) for participants were that they would: 2. become more knowledgeable about ways to prevent the spread of community-borne infectious conditions such as MRSA; 3. become more knowledgeable about NLM consumer health resources; 4. find more NLM resources available in their healthcare providers’ facilities; 5. indicate a preference to search for health-related information using NLM resources; 6. actually indicate, at the twelve-month follow-up, a pattern of using NLM resources for health information.

Moving Forward: Supporting New Librarians at MLA through a New Member Toolkit (72)

Track: Professionalism & Leadership

LaTeesa N. James - Health Sciences Informationist, University of Michigan Taubman Health Sciences Library, Northville, Michigan

Hilary M. Jasmin, MSIS - Assistant Professor, Research and Learning Services Librarian, Health Sciences Library, Memphis, Tennessee

Alessia Zanin-Yost, AHIP - Health Science Librarian, Bailey Library/ SRU, Slippery Rock, Pennsylvania

Steph Hendren, AHIP - Research and Education Librarian, Duke University Medical Center Library, Durham, North Carolina

Background: Professional organizations offer unique opportunities to their members such as continuing education, funds, networking, and engagement at a professional level. For new members of professional organizations like the Medical Library Association, these opportunities may be challenging to navigate and discover. To better understand the needs of new members, their retention, and what outreach efforts are needed, this study investigated the perceived value of becoming a member of MLA. This research emerged from the interests of the 2022-2023 MLA Rising Stars to investigate what potential barriers new members are facing when using the MLA webpage. The questions that lead the study are “How are new members engaged in the association?” and “How does MLA support the retention of new members?”

Description: The authors aimed to answer the two questions by creating a survey that would further inform the creation of a new member toolkit. This initial proposal was accepted by the MLA Rising Stars Leaders. The proposal included a draft of the survey and a timeline to complete the project by May 2023. A test of the survey was completed in December 2022, and adjustments were made accordingly before its launch in January 2023. The survey was sent to members of MLA who had joined since January 2020. All responses will be analyzed and used to inform the creation of a New Member Toolkit. The toolkit will be brought online by a collaborative effort between MLA leadership and the Rising Stars.

Conclusion: Feedback from the data analysis will help the authors to determine what new MLA members need to succeed in their professions. The outcome of the project will consist of developing an online toolkit

for new members of MLA. The website will reside within the MLA webpage and will contain resources, links, and explanations of the resources MLA offers to its members and outline how a new member can become more involved within the MLA community.

Nutrition and Health Information Literacy Program in an Urban Public School System (26)

Track: Education

Matthew Wilcox, AHIP - Director, Edward and Barbara Netter Library, Quinnipiac University, Hamden, Connecticut

Jessica Lohret

Nally A. Sahin - Health and Physical Education K-12, New Haven Public Schools, Branford, Connecticut

Kim C. Rogers - Lead Librarian, New Haven Public Schools CT, New Haven, Connecticut

Background: In Confronting Health Misinformation: The U.S. Surgeon General's Advisory on Building a Healthy Information Environment, the authors state that "Health misinformation is a serious threat to public health. It can cause confusion, sow mistrust, harm people’s health, and undermine public health efforts. Limiting the spread of health misinformation is a moral and civic imperative that will require a whole-of-society effort." We are called to implement health literacy programs in our schools to teach students to identify health misinformation, and to identify quality health information, in order to be able to make more informed decisions and to avoid spreading misinformation. This poster will describe such efforts at an urban school district in New England with 20,000 students.

Description: This project (funded by NNLM Region 7) brings public school health teachers and library media specialists, a medical librarian, medical, and nutritional experts together to develop a health information literacy lessons, and update district curriculum frameworks to ensure health literacy concepts will be incorporated where appropriate. We center on two initial areas: The first is to teach students to evaluate and apply nutritional information so students can make better decisions about diet. This includes creating healthy cooking videos to demonstrate the concepts. Second, we teach students to evaluating health information in order to make better decisions about health claims. Materials will be evaluated to see if students are learning the concepts.

Conclusion: This poster will provide an overview of the project, including how we evaluate the materials, and how we are seeking to further the NNLM mission of “...improving individuals’ access to information to enable them to make informed decisions about their health," while building connections with the school system and medical librarians.

Passive Programming as a Pathway: How to Engage with Heavily Scheduled Patrons (124)

Track: Information Services

Lori Mullooly - Events and Programming Librarian, United States Military Academy Library at West Point, West Point, New York

Lisa Gomez - Exhibition Librarian, United States Military Academy Library at West Point, West Point, New York

Jennifer Chess - Communications and Marketing Librarian, United States Military Academy Library at West Point

Background: A unique challenge for academic librarians is to provide programs and outreach to patrons that are heavily scheduled or have an intense workload. Passive programming is one approach. This poster illustrates one highly successful passive initiative at our special library. This poster also shows why this program is ideal for Health Science Libraries and Librarians, as it may successfully reach unique user groups that may have not used the library in the past.

Description: It can be difficult to create academic library programs and exhibits that are interactive, scholarly, and popular. This is especially challenging at our academic library, where there is an exceptionally heavy demand for student time. This makes finding an acceptable time to host a library program particularly difficult. This poster will present a successful initiative at our library: The Tiny Art Show “Magnificent Miniatures.” Several librarians worked together to create a passive library program that was then used to produce a major art exhibit. Librarians created a LibGuide about this art form to accompany the exhibit which gave our patrons a greater awareness of the library’s resources. This passive program allowed students to create original artwork and engage with one another, the exhibit, and the library collection in a unique and scholarly way. Ultimately, this art exhibit was digitized, which provided further engagement outside of the library and across the wider campus community. Importantly, all of this happened at the library and accommodated available student time.

Conclusion: Passive programs such as the Tiny Art Show “Magnificent Miniatures” are a promising pathway to expanding patron engagement. This program increased the number of visitors to the library (and, once the exhibit was digitized, the number of visitors to our website). This passive program was so popular that it led to unprecedented press coverage and high rates of engagement on social media; all of this led to new collaborations with academic departments.

Peer Review Validation: Tracking Scholarly Activity in a Repository (119)

Track: Information Management

Amanda Schwartz - Digital Asset Librarian, System Library Services, Providence, Missoula, Montana

Background: After several successful years of engagement, a large healthcare system’s institutional repository expanded its services to include tracking peer reviewed versus non-peer reviewed articles by affiliated authors. The repository previously tracked number of article submissions, but did not assess if content was published in peer reviewed journals. The request for peer reviewed content tracking originated from research leadership; administration wanted to measure how much peer reviewed content was being published (in contrast to non-peer reviewed publications) by the healthcare organization on a monthly and annual basis. Thus began the simple question and complicated answer to “How can we retroactively and proactively track peer reviewed articles in our institutional repository?”

Description: Library staff had no previous practices in place to record whether content was peer reviewed or not. The initial request seemed simple yet became increasingly complex to create both a cumulative list of current peer reviewed content and best practice guidelines for tracking future materials. While always looking to automate processes, this specific request required considerable manual work. Library staff created a four-point guideline to identify peer reviewed submissions to the repository. First, assess all articles in the repository. Second, check for duplicate journal titles. Third, gather existing lists of peer reviewed content online. Fourth, hand check remaining journal titles using publisher sites. Library staff exported publication data from the repository and refined it to a single list of journal titles. Next, to complete deliverables at zero-cost, staff reviewed options to identify lists and databases of peer reviewed journals.

Staff exported journal lists and, using a cumulative spreadsheet, implemented duplicate data identifiers to mark peer reviewed journal titles. This identifier helped cut out overall worktime for assessing peer reviewed journal titles. Finally came the arduous task of hand-identifying peer versus non-peer reviewed journal titles in the repository for all titles not captured in online lists.

Conclusion: After finalizing the cumulative spreadsheet and duplicate identifier rules, staff could quickly add journal titles from monthly scholarly submissions, compare titles to what exist on the cumulative spreadsheet, and identify potentially new titles for reporting. The project was well received by research leadership and administration and gives library staff a unique foothold with new and emerging research metrics for the healthcare system. Collecting peer reviewed data has allowed the repository to better report on the number of journal titles submitted, peer versus non-peer reviewed articles, and break down reporting structures monthly and annually for stakeholder needs. It can be invaluable to be able to show quantifiable data of peer reviewed research in institutional repositories.

Pennsylvania Hospital Library: The Nation's First Medical Library Reviews its Past and Embraces Its Future (11)

Track: Clinical Support

Michelle Bass, AHIP - Clinical Information Librarian, Pennsylvania Hospital, Philadelphia, Pennsylvania

Donna Quinn, n/a - Intranet and Instructional Design Specialist, Pennsylvania Hospital, Philadelphia, Pennsylvania

Stacey Peeples, n/a - Curator-Lead Archivist, Medical Library, Philadelphia, Pennsylvania

Barbara B. Cavanaugh - Associate University Librarian for Research Services and Director, STEM and Business Libraries, University of Pennsylvania, Bala Cynwyd, Pennsylvania

Background: Pennsylvania Hospital was founded in 1751 by Dr. Thomas Bond with the endorsement and fundraising support of Benjamin Franklin. In 1762, the hospital received its first book donation from Dr. John Fothergill and thus began the nation’s first medical library. Much has been written about the historic collection and its value to medical history and rare book collections. In this poster, we will take a deeper look at the people behind the collection from the first person to hold the position of librarian in the early 1830s to the first clinical information librarian in the early 2000s. A vision for the library to forge ahead as a leader once again amongst hospital libraries will also be shared.

Description: The library’s collection has been well document with the earliest catalogue printed in 1790. Over time, the number of staff maintaining the collection has ebbed and flowed with hospital finances and technological growth necessitating new expertise. Library leadership embraced the expanded access to information into a transformation of the stand-alone library to the library services unit. A detailed review of the library’s placement in the organizational chart of the Pennsylvania Hospital in the past three decades will illuminate a shift in the clinical provider audience of the library’s collection and services. Maintaining the library’s physical footprint in the hospital remains a priority. Determining the most cost-effective resource formats is another priority for the library, especially in terms of accessibility to information for practitioners at the point-of-care as well as the need for simultaneous access to resources by multiple care providers. Increasing the hospital’s collections for technical services providers and non-clinical personnel, including environmental services and nutrition, are also areas for inclusion in the library’s budget request for the fiscal year. Library services also seeks to be a champion for the hospital’s diversity, equity, and inclusion initiatives through participation in committees and new acquisitions to the collection.

Conclusion: Data collection is already underway on the use of newly acquired electronic resources for certification exam preparation materials. Views to the librarian created resource guides in the LibGuides platform will also be reviewed. Top assets and service line pages will be shared as well as the marketing

and multiple online locations of these resource URLs. The current clinical information librarian will share their vision for the library’s future and ensuring the legacy of this exceptional hospital library remains for future generations of clinicians and information professionals.

Piloting an Interdisciplinary Systematic Review Service (47)

Track: Information Services

Beth Auten, AHIP - Health & Human Services Librarian, J. Murrey Atkins Library, Charlotte, North Carolina

Ryan Harris, AHIP - Associate Dean for Public Services, UNC Charlotte / J. Murrey Atkins Library, Charlotte, North Carolina

Background: An academic library that serves a wide variety of departments and programs was approached about offering a formal systematic review service by its College of Health and Human Services (CHHS). CHHS was willing to provide funding for screening software to help support the service. Some systematic review support had been done for CHHS on an ad hoc basis, supported by the college’s subject librarian. Additionally, another librarian with systematic review expertise had provided systematic review support for faculty in the College of Education. However, a formal service had not been offered by the library.

Description: Due to workload demands and staffing, it was determined that a pilot program would be offered to provide formal systematic review support for up to six research teams. Librarians would work with the teams to develop and execute search strategies, help with the screening software, provide documentation of searches, and write the methodology. Librarians would be co-authors on any publications that came from the pilot. A workshop was developed and offered to faculty at CHHS in the fall of 2022 to gauge interest and give faculty a better understanding of the systematic review process. The workshop discussed various systematic review standards, methodology, and the importance of librarian expertise as part of systematic review teams. Faculty interested in participating in the program submitted a proposal with their research question and research team. Librarians and the Associate Dean for Research from CHHS reviewed submissions and selected research teams. While the workshop was being developed, the Associate Dean for Research from the College of Education reached out to librarians to see if a similar service could be offered for the education faculty. CHHS decided to allow them access to the screening software, and a modified workshop was offered for the education faculty.

Conclusion: Six proposals from CHHS and two from the College of Education were accepted to participate in the pilot program. Searches were to be completed by librarians over the fall and spring semester of the 2022-2023 academic year. In addition to feedback gathered from the participants in the workshop, a feedback form will be distributed to participants in the pilot program to gauge their satisfaction with librarian support. Librarians and the associate deans for research will be keeping track of when reviews are published. Librarians are hoping for continued support from the colleges to provide funding for screening software and to make this a more formalized ongoing service. Additional librarians will be receiving training to help with the searches moving forward.

Planning a Scholarly Publishing Symposium: Lessons Learned (75)

Track: Innovation & Research Practice

Gisela Butera - Biomedical Librarian, National Institutes of Health Library, Bethesda, Maryland

Diane Cooper, AHIP - Biomedical Librarian, National Institutes of Health Library, Bethesda, Maryland

Candice Townsend, MLIS, AHIP - Branch Chief, National Institutes of Health Library, Bethesda, Maryland

Kathleen McGlaughlin, MLIS, PMP - Communications Librarian, National Institutes of Health Library, Bethesda, Maryland

Yolanda L. Jones - Senior Writer-Editor, National Institutes of Health Library

Rebecca Jacob, MSLS - Electronic Resources Management Librarian, National Institutes of Health Library, Bethesda, Maryland

Joelle A. Mornini - Biomedical Librarian, National Institutes of Health Library, Bethesda, Maryland

Background: An important goal of a federal research agency is to produce scholarly publications that promote dissemination and accessibility of information. A federal biomedical library team designed and implemented a symposium aimed at that goal. Here we describe the planning, implementation, and lessons learned from our symposium. A publishing symposium was presented in the fall of 2022, focusing on tools and skills to increase access and visibility of research. The agenda aligned with the governmental public access policy to advance and facilitate the dissemination of scientific research.

Description: A scholarly publishing team was created in January 2022, with six librarians and a leadership liaison. The team organized and implemented a virtual scholarly publishing symposium for the target research community, to be held nine months later. In-house scientists, staff, trainees, and fellows were invited to attend. The team met monthly and focused on finding appropriate speakers and topics that would help researchers promote their research and increase access and visibility or research findings. Guest speakers were recruited for specific topics: research diversity, equity, inclusion; sharing biomedical research data ecosystem; holistic approach to research findings; author profiles and researcher identification; preprints; and disseminating research on social media. Library staff volunteers were enlisted to be slide coordinators running presenters' slides and technical support. A series of dry runs were scheduled with speakers, slide coordinators, and technical support staff. The schedule hosted morning speakers and three afternoon panels. The event was limited to the agency's community only and used Zoom and government Videocasting platforms. A survey was sent to attendees at the end of the event.

Conclusion: Survey results and a post-analysis highlighted success from the event. Important successes came from hosting dry-run sessions with the speakers by testing slides and resolving technical issues. This developed a partnership between speakers and the organizers. Key lessons learned from this symposium include: (1) open future events beyond the federal agency's attendees to include other government agencies, (2) ensure improved representation of inclusivity and diversity when recruiting guest speakers and panelists, (3) add non-library partners to the planning group, and (4) move the event to the summertime to better align with the schedule of trainees, fellows, postdoctoral, and junior investigators.

Promoting Library Employee Wellness & Community Building with a Creativity Wellness Challenge (74)

Track: Professionalism & Leadership

Lydia A. Howes, AHIP - Assistant Librarian, Education and Research, Eccles Health Sciences Library | University of Utah, Salt Lake City, Utah

Samantha Nunn - Project Manager, NNLM Region 4, Spencer S. Eccles Health Sciences Library, Salt Lake City, Utah

Carmin Smoot - Digital Publishing Coordinator, Spencer S. Eccles Health Sciences Library, University of Utah

Kenda Connors

Background: During the pandemic, in an effort to improve employee wellness, an academic health sciences library launched a series of virtual wellness challenges. Challenges run for roughly 6 weeks, and completion is eligible for a University-wide wellness initiative, which provides a discount on the employee healthcare plan. Between July 2020 and August 2022, the Library Wellness Team led 7 challenges. The general feedback was positive, but they wanted to try something new to encourage continued or new participation. Hence, the team developed a challenge centered around creative projects and community building.

Description: The Wellness Team created a short survey with challenge ideas and surveyed library employees. All respondents indicated an interest in the idea of a challenge based on creativity, which had never been done before. The team decided to develop a “Creativity Challenge” as the final challenge of 2022. To earn credit toward the healthcare discount, participants were required to engage in a creative pursuit, periodically attend the library’s weekly craft club, and maintain a tracking form. The Team provided suggestions for potential projects, including crafts and DIY projects, writing, and painting. However, project decisions were left open for participant interpretation. Craft Club is a long-standing group, but attendance had declined during the pandemic despite moving to a hybrid format. For credit, participants had to attend 3 out of 6 in-person or virtual meetings. These check-ins provided an opportunity for community building, encouragement, and to share progress. Additionally, the team posted reminders about the challenge on a Microsoft Teams channel, and participants shared updates on their projects. The tracking form included space to describe progress and reflect on their achievement, challenges, or other thoughts.

Conclusion: 10 participants (of ~50 employees) received credit for the challenge. More participated in part, either by working independently at home or attending some craft club meetings, where attendance increased significantly. The last meeting of the challenge consisted of a hybrid “Show and Tell” event where participants shared what they had been working on. Projects included knitting, crocheting, painting, cooking new recipes, tiling a shower, clay sculpting, needlework, and crafting Halloween decorations. Feedback concerning the challenge has been positive and enlightening, both from within the library and from the Director of the University's Wellness Office, who expressed interest in sharing the program with other departments. The challenge will likely return in the future, rotating with other new or previously successful challenges.

PubMed Hacks That Will Make You Cringe (28)

Track: Education

Molly Knapp, AHIP - Training Development Manager, Eccles Health Sciences Library, University of Utah / NNLM NTO, Houston, Texas

Background: Welcome to a PubMed poster like no other. Because this one answers the question: how to grab someone’s attention in yet another library orientation? Speak the language of today. Embrace your inner cringe with some PubMed hacks that are sure to make one of your learners squirm with secondhand embarrassment. Guess what? The best way to grow is to get a little uncomfortable! Read, like, steal, and subscribe to my top 5 PubMed hacks that will get your users interested in using PubMed, save them time, and spark interest in this deep trove of biomedical knowledge.

Description: This poster will cover 5 PubMed strategies and features that are sure to amaze and intrigue, including: Authorbot, the elusive Phrase Index, time-traveling PMIDs on your inevitable subdermal microchip implant, the ultimate PubMed cheatcode, and the case study that proves they were wrong when they said they want ALL the systematic reviews! This poster concludes with ways to get PubMed to ignore you. Stay to the end, you don’t want to miss this!

Conclusion: What’s your cringeworthy PubMed hack? Like, subscribe, and reply in the comments on this poster!

Putting puzzle pieces together: A library’s efforts in addressing researchers’ publishing needs (125)

Track: Information Services

Ella Hu - Biomedical Sciences Reference & Research Informationist, Shiffman Medical Library, Wayne State University, Michigan

Wendy Wu - Medical Librarian, Shiffman Medical Library, Wayne State University, Detroit, Michigan

Background: Researchers often contact librarians regarding their publishing needs. The inquiries range from finding the best matching journals on their research topics to preserving their research outcomes. Librarians respond to individual inquiries and provide tailored consultations upon being contacted. However, certain types of questions appear more frequently than some other questions. Exploring efficient and effective ways to proactively engage researchers regarding their scholarly publishing activities has become necessary.

Description: By reviewing individual inquiries on scholarly publishing received throughout the recent years, a group of librarians has summarized the most frequently asked questions and developed various approaches to address researchers’ general publishing needs, such as creating research guides on how to choose matching journals, developing workshops on how to identify predatory journals, and introducing methods to promote their research outcomes, etc. This poster will share our efforts and experience on supporting researchers’ scholarly publishing needs.

Conclusion: The outcomes of the supporting services, including the usage stats of research guides and feedback of workshops will be presented and discussed in the poster.

Recipe for Success: Seasoning Faculty Through Nontraditional Mentorship (76)

Track: Professionalism & Leadership

Christine Andresen - Associate Professor/Research and Education Informationist, Medical University of South Carolina, Charleston, South Carolina

Rachel Whitney, AHIP - Research & Education Informationist, MUSC Libraries, Charleston, South Carolina

Sarah Fischer - Outreach and Engagement Strategist, NNLM Region 2

Debra Trogdon-Livingston - User Experience and Education Strategist, NNLM Region 2, South Carolina

Background: Establishing the Network of the National Library of Medicine’s (NNLM) new Region 2 Medical Library (RML) at the Medical University of South Carolina (MUSC) Libraries brought on board faculty and staff members with diverse backgrounds, many of whom had no prior experience working at an academic institution and were new to librarianship. A formal onboarding program with senior faculty mentoring junior faculty was established, but with no shared physical workspace available during a campus renovation, it was challenging to build relationships and form strategic partnerships. Fortunately, a nontraditional approach to mentorship evolved to help these new employees navigate the mysteries of teaching and working in academia.

Description: MUSC Libraries’ leadership ensured that seasoned librarians were paired with new RML faculty members in a formal mentor program to help smooth the transition into health sciences academic

librarianship, but other pairings naturally occurred as employees got to know one another. Virtual communications technologies made remote mentoring possible during the renovation and helped cultivate partnerships. Monthly meetups for coffee, casual conversations, and fun, interdepartmental team-bonding activities like virtual ‘Office Olympics’ events helped foster work relationships and provided an informal environment for mentor/mentee bonding. The formal and informal pairings provided an opportunity for existing/mentoring faculty to share best practices and coach new employees through navigating institutional processes, teaching online, and pursuing research and scholarly interests. During the Fall 2022 semester, experienced faculty mentors worked with mentees to co-facilitate a credit-bearing interprofessional course and to provide a safe environment to get comfortable with the learning management system, interprofessional coursework, teaching in-person and online, engaging with students, grading assignments, etc. Scholarship is another area that can be intimidating for new librarians; nontraditional approaches such as peer-to-peer mentoring offered opportunities to explore joint research and writing projects, and to assist with promotion preparations.

Conclusion: Although the traditional mentoring program wrapped up after a year, nontraditional mentorship activities continued and are proving to be a successful model for onboarding faculty new to librarianship and academia. Co-facilitating the interprofessional course gave new faculty confidence to facilitate the course solo during the Spring 2023 semester as they were better equipped to teach the content and support student learning. The mentor pairings produced conference papers, poster presentations, and in-process publications and research projects, demonstrating the success of the nontraditional method in fostering the development of new faculty’s scholarly work. Ultimately, mentorship is invaluable, and all approaches should be explored to ensure the development of the professional and relational skills necessary to be successful in librarianship and higher education.

The Role of Libraries in Fulfilling Research Data Needs through the All of Us Researcher Workbench (77)

Track: Innovation & Research Practice

Kristi Sadowski - Research Associate 3; Coordinator, All of Us Data Training and Engagement for Academic Libraries Program, Oak Ridge Associated Universities, Southington, Connecticut

DeBran Tarver, PhD, MPH - Evaluation Specialist 3, Oak Ridge Associated Universities (ORAU)

Laura Bartlett, MLS - Technical Information Specialist, Outreach and Special Populations Branch, Bethesda, Maryland

Diane Krause, MS-MPH, CPH, RDN - Health Education Specialist, Public Health and Healthcare, ORAU

Jennifer Ann Burnette, MPH - Project Manager, ORAU

Background: Acknowledging the established role of libraries in research team science, the National Library of Medicine® (NLM®) has partnered with the *All of Us* Research Program, a national longitudinal research initiative and key component of the Precision Medicine Initiative. Through training, data engagement, and funding opportunities to academic libraries, the program will increase awareness and usage of the *All of Us* data set in the Researcher Workbench, which is the cloud-based platform used to access and analyze data. NLM and *All of Us* proposed the creation of a three-year program focusing on health sciences and minority-serving institution (MSI) libraries across the United States, which will train, support, and evaluate the impact of libraries in promoting and using the Researcher Workbench.

Description: *All of Us* seeks to engage academic institutions to sign up and use the Researcher Workbench. Their initial efforts focused on working directly with researchers to increase awareness and offer opportunities to their students and program areas. NLM asked “what about libraries as the center of campus communities, serving as facilitators to the Researcher Workbench?” Strengths of libraries were presented:

flexible, inter-disciplinary nature; service oriented to students, researchers, faculty, and administration; interest in data science and research data management; tech savvy skills including programming languages; and more! NLM proposed a three-year program to facilitate Researcher Workbench usage in libraries, with a focus on MSIs. Broken into two phases, the program first amplified existing learning opportunities, beginning with a focused webinar to libraries. Subsequently, the program will focus on MSI libraries promoting the usage of the Researcher Workbench to early career and seasoned researchers, faculty, students, for applied data science skills. A needs assessment was conducted for MSI institution’s libraries and other faculty/staff to understand their experience and motivations to use data, awareness of *All of Us* and the Researcher Workbench, and the culture of data usage on campus, which informed the development of the program, including recruitment and training materials.

Conclusion: The needs assessment and initial outreach during Love Data Week, influenced the creation of the *All of Us* data training and engagement program for libraries. The program will use the *All of Us* Researcher Workbench and build or strengthen the capacity of libraries at MSIs to support and fulfill the data needs of their campus communities. This 3-year program includes training in phase 1, funding in phase 2 to develop meaningful data engagement, and phase 3 will focus on dissemination of the opportunity to other libraries. Success of this program will be demonstrated overarchingly by increased usage of the *All of Us* Researcher Workbench and more narrowly by increased usage from the academic institutions participating in the program.

Silly Question Program, Systematic Review Caucus (127)

Track: Information Services

Megan DeArmond - Systematic Review Services Librarian, Touro University Nevada, Jay Sexter Library, Henderson, Nevada

Traci Mays, MLIS, BMdS - Health and Human Services Librarian, University Library, Fort Myers, Florida

Stephanie Roth, MLIS, AHIP - Medical Librarian, Christiana Care, Helen F. Graham Cancer Center, Junior Board Cancer Resource Library

Leila S. Ledbetter, AHIP - Research and Education Librarian, Liaison to the School of Nursing, Duke University Medical Center Library, Durham, North Carolina

Jenessa M. McElfresh, AHIP - Systematic Review Service Coordinator / Senior Research & Learning Services Librarian, University of Tennessee Health Science Center Health Sciences Library, Memphis, Tennessee

Patti McCall-Junkin, MA, MLS - Systematic Review Librarian, University of Florida, Academic Research Consulting & Services (ARCS), Gainesville, Florida

Background: The systematic review caucus recognized a need for a safe discussion space. The incoming caucus chair noted that members were actively discussing systematic reviews on social media rather than within the caucus and sensed members felt hesitant to connect on the listserv. The listserv has active "systematic review legends" that often post and respond to queries. In order to increase engagement and participation by those new to systematic reviews or who do not feel qualified to be an "expert," the "silly question" program was created. The name came from mentoring new librarians who would often say, "this is a silly question, but…".

Description: In June 2022, volunteers working with caucus leadership brought the concept to fruition using Padlet to create an anonymous and safe space with an official launch in July 2022. The Padlet has been and currently is actively used by members and allows for complete anonymity and confidentiality. Questions are organized into six categories: search-related questions (databases, strategies), project management questions (service, admin, etc.), protocols/training/registering/publishing questions, scoping review questions, methods questions (guidelines, JBI, Cochrane), and other questions. Once posted to Padlet,

other users can upvote a particular query by clicking on the heart icon. Every two weeks, the SR caucus leaders select a "silly" question to highlight on the listserv. The program is best for non-urgent questions that don't require an immediate response. The caucus leadership generally posts the silly question to the listserv on a Monday and invites membership to engage, allowing for comments for the two weeks. Caucus leadership is not aware of who posts the question, only that membership is interested in getting answers to the question.

Conclusion: Since its inception, this innovative new program continues to evolve. The silly question program has been positively received by our members. This is demonstrated by active engagement in question submission to the Padlet and responses over the listserv. We plan to measure: the number of questions submitted under each category, which questions engaged more discussion and conversation, growth in membership after launch, and how we expect the program to continue to serve the caucus. This assessment will inform and enable us to determine the future direction of the program.

A Solo Librarian’s Systematic Review Service (31)

Track: Information Services

Bridget Jivanelli, AHIP - Medical Librarian, Kim Barrett Memorial Library, HSS Education Institute, New York, New York

Background: Kim Barrett Memorial Library supports the staff of Hospital for Special Surgery (HSS) a leader in education, research, and innovation in musculoskeletal healthcare. Library users include medical students, residents, fellows, attendings, nursing staff, physical therapists, and social workers. The library staff consists of one full time librarian, a part time archivist, a part time assistant, and occasional interns. As interest in performing evidence synthesis projects continues to increase, this librarian set to create a formalized systematic review (SR) service to ensure the institution was producing the best quality research.

Description: The library’s systematic review service began in 2017 when the library was staffed by two full time librarians. Since 2019, the service has continued to operate, evolve, and grow with a solo librarian. As requests for SR assistance increased, the library saw value in adding a SR management software to its resources. In order to access the resource, researchers contacted the librarian to create or review their search strategy. This step provided a level of quality control for searches. The librarian created a SR guide and created an intake form to be filled out before the initial meeting. Two service levels (consultant or co-author) allow researchers to decide on how much librarian involvement they want. The librarian as consultant will offer advice on a search created by the research team. The librarian as co-author will participate in a research consultation, provide instruction on SRs and management software, create the search strategy, provide citation management, retrieve and upload full text, and write the manuscript’s methods section. The librarian continues to improve their SR skills by attending webinars, classes, joining professional organizations and listservs, and keeping up to date on the literature.

Conclusion: Solo librarians may find it daunting to manage a systematic review service. By setting clear expectations for oneself and their users, solos can successfully participate in systematic reviews. Solos should outsource or delegate tasks when possible. This can be done by using systematic review management software or by having research assistants aid in retrieving full text articles. At HSS, librarian involvement in systematic reviews has increased 115% since the beginning of the service with support increasing from 19 SRs in 2017 to 41 SRs in 2022.This librarian has become a trusted research partner by creating a clear service guide, educating their users, and giving researchers realistic expectations of time and of librarian involvement.

Standardizing a Graduate Assistant's Medical Library Training: Creating a Strong Foundation for New Librarians (78)

Track: Professionalism & Leadership

Claire Rhode - Graduate Research Assistant, Preston Medical Library, University of Tennessee Graduate School of Medicine

David Petersen, AHIP - Assistant Professor, Research and Learning Services Librarian, Preston Medical Library, University of Tennessee Graduate School of Medicine, Knoxville, Tennessee

Background: The Preston Medical Library (PML) at the University of Tennessee Graduate School of Medicine has a Graduate Research Assistantship (GRA) program partnered with the University of Tennessee School of Information Sciences. This is a yearlong position, exposing the student to skills and experiences and aiding their professional development. The authors, consisting of the current GRA and supervisor, are developing a standardized curriculum for the GRA. As the GRA is currently supervised by a different faculty member each year, this curriculum is necessary to ensure that all students have an equal opportunity to learn the inner workings of the library, especially as PML is in a teaching hospital and its librarians serve a variety of patrons.

Description: To better facilitate the GRA’s training, the authors are developing a new outline and skills list for the duration of an academic year. The authors seek a flexible model while maintaining core competencies, experiences, and assessments to ensure a constructive learning opportunity. Several steps were taken. First, a list of key trainings and goals was written. Second, an outline for the academic year was created, attempting to plan experiences such as presentations, mock interviews, and outreach at an appropriate time. Key milestones, formative, and summative assessments will be included. Third, an introduction file was created with information about the position, the library, links to training, and other pertinent information. An internal LibGuide will be created to house training information to increase accessibility for future GRAs and supervisors.

Conclusion: Results will be analyzed and presented in the last semester of the GRA’s term. Outcomes will be measured based on verbal interviews, reference desk proficiency, oral presentations, and publications. The authors will promote these training materials to other peer institutions who have graduate assistants working in their medical libraries. Appropriate training is essential to ensuring that graduate students have a strong, practical foundation in the medical library field.

Survey Says! Programming in a Cancer Hospital Library (53)

Track: Information Services

Adela V. Justice, AHIP - Senior Librarian, MD Anderson Cancer Center, Houston, Texas

Graciela Ortiz - Library Associate, The Learning Center/Patient Education, Houston, Texas

Dani Hefner, BA - Library Associate, The Learning Center/Patient Education, Houston

Karen Favela - Library Associate, The Learning Center/Patient Education, Houston

Background: The Learning Center is the patient education/consumer health library of the University of Texas MD Anderson Cancer Center. In September 2022, the library staff sought to expand and reinvigorate services post-COVID by offering new programs to patrons. A month-long survey was conducted to assess the needs of its patrons, which included patients, caregivers, and employees. They were asked in general about their programming preferences and there were open-ended response options allowing for some suggestions or questions as well. This poster will outline the survey process and the results gathered from patrons, as well as look at the future of programming at the library.

Description: Library patrons were asked to complete a survey that asked several questions: their basic demographic (patient, caregiver, or employee), what day and time they would like programming to be held, the format in which the library should hold programs, what types of programs they would enjoy, and a fill-in space to write in any other suggestions they had for the library. Surveys were available in both digital and physical format; paper copies were at each of the two libraries in the cancer center and signs were placed around the libraries with QR codes that could be scanned to access the online version. The survey was also translated into Spanish to accommodate Spanish-speaking patrons. Finally, the survey was open for five weeks, from September 26 to October 28. Because of COVID-19 restrictions no onsite outreach could be done in the hospital, so the survey was only available to patrons already visiting the library. This cut down on the number of replies, but good information was still obtained.

Conclusion: In total, 31 patrons responded to the survey. Most of the respondents were patients, plus several caregivers. Results show that these patrons are indeed eager to participate in programming offered by the library. Patrons were open to all program delivery formats: in-person, online, and pre-recorded. Patrons did not want programs held at the beginning or end of the week and preferred afternoon times. Patrons also heavily favored programs that help relieve stress and anxiety. Therefore, beginning in early 2023 library staff has planned mid-week afternoon Laughter Yoga and will begin passive programs that include providing puzzles, coloring pages, and crayons. Library staff will monitor participation numbers and hope to expand programming availability based on additional patron feedback.

Transformative Agreements – Evaluating Our Impact and Planning For Our Next Phases (50)

Track: Information Management

Rie Goto - Assistant University Librarian, Rita & Frits Markus Library

Matthew Covey, PhD - University Librarian, Rita & Frits Markus Library

Background: We have transferred our journal subscriptions with 8 publishers to Transformative Agreements (TA’s) in the past 2 years. This project is to review and evaluate if our TA’s, specifically to see if they are used, and to see what impact it has for our research community. We used data extracted from OA switchboard, OAble and other open access platforms. The data also illustrated a gap between the publishers where our authors are publishing OA and publishers that we have TAs with. This analysis will allow us to prioritize our efforts for our next phase of negotiations towards more impactful Transformative Agreements.

Description: Since there is no centralized data source for OA publishing data, we collected data from several sources, OA Switchboard, OAble, and contacted publishers not using any platform. We collected articles and information about articles that were published by authors from our institution including, Journal title, Publisher name, corresponding author name, APC fee, and whether it was covered under the library’s various TA’s. We used Microsoft Excel to record, sort, and analyze our data. We also ran searches on Web of Science to assess our author’s open access publishing activities in the same 2 year period and the data will be compared and validated against our collected data.

Conclusion: The data is being collected and analyzed at this time, but we are expecting to see 1) The number of papers published under the library’s TA’s 2) Monetary value (dollar amount saved by our researchers because of our TA’s) as a measure of impact. 3) Titles and publishers that our researchers are publishing OA, that are not yet covered under our TA’s. Our TA with Wiley, the biggest publisher that we have a TA with, is just about to start on February 1, 2023. We are anticipating increased OA publications and hoping to include Wiley data into our final findings

Understanding Tools to Engage Students in a Hybrid Learning Environment (32)

Track: Education

Brandon Patterson - Technology Engagement Librarian, Eccles Health Sciences Library, Salt Lake City, Utah

Adrienne Carey - Assistant Professor, Infectious Diseases & Immunology

Karen Eilbeck, PhD - Professor, Biomedical Informatics

Background: Keeping students engaged in an increasingly distracted learning environment, especially in large classes or in a hybrid environment, can be difficult. The use of audience engagement tools can aid in maintaining student attention to the instruction.

Description: This poster uses two classroom case studies to examine the use of audience engagement tools. A medical microbiology and immunology course used Twitter as a way of disseminating useful information relevant to the course, with prizes given to those interacting the most in daily trivia questions related to course material. Another example used audience engagement software, Poll Everywhere, for a nursing course on evaluation information which invited students to answer questions on their phones or laptops. Twitter and Poll Everywhere were selected because of their ease of use and adoption more widely as a communication method for the schools and colleges involved. This poster explores uses of these tools beyond the basics while providing tips and tricks that support their integration into a course.

Conclusion: The library has embedded use of these tools into technology platforms used for course delivery, which helps engage audiences, especially during one-off teaching sessions. Through course evaluations and follow-up questionnaires the authors collected data about the engagement methods. Overall, students appreciate the interactive nature of using these tools in lectures. They commented about how the tools made the learning content more engaging. Students also tended to perform better on assignments and were more likely to reach out to the library for help when these tools were utilized.

Unintended Results of a Successful Systematic Review Service (57)

Track: Information Services

Mia S. White, AHIP - Medical Education & Technologies Informationist, Woodruff Health Sciences Center Library/Reference and Instructional Services, Atlanta, Georgia

John K. Nemeth - Clinical Informationist, Woodruff Health Sciences Center Library/Reference and Instructional Services, Atlanta

Hannah Rogers - Head of Information Services, Woodruff Health Sciences Center Library, Atlanta, Georgia

Background: Stemming from an analysis of the 160 libraries to determine the ways the subject of authorship was addressed, we created a Systematic Review Service (SRS) agreement that included two tiers of service. Tier 1 included the creation of a PubMed search and Tier 2 included translation of the search into other databases and writing the methods section. Beginning in March 2020, requests for our SRS increased dramatically. Coupled with staff transitions, we had to revise our service to meet demand from faculty, staff, and students. We shifted to a Tier 1 only model and introduced a waiting list to accommodate customers’ needs with available labor and resources in an equitable manner.

Description: To provide impartial service to our customer base, we created an interactive online spreadsheet to triage incoming requests. Eligibility for the service was not changed as we wanted to ensure that all our constituents had equal access to our service. The spreadsheet documents the date of the request as well as other identifying data. Our SR coordinator then assigns reviews based on availability and SR workload of Informationists. Once the reviews are assigned, the Informationist and the principal investigator agree upon a start date for the review. We scaled back to Tier 1 service focusing our efforts on creating an initial well-designed PubMed search. After hiring additional Informationists in the spring of 2023, we anticipate being able to offer our previous level of service that includes two tiers and minimizes the waiting period. Nevertheless, we do not anticipate requests for our expertise to decrease and may need to make additional adjustments to our terms of service in the future to respond to demand.

Conclusion: We plan to reassess the service after onboarding new Informationists.

Using Collective Intelligence Workshops to Create Structured Taxonomies (52)

Track: Information Management

Timothy J. Mulholland, Jr., n/a - Senior Manager, Salt Flats LLC, Insights & Analytics, Chicago, Illinois

Background: Organizations extract value from the information they accumulate by organizing their data logically and consistently into categories and subcategories, creating a taxonomy. A structured taxonomy provides value to data-driven organizations by eliminating duplicative strategies and streamlining knowledge management processes. This taxonomic structure of data and metadata becomes a single source of truth. But organizations struggle with the creation of this taxonomic structure due to communication or platform breakdowns often described as “data silos.” A collective intelligence workshop is an effective interactive method to break through these silos, engage with diverse stakeholders, create consensus, and begin the construction of an optimized taxonomic structure.

Description: Collective intelligence workshops are highly disruptive, collaborative, facilitated workshops hosted in-person and/or virtually that span cross-functional stakeholders to document, ideate, and conceptualize solutions. These workshops enable and promote a diverse set of skills and expertise working together to establish the best possible solution. Challenging the status-quo, stakeholders are asked to describe content as they would in an intuitive manner. From there, results are shared and arranged into a structure where groups interact with the content in this new arrangement, voting and making iterations collectively. This method of conceptual interaction constructs the categories and subcategories across teams and platforms to build a unified taxonomy.

Conclusion: To transform disparate data points into a schema, organizations need collaboration and iterative processes. Interactive workshops can bridge the gap between diverse stakeholders and streamline information and knowledge management objectives. Collective intelligence workshops were performed with a national publishing firm incorporating users in data analytics, content management, search engine optimization, editing, and publishing. These workshops resulted in a new metadata schema and a structured taxonomy that allowed the firm to properly describe and organize content consistently. Within this newly created single source of truth stakeholders were able to make data-driven decisions on content across the organization, driving traffic, and eliminating duplicative efforts.

Using a Work Breakdown Structure to Look Back at a Medical Librarian's Role and Enhance Efficiency in Future Guideline Development (128)

Track: Information Services

Marisol Hernandez - Medical Librarian Specialist, College of American Pathologists, Northfield, Illinois

Carol F. Colasacco, AHIP - Medical Librarian Specialist, College of American Pathologists, Northfield, Illinois

Kearin Reid, AHIP - Medical Librarian, College of American Pathologists, Northfield, Illinois

Background: Guideline development is a complex process involving multiple tasks completed by several team members. The poster aims to better illustrate the Medical Librarian (ML) role during each stage of guideline development and demonstrate the ML's proactive approach throughout the guideline development process. The guideline development team created a detailed Work Breakdown Structure (WBS) to delineate the numerous responsibilities in a 13-stage process for establishing evidence-based guidance and recommendations for best practice. Note, the WBS did not align specific team members with each task. The goal was to specify ML responsibilities within the WBS, confirm that written procedures reflected ML responsibilities, and ensure ML onboarding materials were consistent with the WBS.

Description: For this project, the focus is on the expert and collaborative role of the ML throughout the guideline development process. First, a primary spreadsheet was created to include each step of the WBS. A brainstorm session identified all potential ML tasks. Next, all written procedures were mapped to the WBS, confirming that ML tasks were delineated appropriately throughout the process, including the initial environmental scan completed during topic exploration, the entire systematic review process, manuscript development, dissemination, and post-publication awareness and impact. Finally, current documents were assessed for accuracy. During this process, several new procedures were written, and existing procedures were updated to better reflect identified activities. These updates and documents were used to create more comprehensive and up-to-date ML training modules mapped to the WBS in preparation for onboarding a new ML. While onboarding, additional WBS and written procedure edits were noted. The evaluation of written procedures and onboarding modules is ongoing as part of routine project close-out activities when issues are investigated, and remediation occurs.

Conclusion: A look back at the entire guideline development process as outlined in the WBS, illustrated the detailed role the medical librarian plays from topic submission through final publication of a guideline. The aim to increase overall efficiency by promoting a proactive rather than reactive approach to assigned tasks by creating new and updated documentation, including more comprehensive training modules, also facilitated effective new staff onboarding. Future assessment by relevant stakeholders will be used to review and edit materials and support continued quality improvement efforts to streamline processes and improve overall efficiency of clinical practice guideline development.

What's the Big Deal? Preparing for Transformative Agreements (54)

Track: Information Management

Rebecca Gerber - Electronic Resources Librarian, Lamar Soutter Library / Library Operations, Worcester, Massachusetts

Sally A. Gore - Manager, Research & Scholarly Communication Services, Lamar Soutter Library, UMass Chan Medical School, Worcester, Massachusetts

Leah Honor - Research Data & Scholarly Communications Librarian, Lamar Soutter Library, UMass Chan Medical School

Tess Grynoch - Research Data & Scholarly Communications Librarian, Lamar Soutter Library, UMass Chan Medical School

Lisa A. Palmer, AHIP - Institutional Repository Librarian, UMass Chan Medical School, Worcester, Massachusetts

Background: Since the open access (OA) movement emerged more than two decades ago, libraries, publishers, funders and researchers have struggled with sustainable publishing models in the complex scholarly communication environment. Fully OA journals typically use article processing charges (APCs) to generate revenue and many other publishers have moved to this model to offer immediate OA to individual articles. With growing scrutiny on library expenses, publishers have begun to offer transformative, or Read-and-Publish, agreements, which shift payment from subscription-based reading toward OA publishing. Collection development librarians handle subscriptions (“Read”) and Scholarly Communications librarians assist researchers with grants and publishing (“Publish”). There is now an opportunity to work together in the decision-making process to gather data and analyze these agreements.

Description: The library has collected usage data for subscription resources for many years and used that information to inform future acquisitions or renewals. The collection of institution-affiliated author publishing data has not previously been part of this determination, but with the growing push by publishers to sign Read & Publish agreements, we have begun creating a new process. Our ultimate goal is to develop evaluation criteria for the value and benefit of transformative agreements and propose a strategy for approaching university administration and department chairs for funding of transformative agreements. Several steps will need to be completed to reach this goal. For each vendor/publisher, we want to collect and integrate author publishing data with subscription usage data to determine the level of library interest in pursuing a transformative agreement. Author publishing data could include APC dollars paid, number of articles, and departmental affiliation. Determining how APCS are typically paid across the institution, whether by individual grant, by department, or some other fund, can provide a talking point for best ways to fund APCs. Finally, we want to determine the most appropriate ways to disseminate and promote information to the university community on any current and future transformative agreements.

Conclusion: We expect several outcomes from this initiative. We will create an internal campus portal for data concerning the costs of subscriptions and OA publishing in those journals, usage of subscriptions, and any pertinent background information that could impact a decision about transformative agreements. This portal would also allow university authors to determine what publications or publishers have agreements with the university and request assistance from scholarly communications librarians in determining the best place to publish. The evaluation criteria we develop will be utilized in the decision-making process. Most of all, we expect to see an overall decrease in costs associated with subscriptions and research publishing fees.

With Our Powers Combined—We Are Captain Digital Accessibility! (34)

Track: Education

Christine Andresen - Associate Professor/Research and Education Informationist, Medical University of South Carolina, Charleston, South Carolina

Background: Digital accessibility refers to the ability of electronic documents, websites, videos, products, and virtual environments to be easily navigated by every user, including those users who have visual, auditory, motor, or cognitive disabilities. Naturally, this includes the articles, ebooks, videos, and other resource types available in library collections. To support faculty in understanding the importance of digital

accessibility and best practices for creating accessible content, a digital accessibility course was developed using the institution's learning management system (LMS), and a librarian was recruited to serve as one of the course facilitators and to develop the instructional materials for the module on best practices for accessible library resources.

Description: The digital accessibility course consists of 12 modules designed to educate faculty on the basics of digital accessibility, universal design for learning, general best practices for creating accessible documents, and making library resources accessible in the LMS. The course is available with a certificate option requiring learners to complete assignments and earning four hours of Diversity & Inclusion credit hours. One of those assignments is in the library resource accessibility module and allows faculty to gain experience replacing unaccessible journal article PDFs in their own courses with authenticated hyperlinks that allow students to access either the HTML or PDF versions of articles from on- or off-campus. Participants complete a pre- and post-assessment survey to gauge their knowledge on digital accessibility, as well as submit course feedback for evaluation, all of which is used to continuously improve the course.

Conclusion: Since Fall 2019, over 600 course participants completed the course and of those participants, 106 were awarded digital badges, and over 400 hours of Diversity & Inclusion credits have been awarded. During this same reporting period, over 1000 unaccessible journal article PDFs were removed from the LMS and replaced with accessible authenticated permalinks to those articles available in library collections.

Working Smarter Not Harder: Automating Full-Text Retrieval for Systematic and Scoping Reviews at a Small Academic Health Sciences Library (59)

Track: Information Services

Elizabeth Q. Huggins - Research and Education Librarian, Health Sciences Library, Loyola University Chicago, Maywood, Illinois

Christopher Beger - Interlibrary Loan and Access Services Specialist, Health Sciences Library, Loyola University Chicago, Maywood

Background: A small AHSL recently formalized its evidence synthesis service and outlined the specific services through the co-author and advisor models. The co-author includes full-text retrieval for the team. As of January 2023, our health sciences library has a small staff: three full-time librarians and one paraprofessional staff member. Without the ability to utilize a cohort of librarians, staff, and student workers, we faced the significant challenge of retrieving full text for systematic and scoping reviews.

Description: A librarian and staff member collaborated to identify and create processes to retrieve full-text and limit hand retrieval to as few citations as possible. We started by using the automated full-text retrieval in EndNote for the initial batch. Articles not retrieved through this process were exported to an excel sheet and sent to the staff member. The staff member created a python script to check for full text through the libraries’ link resolver. This process is executed twice, once through AHSL’s local collection (catching any that EndNote missed) and once through the institution’s larger library system. The remaining articles are then converted to DOIs (Digital Object Identifier) or PMIDs (PubMed ID) to accelerate the process of requesting items through interlibrary loan. The full-text PDFs are then shared with the team. Methods for sharing have included a controlled folder in the institution’s cloud service; EndNote compressed library; file export from EndNote to Covidence. For EndNote and Covidence management, the script and interlibrary loan retrieved PDFs are added through the bulk PDF import function and then deduplicated.

Conclusion: Evidence synthesis services are growing in health sciences and general education libraries. However, the scope of work can be challenging for small libraries. Leveraging the skills of different team members and evaluating the strengths of existing tools enabled our small department to rise to meet the

burgeoning service that supports the institution’s research and publication needs. This program is in its infancy, but the results so far have been positive. The team continues to identify minor weaknesses and address them to increase the project's inefficiency. These types of collaboration are key in library and information services as they demonstrate the power that comes from a diverse team and help small libraries contribute to the evidence produced in health sciences.

Yes! You Do Really Need to Write and Register Your Review Protocol (79)

Track: Innovation & Research Practice

Gisela Butera - Biomedical Librarian, National Institutes of Health Library, Bethesda, Maryland

Alicia A. Livinski - Biomedical Librarian, National Institutes of Health Library, Bethesda, Maryland

Nancy H. Terry - Biomedical Librarian, National Institutes of Health Library, Bethesda, Maryland

Nancy L. Muir - Director, National Institutes of Health, Maryland

Background: A protocol is the road map prespecifying the process for conducting a review. It states the research question, methods to follow, and decisions made. Creating the protocol a priori requires the team to determine how to conduct each step of the review process to reduce bias and minimize and prevent errors. By depositing the protocol in a repository or publishing in a journal, transparency is increased, and duplication of efforts is reduced. Unfortunately, many review teams do not write a protocol or make it publicly available. We will describe how the Evidence Synthesis Review Service (ESRS) at a federal biomedical library incorporated the support of writing and dissemination of protocols to improve their quality, reduce confusion, and prevent abandonment.

Description: The ESRS service consists of four librarians working in-depth with review teams composed of scientific and public health investigators, fellows, and students. In 2022, a strategy to improve the writing and registering of protocols was implemented as a required deliverable part of the review process. During the first meeting, before discussing search strategies, we discuss writing the protocol, share examples, including a protocol template. Protocol templates for both systematic and scoping reviews were created including example wording. The systematic review protocol template incorporated the required fields for PROSPERO as the framework and the PRISMA for Protocols (PRISMA-P) for guidance on best practices. For scoping reviews, the PRISMA extension for Scoping Reviews and JBI template were used. The ESRS members provide significant edits and improvements to clarify and strengthen the proposed methods. For registration, we consulted Open Science Framework (OSF) to understand the registration process and template to generate a DOI. A handout on how to register a protocol in OSF was created and a video is planned. A class that was already in place, Developing and Publishing Your Review Protocol, was updated to include the new strategy emphasizing the importance of review protocols.

Conclusion: As a result of this effort, ESRS members improved their knowledge and recognized the importance of review protocols, leading to better advocacy for review teams and assistance with writing and publishing protocols. By having the review teams document their methods a priori, we helped to add rigor, ensuring appropriate methods were used for screening, data extraction, risk of bias and data synthesis. Anecdotally, more review teams are writing protocols, and many are publishing or registering on PROSPERO or OSF–often with a librarian co-author. An evaluation of this support is planned for 2023–2024. Future plans include creating an asynchronous OSF protocol registration module, compiling examples of published and registered protocols, and improving internal recordkeeping for capturing librarian protocol support.

A Bone Has Obligations: Anatomy Circulation at a Health Science Library (8)

Track: Education

Alexia Sheck - Librarian, University of St. Augustine for Health Sciences, St. Augustine, Florida

Adam Mills - Circulation Manager, University of St. Augustine, St. Augustine, Florida

Esther Garcia, MLIS - Librarian, University of St. Augustine for Health Sciences, Frisco, Texas

Background: In academic health science institutions, anatomy courses are foundational to the curriculum. Students typically learn anatomy through cadaver dissection, prosections, plastination, and computer-based learning. Libraries can support anatomy learning with lower cost and analog options, for example, bone and anatomy models. Circulating bone models and other anatomy objects are services that are well-received by students and contribute to their success.

Description: At our academic library, we provide circulation services, which include bone models, muscle models, assistive devices, equipment, and other anatomy models. Circulation statistics show that bone and anatomy models are frequently accessed and used resources. Kinesthetic learning and spatial learning are essential to student success because students have varying learning styles. By contributing to student experiential learning, we have found bone model usage at home enhances student supplemental education. We will discuss the value of libraries supporting anatomy curriculum, and explore the circulation rate before, during, and after the COVID-19 shutdown. The circulation of anatomy models isn't without its challenges. Repairs, storage, and costs are constant challenges that must be considered. We will address how our libraries work out these issues and suggest ideas that may be implemented by others.

Conclusion: Due to limitations with cadavers, plastination, and costly software, libraries can support students in loaning anatomical models for at-home experiential learning. Library staff have seen the need, as anatomy models far outpace the circulation rate of other library items.

RESEARCH TRAINING INSTITUTE (RTI) POSTERS

Although poster numbers are included, content in this section is sorted by title in alphabetical order. Posters include early notice of research, research initiatives in process, and research already conducted.

Addressing Accessibility Issues of Library Databases Among Undergraduate Students with Learning Challenges (RTI114)

Ebony Peterson

[No additional information provided]

Assessing Bias in Oncology Research through Informatics (RTI109)

Ciara Seals

[No additional information provided]

Assessing Language about Diversity, Equity, and Inclusion in the Collection Development Policies of AAHSL Member Libraries (RTI105)

Kristen Burroughs - Fellow, Research Training Institute, MLA

Objective: This study analyzed the collection development policies of members of the Association of Academic Health Sciences Libraries (AAHSL) in the United States to assess the presence and context of language related to diversity, equity, and inclusion (DEI). Recommendations for DEI-related language in these policies are provided.

Methods: Seventy-two policies were obtained from 161 AAHSL member libraries in the United States. Policies were qualitatively coded using an inductive codebook created for this study, which identified common themes and contexts of DEI concepts.

Results: Of the 72 policies analyzed, 32 (44.4%) contained DEI-related language and 40 (55.5%) did not. Common themes in the policies with DEI-related language included multiple contexts of diversity including diversity of perspectives, topics, and authorship, diversity of users, commitment to other diversity policies, and a general commitment to diversity as a concept. Other themes include accessibility for people with disabilities, equitable access to information, equitable spaces, inclusive environments, and references to health disparities and cultural competence. Findings also identified vague language about DEI concepts, a lack of publicly available policy documents, and wide variation in collection development policies.

Conclusions: Results suggest significant room for improvement in DEI-related language in AAHSL member

library collection development policies. Health sciences librarians should consider incorporating DEI-related language throughout policies, tailoring language to be institution- and library-specific, making policy documents that highlight DEI efforts publicly available, using more specific language about DEI, and addressing multiple types of diversity.

Consensus for Impact Variable Data Point Terms and Definitions: A Delphi Study (RTI03)

Gwen Wilson, AHIP - Medical Librarian, Dept Family and Community Medicine, University of Missouri, Columbia, Missouri

Objective: Reach a consensus of impact variable data point terms and definitions that could be incorporated into existing reference library tracking systems.

Method: Utilizing the Delphi methodology, informational professionals were recruited to participate in focus groups. Based on the literature and a pilot focus group impact variable data point terms and definitions were drafted. Next the structured focus groups applied an adapted version of the norming process. The norming process utilized example reference service scenarios, participants were asked which impact variable data point best to fit that scenario and if there were conflicting impact area terms the participants were asked what revisions to either the terms and/or definition would provide clarity. Focus groups continued building on the revisions of the previous focus group until the Delphi technique showed a saturation for consensus.

Results: Between the three rounds of focus groups there were 17 information professional participants. The first-round focus group (n=5) made several edits to the impact variable data point terms (i.e., changing research to scholarly) and definitions including the addition of a new impact area (i.e., academic success). Following the Delphi technique, the second focus group (n=6) started with the revised impact variable data point terms and definitions from the first focus group. The second focus group made a few edits to the naming of the terms (i.e., adding risk management to quality improvement), definitions and added a new impact area (i.e., institutional success). The third focus group (n=6) only made a small edit to one definition rewording the phrasing (i.e., moving the phrase for implementation). With no substantial changes to the impact variable data point terms and definitions during the third focus group it was concluded that consensus was made. Participants agreed that these terms and definitions are acceptable for implementation into practice as a new data point within existing reference service statistics.

Conclusion: This Delphi study is the first with the objective to develop a consensus of impact variable data point terms and definitions to be utilized as a category within reference library tracking systems. The impact terms and definitions developed through this study have reached a saturation of consensus. This consensus is a great foundation for libraries to implement these terms and definitions as a new category within their reference library tracking system.

Consumer Health Information on Public Library Websites: Availability and Characteristics (RTI101)

Laurie Najjar - Reference Librarian, Austin Public Library

Objectives: As the availability of online health information grows, the internet has become a common source, but consumers can become frustrated by the volume of information or their inability to locate what they seek. In addition, their search practices can lead them to inaccurate or outdated information. As a result, consumers need help in sorting through the overwhelming volume of information and selecting

reliable sources. As trusted sources, public libraries can play a role; in fact, many have established consumer health information (CHI) sections on their websites. However, research about the prevalence of this practice is limited and dated; the most recent study is more than 20 years old. Therefore, this study sought to measure the availability and characteristics of CHI on public library websites. In addition, it hypothesized a relationship between library (community) size and three key variables: 1) the presence of CHI, 2) the number of CHI sources, and 3) the number of clicks required to reach the sources. Methods Using a comprehensive dataset of U.S. public libraries from the Institute of Museum and Library Services, a random sample of 200 libraries was selected to represent varying community sizes. Content analysis was used to gather and code the data found on each website and statistical tests (Chi square, Kruskal-Wallis) were used to test the stated hypotheses.

Results: On the key research questions, we found that 55% of the libraries provided online CHI, and that the average site offered 28 sources and required two clicks to reach the sources. Regarding the hypotheses, tests of significance showed no relationship between library size and the three selected variables. Among other findings, the study found that on average, 86% of each site's sources were authoritative and 91% of each site's links were functional; most libraries annotated their sources (73%) and offered local sources (58%); and only 16% of libraries provided tips for how to locate trustworthy online information. An unanticipated finding was that a third of the libraries collaborated either by sharing sources (as evidenced by identical content and layout) or by linking to content from a larger library, usually a state or university health sciences library. A significant relationship was found between library size and use of collaboration; specifically, collaborations were used most often by small libraries and least often by very large libraries.

Conclusions: It was surprising that only slightly more than half of public libraries offer online CHI. It was also surprising that a library's capacity to provide CHI is unaffected by size, which seems to challenge the assumption that larger libraries are better resourced. One explanation may be that larger libraries are better resourced, but smaller libraries compensate by collaborating. This is supported by the finding that collaboration is used most often by small libraries. Promoting collaboration—especially by smaller libraries—could, therefore, boost the overall availability of CHI in public libraries. Other benefits of collaboration could include improved quality (e.g., more authoritative sources) and greater efficiency (e.g., fewer staff needed) by pooling resources. In this way, collaboration could be a practical way to expand CHI availability, while also improving quality and managing costs.

Disempowering Terminology in Online Patient Education Materials for People with Type 1 Diabetes: Progress on a Summative Content Analysis (RTI111)

Lisa M. Acuff, AHIP - Education and Research Librarian, University of New Mexico, Health Sciences Library and Informatics Center

Objectives: Type 1 diabetes (T1D) is a complex chronic disease requiring ongoing self-management. Patient education aims to empower people to set goals and choose behaviors that accommodate their lives and support optimal health outcomes. Disempowering language, however, can undermine self-management and psychosocial outcomes. Disempowering language includes stigmatizing, blaming, and condition-first words or phrases. The objective of this study is to assess online patient education materials (PEMs) about T1D for the presence of disempowering language using summative content analysis. This poster reports findings from a pilot study of Phase 1 of the content analysis.

Methods: Summative content analysis quantifies word frequencies (Phase 1) and explores the context of usage (Phase 2). We developed and pilot tested a sampling protocol for Phase 1. Based on the results, the procedures were refined and implemented. Included PEMs were publicly available, specific to T1D, intended as patient education, available for download, and relevant to newly diagnosed patients. Advertisements,

multimedia, resource lists, and manuals were excluded. A data collection checklist of consensus-based disempowering terminology was also created and pilot tested for Phase 1. Implementation of the checklist is underway. Phase 2 will use Dedoose or NVivo to code and describe the context of disempowering word usage.

Results: The Phase 1 pilot study assessed 11 PEMs. Clinical, government, and academic authors were represented. Total instances of disempowering language within individual PEMs ranged from 0 to 28. Across all materials, frequencies were highest in the Imperatives category. Imperatives included variations of should, have to, and must. The second highest category was Prevent, which included the variations preventing and preventions. Results from the implementation of the refined sampling protocol produced 255 online PEMs. Six Google search strings were used to harvest the sample. The materials were screened by two independent researchers. Thirty-two PEMs were identified for inclusion.

Conclusions: After the Phase 1 data collection checklist is completed, frequencies and ratios of disempowering words and phrases will be calculated both within and across online PEMs. Differences between materials by audience and authorship will also be calculated. Phase 2 of the summative content analysis will begin after the completion of Phase 1.

Exploring Clinician Expectations and Preferences of Library Subject Guides (RTI02)

Anna Biszaha - Assistant Professor/Research & Education Librarian, The Ohio State University Health Sciences Library, Columbus, Ohio

Objective: Subject guides are widely used within academic libraries as a means for librarians to share resources and information to potential users or patrons. Much of the literature concerning subject guides discuss them in the context of student or academic use; however, academic medical center and hospital libraries must also consider the needs of clinical users. This study seeks to qualitatively explore the perspectives of clinicians regarding library subject guides.

Methods: A series of 4-8 semi-structured focus groups will be held with clinicians. Focus group discussions will explore the types of information that clinical users think should be included in a subject guide, as well as what they think the purpose of subject guides are and what they would consider to be most important when using one. Additionally, it will ask participants to consider what role library subject guides might play in the course of their clinical work and if they have either experienced or foresee barriers to using them in a clinical setting.

In the event that a target minimum of focus group participants are unable to be recruited, the researcher will pivot to using individual interview methodology with the same essential script and purpose.

Results: The study is currently underway.

Conclusion: The researcher had previously conducted a survey of Health Sciences Library subject guide users and had intended to perform an initial comparison between student and clinical users; however, not enough clinical users participated in the survey to make meaningful comparisons. As such, this study is intended to better target and explore this sub-population of HSL users. It is hoped that the results from this study will inform library subject guide development and customization strategies that libraries can use to better support and target clinical users.

Filling the Gaps: Collecting Instruction Data for Meaningful Benchmarking (RTI102)

Ellie Svoboda, AHIP - Education Informationist, University of Colorado Anschutz Medical Campus, Colorado

Benchmarking is a valuable tool for academic libraries to make informed decisions and actions in line with the practices of their peers. The Association of Academic Health Sciences Libraries (AAHSL) conducts an annual survey that consolidates data on the practices of AAHSL libraries regarding staffing, collections, access, instruction, and much more. This data is useful; however, there are some gaps in the instructional data that can hinder robust decision making. This project aims to remedy this by surveying AAHSL libraries for more granular data related to their instructional activities. In spring of 2023, a survey was sent to AAHSL libraries and received 34 responses. This poster will highlight the preliminary findings of that data and begin to fill the gaps.

Food Security Programming in Missouri Public Libraries (RTI108)

Alex M. Henigman - Student, Emporia State University

The purpose of this study is to identify if there is a need for food security programming in Missouri public libraries. Food insecurity is defined by the United Nation’s Committee of World Food Insecurity as the “means that all people, at all times, have physical, social, and economic access to sufficient, safe, and nutritious foods that meets their food preferences and dietary needs for an active and healthy life” (United Nations). In Missouri, over half a million people face hunger across the state (Hunger in Missouri). Offering food security programming at public libraries in Missouri may assist with food security measures in the state as many public libraries serve food insecure communities. This study will be conducted by surveying a purposive sample of one representative from every tax-supported public library in Missouri. The survey will collect data on which libraries currently offer food security programming, what the perceived barriers are for libraries that do not offer food security programming, what types of programs are currently offered in the state, and how these programs are funded. Results will undergo a t-test to see if there is a significant correlation between food insecure counties and libraries that do not offer food security programming. The intended results will showcase that many libraries serve food insecure counties but do not yet offer food security programs for their communities, justifying a need to begin these offerings.

A Graphic Medicine Reading Experience for Undergraduate Nursing Students (RTI01)

Michelle Nielsen Ott - Sciences/Health Sciences Librarian, Bradley University, Cullom-Davis Library

In spring 2021, an open letter from the LGBTQ+ community was written to the campus community at large. As a response to the open letter, this study was designed to determine if integrating a graphic medicine title into a required course in the undergraduate nursing curriculum was a beneficial way for students to learn about a population that is not covered well in the standard curriculum--in this case, people who are transgender. In partnership with community health nursing faculty members, undergraduate nursing students were assigned to read First Year Out: A Transition Story by Sabrina Symington and answer short reflection questions about their reading experience. The written responses to the reflection questions were analyzed for themes. Preliminary data analysis reveals that the students were able to relate to the main character and acknowledge plans to have more inclusionary practices as healthcare providers. In addition, students found the comic format enjoyable. We plan to continue to include graphic medicine to advance

learning about nondominant populations and cultures and will work to integrate graphic medicine throughout the undergraduate curriculum.

Health Information Needs of Hospitalized Senior Patients (RTI113)

Ellen Kearns - Graduate Student, Emporia State University School of Library and Information Management, Lawrence, Kansas

Previous studies have indicated that senior citizens in the United States have lower rates of health literacy yet are the largest consumers of health care and experience higher rates of hospitalization than other demographic groups. During hospitalizations, senior patients may have greater health information needs necessary to make informed health decisions. Continued research is needed to gather data about the health information needs and health information seeking behaviors of senior patients to identify gaps in access to health information.

The objective of this study is to determine the health information needs and seeking behaviors of senior patients during hospitalization. The study will rely on the gathering of quantitative data through distribution of a paper survey consisting of questions specific to the methods employed and resources used by senior patients when accessing health information while hospitalized. The survey includes multiple choice and ‘yes’ or ‘no’ questions but provides options for participants to fill in responses as needed. Prior to distribution, the survey will be piloted amongst a small group of senior participants. Results from the pilot survey will guide modification of the survey as needed. A paper version of the modified survey will be distributed in early fall 2023 at local senior community centers and events, pending approval. Collected data from the survey will be analyzed in the fall of 2023.

Health Literacy Workshops: Librarian Support in Employee Wellness Programs (RTI08)

Colleen M. Foy, AHIP - Research & Instruction Librarian for the Sciences, Wake Forest University

Objective: How does librarian-facilitated literacy instruction impact health information seeking attitudes and behaviors in a medically directed employee wellness program?

Methods: Existing health literacy frameworks guided instruction and survey design methods including Don Nutbeam’s Health Literacy Model and the Research Triangle Institute Health Literacy Skills Concept Model. The literature provided examples of health literacy (HL) impacts and factors including wellness program participation components (like reach and retention) and results (like weight loss), the efficacies of healthcare organization patient materials and staff communication methods following HL training, and corporate wellness programs intervening with varied formats of materials and assessments via health risk appraisals. With the study objective and research question in mind, however, the literature was missing prime examples of librarian-facilitated health information literacy instruction models. Taking a quantitative approach to a quasi-experimental methodology (without a control group), instruction and pre-post tests were designed for participants in predetermined cohorts of a medically directed employee wellness program at Wake Forest University. Participants were invited to take a 6-question digital survey before and after workshops allowing for the collection of data identifying baseline and completion HL indicators. Responses were collected, coded, and analyzed using a sequential explanatory design and various statistical measures. The intervention included live virtual 60-minute Health Information Literacy Instruction Workshops to assist participants in making informed decisions regarding finding, evaluating, and using health information in

digital environments. Workshop content incorporated educational and behavioral tools, engagement activities utilizing social media content via break out rooms, and database and website demonstrations.

Results: Using Qualtrics and Microsoft Excel tools, data was collected and analyzed. When comparing pre and post workshop responses aggregated from all program cohorts, key information seeking behaviors were noted. The top 60% of survey participants reported health & wellness and medical information is obtained from websites (e.g. WebMD, Healthline, Mayo Clinic), organizations (CDC, American Heart Association), healthcare providers, and word of mouth referrals. Comparatively, from pre to post workshop, an increase of 21% and 33% was evident in the use of databases (e.g. PubMed, MedlinePlus) for health & wellness and medical information respectively. Additionally, social media use for health information seeking decreased 35% for health & wellness topics and 15% for medical concerns. Notably, when attempting to find information about a product, service, or provider, an average of 71% of pre and post survey respondents reportedly seek word of mouth referrals from friends, family, and colleagues. This may indicate the age-old process of information gathering unchanged by extrinsic factors or experiences and, therefore, the necessity of HL awareness and education practices.

Conclusions: The results obtained in this study imply a potential and bigger impact among diverse and larger populations. Although pre-existing knowledge and unrelated factors may have played a role in participant responses, findings can inform and are intended to support future work with other employee wellness programs in corporate, manufacturing, or healthcare environments, undergraduate students in credit bearing courses, and various community groups including high school students, new parents, or older adults.

How Open Are We? An Examination of Open Access Journals within Library and Information Science (RTI112)

Marisa Tutt - Medical Librarian, Island Health, Nanaimo, British Columbia, Canada

Description: Like the patrons we serve, library and information science (LIS) practitioners working outside of academia (e.g., hospital libraries) or in resource-limited settings face barriers to participating in evidence-informed practice. Subscription costs and article processing charges (APCs) limit not only who can participate but how, what, and where knowledge is shared. While most LIS journals offer some sort of open access, many follow a hybrid or green model where large portions of content remain behind paywalls and cost prohibitive APCs and subscription fees restrict access and authorship.

Objective: As part of the 2022-2023 Medical Library Association's Research Training Institute, this research project asks, "How open are LIS journals?" The purpose of this study is to create dialogue and discussion around LIS publishing practices through an analysis of journal websites. Literature shows support for open access within LIS, as do advocacy programs at libraries worldwide, but do our journals?

Methods: This study utilizes a practitioner developed list of English peer-reviewed LIS journals organized topically. Characteristics of each journal are being recorded for analysis including the type of publication model, percentage of open access articles, and fees (subscription and APC).

Results and Conclusions: Results are forthcoming. The answer to the question "how open" lies not in whether or not LIS journals offer the option to publish open but rather in the influencing factors (e.g., cost) and the amount of open access content actually being published.

Impact of COVID-19 on Solo Health Librarian Mentorships (RTI04)

Bridget Jivanelli, AHIP - Medical Librarian, Kim Barrett Memorial Library, HSS Education Institute, New York, New York

[No additional information provided]

Knowing How to See Behind the Data Points: Inclusive Data Ethics Competencies for Health Sciences Librarians (RTI05)

Nancy Shin, AHIP - Scholarly Communications Librarian, Johns Hopkins University, Baltimore, Maryland

Objectives: “Knowing How to See Behind the Data Points: Inclusive Data Ethics Competencies for Health Sciences and Data Librarians” is a research study that explores the competencies of health and data librarians in inclusive data ethics. Assessing the current landscape of ethical competencies for health and data librarians as it relates to inclusive data ethics will impact the fairness and inclusive trajectory of future research done by researchers assisted by these types of librarians. The more that we can reflect the reality of our demographic diversity in medicine, the more effective and impactful medical research will be to society – this is Precision Medicine.

Methods: An inclusive data ethics survey was circulated between February 21st – March 14th, 2023, on both the Medical Library Association (MLA)’s and the Research Data Access and Preservation (RDAP)’s listservs. Survey eligibility required that most of your work was/is done in data or data-related work. The survey asked questions in three parts: data ethics, data skills, and soft skills.

Results: A total of 80 respondents participated in my survey. After cleaning the data, there were 47 complete responses to the survey. Most respondents identified as either a Data Librarian or a Medical Librarian. With regards to data ethics, there was variability in the level of competencies for each of the different ethical skill that was being evaluated. With regards to data skills, most respondents would benefit from an intermediate-advanced and advanced level training among the data skills in this skillset. With regards to soft skills, all respondents would benefit from an advanced level training among the soft skills in this skillset.

Conclusion: With NIH’s 2023 Data Management and Sharing Policy and OSTP’s 2022 Public Access Memo, the political climate will continue to tilt towards more research and data sharing. As such, ethical sharing is going to be more and more important to the researcher and the librarian helping the researcher. This study shows that there is a need to develop more beginner-intermediate, intermediate, and intermediate-advanced level trainings in various data ethics topics more than in data skills or soft skills where content development at an advanced level would be most beneficial to the survey respondents.

Making Room: Clinical Judgement Experiences of Nursing Students in a Hybrid Classroom—Clinical Simulation Design (RTI107)

Carla Fulgham

[No additional information provided]

Maternal Healthcare Outcomes: An Analysis of the Collection of Patient Identifiers (RTI115)

Drew Johnson - Graduate Student, University of North Texas, McKinney, Texas

Black women and AIAN women are more likely than their white counterparts to die from complications during childbirth, regardless of their education level, or socioeconomic status (Artiga et. al., 2022) (See Figure A). While the United States has presented a significant issue of maternal death with 23.8 deaths per 100,000 live births, which is significantly higher than that of other industrialized nations, this statistic has proven to adversely affect the Black and AIAN communities (CDC, 2020). Having adequate health educational materials have been proven to reduce the risks minority mothers face during pregnancy and childbirth, but cannot be effective unless at-risk individuals are identified (CDC, 2020). Currently, there is little research available to demonstrate the adequate identification of expecting mothers to provide relevant care to at-risk suspecting mothers.

Moving DEI Forward: Does Cultural Competence Has a Place in Teaching and Learning? (RTI11)

Alessia Zanin-Yost, AHIP - Health Science Librarian, Bailey Library/ SRU, Slippery Rock, Pennsylvania

Cultural competence in health care (CCHC) is a set of skills that allows an individual or a group to adapt its services to provide optimum care according to unique cultural needs. In the area of librarianship, research focuses on how librarians can develop and provide services to their multicultural patrons and how to integrate cultural competence in library instruction and the library. Through a phenomenological methodology, the author aimed to understand better how health science librarians can position themselves in the CCHC curriculum to support the needs of students and faculty.

Information collected from 30 participants shed some light on how perceptions of collaboration between faculty-librarians may hinder instruction related to CCHC, how CCHC is integrated into the health professions curriculum, and how students understand CCHC.

The results show that while faculty has a high interest in CCHC, there are many obstacles in its integration into the curriculum, among them lack of preparation from faculty, no room for adding new content, and a lack of understanding of who and when CCHC should be taught. Students noted that while CCHC is taught throughout their programs, there is a lack of clinical preparation and in-depth information. Ultimately, this study highlights the need to be more proactive, collaborate, and determine the importance of CCHC to support DEI in academia. Furthermore, the role of the librarian results to be a potential catalyst to link CCHC to students in the health professions.

In My Country, We Don’t Have These: International Students’ Experiences in a Canadian Academic Library (RTI103)

Caitlin Ratcliffe, AHIP - Librarian, Red Deer Polytechnic

How do international students feel in the library? For many international students, libraries in their home countries differ significantly from post-secondary libraries in North America. In Fall 2022 and Winter 2023, we investigated international students’ experiences with the library’s spaces and in-person services at a semi-rural academic library in Canada. This study used the Photovoice method, a combination of photo-

elicitation and focus groups that allows participants to visually represent themselves and share their lived experiences by photographing the world as they see it. Participants responded to prompts identifying which spaces and services in the library made them feel supported, safe, and like they belong in the library, as well as which spaces and services made them feel unsafe or unsupported in the library. Our study found that many international students primarily feel safe and welcome in RDP Library, and appreciated the Library's staff, resources, and spaces. Our study also revealed many of the challenges international students face, including strong feelings of library anxiety (Mellon, 1986), difficulties wayfinding within the library, and uncertainty around how to ask for help, particularly considering common issues with understanding spoken English as an additional language. These findings illustrate how library spaces and services can be further developed to be welcoming and inclusive for a diverse range of patrons.

NIH Public Access Policy After 15 Years: Reviewing Online Communication in Libraries (RTI110)

Kelly Stormking - Library Specialist, St. Jude Children's Research Hospital, Memphis, Tennessee

Objectives: Since 2008, the NIH Public Access Policy has expanded public access by requiring federally-funded researchers to deposit publications in a free online archive. The purpose of this research study is to identify how libraries at NIH-funded institutions communicate the NIH Public Access Policy and related support services offered to researchers.

Methods: Content analysis methodology was used to review websites at the top 100 NIH-funded institutions for 2022, identifying 78 libraries with web-based guides. Each site was reviewed through coding themes of service, education, network and information, with an additional focus on elements of the NIH Public Access Policy.

Results: While many libraries offer general policy information, there are far less variety of services available to researchers. All guides connect to at least one policy site and most provided a policy overview. In contrast, less troubleshooting assistance or policy tool contact information was offered for navigating compliance problems. Conclusions: This research reveals how online promotion is currently conducted, allowing librarians to evaluate their institutions approach to communicating the policy. With the latest 2022 Office of Science and Technology Policy (OSTP) Memo shifting policy requirements, this environmental scan may be useful to libraries revisiting their policy support.

Pivoting When Burn Out Burns Out Your Research Project (RTI06)

Ashley Thomas - Digital Initiatives and Accessibility Librarian, Countway Library of Medicine, Harvard Medical School, Boston, Massachusetts

Occupational burnout does not just permeate one’s work life. It seeps into the personal life as well, infecting projects or exercises one takes out of intellectual curiosity, stimulation, growth, or as a personal challenge. Suddenly, every “extra” is just another overburdened straw on the preverbal broken camel’s back.

There is an exceedingly large amount of shame, guilt, stress, and frustration that comes with burnout. Most often it is internal and internalized. Commitments and deadlines seem like insurmountable obstacles, but they must be dealt with in some capacity. And how that work is accomplished and to what extent varies from person to person, situation to situation.

In this poster, the author discusses their own experience with burnout and how they pivoted when their

original research project became a mental nonstarter. They will provide narrative of how, through finding potential in their own situation, the research is back on track and progressing at a slow, but steady pace.

There have been several surveys conducted to measure librarian burnout, but these studies tend to focus on a particular subgroup of librarians. A study focusing only on health sciences/medical librarians – or even just health sciences/medical libraries – has not been conducted and/or published.

With the United States Congress and President Biden formally concluding the three-year long U.S. national emergency response to the COVID-19 pandemic, there is a distinct and pressing need to understand how health science librarians are doing at the on-set of this “post-pandemic” era. This project seeks to re-examine the burnout levels among library staff workers in health sciences/medical libraries (“staff worker” refers to anyone (non-managerial) at the library who is preforming work/labor under the larger umbrella of librarianship) and understand at what levels library staff are experiencing burnout.

As data collection has not yet begun, the author will gladly welcome any and all advice, suggestions, and general feedback on selecting an appropriate burnout measurement instrument, the process of producing supplemental questions for survey context, and what other librarians and researchers might hope to gain from participating/reading the study.

A Qualitative Study Examining the Research Support Needs of Students in Different Graduate Programs (RTI100)

Donna O'Malley - Research and Instruction Librarian, University of Vermont Charles A. Dana Health Sciences Library, Burlington, Vermont

Objectives: We are interested in understanding how the research needs of UVM graduate students vary depending on their research program requirements and type of research project. The typical graduate student may be seen as someone completing a scholarly dissertation over a period of four or five years. We also have graduate students completing research "projects" for professional doctoral programs, and students in master's programs performing research in an abbreviated period of time.

Methods: We are reviewing the requirements of three different University of Vermont graduate student programs and interviewing graduate students from each program. We are asking questions about challenges students face in completing their research projects, and about resources that did or could have addressed a problem they had. We're also very interested in the degree to which they plan to continue to be involved in research after graduation. At least two researchers have been present for each interview, and interviews are being recorded using Zoom, after which they are transcribed. Some interviews may be conducted remotely. We have begun analyzing the transcripts, developing codes to identify relevant factors in the research environments of these graduate students. We are using Uri Bronfenbrenner's Ecological Systems Theory to organize these factors and understand how they are related.

Results: We have conducted three of the anticipated nine interviews. Sample codes identified so far include Mentors, Journal access, IT environment, and Cost of living.

Conclusions: At this preliminary stage we notice that while in some cases students expressed an obligation to personally address specific factors, students felt that other factors were not their responsibility. Our goal is to highlight commonalities and differences among student research experiences and environments.

A Quantifiable Assessment of Evidence-Based Medicine Instruction Using Case-Based Learning Lab: A Study Proposal (RTI106)

Laura M. Lipke, MS, MLIS, AHIP - Medical Librarian, A. T. Still Memorial Library, Kirksville, Missouri

Osteopathic medical students are expected to develop competency in their ability to independently develop a well-formed, clinically relevant research question and acquire medical evidence to support this question prior to entering residency, according to the Association of Colleges of Osteopathic Medicine (AACOM) (Basehore et al., 2017). As most osteopathic medical schools are at the early stages of implementing evidence-based medicine (EBM) within their curriculum, it follows suit that these students lack the skills to meet these requirements (Guralnick & Yedowitz-Freeman, 2017; Linsenmeyer et al., 2020; McClung et al., 2019; Nicholson et al., 2020; Pearlman et al., 2017).

This proposed experimental study will evaluate the effectiveness of case-based learning (CBL) in the instruction of osteopathic medical students' ability to develop well-formed, clinically relevant questions and acquire medical evidence in support of these questions. Students will be randomly assigned to a traditional lecture-based instruction session and a CBL lab session. The assignment designed for both groups, of 80 students each, will be based on a clinically relevant patient scenario. Participants will be asked to develop a clinical question and design a search strategy to find supporting evidence. These variables of performance will be evaluated by a modified version of the Fresno rubric.

The purpose of this study is to evaluate whether CBL is a more effective instruction method for EBM when compared to a traditional lecture-based instruction method. Significant results from this study will demonstrate that CBL provides context and relevance to learning participants, thus improving their ability to effectively accomplish the first two steps of the EBM process and meet AACOM requirements. These results will also provide evidence to guide future medical librarians in effective EBM instruction.

Student Perceptions of Born-Digital Satellite Library (RTI104)

Karina Kletscher, AHIP - Reference & Instruction Librarian, Creighton University Libraries, Phoenix, Arizona

Background: The born-digital library on the Creighton University Health Sciences Campus – Phoenix is approaching its two-year mark serving its new regional campus. The librarian sought to evaluate current support as well as to understand student perceptions and needs regarding the satellite library. Existing satellite campus literature – particularly centering their libraries – is outdated, leaving little guidance for this born-digital model. The body concerning student information needs and perceptions of library support tends to focus on medicine and nursing; the broader spectrum of professional health sciences, not to mention those on regional campuses, are largely unstudied.

Objectives: 1. Determine students’ perceptions of the services and resources provided by the Health Sciences Library – Phoenix. 2. Identify internal and external factors that enhance or hinder students’ use of services and resources (and perceptions and attitudes). 3. Establish areas of foci to improve student library usage and thus perceptions and attitudes.

Methods: The satellite librarian devised and disseminated an online survey to the Phoenix student body in the Spring 2023 semester. This was inclusive of all active cohorts from the medicine, nursing, occupational therapy, pharmacy, and physical therapy programs. The survey consisted of 11 questions, including both closed and open-ended questions. Of the 524 students, 12% (n=63) responded to the survey. Data analysis

has begun and will include thematic analysis for the qualitative data resulting from the student’s open responses.

Conclusion: This poster will demonstrate the process of designing and implementing a survey instrument for the many disparate cohorts and programs – the first student-centered survey for the campus – as well as share initial findings on use and perceptions of the born-digital satellite library. Considering the gaps and lack of guidance in current literature, this project may be beneficial to the broader conversation on the unique contexts and cultures of satellite campuses, their libraries, and their students, particularly in this increasingly technologically hybrid era.

Student Wellness Initiatives in Jesuit University Libraries (RTI09)

Claire O. Sharifi - Reference Librarian, Liaison to School of Nursing & Health Professions, University of San Francisco, San Francisco, California

Objectives: There’s a growing need for student wellness support on all university campuses, including the 28 institutions that compose the Association of Jesuit Colleges and Universities (AJCU). Wellbeing is not just the absence of physical or mental health concerns, it is a multidimensional concept that includes mental, physical, spiritual, environmental, and other dimensions that, individually or collectively, impact a variety of student outcomes. Student wellbeing should be addressed at the campus level, not siloed in specific departments and units. Libraries are accessible, student-centric spaces, and often the physical and figurative center of a university campus. Librarians and library staff value engagement and are responsive to the user’s changing needs. As such, libraries and those that work in libraries are well positioned to support student wellness initiatives. This study investigates the nature and extent of AJCU library support for student wellness initiatives.

Methods: Semi-structured qualitative interviews were completed via Zoom with librarians at AJCU libraries. Hettler’s Six Dimensions of Wellness and the Jesuit concept of Cura Personalis serve as the conceptual framework. This in-progress study is utilizing thematic analysis to identify themes across interviews.

Results: Very preliminary analysis points to a few emerging themes, including libraries as hubs for campus collaboration, the ongoing impacts of COVID, and student-centric ethos.

Trying Libraries on for Size: Mapping the Anti-fatness Literature in Library Science (RTI07)

Emily Gilbert - Assistant Professor and Information Services & Liaison Librarian, University of Illinois Chicago

This scoping review analyzed the available literature on how libraries perpetuate anti-fatness in their physical spaces, collections, catalogs, and treatment of fat patrons and library workers. The author searched databases and grey literature, tracked citations in included works, and found one source by happenstance. Ultimately 251 written works across different publication channels were reviewed, and only six works met the inclusion criteria of discussing anti-fatness as related to libraries. The included literature denotes a throughline of discrimination against fat people in libraries, whether focusing on library workers, patrons, physical spaces, collections and cataloging, or the catalog and metadata. More research is needed on anti-fatness in libraries to better create an equitable environment for patrons and library workers of all sizes.

Working Toward a Lasting Impression: A Survey Protocol to Measure Recall of Undergraduate Nursing Library Instruction (RTI10)

Jason Wardell - Health & Life Sciences Librarian, University of Dayton, Dayton, Ohio

Background: As information-seeking specialists, we are regularly called-upon to provide instruction on identifying high-quality, evidence-based research at various points in an undergraduate health sciences student's academic career. Much research has been done on improving information recall in undergraduate health sciences students as it pertains to clinical competency, and similarly, research exists that addresses a gap in evidence-based information-seeking skills in Medical School students. There has been little research to determine the timing and content of health science information-seeking instruction, nor the staying power of such instruction. Cognitive Load Theory (CLT) is a conceptual framework that may shed some light on what materials are retained and what are lost over the course of an undergraduate program.

Objectives: This study aims to ascertain both the recall of information within a single cohort of undergraduate nursing students and longitudinally between their first-year introductory course and their third-year research methods course, with a goal of better understanding our students' information-seeking needs and competencies. Applying the CLT framework, we will identify areas of information literacy instruction requiring reinforcement throughout an undergraduate health sciences curriculum and whether much of our traditional library instruction would be better suited if delivered in a "just in time" model rather than "just in case." Additionally, we will measure the effects of library instruction on negative attitudes toward research and literature searching.

Methods: The prospective research will take place in Fall 2023 with two test groups of undergraduate nursing students, group A in their first year of study, and group B in their third. The survey—including basic questions on information literacy, qualities of evidence-based research, search mechanics, and general attitudes toward research and information-seeking—will be administered one day in advance of scheduled library information-seeking instruction for both groups, and again three weeks following the session, with all questions randomized at the start of each survey. The resulting effect size data will be analyzed for change using an average-based statistical methodology, and any change in attitudes over time will be measured using the NRC Emotion Lexicon for sentiment analysis.

Future considerations: For best results, this study will be conducted annually for a minimum of three years, to ensure a single cohort of students is followed from their first to their third year. Additionally, the current selection of survey points is one of convenience: the author currently has existing relationships with the nursing faculty instructors, and the library instruction sessions are consistently timed year-over-year. Future research will need to be done among other disciplines, as what may be true for undergraduate nursing students may not be the same in other fields. Lastly, this is not a prescriptive study and will not deliver results showing best practices or potential education interventions. Future research will need to be cognizant of any changes made to instruction over time.
